# A DFT Study on the Excited Electronic States of Cyanopolyynes: Benchmarks and Applications

**DOI:** 10.3390/molecules27185829

**Published:** 2022-09-08

**Authors:** Marcin Gronowski, Robert Kołos

**Affiliations:** Institute of Physical Chemistry, Polish Academy of Sciences, Kasprzaka 44/52, 01-224 Warsaw, Poland

**Keywords:** polyynes, nitriles, TD-DFT, oligomers, nanowire, spectroscopy, electronic states, orbitals, quantum chemistry, vibrational frequencies

## Abstract

Highly unsaturated chain molecules are interesting due to their potential application as nanowires and occurrence in interstellar space. Here, we focus on predicting the electronic spectra of polyynic nitriles HC_2*m*+1_N (*m* = 0–13) and dinitriles NC_2*n*+2_N (*n* = 0–14). The results of time-dependent density functional theory (TD-DFT) calculations are compared with the available gas-phase and noble gas matrix experimental data. We assessed the performance of fifteen functionals and five basis sets for reproducing (i) vibrationless electronic excitation energies and (ii) vibrational frequencies in the singlet excited states. We found that the basis sets of at least triple-ζ quality were necessary to describe the long molecules with alternate single and triple bonds. Vibrational frequency scaling factors are similar for the ground and excited states. The benchmarked spectroscopic parameters were shown to be acceptably reproduced with adequately chosen functionals, in particular ωB97X, CAM-B3LYP, B3LYP, B971, and B972. Select functionals were applied to study the electronic excitation of molecules up to HC_27_N and C_30_N_2_. It is demonstrated that optical excitation leads to a shift from the polyyne- to a cumulene-like electronic structure.

## 1. Introduction

One of the challenges of modern quantum chemistry is to develop an approach that would make the predictions on the excited electronic states simple, reliable, and relatively cheap. The most precise results are obtained by ab initio electronic structure methods, which are computationally demanding. Conversely, a popular, inexpensive method that certainly lacks the accuracy of advanced ab initio approaches is the time-dependent density functional theory (TD-DFT). It assumes that electronic density slowly varies over time, allowing for the direct application of density functionals developed for ground-state calculations [1]. That approximation, the adopted linear response formalism, and the lack of the exact ground state functional are the main deficiencies of TD-DFT. Additionally, the use of extended orbital basis sets is usually impossible. As a result, proper testing of DFT methods is crucial for their subsequent application.

It may seem that a statistical comparison of the calculation results with experimental data should not pose any problems. However, in the case of electronic transitions, where both the electronic and vibrational contributions shape the spectrum, selecting the appropriate reference data is not trivial. Large-scale testing projects are constrained by the necessity of relatively time-consuming optimization of excited state molecular geometries. Additionally, a large part of experimental data come from the measurement of solutions, and the solvent always modifies the spectrum, sometimes very significantly. TD DFT performance benchmarks obtained for the gas-phase atomic excitation energies [2], where experimental data are well known, are hardly transferrable to molecular species, in particular the polyatomic ones. Alternatively, reference data for vertical electronic excitations of the molecules can be obtained ab initio [3,4,5] and then used to test various DFT methods [6], although the adequacy of theoretically derived reference databases may be a nontrivial problem [7]. Energetic separation between the vibrationless levels of different electronic states (“0-0 transition” energy) is a popular benchmark in electronic spectroscopy. Some studies [7,8,9,10] gave a good insight into the accuracy of the corresponding theoretical predictions, but the number of the considered molecules was rather limited.

An extensive vibrational structure is a common feature of electronic spectra, in both absorption and emission. For smaller molecules, vibrational frequencies can be provided by anharmonic calculations with high-level ab initio methods. The same is usually not feasible for larger systems. Interestingly, DFT-level predictions of ground-state vibrational frequencies often provide qualitatively similar agreement with the experiment, irrespective of whether an anharmonic or scaled harmonic approach is applied [11]. Scaling factors routinely applied to harmonic values depend on the functional and basis set, but a typical value for the ground electronic states is close to 0.96 [12,13], According to Furche and Ahlrichs [14], the TD-DFT-derived excited-state vibrational frequencies are of “remarkably high quality, which is comparable to that obtained in ground state density functional calculations.” The question remains whether the same factors can be recommended for scaling the ground- and excited-state frequencies obtained with TD-DFT.

**Figure 1:**
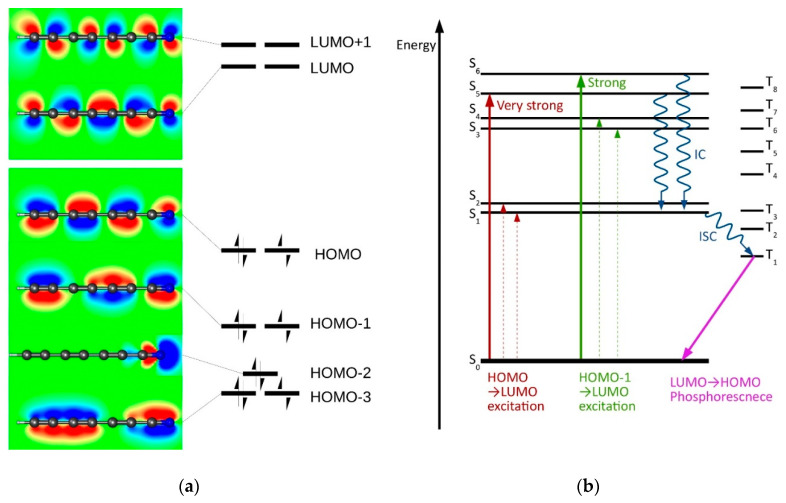
Photophysics of polyynes, illustrated with the example of HC_7_N. (**a**) The predicted contours of molecular orbitals together with a scheme of ground-state orbital occupation; (**b**) Jablonski diagram. Dashed arrows represent weak, formally forbidden transitions.

This work focuses on polyynic nitriles HC_2*m*+1_N and dinitriles NC_2*n*+2_N, the linear molecules featuring alternate single and triple bonds [15]. Henceforth, for brevity, the name “cyanopolyyne” (abbreviated as CPY) is used for both homologous series. These are of astrochemical significance [16,17] and, as postulated in a report on NC_16_N [18], may find applications as the semiconducting nanowires. To date, the longest well-defined, spectroscopically analyzed polyynic chain is -C_22_- capped on both sides with the bulky tris(3,5-di-*t*-butylphenyl)methyl groups [19]. The effect of terminal substituents on the properties of a long, rod-like carbon backbone is diminishing exponentially with the chain length [20]. Such molecules may therefore serve as suitable models for linear carbon structures with tunable optical, mechanical, and electronic properties [21]. Their nonlinear optical properties will likely have applications in optoelectronic devices [22,23,24]. From that perspective, it was appealing to use CPY family molecules as test species for the assessment of TD-DFT performance in reproducing (i) energetics of the lowest excited electronic states and (ii) the corresponding vibrational frequencies. The CPY family is also worth of attention. For some of these molecules, importantly, transition energies from the ground, S_0_, to the lowest excited singlet states, S_1_, S_2_, have been measured, often along with the singlet-triplet (S_0_-T_1_) gap. The S_1_-S_0_ and S_2_-S_0_ transitions, forbidden by symmetry of electronic wavefunctions, can be detected due to the Herzberg–Teller coupling with π-symmetry vibrations. Although the lowest-lying electronic states may not be observed in absorption experiments, they play a significant role in the electronic relaxation. Internal conversion (IC) leads to S_1_, from where an intersystem crossing (ISC) to T_1_ (Figure 1) is possible. Hydrogen-capped polyynes are a good example here. Their allowed transitions S*_n_*-S_0_ (*n* > 2), in particular ^1^Σ^+^–^1^Σ^+^, are often known [25,26,27,28,29,30,31], but the relaxation proceeds through largely unexplored S_1_ and T_1_ states [32].

In this work, we computed the electronic excitation energies for long (*n* up to 14, *m* up to 13) CPYs, including those that could not, to date, have been measured. We preceded these predictions with benchmark studies on some shorter species, in order to select the most promising functional for that class of molecules. Additionally, scaling factors for vibrational frequencies in the excited electronic states could be estimated here for the first time.

## 2. Results and Discussion

### 2.1. Training Sets

Our CPY training set comprises all available experimental gas-phase vibrational frequencies and the energies of 0-0 transitions linking the ground state with the two lowest excited singlet states and with the lowest triplet [33,34,35,36,37,38,39,40,41,42,43,44]. Gas-phase spectroscopic measurements are primarily available for short cyanopolyynes (HCN, HC_3_N, HC_5_N, C_2_N_2_, C_4_N_2_). Noble gas (Ar, Kr) matrix experiments provided information on the longer ones (HC_5_N, HC_7_N, HC_9_N, C_4_N_2_, C_6_N_2_, C_8_N_2_, C_10_N_2_) [44,45,46,47,48,49,50,51,52,53,54,55]. These cryogenic solid environments are not completely inert, but their effect on the energies of electronic transitions is small, compared to the expected inaccuracies of computational methods. Our experimental reference data can be divided into three separate training sets, namely (i) gas-phase: concerning the singlets S_1_ and S_2_ (denoted also as $\tilde{A}$ and $\tilde{B}$, respectively), (ii) Kr-matrix: concerning mostly the S_2_ states, (iii) Ar matrix concerning only the triplet T_1_ (denoted also $\tilde{a}$).

### 2.2. Electronic Excitation Energy

For a given state i, we define the percent error between the theoretical $E_{i}^{t}$ and experimental $E_{i}^{e}$ excitation energy as:

(1) $$\Delta_{i}=100\frac{E_{i}^{t}-E_{i}^{e}}{E_{i}^{e}}$$

For the whole training set encompassing the data for n excited states, we define:

1. mean percent error:
  (2) $$\bar{\Delta }=\frac{1}{n}\overset{n}{\underset{i=1}{\sum }}\Delta_{i}$$
2. mean square percent error:
  (3) $$\Delta_{st}=\sqrt{\frac{1}{n-1}\overset{n}{\underset{i=1}{\sum }}{\left(\Delta_{i}-\bar{\Delta }\right)}^{2}}=\sqrt{\frac{1}{n-1}\left(\left(\overset{n}{\underset{i=1}{\sum }}\Delta_{i}^{2}\right)-n{\bar{\Delta }}^{2}\right)}$$
3. mean absolute percent error:
  (4) $${\bar{\Delta }}_{abs}=\frac{1}{n}\overset{n}{\underset{i=1}{\sum }}\left|\Delta_{i}\right|$$

**Figure 2:**
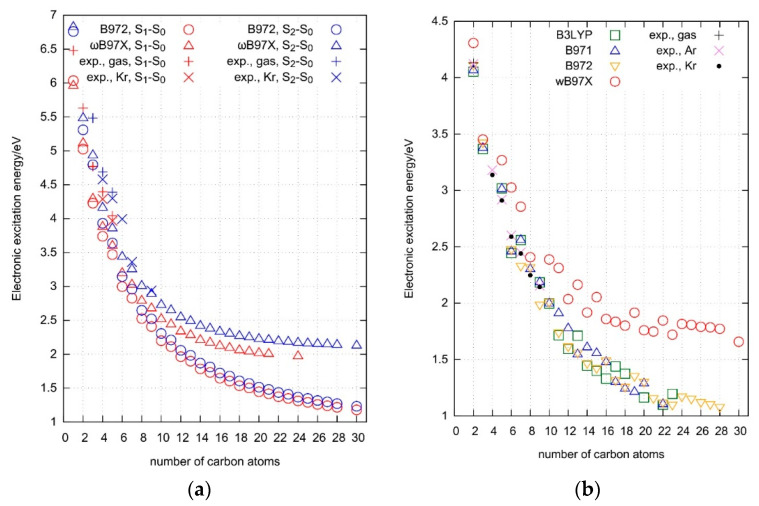
Vibrationless (0-0) excitation energy values plotted against the number of carbon atoms; (**a**) S_1_-S_0_ and S_2_-S_0_; (**b**) T_1_-S_0_. All calculations performed with the aug-cc-pVTZ basis set; results for the other functionals and basis sets are provided as the Supplementary Materials (Tables S4–S85).

Table 1 and Table 2 list these statistical parameters for our TD-DFT computations, while Figure 2 shows the S_1_-S_0_ and S_2_-S_0_ excitation energies derived with select functionals, together with the respective experimental values. There, regardless of the functional and basis set, the computed singlet-singlet excitation energies are lower by about 10% than the experimental ones. Underestimation of electronic excitation energy by TD-B3LYP was already reported for some polyynes [56] and for a variety of extended π-electronic systems [57]. This observation is rather generally applicable to TD DFT predictions, at least for the appropriately selected basis sets; similar conclusions were drawn from our study on atomic excitation energies [2] and from the comparison of TD-DFT results with high-quality ab initio benchmark data [6]. As anticipated, the accuracy of calculations grew with the size of the applied orbital basis set (i.e., we did not observe any error-cancelation effects that would produce an opposite trend). The smallest Dunning-type basis set, aug-cc-pVDZ, tended to provide slightly bent structures (rather than the linear ones), in disagreement with the results derived with more extensive basis sets and, most importantly, contradicting the available experimental data. In these cases, forcing a linear symmetry led to the appearance of imaginary frequencies. Consequently, we do not recommend the use of aug-cc-pVDZ in the calculations of excited electronic states of polyynic chains. On the other hand, no statistically meaningful difference appeared between the triple-ζ quality basis sets aug-cc-pVTZ and def2-TZVPD. The above remarks concerning the influence of the orbital basis set on the quality of TD-DFT-derived results do not entirely agree with what was recently reported by the Head-Gordon’s group [6]. We associate that discrepancy with entirely different training sets and the nature of the test. Combining the observations from both studies, we can safely recommend the use of aug-cc-pVTZ in TD-DFT analyses of excited state potential energy surfaces.

**Figure 3:**
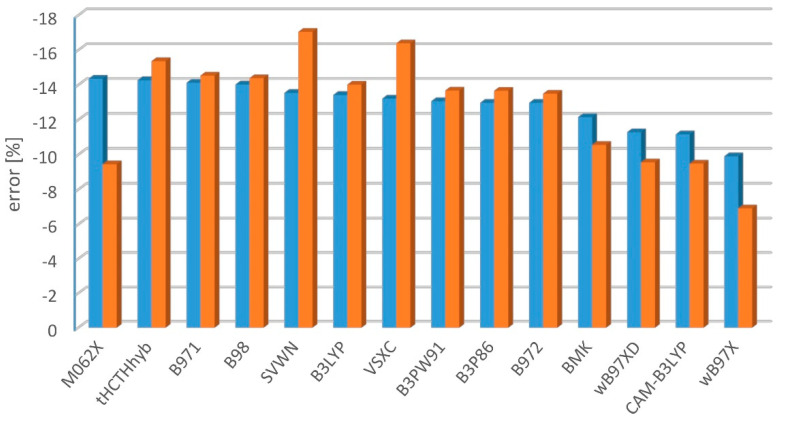
Mean percent error of TD-DFT/aug-cc-pVTZ-derived singlet-singlet excitation energy predictions, with respect to the experimental gas-phase (blue) or Kr-matrix (orange) values.

**Figure 4:**
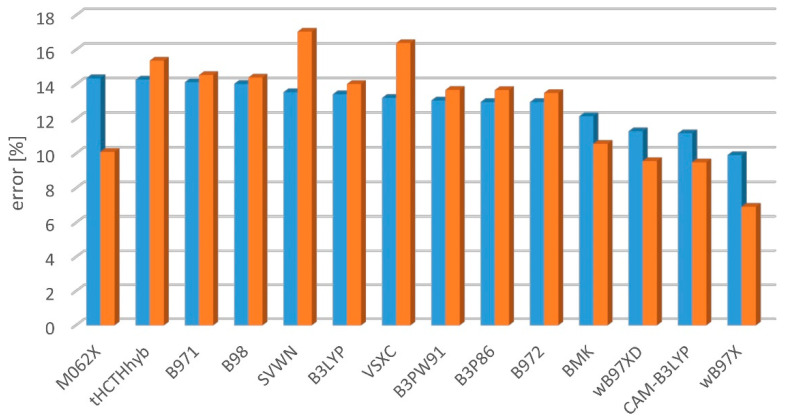
Mean absolute percent error of TD-DFT/aug-cc-pVTZ-derived singlet-singlet excitation energy predictions, with respect to the experimental gas-phase (blue) or Kr-matrix (orange) values.

The ωB97X significantly outperforms (for the S_1_-S_0_ and S_2_-S_0_ excitation) the other tested functionals regardless of the applied training set (Figure 3 and Figure 4). However, the ωB97XD, CAM-B3LYP, and BMK seem to be the reasonable choices as well. The good performance of CAM-B3LYP, BMK, and of ωB97-family functionals was expected based on the previous works [2,6,58]. The biggest surprise is the performance of M06-2X, which seems to be good when compared with the results from Kr-matrix and bad when compared with gas-phase data. Matrix-induced shifts of 0-0 transitions are usually much smaller than the difference between the experimental and TD-DFT results. Here, however, M06-2X describes the S_2_ states (Δ symmetry) perfectly well, but it encounters a massive problem with the S_1_ states (Σ^−^ symmetry). An equivocal behavior of M06-2X stems from the number of S_1_ states being small in the Kr training set. M06-2X is a hybrid-meta GGA functional, of high rank in the DFT Jacob’s ladder, performing well in other recent benchmark computations [6,10]. That case demonstrates that an a priori estimation of the accuracy of TD-DFT excitation energy is challenging and, regardless of global tests for various molecules and states, it is advisable to perform specifically addressed benchmark studies.

**Figure 5:**
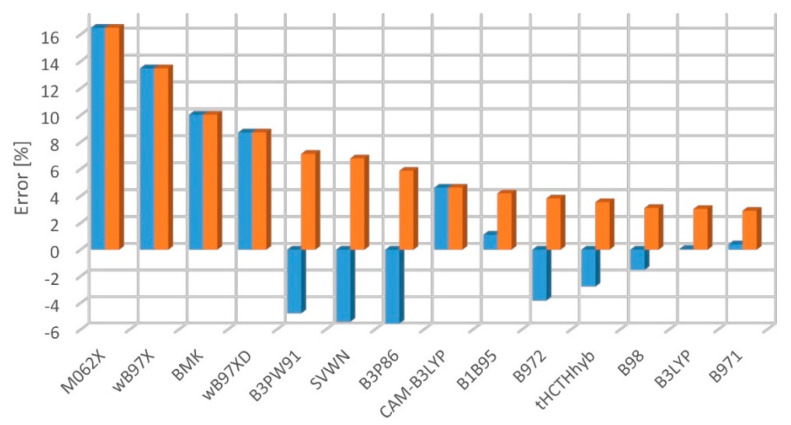
Mean percent difference (blue) and mean absolute percent error (orange) for the DFT/aug-cc-pVTZ-derived singlet-triplet excitation energy predictions, with respect to the experimental Ar-matrix values.

The above-revealed hierarchy of methods is by far not preserved for the singlet-triplet splitting (Table 3, Figure 5). The functionals BMK, ωB97X, and M06-2X are among the worst options, while B3LYP and B971 perform very well. The T_1_-S_0_ gap is in general satisfactorily predicted, for the majority of functionals the error being less than 10%, i.e., smaller than in singlet-singlet excitation energy calculations. This is not surprising, considering that functionals are mostly designed for predicting the energy of the lowest state of a given multiplicity. Noteworthy, M06-2X performs poorly once again, even though it was shown to give excellent results for many aromatic conjugated systems [59]. The good performance of B3LYP is in line with the findings reported by Nguyen et al. [60] who predicted the energies of excited triplet states. Present results for the triplets should be treated with caution, however. The energies and geometric parameters meet the required convergence criteria, but the singlet-triplet splitting is not monotonically decreasing with the size of a carbon chain. We attribute that “noise” to the instability of the obtained triplet-state electronic wavefunction. This shows that even the simplest excited-state calculations do not work like a “black box”. An appropriate multi-reference method (CASPT2 type) would likely remedy such instability problems, but the cost, for large systems, can be prohibitive.

### 2.3. Vibrational Frequencies

For the comparison of theoretically derived harmonic vibrational frequencies $\omega_{j}$ with experimental fundamental frequencies $\nu_{j}$, a linear relationship was assumed:

(5) $$\nu_{j}=f\omega_{j}$$

The least squares fitting procedure supplies the mean value of *f*:

(6) $$\bar{f}=\frac{\sum_{j=1}^{M}\nu_{j}\omega_{j}}{\sum_{j=1}^{M}\omega_{j}^{2}}$$

for a set of M normal modes.

The following two statistical parameters were calculated:

- root mean square error
  (7) $$RMS=\sqrt{\frac{1}{M}\overset{M}{\underset{j=1}{\sum }}{\left(\bar{f}\;\omega_{j}-\nu_{j}\right)}^{2}}$$
- uncertainty of $\bar{f}$
  (8) $$s=\sqrt{\frac{M}{M-2}\frac{RMS^{2}}{\sum_{j=1}^{M}{\left(\omega_{j}-\bar{\omega }\right)}^{2}}}$$

The $\bar{f}$, s, and RMS values have been obtained for the whole training set. Separately, analysis of the bending modes only (for the linear structures) supplied ${\bar{f}}_{b}$, $s_{b},$ and $RMS_{b}$.

**Figure 6:**
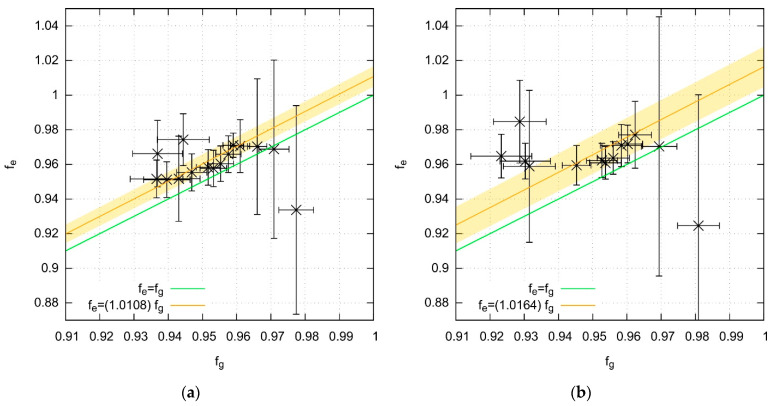
Correlation between the vibrational frequency scaling factors obtained for the ground electronic states, *f*_g_, and for the excited states, *f*_e_. Orange line represents the best fit of the *f*_e_ = *slope* × *f*_g_ function. Yellow strap marks the 3σ confidence interval (the green line corresponding to *slope* = 1 falls out of 3σ). Computations were performed with all functionals listed in Table 4 and Table 5 and with the aug-cc-pVTZ basis set. (**a**) Reference data taken from gas-phase measurements; the uncertainty of *slope* value is 0.0019. (**b**) Reference data taken from Kr-matrix measurements; the uncertainty of *slope* value is 0.004.

The results are collected in Table 4 and Table 5 and in Supplementary Materials (Tables S116–S119). Differences between the ground- and excited-state scaling factors are generally small and are superimposed on the uncertainty of the determined $\bar{f}$ coefficients. However, as illustrated by Figure 6, scaling factors are slightly (on the order of 1%) higher in the excited than in the ground states for almost all functionals studied (irrespective of whether the reference data came from the gas phase or from the Kr matrix measurements). We are cautious about the generalization of that finding. The present database of vibrational frequencies is small, and it rarely includes both ground and excited state frequencies for a given vibrational mode. Additionally, the uncertainties inherent (due to a limited spectral resolution) to the experimental determinations are often larger for the excited states than for the ground states and may even approach the *RMS* errors. Finally, only one specific class of molecules has been tested here, and, to the best of our knowledge, there exist no published reports devoted to the scaling of excited-state harmonic vibrational frequencies. We can nevertheless confirm the conclusion formulated by Furche and Ahlrichs [14] that the qualities of ground and excited state vibrational frequency computations are similar. It should also be remarked that the overall performance of functionals applied here to ground-state vibrational spectroscopy predictions is not significantly different from what was previously reported [12,13].

### 2.4. Recommendations

Presently collected data and the published results unable us to formulate certain recommendations for the TD-DFT calculations on cyanopolyyne-family molecules. These can likely be extended also to other polyynes. Our study shows, however, that it is necessary to test a given TD-DFT method for a select class of molecules and electronic states, without relying too heavily on benchmark results reported for other species.

While it is not possible to indicate a functional perfectly suited for the description of both the ground and the lowest excited states of the tested molecules, the use of ωB97X and CAM-B3LYP can be advised when the goal consists in predicting the singlet-singlet excitation energies. The above two functionals, however, are not the best choice when it comes to the computation of triplet-singlet spacings or the ground-state vibrational frequencies. B3LYP or B971 should rather be applied for these tasks, but then the description of excited singlet states would suffer. The B972 functional seems to produce the most balanced results. It works decently well in predicting the excitation energies to singlet states, the excited singlet state vibrational frequencies, singlet-triplet splittings, and the ground-state vibrational spectroscopy. The appropriate choice of a basis set is either aug-cc-pVTZ or def2TZVPD. Figure S1 illustrates how the calculation method may affect the predicted band positions and the associated oscillator strengths. It should be remarked, however, that neither the inclusion of important effects coming from the vibronic coupling nor predictions of the vibrational structure of electronic transitions were within the scope of this work.

### 2.5. Electronic Excitation Energy of Longer Cyanoacetylenes

**Figure 7:**
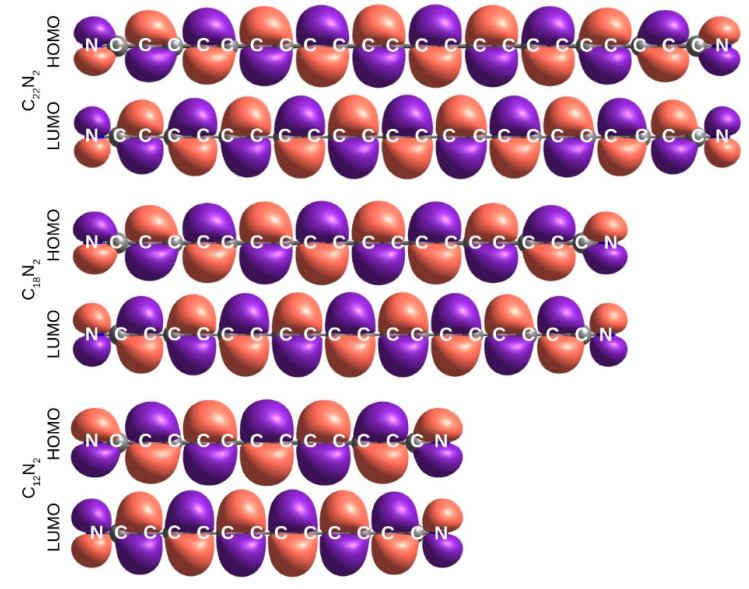
HOMO and LUMO molecular orbitals of select C_2*n*+2_N_2_ molecules (B972/aug-cc-pVTZ). The unabridged figure is available in Supplementary Materials (Figure S2).

**Figure 8:**
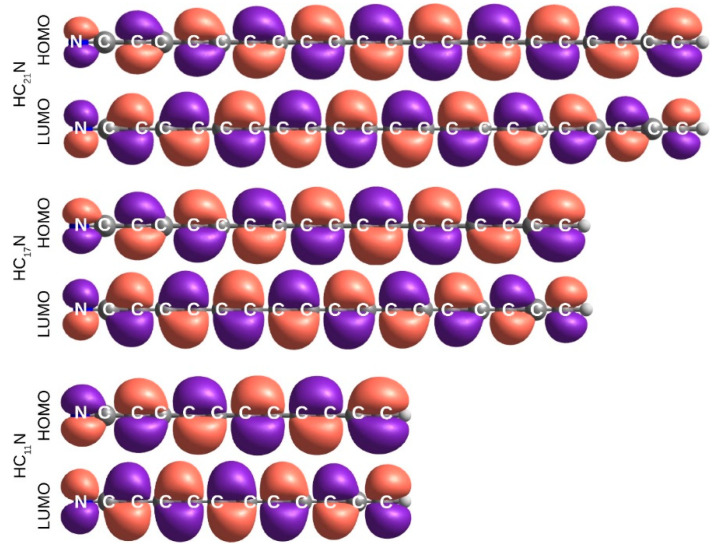
HOMO and LUMO molecular orbitals of select HC_2*n*+1_N molecules (B972/aug-cc-pVTZ). The unabridged figure is available in Supplementary Materials (Figure S3).

**Figure 9:**
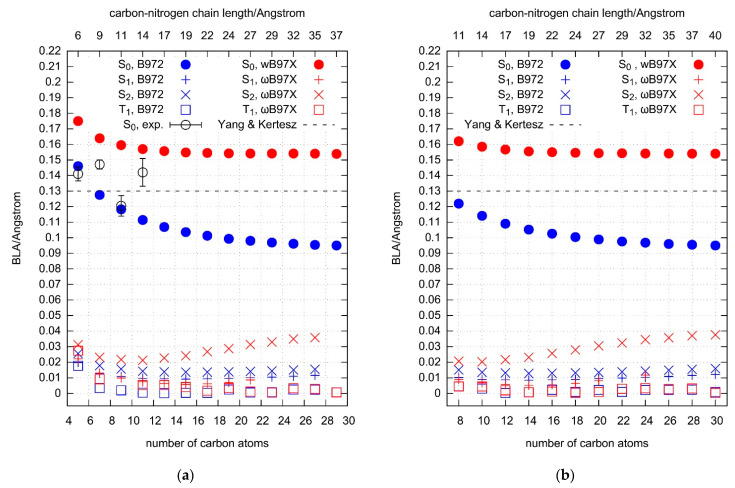
Bond length alternation plotted as a function of the number of carbon atoms in HC_2*n*+1_N (**a**) and C_2*n*+2_N_2_ (**b**) families. The upper horizontal scale gives approximate lengths of the carbon-nitrogen chains. The black dash horizontal line represents the BLA value estimated for a long polyyne [64]. Experimental values (with 1σ experimental uncertainty) are based on microwave spectroscopic measurements of the ground vibrational states [65].

The S_1_-S_0_, S_2_-S_0_, and T_1_-S_0_ transitions are adequately described in terms of HOMO-LUMO excitations. The HOMO and LUMO orbitals, π and π*, respectively (Figure 7 and Figure 8), do not exhibit any helical allene-like [61,62] arrangement. Electron density maxima in HOMOs correspond to the triple bond positions. The shape of LUMOs is unusual. At the molecular extremities LUMO resembles typical π-antibonding orbitals; inside the chain, however, it behaves rather like a π-bonding, with electron density maxima coinciding with the locations of single bonds. This indicates that the HOMO-LUMO excitations cause a shift from the polyyne-like structure towards a cumulenic one. Such a conjecture is confirmed with the observed bond length alternation (BLA; defined as the difference in length of the neighboring bonds near the center of the molecule [21]). For the ground state, as can be seen in Figure 9, even the longest chains show substantial BLA. After the electronic excitation, BLA drops down drastically (a similar theoretical result was reported for hexayne [32]). In T_1_, it oscillates around 0.003 Å, and in the S_1_ and S_2_ states it is around 0.01 Å. Such low values are characteristic for cumulenes and allenes [63].

B972/aug-cc-pVTZ computations lead to ground-state BLA in the vicinity of 0.1 Å. Similar predictions were reported for variously capped polyynes [63,66]. The B3LYP result for a chain oligomer C_72_ is 0.088 Å [64], i.e., close to 0.095 Å obtained here for C_30_N_2_. The recommended BLA of a long polyyne chain [64] falls between the values currently predicted by B972 and ωB97X for the chains of the length 3–4 nm. Experimental bond lengths, available for HC_5_N, HC_7_N, HC_9_N, HC_11_N [65], also lead to the BLA confined between the B972- and ωB97X-derived values, usually closer to B972. As can be seen in Figure 9, the experimental BLA does not necessarily change monotonically with the size of the carbon chain, unlike the predictions for S_0_. However, these latter refer to the equilibrium bond lengths, i.e., the ones at the minimum of the potential energy surface. The experiment, on the other hand, supplies vibrationally averaged values for the ground vibrational state. That difference may alter the BLA. Nevertheless, the disagreement between theory and experiment is only a bit higher than the typical accuracy of BLA predictions accomplished with the DFT methods [67].

The transition from polyyne to cumulene structure is visible in the vibrational spectroscopy as well. Frequencies (Tables S117–S168) assigned to the triple bond stretching modes decrease after the electronic excitation. An opposite comportment is observed for the single bond stretches.

Significant changes in the electronic structure produced upon excitation may be of practical importance. A polyyne molecule can be classified, in its ground state, as a semiconductor. Singlet-triplet separation is 1–2 eV for the chain lengths of 4–1.5 nm, and the electrical conductivity likely decreases for longer molecules [68]. The cumulene chains, on the other hand, behave as electrical conductors and that property increases with their length [68]. Using light to toggle between the two structures is a challenge. So far, it has been demonstrated, to a limited extent, for a C_12_ polyyne capped with the adamantyl groups [69]. Figure 2 illustrates a decline in electronic excitation energies with the increasing molecular size, well known from the other studies on polyynes [18,56,70,71]. 

## 3. Materials and Methods

Gaussian 09 Rev. B.01 [72] was applied for benchmark computations and Gaussian 16 Rev. B.01 [73] for studying the longest CPYs. Singlet-state excitation energies were calculated using the standard TD-DFT approach [14,74,75,76,77]. The S_0_-T_1_ energy gaps were obtained as ΔSCF, i.e., as the difference in energies of the lowest triplet and singlet states. The triplet energy was given by unrestricted DFT computations (α and β orbitals relaxed independently). The benchmark studies used the XQC option, which automatically switched from the default DIIS-type [78,79] to a quadratically convergent [80] SCF technique in case of a numerical problem. For longer molecules, where the XQC procedure did not converge, the YQC algorithm was employed. In all calculations (i) two-electron integrals were computed with pruned UltraFine grid and (ii) tight convergences criteria were applied for geometry optimization and electronic energy computations.

We tested the three-parameter Becke hybrid functional [81] with various non-local correlation parts (B3LYP [82], B3PW91 [83], B3P86 [84]), one-parameter Becke hybrid functional (B1B95 [85]), revised versions of B97 [86] (B98 [87], B971 [88], B972 [89]), high-nonlocality functional of Zhao and Truhlar (M06-2X [90]), Local Spin Density functional (SVWN [91,92]), τ-dependent gradient-corrected correlation functionals (tHCTHhyb [93], BMK [94], VSXC [95]), long-range-corrected (CAM-B3LYP [96], ωB97X [97], also in the version with an empirical dispersion correction [98]: (ωB97XD [99]). All functionals were used as implemented in Gaussian. The correlation consistent basis sets aug-cc-pVDZ and aug-cc-pVTZ [100,101] came from the Gaussian database, whereas the def2 sets [102,103] were downloaded from the Basis Set Exchange Library [104,105,106].

Computations were carried out at our local computer cluster equipped with Intel Xeon CPU E5-1660 v4 and Intel Xeon CPU E3-1240 processors.

## 4. Conclusions

The performance of DFT functionals and basis sets has been benchmarked with respect to electronic spectroscopy predictions for cyanopolyynes. While we cannot indicate a single functional ideally suited for describing both the ground and the lowest excited electronic states, B972 generally provided reasonably balanced results. The ωB97X and CAM-B3LYP functionals are, however, a better choice for the derivation of singlet-singlet excitation energies, whereas B3LYP and B971 should rather be applied for predicting the triplet-singlet spacing values or the ground-state vibrational frequencies. The aug-cc-pVTZ and def2TZVPD basis sets offer a good compromise between the cost and quality of computations.

Present results provide the functional- and basis set-dependent scaling factors for the correction of vibrational frequencies obtained with the harmonic approximation. Our recommendation is to use the same scaling factors for the ground and for the excited electronic states.

This study shows that DFT calculations should be preceded by appropriate benchmarking with regard to the class of molecules and electronic states under study. Even then, the results are subject to considerable uncertainty.

Our predictions pertaining to relatively long cyanopolyynes indicate that their optical excitation leads to a shift from the polyyne- to cumulene-like electronic structures. These latter structures conduct electricity, which is of possible interest for the design of future nanowires. We expect the first electronic states of 1.5–4 nm long molecules (C_12_N_2_, C_14_N_2_, C_16_N_2_, C_18_N_2_, C_20_N_2_, C_22_N_2_, C_24_N_2_, C_26_N_2_, C_30_N_2_, HC_13_N, HC_15_N, HC_19_N, HC_21_N, HC_23_N, HC_25_N, HC_27_N) to be excited with green-orange light, while phosphorescence may occur in the red or near-infrared range.

## Figures and Tables

**Table 1:** Mean percent errors (with respect to the measured gas-phase values) of the singlet-singlet excitation energy calculations performed with various functional/basis set combinations. $\bar{\Delta }$ is defined by Equation (2). See Table S1 for $\Delta_{st}$, and ${\bar{\Delta }}_{abs}$ (defined by Equations (3) and (4)).

	aug-cc-pVDZ	aug-cc-pVTZ	def2SVPD	def2TZVPD	def2QZVPD
**tHCTHhyb**	−16.1	−14.28	−14.87	−14.28	−14.13
**VSXC**	−14.53	−13.22	−13.62	−13.41	−13.04
**BMK**	−14.93	−12.16	−13.71	−12.53	−11.88
**B1B95**	−14.64	−12.73	−13.19	−12.96	−12.9
**B3P86**	−14.79	−12.98	−13.63	−13	−12.91
**B98**	−15.98	−14.03	−14.76	−14.08	−13.92
**B971**	−16.01	−14.13	−14.81	−14.17	−14.03
**B972**	−14.61	−12.98	−13.43	−12.97	−12.88
**wB97X**	−11.7	−9.91	−10.52	−9.97	−9.83
**wB97XD**	−13.19	−11.29	−11.97	−11.37	−11.16
**CAM-B3LYP**	−13.14	−11.17	−11.9	−11.19	−11.04
**M062X**	−16.13	−14.36	−14.99	−14.02	−14.06
**SVWN**	−15.57	−13.55	−14.4	−14.16	-
**B3PW91**	−14.83	−13.07	−13.67	−13.08	−12.98
**B3LYP**	−15.78	−13.43	−14.59	−13.9	−13.81

**Table 2:** Mean percent errors (with respect to the measured Kr-matrix values) of the singlet-singlet excitation energy calculations performed with various functional/basis set combinations. See Table S2 for $\Delta_{st}$, and ${\bar{\Delta }}_{abs}$ (defined by Equations (3) and (4)).

	aug-cc-pVDZ	aug-cc-pVTZ	def2SVPD	def2TZVPD	def2QZVPD
**tHCTHhyb**	−17.0	−15.4	−16.3	−15.5	−14.9
**VSXC**	−16.8	−16.4	−16.5	−16.5	-
**BMK**	−12.6	−10.6	−12.0	−10.5	-
**B1B95**	−14.0	−12.6	−13.2	−12.6	−12.8
**B3P86**	−15.3	−13.7	−14.6	−13.8	−13.5
**B98**	−16.2	−14.4	−15.5	−14.6	−14.2
**B971**	−16.3	−14.5	−15.6	−14.9	−14.3
**B972**	−14.9	−13.5	−14.3	−13.6	−13.3
**wB97X**	−8.5	−6.9	−7.9	−7.3	−7.9
**wB97XD**	−11.3	−9.6	−10.6	−9.8	−10.1
**CAM-B3LYP**	−11.4	−9.5	−10.5	−9.6	−10.0
**M062X**	−10.8	−9.5	−10.3	−9.1	−11.2
**SVWN**	−18.3	−17.1	−17.6	−17.7	-
**B3PW91**	−15.2	−13.7	−14.6	−13.8	-
**B3LYP**	−16.1	−14.0	−15.4	−14.5	−14.2

**Table 3:** Mean percent errors (with respect to the measured Ar-matrix values) of the singlet-triplet excitation energy calculations performed with various functional/basis set combinations. See Table S3 for $\Delta_{st}$, and ${\bar{\Delta }}_{abs}$ (defined by Equations (3) and (4)).

	aug-cc-pVDZ	aug-cc-pVTZ	def2SVPD	def2TZVPD
**tHCTHhyb**	−7.77	−2.76	−4.31	−4.52
**VSXC**	−12.81	-	−13.44	−14.51
**BMK**	4.41	10.04	8.31	10.42
**B1B95**	-0.37	1.12	5.42	1.96
**B3P86**	−6.02	−5.55	−3.63	−5.66
**B98**	−1.47	−1.52	−3.51	−5.10
**B971**	0.49	0.40	−0.72	0.09
**B972**	−2.13	−3.82	−5.26	−1.80
**wB97X**	6.96	13.48	6.79	15.5
**wB97XD**	6.41	8.72	6.80	6.45
**CAM-B3LYP**	5.96	4.62	4.83	6.82
**M062X**	16.3	16.49	16.73	18.14
**SVWN**	−4.84	−5.41	−5.52	−5.56
**B3PW91**	−2.84	−4.77	−6.14	−4.42
**B3LYP**	−3.72	0.05	−3.07	−3.6

**Table 4:** Scaling factors for fundamental vibrational frequencies in the lowest excited singlet states (S_1_ and S_2_). Reference values taken from gas-phase experiments.

	aug-cc-pVTZ						def2-SVPD						def2-TZVPD					
	$\bar{f}$	s	RMS	${\bar{f}}_{b}$	$s_{b}$	$RMS_{b}$	$\bar{f}$	s	RMS	${\bar{f}}_{b}$	$s_{b}$	$RMS_{b}$	$\bar{f}$	s	RMS	${\bar{f}}_{b}$	$s_{b}$	$RMS_{b}$
**tHCTHhyb**	0.97	0.039	78	0.91	0.31	100	0.937	0.031	80	0.753	0.086	53	0.976	0.01	24	1.007	0.078	26
**VSXC**	0.969	0.051	108	0.925	0.113	39	0.946	0.024	61	0.787	0.051	28	n/a	n/a	n/a	n/a	n/a	n/a
**BMK**	0.974	0.015	26	n/a	n/a	n/a	0.92	0.038	98	0.701	0.082	55	0.965	0.011	29	0.937	0.072	32
**B1B95**	0.955	0.011	28	0.914	0.071	29	0.929	0.025	65	0.77	0.082	44	0.955	0.011	28	0.921	0.066	30
**B3P86**	0.958	0.01	28	0.921	0.067	29	0.923	0.031	82	0.735	0.079	49	0.957	0.011	28	0.924	0.067	30
**B98**	0.971	0.007	17	0.979	0.054	18	0.932	0.03	79	0.749	0.082	50	0.968	0.01	28	0.95	0.073	32
**B971**	0.971	0.015	30	0.938	0.113	36	0.936	0.028	74	0.763	0.082	48	0.972	0.008	21	0.994	0.069	23
**B972**	0.958	0.01	27	0.925	0.068	30	0.929	0.026	67	0.772	0.077	45	0.958	0.01	27	0.932	0.069	31
**wB97X**	0.952	0.011	29	0.888	0.048	22	0.925	0.025	66	0.765	0.061	35	0.95	0.011	30	0.886	0.052	25
**wB97XD**	0.952	0.025	66	0.885	0.175	79	0.922	0.028	75	0.746	0.071	43	0.956	0.007	18	0.936	0.046	16
**CAM-B3LYP**	0.951	0.01	27	0.892	0.049	23	0.917	0.031	81	0.725	0.066	40	0.949	0.011	29	0.886	0.052	24
**M062X**	0.966	0.019	50	0.883	0.055	26	0.934	0.034	90	0.74	0.064	38	0.965	0.019	51	0.884	0.056	26
**SVWN**	0.934	0.06	119	0.68	0.14	82	0.913	0.048	125	0.658	0.097	72	0.976	0.038	43	n/a	n/a	n/a
**B3PW91**	0.96	0.01	27	0.925	0.066	29	0.925	0.03	80	0.741	0.079	49	0.96	0.011	28	0.927	0.068	31
**B3LYP**	0.966	0.011	28	0.922	0.072	29	0.93	0.031	82	0.739	0.077	47	0.964	0.011	28	0.926	0.067	30

**Table 5:** Scaling factors for fundamental vibrational frequencies of S_0_ states. Reference values taken from gas-phase experiments.

	aug-cc-pVTZ						def2-SVPD						def2-TZVPD					
	$\bar{f}$	s	RMS	${\bar{f}}_{b}$	$s_{b}$	$RMS_{b}$	$\bar{f}$	s	RMS	${\bar{f}}_{b}$	$s_{b}$	$RMS_{b}$	$\bar{f}$	s	RMS	${\bar{f}}_{b}$	$s_{b}$	$RMS_{b}$
**tHCTHhyb**	0.966	0.003	13	0.939	0.023	14	0.954	0.021	98	0.781	0.145	109	0.966	0.002	11	0.945	0.017	11
**VSXC**	0.971	0.004	21	0.947	0.023	13	0.961	0.018	84	0.82	0.139	99	0.971	0.004	19	0.953	0.019	11
**BMK**	0.944	0.008	37	0.902	0.039	25	0.933	0.022	106	0.75	0.14	109	0.943	0.006	29	0.912	0.028	18
**B1B95**	0.947	0.004	21	0.905	0.025	16	0.936	0.018	88	0.784	0.127	96	0.947	0.004	18	0.912	0.018	11
**B3P86**	0.953	0.004	19	0.913	0.026	17	0.942	0.021	99	0.766	0.139	107	0.953	0.003	16	0.919	0.02	12
**B98**	0.959	0.003	14	0.93	0.02	13	0.948	0.02	92	0.786	0.137	102	0.958	0.003	12	0.934	0.017	10
**B971**	0.961	0.003	13	0.933	0.019	12	0.95	0.019	88	0.796	0.135	99	0.961	0.002	11	0.937	0.016	10
**B972**	0.952	0.003	17	0.912	0.021	13	0.941	0.018	86	0.791	0.129	97	0.952	0.003	13	0.921	0.012	8
**wB97X**	0.937	0.008	37	0.881	0.031	20	0.928	0.018	87	0.785	0.115	87	0.937	0.007	35	0.889	0.023	15
**wB97XD**	0.943	0.006	30	0.895	0.03	20	0.934	0.02	94	0.774	0.129	99	0.943	0.006	27	0.905	0.02	13
**CAM-B3LYP**	0.94	0.007	33	0.89	0.029	19	0.93	0.019	90	0.776	0.12	90	0.939	0.007	32	0.893	0.025	16
**M062X**	0.937	0.007	36	0.885	0.03	20	0.927	0.018	86	0.786	0.115	86	0.936	0.007	33	0.893	0.021	14
**SVWN**	0.977	0.005	24	0.947	0.038	23	0.955	0.034	156	0.68	0.17	153	0.976	0.006	26	0.941	0.044	26
**B3PW91**	0.955	0.004	18	0.915	0.025	16	0.943	0.021	99	0.768	0.139	107	0.955	0.003	15	0.921	0.019	12
**B3LYP**	0.958	0.004	18	0.918	0.024	15	0.947	0.019	91	0.782	0.132	98	0.957	0.003	16	0.921	0.021	13

## Data Availability

See Supplementary Materials.

## Supplementary Materials

The following supporting information can be downloaded at: https://www.mdpi.com/article/10.3390/molecules27185829/s1, Figure S1: Oscillator strength vs. wavelength graphics, as predicted for HC_7_N with several TD DFT methods; Figure S2: Extended version of Figure 7; Figure S3: Extended version of Figure 8; Table S1: Full version of Table 1; Table S2: Full version of Table 2; Table S3: Full version of Table 3; Tables S4–S85: Electronic excitation energies predicted by various combinations of basis sets and functionals; Tables S86–S115: Harmonic vibrational frequencies predicted by various combinations of basis sets and functionals (shorter molecules); Tables S116–S119: Harmonic vibrational frequency scaling factors; Tables S120–S171: Harmonic vibrational frequencies predicted by various combinations of basis sets and functionals (longer molecules).
