# Supplementary material for: A DFT Study on the Excited Electronic States of Cyanopolyynes: Benchmarks and Applications
Source: Molecules. 2022 Sep 8;27(18):5829. doi: 10.3390/molecules27185829 (PMC9500640; doi:10.3390/molecules27185829)
Supplement: Supplementary file 1 [file molecules-27-05829-s001.zip › molecules-1855585-supplementary.pdf]

Supplementary Materials to article: A DFT study on the excited electronic states of cyanopolyynes. Benchmarks and applications.

Marcin Gronowski* and Robert Kołos
Institute of Physical Chemistry Polish Academy of Sciences, Kasprzaka 44/52, 01-224 Warsaw, Poland

July 2022

List of Figures

S1 TD-DFT results for HC_7N. Energies of the vertical electronic excitations from S_0 to S_1-S_5 states and of the vibrationless (0-0) transitions from S_0 to S_1,S_2. Ordinate axis values represent the oscillator strength for vertical transitions. Triple-zeta basis sets were employed in all computations. The experimentally derived wavelength of the vibrationless S_2-S_0 transition is marked with a gray line. . . . 3
S2 HOMO and LUMO molecular orbitals of $C_{2n+2}N_2$ molecules (B972/aug-cc-pVTZ). . . . 3
S3 HOMO and LUMO molecular orbitals of $HC_{2n+1}N$ molecules (B972/aug-cc-pVTZ). . . . 4

List of Tables

S1 Mean percent errors (with respect to the measured gas-phase values) of the singlet-singlet excitation energy calculations performed with various functional / basis set combinations. $\bar{\Delta}$, Δ_{st}, $\bar{\Delta}_{abs}$ are defined by Eqs. 2-5. . . . 5
S2 Mean percent errors (with respect to the measured Kr-matrix values) of the singlet-singlet excitation energy calculations performed with various functional / basis set combinations. $\bar{\Delta}$, Δ_{st}, $\bar{\Delta}_{abs}$ are defined by Eqs. 2-5. . . . 5
S3 Mean percent errors (with respect to the measured Ar-matrix values) of the singlet-triplet excitation energy calculations performed with various functional / basis set combinations. $\bar{\Delta}$, Δ_{st}, $\bar{\Delta}_{abs}$ are defined by Eqs. 2-5. . . . 5
S4 Energy separation (eV) between the ground vibrational states of S_0 and S_1, calculated for HC_1N. Experimental value(s): 6.48 eV (gas) [1] . . . 5
S5 Energy separation (eV) between the ground vibrational states of S_0 and S_2, calculated for HC_1N. Experimental value(s): 7.1 eV (gas) [2] . . . 5
S6 Energy separation (eV) between the ground vibrational states of S_0 and T_1, calculated for HC_1N. Experimental value(s): 4.9 eV (gas) [2] . . . 5
S7 Energy separation (eV) between the ground vibrational states of S_0 and S_1, calculated for HC_3N. Experimental value(s): 4.77 eV (gas) [3] . . . 5
S8 Energy separation (eV) between the ground vibrational states of S_0 and S_2, calculated for HC_3N. Experimental value(s): 5.484 eV (gas) [4] . . . 5
S9 Energy separation (eV) between the ground vibrational states of S_0 and T_1, calculated for HC_3N. . . . 5
S10 Energy separation (eV) between the ground vibrational states of S_0 and S_1, calculated for HC_5N. Experimental value(s): 4.041 eV (gas) [5] , 3.983 eV (Ar) [5] , 3.968 eV (Kr) [5] . . . 5
S11 Energy separation (eV) between the ground vibrational states of S_0 and S_2, calculated for HC_5N. Experimental value(s): 4.389 eV (gas) [5] , 4.316 eV (Ar) [5] , 4.298 eV (Kr) [5] . . . 5
S12 Energy separation (eV) between the ground vibrational states of S_0 and T_1, calculated for HC_5N. Experimental value(s): 2.919 eV (Ar) [5] , 2.911 eV (Kr) [5] . . . 6
S13 Energy separation (eV) between the ground vibrational states of S_0 and S_1, calculated for HC_7N. . . . 6
S14 Energy separation (eV) between the ground vibrational states of S_0 and S_2, calculated for HC_7N. Experimental value(s): 3.36 eV (Kr) [6] . . . 6
S15 Energy separation (eV) between the ground vibrational states of S_0 and T_1, calculated for HC_7N. Experimental value(s): 2.4504 eV (Ar) [6] , 2.4412 eV (Kr) [6] . . . 6
S16 Energy separation (eV) between the ground vibrational states of S_0 and S_1, calculated for HC_9N. . . . 6
S17 Energy separation (eV) between the ground vibrational states of S_0 and S_2, calculated for HC_9N. Experimental value(s): 2.938 eV (Ar) [7] , 2.94 eV (Kr) [7] . . . 6
S18 Energy separation (eV) between the ground vibrational states of S_0 and T_1, calculated for HC_9N. Experimental value(s): 2.1547 eV (Ar) [7] , 2.1449 eV (Kr) [7] . . . 6
S19 Energy separation (eV) between the ground vibrational states of S_0 and S_1, calculated for C_2N_2. Experimental value(s): 5.63 eV (gas) [8] . . . 7
S20 Energy separation (eV) between the ground vibrational states of S_0 and S_2, calculated for C_2N_2. . . . 7
S21 Energy separation (eV) between the ground vibrational states of S_0 and T_1, calculated for C_2N_2. Experimental value(s): 4.13 eV (gas) [9] , 4.1219 eV (Ar) [10] . . . 7
S22 Energy separation (eV) between the ground vibrational states of S_0 and S_1, calculated for C_4N_2. Experimental value(s): 4.3966 eV (gas) [11] , 4.288 eV (Kr) [12] . . . 7
S23 Energy separation (eV) between the ground vibrational states of S_0 and S_2, calculated for C_4N_2. Experimental value(s): 4.688 eV (gas) [13] , 4.578 eV (Kr) [12] . . . 7
S24 Energy separation (eV) between the ground vibrational states of S_0 and T_1, calculated for C_4N_2. Experimental value(s): 3.181 eV (Ar) [14] , 3.138 eV (Kr) [14] . . . 7
S25 Energy separation (eV) between the ground vibrational states of S_0 and S_1, calculated for C_6N_2. . . . 7
S26 Energy separation (eV) between the ground vibrational states of S_0 and S_2, calculated for C_6N_2. Experimental value(s): 3.994 eV (gas) [15] . . . 7
S27 Energy separation (eV) between the ground vibrational states of S_0 and T_1, calculated for C_6N_2. Experimental value(s): 2.601 eV (Ar) [16] , 2.590 eV (Kr) [16] . . . 7
S28 Energy separation (eV) between the ground vibrational states of S_0 and S_1, calculated for C_8N_2. . . . 7
S29 Energy separation (eV) between the ground vibrational states of S_0 and S_2, calculated for C_8N_2. . . . 7
S30 Energy separation (eV) between the ground vibrational states of S_0 and T_1, calculated for C_8N_2. Experimental value(s): 2.248 eV (Kr) [17] . . . 8
S31 Energy separation (eV) between the ground vibrational states of S_0 and S_1, calculated for $C_{10}N_2$. . . . 8
S32 Energy separation (eV) between the ground vibrational states of S_0 and S_2, calculated for $C_{10}N_2$. . . . 8
S33 Energy separation (eV) between the ground vibrational states of S_0 and T_1, calculated for $C_{10}N_2$. Experimental value(s): 2.024 eV (Ar) [18] , 2.006 eV (Kr) [18] . . . 8
S34 Energy separation (eV) between the ground vibrational states of S_0 and S_1, calculated for $HC_{11}N$. . . . 9
S35 Energy separation (eV) between the ground vibrational states of S_0 and S_2, calculated for $HC_{11}N$. . . . 9
S36 Energy separation (eV) between the ground vibrational states of S_0 and T_1, calculated for $HC_{11}N$. . . . 9
S37 Energy separation (eV) between the ground vibrational states of S_0 and S_1, calculated for $HC_{13}N$. . . . 9
S38 Energy separation (eV) between the ground vibrational states of S_0 and S_2, calculated for $HC_{13}N$. . . . 9
S39 Energy separation (eV) between the ground vibrational states of S_0 and T_1, calculated for $HC_{13}N$. . . . 9
S40 Energy separation (eV) between the ground vibrational states of S_0 and S_1, calculated for $HC_{15}N$. . . . 9
S41 Energy separation (eV) between the ground vibrational states of S_0 and S_2, calculated for $HC_{15}N$. . . . 9
S42 Energy separation (eV) between the ground vibrational states of S_0 and T_1, calculated for $HC_{15}N$. . . . 9
S43 Energy separation (eV) between the ground vibrational states of S_0 and S_1, calculated for $HC_{17}N$. . . . 9
S44 Energy separation (eV) between the ground vibrational states of S_0 and S_2, calculated for $HC_{17}N$. . . . 9
S45 Energy separation (eV) between the ground vibrational states of S_0 and T_1, calculated for $HC_{17}N$. . . . 9
S46 Energy separation (eV) between the ground vibrational states of S_0 and S_1, calculated for $HC_{19}N$. . . . 9
S47 Energy separation (eV) between the ground vibrational states of S_0 and S_2, calculated for $HC_{19}N$. . . . 9
S48 Energy separation (eV) between the ground vibrational states of S_0 and T_1, calculated for $HC_{19}N$. . . . 9
S49 Energy separation (eV) between the ground vibrational states of S_0 and S_1, calculated for $HC_{21}N$. . . . 9
S50 Energy separation (eV) between the ground vibrational states of S_0 and S_2, calculated for $HC_{21}N$. . . . 9
S51 Energy separation (eV) between the ground vibrational states of S_0 and T_1, calculated for $HC_{21}N$. . . . 9
S52 Energy separation (eV) between the ground vibrational states of S_0 and T_1, calculated for $HC_{23}N$. . . . 9
S53 Energy separation (eV) between the ground vibrational states of S_0 and T_1, calculated for $HC_{25}N$. . . . 9
S54 Energy separation (eV) between the ground vibrational states of S_0 and T_1, calculated for $HC_{27}N$. . . . 9
S55 Energy separation (eV) between the ground vibrational states of S_0 and T_1, calculated for $HC_{29}N$. . . . 9
S56 Energy separation (eV) between the ground vibrational states of S_0 and S_1, calculated for $C_{12}N_2$. . . . 10
S57 Energy separation (eV) between the ground vibrational states of S_0 and S_2, calculated for $C_{12}N_2$. . . . 10
S58 Energy separation (eV) between the ground vibrational states of S_0 and T_1, calculated for $C_{12}N_2$. . . . 10
S59 Energy separation (eV) between the ground vibrational states of S_0 and S_1, calculated for $C_{14}N_2$. . . . 10
S60 Energy separation (eV) between the ground vibrational states of S_0 and S_2, calculated for $C_{14}N_2$. . . . 10
S61 Energy separation (eV) between the ground vibrational states of S_0 and T_1, calculated for $C_{14}N_2$. . . . 10
S62 Energy separation (eV) between the ground vibrational states of S_0 and S_1, calculated for $C_{16}N_2$. . . . 10
S63 Energy separation (eV) between the ground vibrational states of S_0 and S_2, calculated for $C_{16}N_2$. . . . 10
S64 Energy separation (eV) between the ground vibrational states of S_0 and T_1, calculated for $C_{16}N_2$. . . . 10
S65 Energy separation (eV) between the ground vibrational states of S_0 and S_1, calculated for $C_{18}N_2$. . . . 10
S66 Energy separation (eV) between the ground vibrational states of S_0 and S_2, calculated for $C_{18}N_2$. . . . 10
S67 Energy separation (eV) between the ground vibrational states of S_0 and T_1, calculated for $C_{18}N_2$. . . . 10
S68 Energy separation (eV) between the ground vibrational states of S_0 and S_1, calculated for $C_{20}N_2$. . . . 10
S69 Energy separation (eV) between the ground vibrational states of S_0 and S_2, calculated for $C_{20}N_2$. . . . 10
S70 Energy separation (eV) between the ground vibrational states of S_0 and T_1, calculated for $C_{20}N_2$. . . . 10
S71 Energy separation (eV) between the ground vibrational states of S_0 and S_1, calculated for $C_{22}N_2$. . . . 10
S72 Energy separation (eV) between the ground vibrational states of S_0 and S_2, calculated for $C_{22}N_2$. . . . 10
S73 Energy separation (eV) between the ground vibrational states of S_0 and T_1, calculated for $C_{22}N_2$. . . . 10
S74 Energy separation (eV) between the ground vibrational states of S_0 and S_1, calculated for $C_{24}N_2$. . . . 10
S75 Energy separation (eV) between the ground vibrational states of S_0 and S_2, calculated for $C_{24}N_2$. . . . 10
S76 Energy separation (eV) between the ground vibrational states of S_0 and T_1, calculated for $C_{24}N_2$. . . . 10
S77 Energy separation (eV) between the ground vibrational states of S_0 and S_1, calculated for $C_{26}N_2$. . . . 10
S78 Energy separation (eV) between the ground vibrational states of S_0 and S_2, calculated for $C_{26}N_2$. . . . 10
S79 Energy separation (eV) between the ground vibrational states of S_0 and T_1, calculated for $C_{26}N_2$. . . . 10
S80 Energy separation (eV) between the ground vibrational states of S_0 and S_1, calculated for $C_{28}N_2$. . . . 10
S81 Energy separation (eV) between the ground vibrational states of S_0 and S_2, calculated for $C_{28}N_2$. . . . 10
S82 Energy separation (eV) between the ground vibrational states of S_0 and T_1, calculated for $C_{28}N_2$. . . . 10
S83 Energy separation (eV) between the ground vibrational states of S_0 and S_1, calculated for $C_{30}N_2$. . . . 10
S84 Energy separation (eV) between the ground vibrational states of S_0 and S_2, calculated for $C_{30}N_2$. . . . 10
S85 Energy separation (eV) between the ground vibrational states of S_0 and T_1, calculated for $C_{30}N_2$. . . . 10
S86 Harmonic unscaled vibrational frequencies (in cm^{-1}) for S_1 of HC_1N . . . 11
S87 Harmonic unscaled vibrational frequencies (in cm^{-1}) for S_2 of HC_1N . . . 11
S88 Harmonic unscaled vibrational frequencies (in cm^{-1}) for S_0 of HC_1N . . . 12
S89 Harmonic unscaled vibrational frequencies (in cm^{-1}) for S_1 of HC_3N . . . 12
S90 Harmonic unscaled vibrational frequencies (in cm^{-1}) for S_2 of HC_3N . . . 13
S91 Harmonic unscaled vibrational frequencies (in cm^{-1}) for S_0 of HC_3N . . . 13
S92 Harmonic unscaled vibrational frequencies (in cm^{-1}) for S_1 of HC_5N . . . 14
S93 Harmonic unscaled vibrational frequencies (in cm^{-1}) for S_2 of HC_5N . . . 14
S94 Harmonic unscaled vibrational frequencies (in cm^{-1}) for S_0 of HC_5N . . . 15
S95 Harmonic unscaled vibrational frequencies (in cm^{-1}) for S_1 of HC_7N . . . 15
S96 Harmonic unscaled vibrational frequencies (in cm^{-1}) for S_2 of HC_7N . . . 16
S97 Harmonic unscaled vibrational frequencies (in cm^{-1}) for S_0 of HC_7N . . . 16
S98 Harmonic unscaled vibrational frequencies (in cm^{-1}) for S_1 of HC_9N . . . 16
S99 Harmonic unscaled vibrational frequencies (in cm^{-1}) for S_2 of HC_9N . . . 17
S100 Harmonic unscaled vibrational frequencies (in cm^{-1}) for S_0 of HC_9N . . . 17
S101 Harmonic unscaled vibrational frequencies (in cm^{-1}) for S_1 of C_2N_2 . . . 18
S102 Harmonic unscaled vibrational frequencies (in cm^{-1}) for S_2 of C_2N_2 . . . 18
S103 Harmonic unscaled vibrational frequencies (in cm^{-1}) for S_0 of C_2N_2 . . . 19
S104 Harmonic unscaled vibrational frequencies (in cm^{-1}) for S_1 of C_4N_2 . . . 19
S105 Harmonic unscaled vibrational frequencies (in cm^{-1}) for S_2 of C_4N_2 . . . 20
S106 Harmonic unscaled vibrational frequencies (in cm^{-1}) for S_0 of C_4N_2 . . . 20
S107 Harmonic unscaled vibrational frequencies (in cm^{-1}) for S_1 of C_6N_2 . . . 21
S108 Harmonic unscaled vibrational frequencies (in cm^{-1}) for S_2 of C_6N_2 . . . 21
S109 Harmonic unscaled vibrational frequencies (in cm^{-1}) for S_0 of C_6N_2 . . . 22
S110 Harmonic unscaled vibrational frequencies (in cm^{-1}) for S_1 of C_8N_2 . . . 22
S111 Harmonic unscaled vibrational frequencies (in cm^{-1}) for S_2 of C_8N_2 . . . 22
S112 Harmonic unscaled vibrational frequencies (in cm^{-1}) for S_0 of C_8N_2 . . . 23
S113 Harmonic unscaled vibrational frequencies (in cm^{-1}) for S_1 of $C_{10}N_2$ . . . 23
S114 Harmonic unscaled vibrational frequencies (in cm^{-1}) for S_2 of $C_{10}N_2$ . . . 23
S115 Harmonic unscaled vibrational frequencies (in cm^{-1}) for S_0 of $C_{10}N_2$ . . . 24
S116 Scaling factors for fundamental vibrational frequencies in excited electronic states. Experimental values measured in gas phase . . . 25
S117 Scaling factors for fundamental vibrational frequencies in the ground electronic state. Experimental values measured in gas phase . . . 25
S118 Scaling factors for fundamental vibrational frequencies in excited electronic states. Experimental values measured in solid Kr . . . 25
S119 Scaling factors for fundamental vibrational frequencies in the ground electronic state. Experimental values measured in solid Kr . . . 25
S120 Harmonic unscaled vibrational frequencies (in cm^{-1}) for S_1 of $HC_{11}N$ . . . 26
S121 Harmonic unscaled vibrational frequencies (in cm^{-1}) for S_2 of $HC_{11}N$ . . . 26
S122 Harmonic unscaled vibrational frequencies (in cm^{-1}) for S_0 of $HC_{11}N$ . . . 26
S123 Harmonic unscaled vibrational frequencies (in cm^{-1}) for S_1 of $HC_{13}N$ . . . 26
S124 Harmonic unscaled vibrational frequencies (in cm^{-1}) for S_2 of $HC_{13}N$ . . . 26
S125 Harmonic unscaled vibrational frequencies (in cm^{-1}) for S_0 of $HC_{13}N$ . . . 26
S126 Harmonic unscaled vibrational frequencies (in cm^{-1}) for S_1 of $HC_{15}N$ . . . 26
S127 Harmonic unscaled vibrational frequencies (in cm^{-1}) for S_2 of $HC_{15}N$ . . . 26
S128 Harmonic unscaled vibrational frequencies (in cm^{-1}) for S_0 of $HC_{15}N$ . . . 26
S129 Harmonic unscaled vibrational frequencies (in cm^{-1}) for S_1 of $HC_{17}N$ . . . 26
S130 Harmonic unscaled vibrational frequencies (in cm^{-1}) for S_2 of $HC_{17}N$ . . . 26
S131 Harmonic unscaled vibrational frequencies (in cm^{-1}) for S_0 of $HC_{17}N$ . . . 26
S132 Harmonic unscaled vibrational frequencies (in cm^{-1}) for S_1 of $HC_{19}N$ . . . 26
S133 Harmonic unscaled vibrational frequencies (in cm^{-1}) for S_2 of $HC_{19}N$ . . . 26
S134 Harmonic unscaled vibrational frequencies (in cm^{-1}) for S_0 of $HC_{19}N$ . . . 26
S135 Harmonic unscaled vibrational frequencies (in cm^{-1}) for S_1 of $HC_{21}N$ . . . 26
S136 Harmonic unscaled vibrational frequencies (in cm^{-1}) for S_2 of $HC_{21}N$ . . . 26
S137 Harmonic unscaled vibrational frequencies (in cm^{-1}) for S_0 of $HC_{21}N$ . . . 26
S138 Harmonic unscaled vibrational frequencies (in cm^{-1}) for S_0 of $HC_{23}N$ . . . 26
S139 Harmonic unscaled vibrational frequencies (in cm^{-1}) for S_0 of $HC_{25}N$ . . . 26
S140 Harmonic unscaled vibrational frequencies (in cm^{-1}) for S_0 of $HC_{27}N$ . . . 26
S141 Harmonic unscaled vibrational frequencies (in cm^{-1}) for S_0 of $HC_{29}N$ . . . 26
S142 Harmonic unscaled vibrational frequencies (in cm^{-1}) for S_1 of $C_{12}N_2$ . . . 27
S143 Harmonic unscaled vibrational frequencies (in cm^{-1}) for S_2 of $C_{12}N_2$ . . . 27
S144 Harmonic unscaled vibrational frequencies (in cm^{-1}) for S_0 of $C_{12}N_2$ . . . 27
S145 Harmonic unscaled vibrational frequencies (in cm^{-1}) for S_1 of $C_{14}N_2$ . . . 27
S146 Harmonic unscaled vibrational frequencies (in cm^{-1}) for S_2 of $C_{14}N_2$ . . . 27
S147 Harmonic unscaled vibrational frequencies (in cm^{-1}) for S_0 of $C_{14}N_2$ . . . 27
S148 Harmonic unscaled vibrational frequencies (in cm^{-1}) for S_1 of $C_{16}N_2$ . . . 27
S149 Harmonic unscaled vibrational frequencies (in cm^{-1}) for S_2 of $C_{16}N_2$ . . . 27
S150 Harmonic unscaled vibrational frequencies (in cm^{-1}) for S_0 of $C_{16}N_2$ . . . 27
S151 Harmonic unscaled vibrational frequencies (in cm^{-1}) for S_1 of $C_{18}N_2$ . . . 27
S152 Harmonic unscaled vibrational frequencies (in cm^{-1}) for S_2 of $C_{18}N_2$ . . . 27
S153 Harmonic unscaled vibrational frequencies (in cm^{-1}) for S_0 of $C_{18}N_2$ . . . 27
S154 Harmonic unscaled vibrational frequencies (in cm^{-1}) for S_1 of $C_{20}N_2$ . . . 27
S155 Harmonic unscaled vibrational frequencies (in cm^{-1}) for S_2 of $C_{20}N_2$ . . . 27
S156 Harmonic unscaled vibrational frequencies (in cm^{-1}) for S_0 of $C_{20}N_2$ . . . 27
S157 Harmonic unscaled vibrational frequencies (in cm^{-1}) for S_1 of $C_{22}N_2$ . . . 27
S158 Harmonic unscaled vibrational frequencies (in cm^{-1}) for S_2 of $C_{22}N_2$ . . . 27
S159 Harmonic unscaled vibrational frequencies (in cm^{-1}) for S_0 of $C_{22}N_2$ . . . 27
S160 Harmonic unscaled vibrational frequencies (in cm^{-1}) for S_1 of $C_{24}N_2$ . . . 27
S161 Harmonic unscaled vibrational frequencies (in cm^{-1}) for S_2 of $C_{24}N_2$ . . . 27
S162 Harmonic unscaled vibrational frequencies (in cm^{-1}) for S_0 of $C_{24}N_2$ . . . 27
S163 Harmonic unscaled vibrational frequencies (in cm^{-1}) for S_1 of $C_{26}N_2$ . . . 27
S164 Harmonic unscaled vibrational frequencies (in cm^{-1}) for S_2 of $C_{26}N_2$ . . . 27
S165 Harmonic unscaled vibrational frequencies (in cm^{-1}) for S_0 of $C_{26}N_2$ . . . 27
S166 Harmonic unscaled vibrational frequencies (in cm^{-1}) for S_1 of $C_{28}N_2$ . . . 27
S167 Harmonic unscaled vibrational frequencies (in cm^{-1}) for S_2 of $C_{28}N_2$ . . . 27
S168 Harmonic unscaled vibrational frequencies (in cm^{-1}) for S_0 of $C_{28}N_2$ . . . 27
S169 Harmonic unscaled vibrational frequencies (in cm^{-1}) for S_1 of $C_{30}N_2$ . . . 28
S170 Harmonic unscaled vibrational frequencies (in cm^{-1}) for S_2 of $C_{30}N_2$ . . . 28
S171 Harmonic unscaled vibrational frequencies (in cm^{-1}) for S_0 of $C_{30}N_2$ . . . 28

*e-mail: mgronowski@ichf.edu.pl

References

[1] G. Herzberg and K. K. Innes, "Ultraviolet absorption spectra of hcn and dcn: I," *Canadian Journal of Physics*, vol. 35, no. 8, pp. 842–879, 1957.

[2] A. Chutjian, H. Tanaka, B. G. Wicke, and S. K. Srivastava, "Electron-impact excitation of the low-lying electronic states of hcn," *The Journal of Chemical Physics*, vol. 67, no. 11, pp. 4835–4839, 1977.

[3] V. Job and G. King, "The electronic spectrum of cyanoacetylene: Part i. analysis of the 2600 a system," *Journal of Molecular Spectroscopy*, vol. 19, no. 1, pp. 155–177, 1966.

[4] V. Job and G. King, "The electronic spectrum of cyanoacetylene: Part ii. analysis of the 2300 a system," *Journal of Molecular Spectroscopy*, vol. 19, no. 1, pp. 178–184, 1966.

[5] M. Turowski, C. Crépin, M. Gronowski, J.-C. Guillemin, A. Coupeaud, I. Couturier-Tamburelli, N. Piétri, and R. Kołos, "Electronic absorption and phosphorescence of cyanodiacetylene," *The Journal of Chemical Physics*, vol. 133, no. 7, p. 074310, 2010.

[6] U. Szczepaniak, R. Kołos, M. Gronowski, J.-C. Guillemin, and C. Crépin, "Low temperature synthesis and phosphorescence of methylcyanotriacetylene," *The Journal of Physical Chemistry A*, vol. 122, no. 1, pp. 89–99, 2018.

[7] U. Szczepaniak, R. Kołos, M. Gronowski, M. Chevalier, J.-C. Guillemin, M. Turowski, T. Custer, and C. Crépin, "Cryogenic photochemical synthesis and electronic spectroscopy of cyanotetracetylene," *The Journal of Physical Chemistry A*, vol. 121, no. 39, pp. 7374–7384, 2017.

[8] G. Fish, G. Cartwright, A. Walsh, and P. Warsop, "Rotational structure in the transition of cyanogen at 2200 a," *Journal of Molecular Spectroscopy*, vol. 41, no. 1, pp. 20–32, 1972.

[9] J. H. Callomon and A. B. Davey, "Rotational analysis of the 3000   absorption system of cyanogen," *Proceedings of the Physical Society*, vol. 82, pp. 335–336–1, aug 1963.

[10] J.-W. Chang and Y.-P. Lee, "The c2n2 chemiluminescence in matrices," *Journal of Molecular Structure*, vol. 157, no. 1, pp. 155–165, 1987.

[11] G. Fischer, G. D. Johnson, D. A. Ramsay, and I. G. Ross, "Electronic spectrum of dicyanoacetylene. 2. interpretation of the 2800 Å transition," *The Journal of Physical Chemistry A*, vol. 107, no. 49, pp. 10637–10641, 2003.

[12] M. Turowski, *Niskotemperaturowe badania fotochemii i spektroskopii cyjanoacetylenów o znaczeniu astrofizycznym.* PhD thesis, Institute of Physical Chemistry Polish Academy of Sciences, Kasprzaka 44/52; 01-224 Warsaw, Poland, August 2012.

[13] F. Miller and R. Hannan, "The ultraviolet absorption spectrum of dicyanoacetylene," *Spectrochimica Acta*, vol. 12, no. 4, pp. 321–331, 1958.

[14] M. Turowski, C. Crépin, I. Couturier-Tamburelli, N. Piétri, and R. Kołos, "Low-temperature phosphorescence of dicyanoacetylene in rare gas solids," *Low Temperature Physics*, vol. 38, no. 8, pp. 723–726, 2012.

[15] H. D. McSwiney and J. A. Merritt, "3100-Å electronic absorption of dicyanodiacetylene," *The Journal of Chemical Physics*, vol. 52, no. 10, pp. 5184–5186, 1970.

[16] C. Crépin, M. Turowski, J. Ceponkus, S. Douin, S. Boyé-Péronne, M. Gronowski, and R. Kołos, "Uv-induced growth of cyanopolyyne chains in cryogenic solids," *Phys. Chem. Chem. Phys.*, vol. 13, pp. 16780–16785, 2011.

[17] M. Turowski, C. Crépin, S. Douin, and R. Kołos, "Formation and spectroscopy of dicyanotriacetylene (nc8n) in solid kr," *The Journal of Physical Chemistry A*, vol. 119, no. 11, pp. 2701–2708, 2015.

[18] U. Szczepaniak, R. Kołos, M. Gronowski, M. Chevalier, J.-C. Guillemin, and C. Crépin, "Synthesis and electronic phosphorescence of dicyanooctatetrayne (nc10n) in cryogenic matrixes," *The Journal of Physical Chemistry A*, vol. 122, no. 25, pp. 5580–5588, 2018.

[19] N. I. of Standards and T. (U.S.), *NIST Chemistry Webbook: NIST Standard Reference Database Number 69.* NIST, 2022.

[20] A. Dargelos, P. Karamanis, and C. Pouchan, "Ab-initio calculations of the ir spectra of dicyanodiacetylene (c6n2) beyond the harmonic approximation," *Chemical Physics Letters*, vol. 723, pp. 155–159, 2019.

[21] F. A. Miller and D. H. Lemmon, "The infrared and raman spectra of dicyanodiacetylene," *Spectrochimica Acta Part A: Molecular Spectroscopy*, vol. 23, no. 5, pp. 1415–1423, 1967.

Figure S1: TD-DFT results for HC_7N. Energies of the vertical electronic excitations from S_0 to S_1-S_5 states and of the vibrationless (0-0) transitions from S_0 to S_1,S_2. Ordinate axis values represent the oscillator strength for vertical transitions. Triple-zeta basis sets were employed in all computations. The experimentally derived wavelength of the vibrationless S_2-S_0 transition is marked with a gray line.

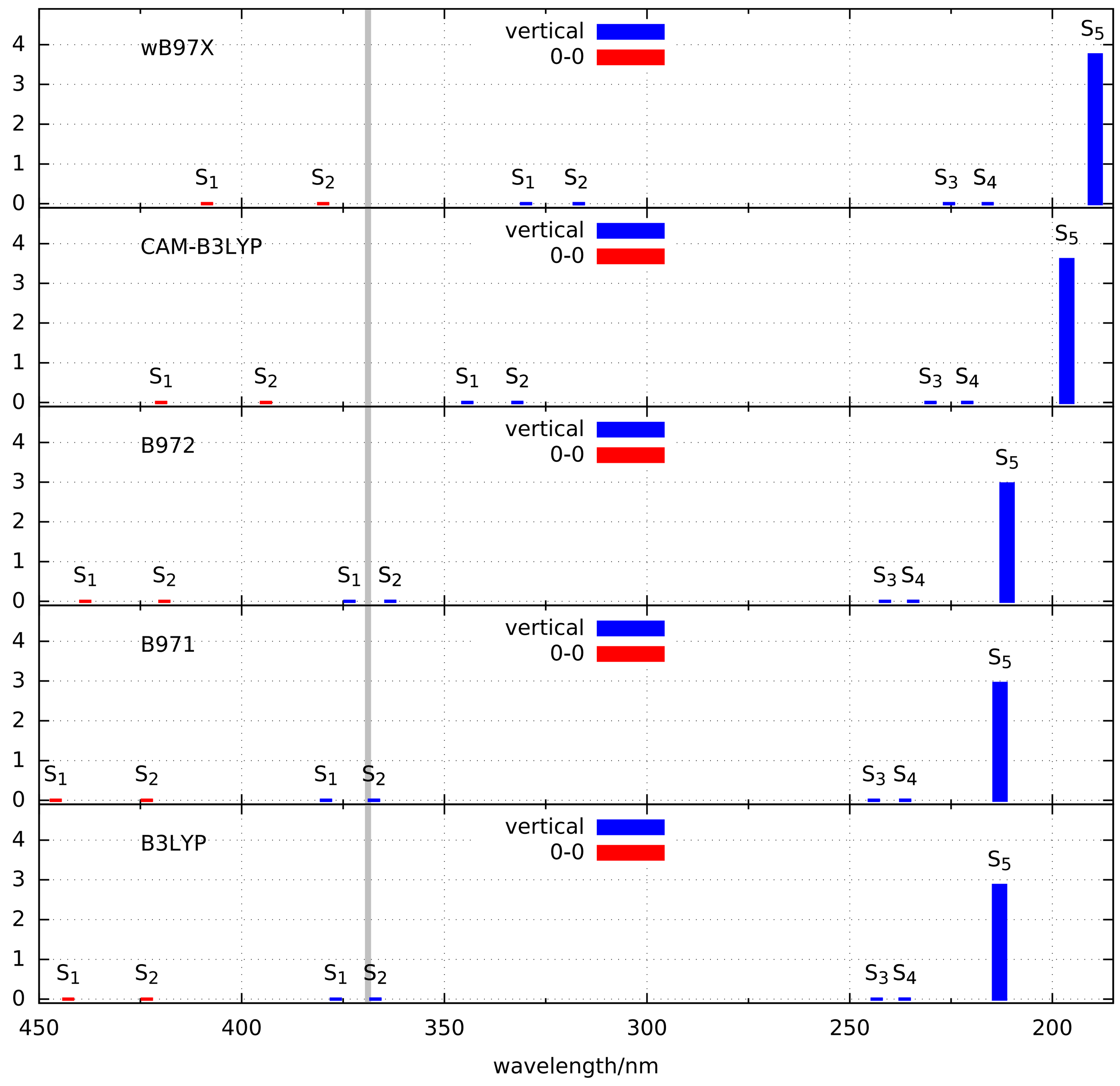

Figure S2: HOMO and LUMO molecular orbitals of $C_{2n+2}N_2$ molecules (B972/aug-cc-pVTZ).

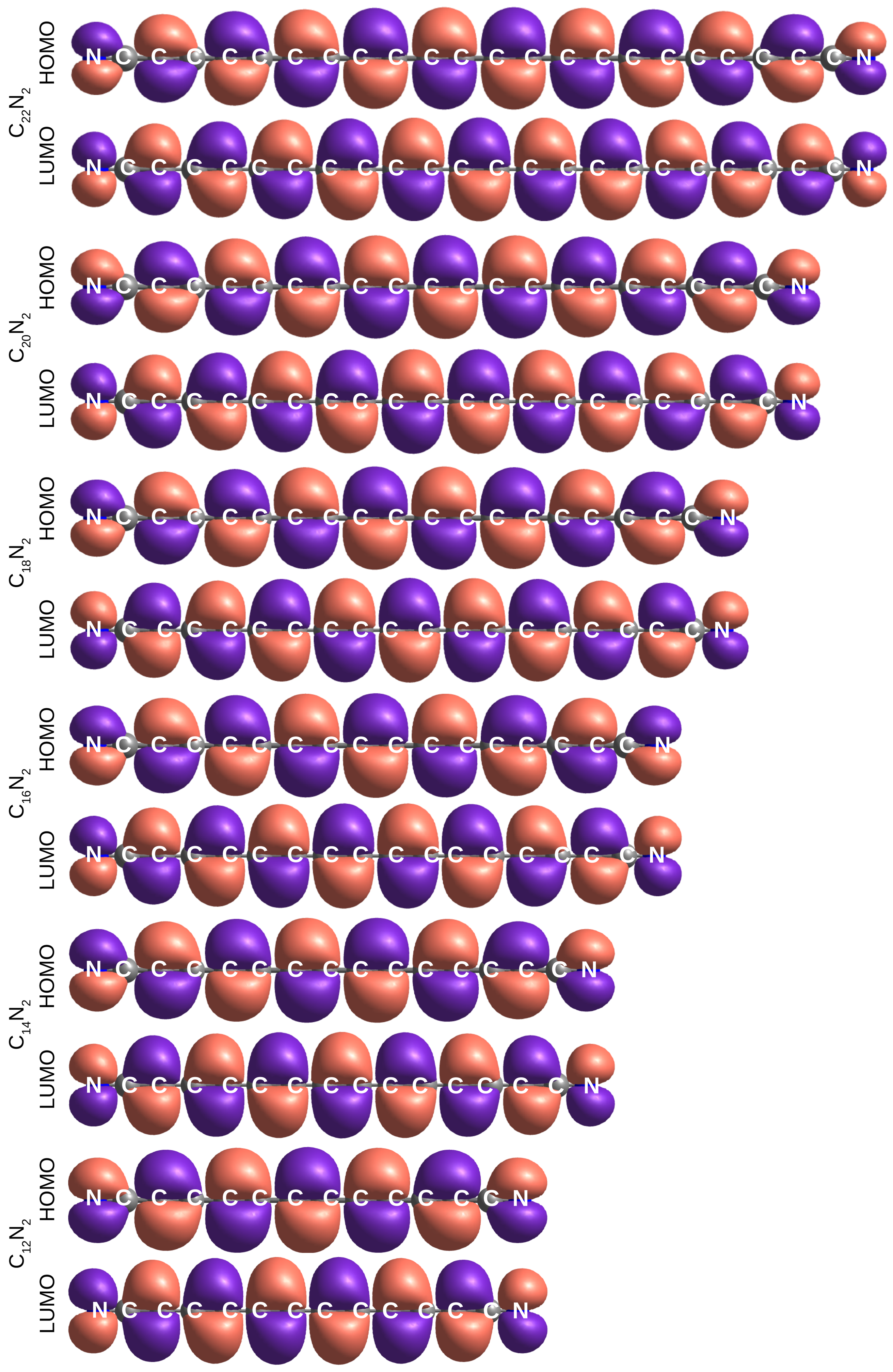

Figure S3: HOMO and LUMO molecular orbitals of $HC_{2n+1}N$ molecules (B972/aug-cc-pVTZ).

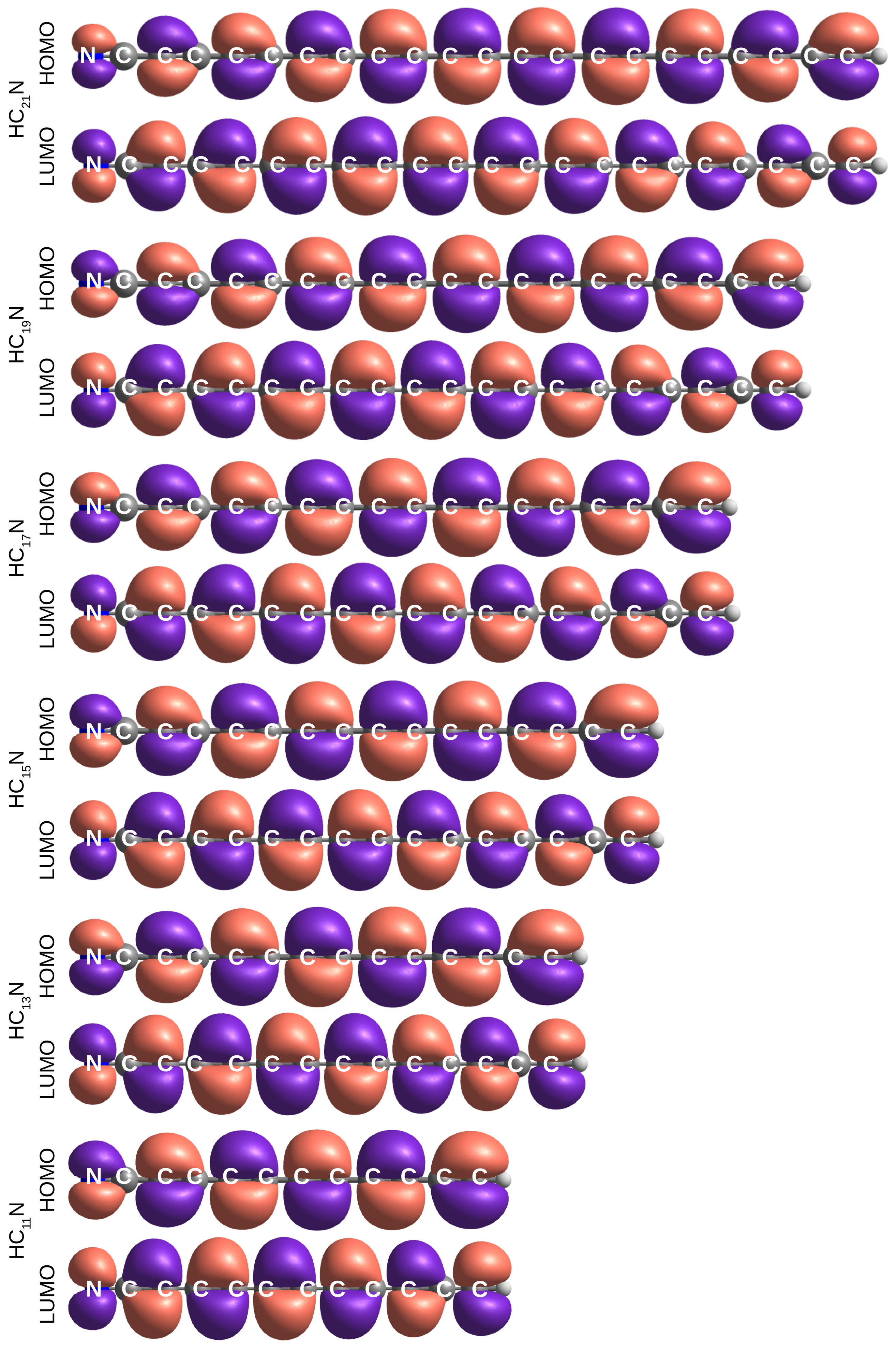

Table S1: Mean percent errors (with respect to the measured gas-phase values) of the singlet-singlet excitation energy calculations performed with various functional / basis set combinations. $\bar{\Delta}$, Δ_{st}, $\bar{\Delta}_{abs}$ are defined by Eqs. 2-5.

	aug-cc-pVDZ			aug-cc-pVTZ			def2SVPD			def2TZVPD			def2QZVPD		
	$\bar{\Delta}$	Δ_{st}	$\bar{\Delta}_{abs}$	$\bar{\Delta}$	Δ_{st}	$\bar{\Delta}_{abs}$	$\bar{\Delta}$	Δ_{st}	$\bar{\Delta}_{abs}$	$\bar{\Delta}$	Δ_{st}	$\bar{\Delta}_{abs}$	$\bar{\Delta}$	Δ_{st}	$\bar{\Delta}_{abs}$
tHCTHhyb	-16.1	5.04	16.1	-14.28	5.27	14.28	-14.87	5.45	14.87	-14.28	5.32	14.28	-14.13	5.28	14.13
VSXC	-14.53	6.52	14.53	-13.22	7.11	13.22	-13.62	6.84	13.62	-13.41	7.15	13.41	-13.04	6.91	13.04
BMK	-14.93	4.43	14.93	-12.16	3.99	12.16	-13.71	4.58	13.71	-12.53	4.14	12.53	-11.88	3.99	11.88
B1B95	-14.64	4.06	14.64	-12.73	4.37	12.73	-13.19	4.63	13.19	-12.96	4.26	12.96	-12.9	4.24	12.9
B3P86	-14.79	4.79	14.79	-12.98	5.01	12.98	-13.63	5.19	13.63	-13	5.07	13	-12.91	5.04	12.91
B98	-15.98	4.56	15.98	-14.03	4.72	14.03	-14.76	4.94	14.76	-14.08	4.78	14.08	-13.92	4.75	13.92
B971	-16.01	4.59	16.01	-14.13	4.74	14.13	-14.81	4.96	14.81	-14.17	4.8	14.17	-14.03	4.76	14.03
B972	-14.61	4.63	14.61	-12.98	4.87	12.98	-13.43	5.05	13.43	-12.97	4.93	12.97	-12.88	4.9	12.88
wB97X	-11.7	2.64	11.7	-9.91	2.71	9.91	-10.52	2.97	10.52	-9.97	2.79	9.97	-9.83	2.72	9.83
wB97XD	-13.19	3.37	13.19	-11.29	3.5	11.29	-11.97	3.76	11.97	-11.37	3.58	11.37	-11.16	3.5	11.16
CAM-B3LYP	-13.14	3.32	13.14	-11.17	3.45	11.17	-11.9	3.64	11.9	-11.19	3.51	11.19	-11.04	3.47	11.04
M062X	-16.13	8.23	16.13	-14.36	7.41	14.36	-14.99	8.06	14.99	-14.02	7.67	14.02	-14.06	7.49	14.06
SVWN	-15.57	6.43	15.57	-13.55	6.53	13.55	-14.4	6.8	14.4	-14.16	7.02	14.16	-	-	-
B3PW91	-14.83	4.7	14.83	-13.07	4.92	13.07	-13.67	5.1	13.67	-13.08	4.98	13.08	-12.98	4.95	12.98
B3LYP	-15.78	4.61	15.78	-13.43	4.88	13.43	-14.59	4.99	14.59	-13.9	4.88	13.9	-13.81	4.85	13.81

Table S2: Mean percent errors (with respect to the measured Kr-matrix values) of the singlet-singlet excitation energy calculations performed with various functional / basis set combinations. $\bar{\Delta}$, Δ_{st}, $\bar{\Delta}_{abs}$ are defined by Eqs. 2-5.

	aug-cc-pVDZ			aug-cc-pVTZ			def2SVPD			def2TZVPD			def2QZVPD		
	$\bar{\Delta}$	Δ_{st}	$\bar{\Delta}_{abs}$	$\bar{\Delta}$	Δ_{st}	$\bar{\Delta}_{abs}$	$\bar{\Delta}$	Δ_{st}	$\bar{\Delta}_{abs}$	$\bar{\Delta}$	Δ_{st}	$\bar{\Delta}_{abs}$	$\bar{\Delta}$	Δ_{st}	$\bar{\Delta}_{abs}$
tHCTHhyb	-17.00	1.32	17.00	-15.37	1.50	15.37	-16.27	1.48	16.27	-15.48	1.50	15.48	-14.88	1.40	14.88
VSXC	-16.77	3.03	16.77	-16.39	4.04	16.39	-16.51	3.57	16.51	-16.50	4.11	16.50	-13.99	3.57	13.99
BMK	-12.58	5.17	12.58	-10.56	4.90	10.56	-12.02	4.82	12.02	-10.47	4.61	10.47	-11.87	3.90	11.87
B1B95	-13.97	2.15	13.97	-	-	-	-13.16	2.19	13.16	-12.62	1.86	12.62	-12.75	2.00	12.75
B3P86	-15.27	1.12	15.27	-13.67	1.15	13.67	-14.63	1.11	14.63	-13.80	1.12	13.80	-13.49	1.22	13.49
B98	-16.22	1.16	16.22	-14.39	1.21	14.39	-15.47	1.16	15.47	-14.55	1.18	14.55	-14.20	1.34	14.20
B971	-16.28	1.13	16.28	-14.54	1.16	14.54	-15.57	1.13	15.57	-14.87	1.35	14.87	-14.34	1.28	14.34
B972	-14.87	1.20	14.87	-13.50	1.27	13.50	-14.25	1.22	14.25	-13.58	1.26	13.58	-13.30	1.34	13.30
wB97X	-8.52	3.84	8.52	-6.92	3.76	6.92	-7.88	3.65	7.88	-7.27	3.37	7.27	-7.91	2.96	7.91
wB97XD	-11.31	2.72	11.31	-9.56	2.65	9.56	-10.62	2.51	10.62	-9.77	2.56	9.77	-10.12	2.30	10.12
CAM-B3LYP	-11.36	2.54	11.36	-9.49	2.48	9.49	-10.48	2.45	10.48	-9.63	2.40	9.63	-10.01	2.19	10.01
M062X	-10.83	11.86	10.87	-9.45	10.88	9.89	-10.28	11.50	10.38	-9.07	11.10	9.77	-11.17	10.84	11.47
SVWN	-18.33	1.60	18.33	-17.05	2.19	17.05	-17.62	1.41	17.62	-17.65	2.37	17.65	-15.96	0.72	15.96
B3PW91	-15.22	1.12	15.22	-13.68	1.14	13.68	-14.57	1.10	14.57	-13.79	1.11	13.79	-13.90	0.93	13.90
B3LYP	-16.12	1.20	16.12	-14.02	1.05	14.02	-15.37	1.24	15.37	-14.52	1.27	14.52	-14.17	1.39	14.17

Table S3: Mean percent errors (with respect to the measured Ar-matrix values) of the singlet-triplet excitation energy calculations performed with various functional / basis set combinations. $\bar{\Delta}$, Δ_{st}, $\bar{\Delta}_{abs}$ are defined by Eqs. 2-5.

	aug-cc-pVDZ			aug-cc-pVTZ			def2SVPD			def2TZVPD		
	$\bar{\Delta}$	Δ_{st}	$\bar{\Delta}_{abs}$	$\bar{\Delta}$	Δ_{st}	$\bar{\Delta}_{abs}$	$\bar{\Delta}$	Δ_{st}	$\bar{\Delta}_{abs}$	$\bar{\Delta}$	Δ_{st}	$\bar{\Delta}_{abs}$
tHCTHhyb	-7.77	7.14	7.93	-2.76	3.75	3.54	-4.31	7.31	5.18	-4.52	7.71	5.88
VSXC	-12.81	7.50	12.81	-	-	-	-13.44	7.85	13.44	-14.51	6.73	14.51
BMK	4.41	3.60	4.41	10.04	4.2	10.04	8.31	3.45	8.31	10.42	4.03	10.42
B1B95	-0.37	4.42	3.53	1.12	5.14	4.19	5.42	2.73	5.42	1.96	3.19	2.91
B3P86	-6.02	6.19	6.81	-5.55	5.37	5.87	-3.63	4.15	4.15	-5.66	7.33	7.57
B98	-1.47	5.61	4.47	-1.52	3.63	3.11	-3.51	7.86	6.64	-5.10	5.20	6.10
B971	0.49	3.34	2.78	0.40	3.65	2.90	-0.72	6.22	4.04	0.09	6.1	4.28
B972	-2.13	6.07	4.78	-3.82	3.21	3.82	-5.26	5.38	6.12	-1.80	6.34	4.68
wB97X	6.96	4.89	6.96	13.48	5.49	13.48	6.79	4.97	6.79	15.5	2.14	15.5
wB97XD	6.41	5.25	6.55	8.72	5.08	8.72	6.80	5.34	6.8	6.45	4.85	6.45
CAM-B3LYP	5.96	5.78	6.87	4.62	4.92	4.62	4.83	5.33	5.17	6.82	5.2	6.82
M062X	16.3	5.41	16.3	16.49	4.71	16.49	16.73	4.65	16.73	18.14	4.87	18.14
SVWN	-4.84	3.85	5.39	-5.41	6.45	6.79	-5.52	5.13	6.65	-5.56	6.59	6.99
B3PW91	-2.84	4.45	4.11	-4.77	7.47	7.13	-6.14	7.75	7.68	-4.42	3.56	4.42
B3LYP	-3.72	6.4	5.34	0.05	3.84	3.04	-3.07	6.06	4.54	-3.6	6.86	6.27

Table S4: Energy separation (eV) between the ground vibrational states of S_0 and S_1, calculated for HC_1N. Experimental value(s): 6.48 eV (gas) [1] ..

metoda	aug-cc-pVDZ	aug-cc-pVTZ	def2SVPD	def2TZVPD	def2QZVPD
tHCTHhyb	5.87	6.01	5.98	6.01	6.02
VSXC	6.14	6.26	6.23	6.27	6.26
BMK	5.63	5.81	5.75	5.79	5.82
B1B95	5.82	5.95	5.93	5.96	5.96
B3P86	5.92	6.06	6.03	6.06	6.07
B98	5.79	5.94	5.90	5.94	5.95
B971	5.80	5.94	5.91	5.94	5.95
B972	5.91	6.04	6.02	6.04	6.05
wB97X	5.83	5.97	5.95	5.97	5.98
wB97XD	5.84	5.98	5.95	5.98	5.99
CAM-B3LYP	5.82	5.97	5.93	5.97	5.98
M062X	5.30	5.46	5.41	5.47	5.47
SVWN	6.16	6.30	6.28	6.30	6.31
B3PW91	5.91	6.04	6.02	6.05	6.06
B3LYP	5.82	5.96	5.93	5.97	5.97

Table S5: Energy separation (eV) between the ground vibrational states of S_0 and S_2, calculated for HC_1N. Experimental value(s): 7.1 eV (gas) [2] ..

metoda	aug-cc-pVDZ	aug-cc-pVTZ	def2SVPD	def2TZVPD	def2QZVPD
tHCTHhyb	6.57	6.69	6.68	6.69	6.70
VSXC	6.66	6.75	6.73	6.76	6.76
BMK	6.65	6.78	6.77	6.78	6.79
B1B95	6.65	6.75	6.76	6.76	6.76
B3P86	6.66	6.78	6.77	6.79	6.79
B98	6.58	6.70	6.69	6.71	6.71
B971	6.57	6.69	6.68	6.70	6.70
B972	6.65	6.76	6.76	6.77	6.77
wB97X	6.72	6.84	6.83	6.84	6.84
wB97XD	6.69	6.81	6.80	6.82	6.82
CAM-B3LYP	6.69	6.81	6.80	6.82	6.82
M062X	6.55	6.65	6.68	6.66	6.67
SVWN	6.60	6.70	6.70	6.71	6.71
B3PW91	6.65	6.76	6.76	6.77	6.77
B3LYP	6.57	6.70	6.68	6.71	6.71

Table S6: Energy separation (eV) between the ground vibrational states of S_0 and T_1, calculated for HC_1N. Experimental value(s): 4.9 eV (gas) [2] ..

metoda	aug-cc-pVDZ	aug-cc-pVTZ	def2SVPD	def2TZVPD	def2QZVPD
tHCTHhyb	4.46	4.56	4.53	4.57	4.57
VSXC	4.50	4.57	4.53	4.58	4.57
BMK	4.65	4.77	4.71	4.76	4.78
B1B95	4.59	4.69	4.66	4.69	4.70
B3P86	4.46	4.56	4.52	4.56	4.57
B98	4.47	4.57	4.54	4.58	4.58
B971	4.50	4.60	4.57	4.60	4.61
B972	4.54	4.64	4.62	4.64	4.65
wB97X	4.53	4.63	4.59	4.63	4.64
wB97XD	4.52	4.63	4.58	4.63	4.64
CAM-B3LYP	4.48	4.60	4.55	4.60	4.60
M062X	4.71	4.78	4.77	4.80	4.79
SVWN	4.92	5.04	5.01	5.04	5.05
B3PW91	4.39	4.50	4.46	4.50	4.50
B3LYP	4.45	4.56	4.52	4.56	4.57

Table S7: Energy separation (eV) between the ground vibrational states of S_0 and S_1, calculated for HC_3N. Experimental value(s): 4.77 eV (gas) [3] ..

metoda	aug-cc-pVDZ	aug-cc-pVTZ	def2SVPD	def2TZVPD	def2QZVPD
tHCTHhyb	4.01	4.16	4.12	4.16	4.16
VSXC	4.15	4.25	4.23	4.27	n/a
BMK	3.92	4.12	4.03	4.09	4.15
B1B95	4.08	4.21	4.17	4.21	4.21
B3P86	4.11	4.24	4.20	4.24	4.25
B98	4.01	4.15	4.11	4.15	4.16
B971	4.01	4.15	4.11	4.14	4.16
B972	4.10	4.23	4.20	4.23	4.23
wB97X	4.16	4.30	4.26	4.30	4.30
wB97XD	4.12	4.26	4.22	4.26	4.27
CAM-B3LYP	4.14	4.28	4.23	4.28	4.29
M062X	3.78	3.94	3.87	3.94	3.94
SVWN	4.16	4.29	4.25	4.29	4.31
B3PW91	4.10	4.23	4.20	4.23	4.24
B3LYP	4.05	4.19	4.15	4.19	4.19

Table S8: Energy separation (eV) between the ground vibrational states of S_0 and S_2, calculated for HC_3N. Experimental value(s): 5.484 eV (gas) [4] ..

metoda	aug-cc-pVDZ	aug-cc-pVTZ	def2SVPD	def2TZVPD	def2QZVPD
tHCTHhyb	4.63	4.72	4.70	4.73	4.73
VSXC	4.68	4.80	4.72	4.76	4.75
BMK	4.88	5.03	4.95	5.01	5.05
B1B95	4.78	4.87	4.85	4.87	4.87
B3P86	4.71	4.81	4.78	4.81	4.81
B98	4.63	4.75	4.71	4.74	4.74
B971	4.64	4.75	4.71	4.75	4.74
B972	4.70	4.80	4.77	4.80	4.80
wB97X	4.85	4.94	4.91	4.94	4.94
wB97XD	4.78	4.88	4.85	4.88	4.89
CAM-B3LYP	4.78	4.88	4.84	4.88	4.88
M062X	5.09	5.14	5.14	5.18	5.16
SVWN	4.73	4.86	4.80	4.79	n/a
B3PW91	4.71	4.81	4.78	4.81	4.81
B3LYP	4.63	4.74	4.71	4.74	4.74

Table S9: Energy separation (eV) between the ground vibrational states of S_0 and T_1, calculated for HC_3N. .

metoda	aug-cc-pVDZ	aug-cc-pVTZ	def2SVPD	def2TZVPD	def2QZVPD
tHCTHhyb	3.22	3.33	3.27	3.32	3.33
VSXC	3.24	3.31	3.26	3.32	3.30
BMK	3.37	3.52	3.42	3.49	3.54
B1B95	3.39	3.49	3.45	3.49	3.49
B3P86	3.23	3.34	3.28	3.33	3.34
B98	3.24	3.36	3.30	3.35	3.36
B971	3.27	3.38	3.33	3.38	3.39
B972	3.32	3.42	3.38	3.42	3.42
wB97X	3.34	3.45	3.39	3.45	3.45
wB97XD	3.31	3.43	3.36	3.42	3.43
CAM-B3LYP	3.30	3.43	3.36	3.42	3.43
M062X	3.50	3.60	3.55	3.61	3.60
SVWN	3.57	3.68	3.62	3.68	3.69
B3PW91	3.18	3.29	3.23	3.28	3.29
B3LYP	3.25	3.37	3.31	3.36	3.37

Table S10: Energy separation (eV) between the ground vibrational states of S_0 and S_1, calculated for HC_5N. Experimental value(s): 4.041 eV (gas) [5] , 3.983 eV (Ar) [5] , 3.968 eV (Kr) [5] ..

metoda	aug-cc-pVDZ	aug-cc-pVTZ	def2SVPD	def2TZVPD	def2QZVPD
tHCTHhyb	3.34	3.42	3.37	3.41	3.42
VSXC	3.44	3.50	3.47	3.51	3.56
BMK	3.22	3.35	3.24	3.33	3.37
B1B95	3.34	3.40	3.36	3.40	3.40
B3P86	3.38	3.45	3.40	3.45	3.45
B98	3.35	3.42	3.37	3.42	3.42
B971	3.34	3.42	3.37	3.41	3.41
B972	3.41	3.47	3.43	3.47	3.47
wB97X	3.54	3.61	3.56	3.60	3.61
wB97XD	3.47	3.54	3.49	3.53	3.54
CAM-B3LYP	3.47	3.55	3.49	3.54	3.55
M062X	2.95	3.06	2.98	3.06	3.07
SVWN	3.28	3.35	3.30	3.35	3.36
B3PW91	3.38	3.45	3.40	3.44	3.44
B3LYP	3.36	3.44	3.39	3.43	3.43

Table S11: Energy separation (eV) between the ground vibrational states of S_0 and S_2, calculated for HC_5N. Experimental value(s): 4.389 eV (gas) [5] , 4.316 eV (Ar) [5] , 4.298 eV (Kr) [5] ..

metoda	aug-cc-pVDZ	aug-cc-pVTZ	def2SVPD	def2TZVPD	def2QZVPD
tHCTHhyb	3.51	3.57	3.53	3.56	3.57
VSXC	3.48	3.49	3.47	3.49	3.54
BMK	3.78	3.89	3.79	3.87	3.90
B1B95	3.67	n/a	n/a	3.72	3.72
B3P86	3.58	3.64	3.60	3.64	3.64
B98	3.54	3.60	3.56	3.60	3.60
B971	3.54	3.60	3.56	3.60	3.60
B972	3.59	3.64	3.61	3.64	3.64
wB97X	3.81	3.87	3.83	3.86	3.87
wB97XD	3.72	3.78	3.73	3.77	3.79
CAM-B3LYP	3.71	3.78	3.74	3.77	3.78
M062X	3.99	4.02	4.01	4.05	4.04
SVWN	3.49	3.62	3.53	3.49	3.57
B3PW91	3.59	3.64	3.61	3.64	3.65
B3LYP	3.54	n/a	3.56	3.60	3.60

Table S12: Energy separation (eV) between the ground vibrational states of S_0 and T_1, calculated for HC_5N. Experimental value(s): 2.919 eV (Ar) [5] , 2.911 eV (Kr) [5] ..

metoda	aug-cc-pVDZ	aug-cc-pVTZ	def2SVPD	def2TZVPD	def2QZVPD
tHCTHhyb	2.90	2.94	n/a	2.94	2.95
VSXC	2.87	n/a	2.82	n/a	n/a
BMK	3.17	3.27	3.16	3.24	3.29
B1B95	3.11	3.14	3.09	n/a	3.15
B3P86	2.99	n/a	2.96	n/a	3.02
B98	2.97	3.01	n/a	3.01	3.02
B971	2.98	3.02	2.96	3.01	3.03
B972	3.02	n/a	2.99	n/a	3.04
wB97X	3.23	3.27	n/a	3.26	3.27
wB97XD	3.14	3.19	n/a	3.18	3.19
CAM-B3LYP	3.15	3.20	3.13	3.20	3.21
M062X	3.39	3.41	3.36	3.43	3.43
SVWN	n/a	2.87	2.80	2.86	2.87
B3PW91	2.97	3.00	2.94	n/a	3.00
B3LYP	2.98	3.02	2.96	3.01	3.02

Table S13: Energy separation (eV) between the ground vibrational states of S_0 and S_1, calculated for HC_7N. .

metoda	aug-cc-pVDZ	aug-cc-pVTZ	def2SVPD	def2TZVPD	def2QZVPD
tHCTHhyb	2.71	2.77	2.73	2.76	2.77
VSXC	2.76	2.77	2.80	2.82	n/a
BMK	2.64	2.76	2.65	2.72	n/a
B1B95	2.73	2.78	2.74	2.77	2.78
B3P86	2.75	2.81	2.76	2.79	2.81
B98	2.73	2.79	2.74	2.78	2.79
B971	2.72	2.78	2.74	2.77	2.78
B972	2.78	2.83	2.79	2.82	2.83
wB97X	2.97	3.03	2.99	3.03	3.04
wB97XD	2.88	2.94	2.90	2.93	2.94
CAM-B3LYP	2.89	2.95	2.91	2.94	2.96
M062X	2.39	2.49	2.41	2.48	2.49
SVWN	2.61	2.67	2.62	2.65	2.67
B3PW91	2.75	2.80	2.76	2.79	2.81
B3LYP	2.74	2.80	2.76	2.79	2.80

Table S14: Energy separation (eV) between the ground vibrational states of S_0 and S_2, calculated for HC_7N. Experimental value(s): 3.36 eV (Kr) [6] ..

metoda	aug-cc-pVDZ	aug-cc-pVTZ	def2SVPD	def2TZVPD	def2QZVPD
tHCTHhyb	2.83	2.88	2.85	2.87	2.89
VSXC	2.79	2.77	2.80	2.82	n/a
BMK	3.13	3.22	3.13	3.19	n/a
B1B95	3.00	3.04	3.02	3.04	3.04
B3P86	2.91	2.96	2.92	2.95	2.96
B98	2.88	2.93	2.89	2.92	2.94
B971	2.88	2.93	2.89	2.92	2.93
B972	2.92	2.96	2.93	2.96	2.96
wB97X	3.22	3.26	3.23	3.25	3.27
wB97XD	3.10	3.15	3.11	3.14	3.16
CAM-B3LYP	3.09	3.15	3.11	3.14	3.15
M062X	3.35	3.37	3.36	3.40	3.39
SVWN	2.78	2.74	2.78	2.79	2.82
B3PW91	2.92	2.96	2.93	2.95	n/a
B3LYP	2.88	2.93	2.89	2.92	2.93

Table S15: Energy separation (eV) between the ground vibrational states of S_0 and T_1, calculated for HC_7N. Experimental value(s): 2.4504 eV (Ar) [6] , 2.4412 eV (Kr) [6] ..

metoda	aug-cc-pVDZ	aug-cc-pVTZ	def2SVPD	def2TZVPD	def2QZVPD
tHCTHhyb	2.46	2.49	2.46	2.48	2.49
VSXC	2.10	2.12	2.08	2.12	n/a
BMK	2.50	2.60	2.75	2.58	n/a
B1B95	2.38	2.39	2.64	2.42	2.66
B3P86	2.22	2.27	2.23	2.27	n/a
B98	2.53	2.31	2.53	2.30	2.31
B971	2.54	2.57	2.54	2.56	2.57
B972	2.55	2.33	2.29	2.32	2.33
wB97X	2.81	2.86	2.55	2.85	n/a
wB97XD	2.72	2.76	2.72	2.50	n/a
CAM-B3LYP	2.70	2.49	2.72	2.48	2.49
M062X	2.95	2.98	2.95	2.80	2.99
SVWN	2.26	2.32	2.27	2.32	2.32
B3PW91	2.50	2.25	2.21	2.24	2.53
B3LYP	2.52	2.56	2.53	2.29	2.56

Table S16: Energy separation (eV) between the ground vibrational states of S_0 and S_1, calculated for HC_9N. .

metoda	aug-cc-pVDZ	aug-cc-pVTZ	def2SVPD	def2TZVPD	def2QZVPD
tHCTHhyb	2.29	2.34	2.31	2.33	n/a
VSXC	2.32	2.29	2.31	2.29	n/a
BMK	2.25	2.38	2.26	2.30	n/a
B1B95	2.33	n/a	2.34	2.37	n/a
B3P86	2.34	2.39	2.35	2.38	n/a
B98	2.32	2.38	2.34	2.37	n/a
B971	2.32	2.37	2.33	2.33	n/a
B972	2.36	2.41	2.38	2.40	n/a
wB97X	2.62	2.69	2.64	2.65	n/a
wB97XD	2.52	2.57	2.53	2.56	n/a
CAM-B3LYP	2.52	2.58	2.54	2.57	n/a
M062X	1.98	2.10	1.99	2.07	n/a
SVWN	2.17	2.22	2.18	2.21	n/a
B3PW91	2.34	2.39	2.35	2.38	n/a
B3LYP	2.33	2.39	2.35	2.38	n/a

Table S17: Energy separation (eV) between the ground vibrational states of S_0 and S_2, calculated for HC_9N. Experimental value(s): 2.938 eV (Ar) [7] , 2.94 eV (Kr) [7] ..

metoda	aug-cc-pVDZ	aug-cc-pVTZ	def2SVPD	def2TZVPD	def2QZVPD
tHCTHhyb	2.39	2.44	2.41	2.43	n/a
VSXC	2.34	2.29	2.31	2.29	n/a
BMK	2.70	n/a	2.70	2.73	n/a
B1B95	2.57	n/a	2.58	2.60	n/a
B3P86	2.47	2.51	2.48	2.51	n/a
B98	2.45	2.50	2.46	2.49	n/a
B971	2.44	2.49	2.46	2.45	n/a
B972	2.48	2.52	2.49	2.52	n/a
wB97X	2.85	2.90	2.86	2.86	n/a
wB97XD	2.71	2.76	2.72	2.75	n/a
CAM-B3LYP	2.70	2.75	2.72	2.74	n/a
M062X	2.94	2.97	2.95	2.96	n/a
SVWN	2.31	2.32	2.34	2.30	n/a
B3PW91	2.47	2.52	2.49	2.51	n/a
B3LYP	2.45	2.49	2.46	2.49	n/a

Table S18: Energy separation (eV) between the ground vibrational states of S_0 and T_1, calculated for HC_9N. Experimental value(s): 2.1547 eV (Ar) [7] , 2.1449 eV (Kr) [7] ..

metoda	aug-cc-pVDZ	aug-cc-pVTZ	def2SVPD	def2TZVPD	def2QZVPD
tHCTHhyb	1.85	2.11	2.08	1.88	n/a
VSXC	1.76	1.76	1.73	1.73	n/a
BMK	2.17	2.48	2.39	2.40	n/a
B1B95	2.04	2.29	2.27	2.29	n/a
B3P86	2.13	2.17	2.14	1.92	n/a
B98	1.93	2.19	1.93	1.96	n/a
B971	2.15	2.19	2.16	2.15	n/a
B972	2.16	1.98	1.95	2.19	n/a
wB97X	2.27	n/a	2.26	2.49	n/a
wB97XD	2.38	2.43	2.38	2.41	n/a
CAM-B3LYP	2.36	2.41	2.14	2.40	n/a
M062X	2.60	2.47	2.60	2.62	n/a
SVWN	2.08	1.93	1.89	1.93	n/a
B3PW91	2.11	1.91	1.87	2.15	n/a
B3LYP	2.14	2.19	2.15	1.95	n/a

Table S19: Energy separation (eV) between the ground vibrational states of S_0 and S_1, calculated for C_2N_2. Experimental value(s): 5.63 eV (gas) [8] ..

metoda	aug-cc-pVDZ	aug-cc-pVTZ	def2SVPD	def2TZVPD	def2QZVPD
tHCTHhyb	4.89	4.99	4.98	4.99	5.00
VSXC	5.10	5.18	5.18	5.15	5.18
BMK	4.69	4.83	4.78	4.82	4.83
B1B95	4.83	4.92	4.91	4.92	4.93
B3P86	4.91	5.01	4.99	5.01	5.02
B98	4.86	4.96	4.95	4.97	4.97
B971	4.86	4.95	4.94	4.96	4.96
B972	4.93	5.02	5.02	5.03	5.03
wB97X	5.02	5.12	5.11	5.12	5.13
wB97XD	4.95	5.05	5.04	5.05	5.06
CAM-B3LYP	4.97	5.07	5.05	5.08	5.08
M062X	4.29	4.41	4.38	4.42	4.43
SVWN	4.90	5.00	4.98	5.01	5.01
B3PW91	4.90	5.00	4.99	5.00	5.01
B3LYP	4.87	4.97	4.95	4.98	4.98

Table S20: Energy separation (eV) between the ground vibrational states of S_0 and S_2, calculated for C_2N_2. .

metoda	aug-cc-pVDZ	aug-cc-pVTZ	def2SVPD	def2TZVPD	def2QZVPD
tHCTHhyb	5.16	5.24	5.24	5.24	5.25
VSXC	5.21	5.26	5.26	5.24	5.24
BMK	5.48	5.58	5.57	5.58	5.59
B1B95	5.31	5.37	5.39	5.38	5.38
B3P86	5.24	5.32	5.32	5.33	5.33
B98	5.16	5.25	5.25	5.26	5.26
B971	5.17	5.25	5.25	5.26	5.26
B972	5.24	5.31	5.32	5.32	5.32
wB97X	5.42	5.49	5.50	5.50	5.50
wB97XD	5.33	5.41	5.42	5.42	5.42
CAM-B3LYP	5.32	5.41	5.41	5.42	5.42
M062X	5.58	5.63	5.65	5.66	5.65
SVWN	5.25	5.32	5.31	5.31	5.32
B3PW91	5.24	5.32	5.32	5.32	5.32
B3LYP	5.16	5.25	5.24	5.25	5.25

Table S21: Energy separation (eV) between the ground vibrational states of S_0 and T_1, calculated for C_2N_2. Experimental value(s): 4.13 eV (gas) [9] , 4.1219 eV (Ar) [10] ..

metoda	aug-cc-pVDZ	aug-cc-pVTZ	def2SVPD	def2TZVPD	def2QZVPD
tHCTHhyb	3.90	4.00	3.98	4.00	4.01
VSXC	3.86	3.93	3.90	3.93	3.92
BMK	4.24	4.39	4.33	4.38	4.39
B1B95	4.11	4.21	4.19	4.21	4.21
B3P86	3.95	4.06	4.03	4.06	4.06
B98	3.95	4.06	4.03	4.06	4.07
B971	3.96	4.07	4.05	4.07	4.08
B972	4.01	4.11	4.10	4.11	4.11
wB97X	4.20	4.31	4.27	n/a	4.31
wB97XD	4.11	4.23	4.19	4.23	4.24
CAM-B3LYP	4.09	4.21	4.18	n/a	4.22
M062X	4.46	4.55	4.54	4.56	4.56
SVWN	4.18	4.29	4.26	4.30	4.30
B3PW91	3.91	4.02	3.99	4.02	4.02
B3LYP	3.94	4.05	4.02	4.06	4.06

Table S22: Energy separation (eV) between the ground vibrational states of S_0 and S_1, calculated for C_4N_2. Experimental value(s): 4.3966 eV (gas) [11] , 4.288 eV (Kr) [12] ..

metoda	aug-cc-pVDZ	aug-cc-pVTZ	def2SVPD	def2TZVPD	def2QZVPD
tHCTHhyb	3.59	3.68	3.65	3.68	3.69
VSXC	3.72	3.79	3.75	3.77	3.79
BMK	3.48	3.63	3.54	3.61	3.63
B1B95	3.59	3.67	3.64	3.67	3.68
B3P86	3.63	3.72	3.68	3.72	3.72
B98	3.59	3.69	3.66	3.69	3.70
B971	3.59	3.68	3.65	3.68	3.69
B972	3.66	3.74	3.71	3.74	3.74
wB97X	3.80	3.89	3.85	3.89	3.89
wB97XD	3.71	3.81	3.77	3.81	3.81
CAM-B3LYP	3.73	3.83	3.79	3.83	3.84
M062X	3.19	3.30	3.25	3.31	3.32
SVWN	3.52	3.62	3.58	3.61	3.62
B3PW91	3.63	3.72	3.68	3.71	3.72
B3LYP	3.61	3.70	3.67	3.70	3.71

Table S23: Energy separation (eV) between the ground vibrational states of S_0 and S_2, calculated for C_4N_2. Experimental value(s): 4.688 eV (gas) [13] , 4.578 eV (Kr) [12] ..

metoda	aug-cc-pVDZ	aug-cc-pVTZ	def2SVPD	def2TZVPD	def2QZVPD
tHCTHhyb	3.78	3.85	3.83	3.85	3.86
VSXC	3.76	3.83	3.80	3.75	3.83
BMK	4.09	4.20	4.14	4.19	4.22
B1B95	3.95	4.01	3.99	4.01	4.01
B3P86	3.86	3.93	3.91	3.93	3.94
B98	3.82	3.89	3.86	3.89	3.90
B971	3.82	3.89	3.86	3.89	3.90
B972	3.87	3.93	3.91	3.93	3.94
wB97X	4.11	4.17	4.15	4.17	4.18
wB97XD	4.00	4.08	4.04	4.07	4.08
CAM-B3LYP	4.00	4.08	4.05	4.08	4.08
M062X	4.28	4.32	4.31	4.35	4.34
SVWN	3.79	3.84	3.81	3.82	n/a
B3PW91	3.87	3.93	3.91	3.93	3.94
B3LYP	3.81	3.89	3.86	3.89	3.89

Table S24: Energy separation (eV) between the ground vibrational states of S_0 and T_1, calculated for C_4N_2. Experimental value(s): 3.181 eV (Ar) [14] , 3.138 eV (Kr) [14] ..

metoda	aug-cc-pVDZ	aug-cc-pVTZ	def2SVPD	def2TZVPD	def2QZVPD
tHCTHhyb	n/a	n/a	n/a	n/a	n/a
VSXC	n/a	n/a	n/a	n/a	n/a
BMK	n/a	n/a	n/a	n/a	n/a
B1B95	n/a	n/a	n/a	n/a	n/a
B3P86	n/a	n/a	n/a	n/a	n/a
B98	n/a	n/a	n/a	n/a	n/a
B971	n/a	n/a	n/a	n/a	n/a
B972	n/a	n/a	n/a	n/a	n/a
wB97X	n/a	n/a	n/a	n/a	n/a
wB97XD	n/a	n/a	n/a	n/a	n/a
CAM-B3LYP	n/a	n/a	n/a	n/a	n/a
M062X	n/a	n/a	n/a	n/a	n/a
SVWN	n/a	n/a	n/a	n/a	n/a
B3PW91	n/a	n/a	n/a	n/a	n/a
B3LYP	n/a	n/a	n/a	n/a	n/a

Table S25: Energy separation (eV) between the ground vibrational states of S_0 and S_1, calculated for C_6N_2. .

metoda	aug-cc-pVDZ	aug-cc-pVTZ	def2SVPD	def2TZVPD	def2QZVPD
tHCTHhyb	2.88	2.94	2.90	2.93	2.94
VSXC	2.94	2.99	2.96	2.98	2.99
BMK	2.81	2.93	2.84	2.90	2.94
B1B95	2.89	2.95	2.92	2.95	2.96
B3P86	2.91	2.98	2.94	2.97	2.98
B98	2.90	2.96	2.92	2.95	2.97
B971	2.89	2.95	2.92	2.95	2.96
B972	2.94	3.00	2.97	2.99	3.00
wB97X	3.14	3.21	3.17	3.20	3.21
wB97XD	3.05	3.11	3.07	3.10	3.12
CAM-B3LYP	3.06	3.13	3.09	3.12	3.14
M062X	2.55	2.65	2.58	2.65	2.66
SVWN	2.77	2.83	2.80	2.83	2.84
B3PW91	2.92	2.97	2.94	2.97	2.98
B3LYP	2.90	2.97	2.94	2.97	2.98

Table S26: Energy separation (eV) between the ground vibrational states of S_0 and S_2, calculated for C_6N_2. Experimental value(s): 3.994 eV (gas) [15] ..

metoda	aug-cc-pVDZ	aug-cc-pVTZ	def2SVPD	def2TZVPD	def2QZVPD
tHCTHhyb	3.02	3.06	3.04	3.06	3.07
VSXC	2.95	2.94	2.98	2.96	3.02
BMK	3.33	3.42	3.35	3.40	3.44
B1B95	3.19	3.23	3.21	3.23	3.23
B3P86	3.09	3.14	3.12	3.14	3.15
B98	3.07	3.12	3.08	3.11	3.12
B971	3.06	3.11	3.08	3.11	3.12
B972	3.11	3.14	3.13	3.14	3.15
wB97X	3.40	3.45	3.42	3.44	3.45
wB97XD	3.28	3.33	3.30	3.33	3.34
CAM-B3LYP	3.28	3.33	3.31	3.33	3.34
M062X	3.54	3.56	3.56	3.60	3.58
SVWN	2.97	3.02	3.00	2.98	3.02
B3PW91	3.10	3.14	3.12	3.14	3.15
B3LYP	3.06	3.11	3.08	3.11	3.12

Table S27: Energy separation (eV) between the ground vibrational states of S_0 and T_1, calculated for C_6N_2. Experimental value(s): 2.601 eV (Ar) [16] , 2.590 eV (Kr) [16] ..

metoda	aug-cc-pVDZ	aug-cc-pVTZ	def2SVPD	def2TZVPD	def2QZVPD
tHCTHhyb	2.34	2.38	2.65	2.66	2.66
VSXC	2.24	2.27	2.24	2.27	2.27
BMK	2.67	2.77	2.69	3.02	3.05
B1B95	2.52	2.57	2.83	2.57	2.85
B3P86	2.37	2.42	2.39	2.73	2.43
B98	2.71	2.46	2.72	2.45	2.46
B971	2.71	2.46	2.73	2.74	2.47
B972	2.43	2.47	2.45	2.75	2.75
wB97X	2.69	3.03	3.01	3.02	2.74
wB97XD	2.60	2.93	2.61	2.64	2.94
CAM-B3LYP	2.89	2.64	2.60	2.63	2.64
M062X	2.90	2.94	3.14	3.17	2.95
SVWN	2.42	2.47	2.44	2.47	2.48
B3PW91	2.35	2.72	2.70	2.40	2.40
B3LYP	2.39	2.44	2.41	2.73	2.74

Table S28: Energy separation (eV) between the ground vibrational states of S_0 and S_1, calculated for C_8N_2. .

metoda	aug-cc-pVDZ	aug-cc-pVTZ	def2SVPD	def2TZVPD	def2QZVPD
tHCTHhyb	2.41	2.46	2.43	2.46	2.47
VSXC	2.44	2.47	2.44	2.47	n/a
BMK	2.36	2.49	2.39	2.46	n/a
B1B95	2.45	2.50	2.46	2.49	n/a
B3P86	2.45	2.50	2.47	2.50	n/a
B98	2.44	2.50	2.46	2.49	n/a
B971	2.43	2.49	2.45	2.48	n/a
B972	2.48	2.53	2.50	2.52	n/a
wB97X	2.74	2.80	2.76	2.79	n/a
wB97XD	2.62	2.69	2.65	2.67	n/a
CAM-B3LYP	2.63	2.70	2.66	2.69	n/a
M062X	n/a	2.21	2.13	2.21	n/a
SVWN	2.28	2.34	2.30	2.33	n/a
B3PW91	2.46	2.50	2.47	2.50	n/a
B3LYP	2.45	n/a	2.47	2.50	n/a

Table S29: Energy separation (eV) between the ground vibrational states of S_0 and S_2, calculated for C_8N_2. .

metoda	aug-cc-pVDZ	aug-cc-pVTZ	def2SVPD	def2TZVPD	def2QZVPD
tHCTHhyb	2.52	2.56	2.54	2.56	n/a
VSXC	2.44	2.49	2.44	2.44	n/a
BMK	2.83	2.94	2.86	2.91	n/a
B1B95	2.71	2.74	2.72	2.73	n/a
B3P86	2.60	2.64	2.62	2.64	n/a
B98	2.57	2.62	2.59	2.62	n/a
B971	2.57	2.62	2.59	2.61	n/a
B972	2.61	2.65	2.63	2.65	n/a
wB97X	2.98	3.02	2.99	3.01	n/a
wB97XD	2.84	2.88	2.85	2.87	n/a
CAM-B3LYP	2.82	2.88	2.85	2.87	n/a
M062X	n/a	3.10	3.09	3.13	n/a
SVWN	2.44	2.47	2.46	2.46	n/a
B3PW91	2.61	2.64	2.62	2.64	n/a
B3LYP	2.57	n/a	2.59	2.62	n/a

Table S30: Energy separation (eV) between the ground vibrational states of S_0 and T_1, calculated for C_8N_2. Experimental value(s): 2.248 eV (Kr) [17] ..

metoda	aug-cc-pVDZ	aug-cc-pVTZ	def2SVPD	def2TZVPD	def2QZVPD
tHCTHhyb	2.19	1.99	1.96	2.22	n/a
VSXC	1.85	1.87	1.84	1.86	n/a
BMK	2.28	2.61	2.53	2.58	n/a
B1B95	2.14	2.41	2.39	2.18	n/a
B3P86	2.26	2.29	2.00	2.03	n/a
B98	2.27	2.07	2.28	2.06	n/a
B971	2.03	2.31	2.28	2.07	n/a
B972	2.05	2.31	2.30	2.31	n/a
wB97X	2.36	2.41	2.61	2.63	n/a
wB97XD	2.25	2.54	2.50	2.52	n/a
CAM-B3LYP	2.22	2.53	2.50	2.52	n/a
M062X	n/a	2.57	2.73	2.58	n/a
SVWN	1.99	2.25	2.01	2.04	n/a
B3PW91	1.97	2.28	1.98	2.01	n/a
B3LYP	2.26	n/a	2.28	2.30	n/a

Table S31: Energy separation (eV) between the ground vibrational states of S_0 and S_1, calculated for $C_{10}N_2$. .

metoda	aug-cc-pVDZ	aug-cc-pVTZ	def2SVPD	def2TZVPD	def2QZVPD
tHCTHhyb	2.08	2.13	2.10	2.12	n/a
VSXC	2.09	2.10	2.09	2.08	n/a
BMK	2.07	2.19	2.09	2.16	n/a
B1B95	2.14	2.18	2.15	2.18	n/a
B3P86	2.13	2.18	2.15	2.17	n/a
B98	2.12	2.17	2.14	2.17	n/a
B971	2.11	2.16	2.13	2.16	n/a
B972	2.17	2.20	2.17	2.20	n/a
wB97X	2.48	2.53	2.49	2.52	n/a
wB97XD	2.34	2.40	2.36	2.39	n/a
CAM-B3LYP	2.35	2.41	2.38	2.40	n/a
M062X	n/a	n/a	n/a	n/a	n/a
SVWN	1.94	1.99	1.96	1.98	n/a
B3PW91	2.13	2.18	2.15	2.17	n/a
B3LYP	2.13	2.18	2.15	2.17	n/a

Table S32: Energy separation (eV) between the ground vibrational states of S_0 and S_2, calculated for $C_{10}N_2$. .

metoda	aug-cc-pVDZ	aug-cc-pVTZ	def2SVPD	def2TZVPD	def2QZVPD
tHCTHhyb	2.18	2.22	2.19	2.21	n/a
VSXC	2.09	2.10	2.10	2.08	n/a
BMK	2.50	2.61	2.52	2.58	n/a
B1B95	2.37	2.40	2.38	2.39	n/a
B3P86	2.25	2.30	2.27	2.29	n/a
B98	2.24	2.29	2.25	2.28	n/a
B971	2.23	2.28	2.25	2.27	n/a
B972	2.29	2.31	2.28	2.30	n/a
wB97X	2.71	2.74	2.71	2.73	n/a
wB97XD	2.53	2.58	2.55	2.58	n/a
CAM-B3LYP	2.52	2.57	2.55	2.57	n/a
M062X	2.76	2.78	2.77	2.81	n/a
SVWN	2.06	2.06	2.10	2.10	n/a
B3PW91	2.26	2.30	2.27	2.29	n/a
B3LYP	2.23	2.28	2.26	2.28	n/a

Table S33: Energy separation (eV) between the ground vibrational states of S_0 and T_1, calculated for $C_{10}N_2$. Experimental value(s): 2.024 eV (Ar) [18] , 2.006 eV (Kr) [18] ..

metoda	aug-cc-pVDZ	aug-cc-pVTZ	def2SVPD	def2TZVPD	def2QZVPD
tHCTHhyb	1.68	1.92	1.69	1.71	n/a
VSXC	1.58	n/a	1.56	1.58	n/a
BMK	2.21	2.31	2.23	2.28	n/a
B1B95	2.08	1.91	2.09	2.10	n/a
B3P86	1.72	1.76	1.96	1.76	n/a
B98	1.96	2.00	1.77	1.79	n/a
B971	1.97	2.00	1.77	1.80	n/a
B972	1.79	2.01	1.79	1.81	n/a
wB97X	2.14	2.39	2.14	2.38	n/a
wB97XD	2.23	2.07	2.23	2.26	n/a
CAM-B3LYP	1.98	2.04	2.23	2.24	n/a
M062X	2.44	2.47	2.29	2.49	n/a
SVWN	1.87	1.73	1.89	1.73	n/a
B3PW91	1.94	1.75	1.72	1.96	n/a
B3LYP	1.75	2.00	1.76	1.78	n/a

Table S34: Energy separation (eV) between the ground vibrational states of S_0 and S_1, calculated for $HC_{11}N$. .

metoda	aug-cc-pVTZ
B972	2.11
wB97X	2.45

Table S35: Energy separation (eV) between the ground vibrational states of S_0 and S_2, calculated for $HC_{11}N$. .

metoda	aug-cc-pVTZ
B972	2.21
wB97X	2.66

Table S36: Energy separation (eV) between the ground vibrational states of S_0 and T_1, calculated for $HC_{11}N$. .

metoda	aug-cc-pVTZ
B972	1.74
wB97X	2.31

Table S37: Energy separation (eV) between the ground vibrational states of S_0 and S_1, calculated for $HC_{13}N$. .

metoda	aug-cc-pVTZ
B972	1.90
wB97X	2.29

Table S38: Energy separation (eV) between the ground vibrational states of S_0 and S_2, calculated for $HC_{13}N$. .

metoda	aug-cc-pVTZ
B972	1.98
wB97X	2.50

Table S39: Energy separation (eV) between the ground vibrational states of S_0 and T_1, calculated for $HC_{13}N$. .

metoda	aug-cc-pVTZ
B972	1.56
wB97X	2.16

Table S40: Energy separation (eV) between the ground vibrational states of S_0 and S_1, calculated for $HC_{15}N$. .

metoda	aug-cc-pVTZ
B972	1.73
wB97X	2.18

Table S41: Energy separation (eV) between the ground vibrational states of S_0 and S_2, calculated for $HC_{15}N$. .

metoda	aug-cc-pVTZ
B972	1.81
wB97X	2.39

Table S42: Energy separation (eV) between the ground vibrational states of S_0 and T_1, calculated for $HC_{15}N$. .

metoda	aug-cc-pVTZ
B972	1.42
wB97X	2.05

Table S43: Energy separation (eV) between the ground vibrational states of S_0 and S_1, calculated for $HC_{17}N$. .

metoda	aug-cc-pVTZ
B972	1.60
wB97X	2.10

Table S44: Energy separation (eV) between the ground vibrational states of S_0 and S_2, calculated for $HC_{17}N$. .

metoda	aug-cc-pVTZ
B972	1.68
wB97X	2.31

Table S45: Energy separation (eV) between the ground vibrational states of S_0 and T_1, calculated for $HC_{17}N$. .

metoda	aug-cc-pVTZ
B972	1.31
wB97X	1.84

Table S46: Energy separation (eV) between the ground vibrational states of S_0 and S_1, calculated for $HC_{19}N$. .

metoda	aug-cc-pVTZ
B972	1.50
wB97X	2.05

Table S47: Energy separation (eV) between the ground vibrational states of S_0 and S_2, calculated for $HC_{19}N$. .

metoda	aug-cc-pVTZ
B972	1.57
wB97X	2.26

Table S48: Energy separation (eV) between the ground vibrational states of S_0 and T_1, calculated for $HC_{19}N$. .

metoda	aug-cc-pVTZ
B972	1.35
wB97X	1.92

Table S49: Energy separation (eV) between the ground vibrational states of S_0 and S_1, calculated for $HC_{21}N$. .

metoda	aug-cc-pVTZ
B972	1.42
wB97X	2.02

Table S50: Energy separation (eV) between the ground vibrational states of S_0 and S_2, calculated for $HC_{21}N$. .

metoda	aug-cc-pVTZ
B972	1.48
wB97X	2.22

Table S51: Energy separation (eV) between the ground vibrational states of S_0 and T_1, calculated for $HC_{21}N$. .

metoda	aug-cc-pVTZ
B972	1.15
wB97X	1.75

Table S52: Energy separation (eV) between the ground vibrational states of S_0 and T_1, calculated for $HC_{23}N$. .

metoda	aug-cc-pVTZ
B972	1.09
wB97X	1.72

Table S53: Energy separation (eV) between the ground vibrational states of S_0 and T_1, calculated for $HC_{25}N$. .

metoda	aug-cc-pVTZ
B972	1.15
wB97X	1.81

Table S54: Energy separation (eV) between the ground vibrational states of S_0 and T_1, calculated for $HC_{27}N$. .

metoda	aug-cc-pVTZ
B972	1.10
wB97X	1.79

Table S55: Energy separation (eV) between the ground vibrational states of S_0 and T_1, calculated for $HC_{29}N$. .

metoda	aug-cc-pVTZ
B972	0.97
wB97X	1.67

Table S56: Energy separation (eV) between the ground vibrational states of S_0 and S_1, calculated for $C_{12}N_2$. .

metoda	aug-cc-pVTZ
B972	1.96
wB97X	2.35

Table S57: Energy separation (eV) between the ground vibrational states of S_0 and S_2, calculated for $C_{12}N_2$. .

metoda	aug-cc-pVTZ
B972	2.06
wB97X	2.56

Table S58: Energy separation (eV) between the ground vibrational states of S_0 and T_1, calculated for $C_{12}N_2$. .

metoda	aug-cc-pVTZ
B972	1.61
wB97X	2.04

Table S59: Energy separation (eV) between the ground vibrational states of S_0 and S_1, calculated for $C_{14}N_2$. .

metoda	aug-cc-pVTZ
B972	1.79
wB97X	2.22

Table S60: Energy separation (eV) between the ground vibrational states of S_0 and S_2, calculated for $C_{14}N_2$. .

metoda	aug-cc-pVTZ
B972	1.87
wB97X	2.43

Table S61: Energy separation (eV) between the ground vibrational states of S_0 and T_1, calculated for $C_{14}N_2$. .

metoda	aug-cc-pVTZ
B972	1.46
wB97X	1.92

Table S62: Energy separation (eV) between the ground vibrational states of S_0 and S_1, calculated for $C_{16}N_2$. .

metoda	aug-cc-pVTZ
B972	1.65
wB97X	2.13

Table S63: Energy separation (eV) between the ground vibrational states of S_0 and S_2, calculated for $C_{16}N_2$. .

metoda	aug-cc-pVTZ
B972	1.72
wB97X	2.34

Table S64: Energy separation (eV) between the ground vibrational states of S_0 and T_1, calculated for $C_{16}N_2$. .

metoda	aug-cc-pVTZ
B972	1.49
wB97X	1.86

Table S65: Energy separation (eV) between the ground vibrational states of S_0 and S_1, calculated for $C_{18}N_2$. .

metoda	aug-cc-pVTZ
B972	1.54
wB97X	2.07

Table S66: Energy separation (eV) between the ground vibrational states of S_0 and S_2, calculated for $C_{18}N_2$. .

metoda	aug-cc-pVTZ
B972	1.61
wB97X	2.28

Table S67: Energy separation (eV) between the ground vibrational states of S_0 and T_1, calculated for $C_{18}N_2$. .

metoda	aug-cc-pVTZ
B972	1.25
wB97X	1.80

Table S68: Energy separation (eV) between the ground vibrational states of S_0 and S_1, calculated for $C_{20}N_2$. .

metoda	aug-cc-pVTZ
B972	1.45
wB97X	2.03

Table S69: Energy separation (eV) between the ground vibrational states of S_0 and S_2, calculated for $C_{20}N_2$. .

metoda	aug-cc-pVTZ
B972	1.51
wB97X	2.24

Table S70: Energy separation (eV) between the ground vibrational states of S_0 and T_1, calculated for $C_{20}N_2$. .

metoda	aug-cc-pVTZ
B972	1.30
wB97X	1.76

Table S71: Energy separation (eV) between the ground vibrational states of S_0 and S_1, calculated for $C_{22}N_2$. .

metoda	aug-cc-pVTZ
B972	1.37
wB97X	n/a

Table S72: Energy separation (eV) between the ground vibrational states of S_0 and S_2, calculated for $C_{22}N_2$. .

metoda	aug-cc-pVTZ
B972	1.44
wB97X	2.20

Table S73: Energy separation (eV) between the ground vibrational states of S_0 and T_1, calculated for $C_{22}N_2$. .

metoda	aug-cc-pVTZ
B972	1.11
wB97X	1.85

Table S74: Energy separation (eV) between the ground vibrational states of S_0 and S_1, calculated for $C_{24}N_2$. .

metoda	aug-cc-pVTZ
B972	1.31
wB97X	1.98

Table S75: Energy separation (eV) between the ground vibrational states of S_0 and S_2, calculated for $C_{24}N_2$. .

metoda	aug-cc-pVTZ
B972	1.37
wB97X	2.18

Table S76: Energy separation (eV) between the ground vibrational states of S_0 and T_1, calculated for $C_{24}N_2$. .

metoda	aug-cc-pVTZ
B972	1.17
wB97X	1.82

Table S77: Energy separation (eV) between the ground vibrational states of S_0 and S_1, calculated for $C_{26}N_2$. .

metoda	aug-cc-pVTZ
B972	1.26
wB97X	n/a

Table S78: Energy separation (eV) between the ground vibrational states of S_0 and S_2, calculated for $C_{26}N_2$. .

metoda	aug-cc-pVTZ
B972	1.32
wB97X	2.16

Table S79: Energy separation (eV) between the ground vibrational states of S_0 and T_1, calculated for $C_{26}N_2$. .

metoda	aug-cc-pVTZ
B972	1.12
wB97X	1.79

Table S80: Energy separation (eV) between the ground vibrational states of S_0 and S_1, calculated for $C_{28}N_2$. .

metoda	aug-cc-pVTZ
B972	1.22
wB97X	n/a

Table S81: Energy separation (eV) between the ground vibrational states of S_0 and S_2, calculated for $C_{28}N_2$. .

metoda	aug-cc-pVTZ
B972	1.27
wB97X	2.15

Table S82: Energy separation (eV) between the ground vibrational states of S_0 and T_1, calculated for $C_{28}N_2$. .

metoda	aug-cc-pVTZ
B972	1.08
wB97X	1.77

Table S83: Energy separation (eV) between the ground vibrational states of S_0 and S_1, calculated for $C_{30}N_2$. .

metoda	aug-cc-pVTZ
B972	1.18
wB97X	n/a

Table S84: Energy separation (eV) between the ground vibrational states of S_0 and S_2, calculated for $C_{30}N_2$. .

metoda	aug-cc-pVTZ
B972	1.23
wB97X	2.14

Table S85: Energy separation (eV) between the ground vibrational states of S_0 and T_1, calculated for $C_{30}N_2$. .

metoda	aug-cc-pVTZ
B972	0.95
wB97X	1.66

Table S86: Harmonic unscaled vibrational frequencies (in cm^{-1}) for S_1 of HC_1N

method	basis	1	2	3
tHCTHhyb	aug-cc-pVDZ	2581.7	1515.7	971.4
VSXC	aug-cc-pVDZ	2436.9	1502.1	976.1
BMK	aug-cc-pVDZ	2798.5	1514.1	954.9
B1B95	aug-cc-pVDZ	2723.6	1549.2	964.1
B3P86	aug-cc-pVDZ	2668.2	1544.7	973.5
B98	aug-cc-pVDZ	2645.8	1522.4	972.8
B971	aug-cc-pVDZ	2642.4	1520.1	970.5
B972	aug-cc-pVDZ	2680.3	1543.9	978.0
wB97X	aug-cc-pVDZ	2806.8	1564.0	972.9
wB97XD	aug-cc-pVDZ	2734.4	1550.8	974.1
CAM-B3LYP	aug-cc-pVDZ	2770.7	1560.7	974.5
M062X	aug-cc-pVDZ	2934.6	1433.9	874.4
SVWN	aug-cc-pVDZ	2427.2	1532.1	939.8
B3PW91	aug-cc-pVDZ	2659.4	1540.2	972.4
B3LYP	aug-cc-pVDZ	2646.8	1524.7	975.0
tHCTHhyb	aug-cc-pVTZ	2594.9	1520.5	985.5
VSXC	aug-cc-pVTZ	2462.8	1499.8	995.4
BMK	aug-cc-pVTZ	2794.1	1527.7	974.6
B1B95	aug-cc-pVTZ	2726.7	1549.2	977.2
B3P86	aug-cc-pVTZ	2673.2	1547.2	987.0
B98	aug-cc-pVTZ	2649.5	1526.3	986.7
B971	aug-cc-pVTZ	2645.3	1522.8	983.6
B972	aug-cc-pVTZ	2684.7	1544.0	990.3
wB97X	aug-cc-pVTZ	2817.3	1564.8	982.6
wB97XD	aug-cc-pVTZ	2746.5	1553.3	986.4
CAM-B3LYP	aug-cc-pVTZ	2777.6	1564.6	987.6
M062X	aug-cc-pVTZ	2928.6	1440.9	891.4
SVWN	aug-cc-pVTZ	2422.2	1532.8	965.5
B3PW91	aug-cc-pVTZ	2663.0	1542.2	985.5
B3LYP	aug-cc-pVTZ	2656.2	1528.3	989.0
tHCTHhyb	def2SVPD	2606.2	1556.1	987.4
VSXC	def2SVPD	2451.8	1542.0	993.7
BMK	def2SVPD	2827.1	1558.4	967.0
B1B95	def2SVPD	2746.5	1589.7	978.8
B3P86	def2SVPD	2689.8	1583.0	987.8
B98	def2SVPD	2669.6	1563.1	988.4
B971	def2SVPD	2665.0	1560.4	985.9
B972	def2SVPD	2702.4	1582.9	993.9
wB97X	def2SVPD	2827.2	1603.0	985.2
wB97XD	def2SVPD	2758.3	1590.5	988.4
CAM-B3LYP	def2SVPD	2789.6	1601.4	987.6
M062X	def2SVPD	2957.1	1478.9	884.7
SVWN	def2SVPD	2451.0	1574.8	955.2
B3PW91	def2SVPD	2681.1	1578.7	987.4
B3LYP	def2SVPD	2667.3	1566.2	990.1
tHCTHhyb	def2TZVPD	2593.7	1523.7	985.3
VSXC	def2TZVPD	2445.6	1505.2	989.5
BMK	def2TZVPD	2813.3	1526.1	973.5
B1B95	def2TZVPD	2724.2	1554.1	976.8
B3P86	def2TZVPD	2668.3	1551.3	986.7
B98	def2TZVPD	2650.7	1530.0	986.7
B971	def2TZVPD	2646.4	1526.7	984.1
B972	def2TZVPD	2680.5	1548.3	990.1
wB97X	def2TZVPD	2809.5	1569.9	983.5
wB97XD	def2TZVPD	2739.8	1559.2	986.2
CAM-B3LYP	def2TZVPD	2771.3	1568.9	987.3
M062X	def2TZVPD	2928.6	1445.3	886.5
SVWN	def2TZVPD	2415.4	1540.4	959.7
B3PW91	def2TZVPD	2659.6	1546.4	985.3
B3LYP	def2TZVPD	2652.4	1532.9	989.3
tHCTHhyb	def2QZVPD	2593.9	1522.1	985.5
VSXC	def2QZVPD	2508.8	1500.9	978.7
BMK	def2QZVPD	2806.9	1524.0	973.1
B1B95	def2QZVPD	2726.7	1552.4	976.5
B3P86	def2QZVPD	2672.9	1549.4	986.0
B98	def2QZVPD	2653.2	1528.7	986.4
B971	def2QZVPD	2648.4	1525.3	982.8
B972	def2QZVPD	2683.6	1546.7	990.3
wB97X	def2QZVPD	2809.7	1567.5	985.9
wB97XD	def2QZVPD	2742.5	1556.9	994.9
CAM-B3LYP	def2QZVPD	2767.8	1566.6	990.8
M062X	def2QZVPD	2928.1	1445.7	895.6
SVWN	def2QZVPD	2383.0	1535.3	942.4
B3PW91	def2QZVPD	2664.5	1544.4	984.1
B3LYP	def2QZVPD	2657.7	1530.7	988.0
gas[1]		n/a	1488.8	940.3

Table S87: Harmonic unscaled vibrational frequencies (in cm^{-1}) for S_2 of HC_1N

method	basis	1	2	3
tHCTHhyb	aug-cc-pVDZ	2354.5	1500.4	717.8
VSXC	aug-cc-pVDZ	2047.1	1425.4	773.0
BMK	aug-cc-pVDZ	2598.9	1531.4	776.9
B1B95	aug-cc-pVDZ	2476.8	1556.8	732.0
B3P86	aug-cc-pVDZ	2449.3	1542.3	715.9
B98	aug-cc-pVDZ	2476.5	1524.7	678.2
B971	aug-cc-pVDZ	2452.6	1519.6	691.0
B972	aug-cc-pVDZ	2476.9	1547.5	706.9
wB97X	aug-cc-pVDZ	2739.1	1600.5	641.8
wB97XD	aug-cc-pVDZ	2622.3	1573.9	652.4
CAM-B3LYP	aug-cc-pVDZ	2692.1	1589.0	645.6
M062X	aug-cc-pVDZ	2587.1	1521.5	820.9
SVWN	aug-cc-pVDZ	1891.4	1430.6	824.8
B3PW91	aug-cc-pVDZ	2432.0	1537.4	719.3
B3LYP	aug-cc-pVDZ	2463.3	1523.5	694.8
tHCTHhyb	aug-cc-pVTZ	2369.9	1499.9	732.8
VSXC	aug-cc-pVTZ	2057.3	1420.8	777.9
BMK	aug-cc-pVTZ	2580.1	1548.0	796.1
B1B95	aug-cc-pVTZ	2489.1	1546.9	744.5
B3P86	aug-cc-pVTZ	2451.2	1535.8	729.4
B98	aug-cc-pVTZ	2478.7	1522.9	690.5
B971	aug-cc-pVTZ	2455.0	1516.9	703.0
B972	aug-cc-pVTZ	2486.9	1541.8	713.9
wB97X	aug-cc-pVTZ	2754.3	1595.4	641.8
wB97XD	aug-cc-pVTZ	2631.3	1568.0	660.1
CAM-B3LYP	aug-cc-pVTZ	2707.6	1588.5	644.8
M062X	aug-cc-pVTZ	2604.4	1526.7	835.4
SVWN	aug-cc-pVTZ	1852.2	1401.9	856.7
B3PW91	aug-cc-pVTZ	2437.2	1532.7	733.4
B3LYP	aug-cc-pVTZ	2479.4	1521.8	702.2
tHCTHhyb	def2SVPD	2381.5	1535.7	751.9
VSXC	def2SVPD	2070.9	1445.2	809.9
BMK	def2SVPD	2629.4	1587.2	800.3
B1B95	def2SVPD	2511.6	1595.7	766.2
B3P86	def2SVPD	2478.1	1576.5	746.6
B98	def2SVPD	2504.9	1561.9	704.2
B971	def2SVPD	2480.5	1556.5	719.0
B972	def2SVPD	2506.7	1581.9	735.7
wB97X	def2SVPD	2776.5	1638.2	648.2
wB97XD	def2SVPD	2654.8	1610.4	670.0
CAM-B3LYP	def2SVPD	2724.6	1628.8	654.2
M062X	def2SVPD	2635.7	1574.3	824.9
SVWN	def2SVPD	1915.7	1461.1	871.0
B3PW91	def2SVPD	2460.1	1571.5	752.1
B3LYP	def2SVPD	2492.1	1561.2	718.9
tHCTHhyb	def2TZVPD	2364.8	1503.1	737.6
VSXC	def2TZVPD	2046.2	1428.3	801.6
BMK	def2TZVPD	2593.2	1545.6	803.3
B1B95	def2TZVPD	2480.4	1556.0	750.4
B3P86	def2TZVPD	2445.1	1542.2	735.5
B98	def2TZVPD	2474.7	1526.6	695.0
B971	def2TZVPD	2451.4	1520.8	708.6
B972	def2TZVPD	2477.6	1546.3	720.5
wB97X	def2TZVPD	2748.6	1601.4	643.2
wB97XD	def2TZVPD	2624.9	1574.5	662.5
CAM-B3LYP	def2TZVPD	2697.5	1593.1	649.0
M062X	def2TZVPD	2593.6	1529.0	848.3
SVWN	def2TZVPD	1864.8	1401.7	855.6
B3PW91	def2TZVPD	2428.6	1536.8	738.8
B3LYP	def2TZVPD	2468.9	1526.3	708.2
tHCTHhyb	def2QZVPD	2368.0	1501.1	739.8
VSXC	def2QZVPD	2120.1	1420.4	764.7
BMK	def2QZVPD	2583.5	1544.1	803.7
B1B95	def2QZVPD	2483.8	1553.3	750.6
B3P86	def2QZVPD	2448.6	1539.8	734.2
B98	def2QZVPD	2477.1	1524.9	696.5
B971	def2QZVPD	2451.8	1519.0	708.5
B972	def2QZVPD	2480.3	1544.0	721.4
wB97X	def2QZVPD	2750.3	1598.5	639.6
wB97XD	def2QZVPD	2629.6	1572.1	662.4
CAM-B3LYP	def2QZVPD	2699.3	1590.4	648.3
M062X	def2QZVPD	2602.9	1530.3	838.8
SVWN	def2QZVPD	1848.5	1390.3	841.4
B3PW91	def2QZVPD	2432.3	1534.5	737.5
B3LYP	def2QZVPD	2473.2	1523.7	709.7

Table S88: Harmonic unscaled vibrational frequencies (in cm^{-1}) for S_0 of HC_1N

method	basis	1	2	3
tHCTHhyb	aug-cc-pVDZ	3434.4	2165.2	719.5
VSXC	aug-cc-pVDZ	3447.4	2148.4	715.0
BMK	aug-cc-pVDZ	3467.1	2238.9	732.2
B1B95	aug-cc-pVDZ	3475.1	2216.7	741.3
B3P86	aug-cc-pVDZ	3460.6	2197.8	735.7
B98	aug-cc-pVDZ	3448.4	2184.3	725.4
B971	aug-cc-pVDZ	3446.0	2178.6	723.8
B972	aug-cc-pVDZ	3475.1	2201.1	743.1
wB97X	aug-cc-pVDZ	3475.0	2233.7	754.6
wB97XD	aug-cc-pVDZ	3465.4	2214.1	746.0
CAM-B3LYP	aug-cc-pVDZ	3468.6	2233.3	746.2
M062X	aug-cc-pVDZ	3471.7	2248.5	759.4
SVWN	aug-cc-pVDZ	3375.6	2146.5	701.7
B3PW91	aug-cc-pVDZ	3456.3	2193.7	736.2
B3LYP	aug-cc-pVDZ	3449.3	2186.7	728.8
tHCTHhyb	aug-cc-pVTZ	3426.3	2179.5	746.9
VSXC	aug-cc-pVTZ	3441.4	2155.2	744.5
BMK	aug-cc-pVTZ	3440.3	2256.7	764.2
B1B95	aug-cc-pVTZ	3466.3	2224.8	768.5
B3P86	aug-cc-pVTZ	3453.1	2208.9	762.9
B98	aug-cc-pVTZ	3438.3	2198.0	754.1
B971	aug-cc-pVTZ	3435.4	2191.1	752.6
B972	aug-cc-pVTZ	3467.4	2208.7	769.5
wB97X	aug-cc-pVTZ	3470.6	2244.7	783.4
wB97XD	aug-cc-pVTZ	3462.1	2229.4	774.0
CAM-B3LYP	aug-cc-pVTZ	3460.8	2245.4	775.9
M062X	aug-cc-pVTZ	3466.5	2254.6	785.3
SVWN	aug-cc-pVTZ	3356.7	2159.7	725.1
B3PW91	aug-cc-pVTZ	3446.5	2204.1	762.8
B3LYP	aug-cc-pVTZ	3444.1	2199.8	759.2
tHCTHhyb	def2SVPD	3429.8	2199.6	729.3
VSXC	def2SVPD	3440.5	2179.7	716.8
BMK	def2SVPD	3460.5	2271.0	751.8
B1B95	def2SVPD	3468.7	2250.0	751.5
B3P86	def2SVPD	3455.0	2230.0	746.1
B98	def2SVPD	3443.0	2218.0	736.0
B971	def2SVPD	3440.6	2212.2	734.3
B972	def2SVPD	3469.1	2234.6	750.3
wB97X	def2SVPD	3469.1	2265.9	767.8
wB97XD	def2SVPD	3460.8	2247.0	757.3
CAM-B3LYP	def2SVPD	3462.1	2266.1	760.1
M062X	def2SVPD	3467.7	2282.9	769.3
SVWN	def2SVPD	3373.3	2181.9	717.0
B3PW91	def2SVPD	3450.5	2226.2	745.6
B3LYP	def2SVPD	3444.2	2220.6	741.7
tHCTHhyb	def2TZVPD	3427.0	2182.5	747.5
VSXC	def2TZVPD	3436.3	2159.0	748.2
BMK	def2TZVPD	3460.3	2258.4	767.7
B1B95	def2TZVPD	3466.1	2229.2	770.0
B3P86	def2TZVPD	3452.7	2212.5	764.4
B98	def2TZVPD	3440.8	2200.9	755.0
B971	def2TZVPD	3438.2	2194.1	753.9
B972	def2TZVPD	3465.0	2213.1	770.6
wB97X	def2TZVPD	3466.7	2247.6	786.1
wB97XD	def2TZVPD	3457.2	2231.7	775.0
CAM-B3LYP	def2TZVPD	3460.5	2248.7	777.8
M062X	def2TZVPD	3470.4	2258.4	787.0
SVWN	def2TZVPD	3356.8	2164.3	726.0
B3PW91	def2TZVPD	3446.2	2207.9	763.7
B3LYP	def2TZVPD	3443.8	2203.3	761.0
tHCTHhyb	def2QZVPD	3423.8	2180.9	746.4
VSXC	def2QZVPD	3434.4	2155.1	742.8
BMK	def2QZVPD	3448.6	2256.6	764.2
B1B95	def2QZVPD	3462.6	2226.7	767.8
B3P86	def2QZVPD	3449.7	2210.4	761.6
B98	def2QZVPD	3437.0	2199.8	753.8
B971	def2QZVPD	3433.9	2192.8	752.3
B972	def2QZVPD	3462.8	2210.6	768.7
wB97X	def2QZVPD	3463.4	2245.5	782.3
wB97XD	def2QZVPD	3455.3	2230.6	773.8
CAM-B3LYP	def2QZVPD	3457.3	2246.4	774.7
M062X	def2QZVPD	3468.8	2256.5	786.5
SVWN	def2QZVPD	3354.7	2161.6	722.2
B3PW91	def2QZVPD	3443.6	2205.6	761.6
B3LYP	def2QZVPD	3440.6	2201.1	758.1
gas[19]		3311.47	2096.85	711.98

Table S89: Harmonic unscaled vibrational frequencies (in cm^{-1}) for S_1 of HC_3N

method	basis	1	2	3	4	5	6	7	8	9
tHCTHhyb	aug-cc-pVDZ	3035.9	2037.2	1592.1	936.2	892.0	484.8	168.3	635.3	284.9
VSXC	aug-cc-pVDZ	3038.3	2040.6	1586.0	940.2	896.4	477.5	157.2	620.2	276.4
BMK	aug-cc-pVDZ	3100.9	2064.4	1564.0	930.8	872.5	503.4	184.5	699.9	299.6
B1B95	aug-cc-pVDZ	3100.9	2073.2	1647.4	928.9	891.7	483.9	164.0	625.1	285.7
B3P86	aug-cc-pVDZ	3082.7	2069.2	1638.4	934.0	893.4	478.9	162.9	619.8	282.4
B98	aug-cc-pVDZ	3058.7	2044.3	1605.2	936.0	893.4	490.0	172.6	647.1	290.3
B971	aug-cc-pVDZ	3057.5	2041.2	1603.2	934.5	893.1	487.9	171.1	642.9	288.2
B972	aug-cc-pVDZ	3093.3	2068.0	1634.0	935.7	900.2	486.5	166.2	627.4	286.3
wB97X	aug-cc-pVDZ	3130.2	2129.4	1636.2	945.1	884.6	516.7	187.2	687.4	303.8
wB97XD	aug-cc-pVDZ	3100.8	2092.3	1637.7	939.9	891.7	503.8	177.9	667.3	297.8
CAM-B3LYP	aug-cc-pVDZ	3118.3	2101.1	1653.9	936.3	892.5	505.4	177.8	664.2	301.1
M062X	aug-cc-pVDZ	3145.8	2034.0	1547.5	880.2	850.6	497.0	161.0	633.4	300.3
SVWN	aug-cc-pVDZ	3001.7	2093.0	1628.0	940.6	842.0	432.5	141.4	565.7	261.9
B3PW91	aug-cc-pVDZ	3075.9	2064.0	1634.3	931.6	893.1	479.3	163.4	619.8	282.5
B3LYP	aug-cc-pVDZ	3069.3	2047.9	1621.1	931.8	895.7	483.2	167.0	629.1	288.0
tHCTHhyb	aug-cc-pVTZ	3037.2	2037.7	1611.1	923.6	894.6	476.5	157.4	618.7	283.4
VSXC	aug-cc-pVTZ	3048.7	2029.4	1591.1	912.6	862.0	522.8	-227.5	638.2	284.3
BMK	aug-cc-pVTZ	3080.8	2058.5	1590.5	924.6	874.4	503.6	174.6	681.1	304.8
B1B95	aug-cc-pVTZ	3099.0	2068.9	1658.5	920.3	883.3	473.4	159.8	608.5	281.1
B3P86	aug-cc-pVTZ	3081.8	2072.1	1651.1	926.8	889.0	469.0	156.7	604.5	279.2
B98	aug-cc-pVTZ	3055.1	2041.5	1622.3	925.4	897.1	483.6	163.2	630.8	290.4
B971	aug-cc-pVTZ	3053.4	2038.2	1619.0	923.7	896.3	480.9	163.3	628.0	289.2
B972	aug-cc-pVTZ	3091.1	2065.8	1645.9	924.1	899.3	477.4	159.4	613.1	283.3
wB97X	aug-cc-pVTZ	3132.3	2110.9	1656.1	931.8	886.4	514.0	176.7	671.7	308.3
wB97XD	aug-cc-pVTZ	3106.4	2079.7	1659.4	925.2	894.8	498.5	164.8	649.1	298.6
CAM-B3LYP	aug-cc-pVTZ	3117.0	2088.4	1672.2	924.6	894.5	501.1	160.6	648.6	303.4
M062X	aug-cc-pVTZ	3151.8	1979.5	1607.8	884.3	811.0	471.7	125.2	592.2	290.2
SVWN	aug-cc-pVTZ	2981.6	2108.3	1639.6	932.1	816.5	426.3	131.6	583.8	280.4
B3PW91	aug-cc-pVTZ	3073.5	2065.1	1646.1	923.6	889.8	469.7	158.2	605.2	279.2
B3LYP	aug-cc-pVTZ	3069.5	2049.0	1636.6	922.8	897.5	476.8	159.3	614.9	287.0
tHCTHhyb	def2SVPD	3052.2	2070.0	1635.0	930.4	883.1	467.0	163.9	615.8	282.0
VSXC	def2SVPD	3046.6	2075.4	1622.5	939.2	884.4	456.1	160.9	604.9	274.9
BMK	def2SVPD	3110.4	2084.3	1624.7	913.5	879.3	491.1	173.6	681.1	303.7
B1B95	def2SVPD	3117.2	2104.6	1684.9	933.8	866.6	466.0	159.2	603.1	280.6
B3P86	def2SVPD	3098.1	2102.8	1674.0	937.1	872.6	460.5	158.4	601.4	277.6
B98	def2SVPD	3073.9	2074.0	1646.3	929.2	887.5	474.7	167.1	627.1	288.7
B971	def2SVPD	3072.3	2071.6	1643.7	928.8	885.7	472.8	166.6	623.2	286.6
B972	def2SVPD	3109.3	2099.1	1671.0	936.3	884.4	470.5	164.5	608.3	281.2
wB97X	def2SVPD	3142.6	2144.0	1678.3	930.4	881.4	504.7	179.9	661.9	303.7
wB97XD	def2SVPD	3117.4	2111.9	1679.8	930.0	885.6	488.6	171.1	641.9	294.6
CAM-B3LYP	def2SVPD	3128.7	2121.3	1692.6	929.6	881.5	492.2	167.5	642.3	301.4
M062X	def2SVPD	3174.4	2012.3	1622.5	891.8	782.7	465.7	133.2	589.6	289.7
SVWN	def2SVPD	3016.5	2151.7	1668.2	941.1	800.0	405.3	140.2	563.7	251.4
B3PW91	def2SVPD	3091.5	2097.2	1669.7	934.7	873.7	461.8	159.8	602.3	278.1
B3LYP	def2SVPD	3081.4	2080.3	1658.8	930.4	880.8	466.2	159.0	609.9	285.7
tHCTHhyb	def2TZVPD	3040.8	2040.0	1612.1	924.2	894.3	479.6	159.4	619.2	285.3
VSXC	def2TZVPD	3028.3	2046.4	1602.6	921.7	892.9	472.3	154.0	610.6	293.4
BMK	def2TZVPD	3104.6	2062.8	1596.5	927.7	878.7	507.3	177.3	685.0	307.5
B1B95	def2TZVPD	3101.1	2071.6	1660.9	922.5	883.1	475.1	151.3	607.6	282.5
B3P86	def2TZVPD	3083.6	2074.5	1653.1	928.2	887.5	471.4	155.6	604.7	279.8
B98	def2TZVPD	3059.7	2044.2	1623.2	926.1	897.3	485.9	164.1	632.5	291.9
B971	def2TZVPD	3058.2	2040.8	1620.1	924.5	896.1	483.6	161.6	628.7	290.0
B972	def2TZVPD	3091.7	2067.9	1647.7	924.9	897.2	478.5	158.3	612.6	284.0
wB97X	def2TZVPD	3131.2	2114.3	1657.1	932.9	887.1	516.1	174.2	672.0	310.6
wB97XD	def2TZVPD	3105.8	2083.2	1659.9	927.4	894.9	499.9	163.9	649.1	299.9
CAM-B3LYP	def2TZVPD	3116.8	2092.6	1672.6	927.0	895.0	503.7	162.1	650.0	305.3
M062X	def2TZVPD	3150.7	1987.6	1602.8	882.8	804.9	477.1	123.8	596.4	290.9
SVWN	def2TZVPD	2978.7	2110.4	1642.8	937.1	822.5	437.7	134.2	587.5	257.7
B3PW91	def2TZVPD	3075.7	2067.7	1648.1	925.0	887.6	471.8	156.2	605.1	279.9
B3LYP	def2TZVPD	3071.3	2051.7	1638.2	923.8	896.6	478.3	157.0	615.7	288.4
tHCTHhyb	def2QZVPD	3038.7	2036.8	1610.5	920.5	890.9	476.3	157.3	618.6	284.3
BMK	def2QZVPD	3096.7	2055.3	1599.0	920.7	877.7	502.5	168.8	674.4	305.5
B1B95	def2QZVPD	3099.8	2067.4	1657.6	921.3	885.2	477.6	143.6	608.0	283.9
B3P86	def2QZVPD	3082.0	2070.4	1650.5	926.7	879.8	470.5	152.1	604.0	278.3
B98	def2QZVPD	3057.3	2041.7	1622.5	920.9	892.4	484.1	159.2	630.8	290.4
B971	def2QZVPD	3055.6	2038.3	1618.9	920.7	890.9	482.6	160.7	627.3	288.8
B972	def2QZVPD	3090.3	2064.3	1644.9	923.3	892.0	479.1	153.2	612.7	283.6
wB97X	def2QZVPD	3125.8	2112.3	1652.0	935.5	886.8	515.6	179.2	671.9	306.7
wB97XD	def2QZVPD	3103.8	2080.6	1656.1	931.8	896.7	499.6	167.6	651.3	297.1
CAM-B3LYP	def2QZVPD	3114.5	2088.7	1670.3	926.0	896.4	504.1	168.0	648.2	301.0
M062X	def2QZVPD	3150.6	1985.5	1602.6	881.9	807.8	475.8	113.0	600.5	290.2
SVWN	def2QZVPD	2977.8	2111.4	1637.9	922.4	806.5	450.9	221.5	624.3	316.4
B3PW91	def2QZVPD	3074.8	2063.8	1645.5	923.7	881.4	471.2	149.6	604.4	278.5
B3LYP	def2QZVPD	3069.0	2047.9	1635.8	921.9	892.7	477.4	151.6	614.3	286.8
gas[3]		n/a	n/a	n/a	n/a	884.4	n/a	160.3	n/a	n/a

Table S90: Harmonic unscaled vibrational frequencies (in cm^{-1}) for S_2 of HC_3N

method	basis	1	2	3	4	5	6	7
tHCTHhyb	aug-cc-pVDZ	3445.5	2194.2	1600.5	940.1	386.7	221.6	-193.7
VSXC	aug-cc-pVDZ	3475.1	2188.5	1614.9	940.6	463.7	302.3	-138.7
BMK	aug-cc-pVDZ	3431.4	2213.0	1602.8	944.1	400.1	226.8	-130.1
B1B95	aug-cc-pVDZ	3553.2	2244.4	1625.9	962.5	366.0	242.3	-654.2
B3P86	aug-cc-pVDZ	3470.9	2230.6	1624.9	957.0	405.8	226.6	-79.8
B98	aug-cc-pVDZ	3456.5	2204.4	1598.0	946.7	397.8	225.2	-158.7
B971	aug-cc-pVDZ	3456.4	2203.0	1599.3	945.8	395.1	224.7	-163.6
B972	aug-cc-pVDZ	3488.3	2229.6	1622.5	957.4	407.2	227.4	-122.6
wB97X	aug-cc-pVDZ	3474.1	2239.9	1610.9	960.1	427.9	229.7	151.9
wB97XD	aug-cc-pVDZ	3464.5	2238.5	1610.8	959.1	417.9	228.5	94.8
CAM-B3LYP	aug-cc-pVDZ	3472.1	2237.8	1614.3	962.7	428.8	230.4	142.7
M062X	aug-cc-pVDZ	3481.7	2251.9	1620.0	966.0	409.2	252.0	182.9
SVWN	aug-cc-pVDZ	3388.5	2209.7	1651.0	942.2	416.2	201.3	169.3
B3PW91	aug-cc-pVDZ	3467.3	2225.7	1622.0	954.9	405.7	226.8	-93.9
B3LYP	aug-cc-pVDZ	3461.0	2207.5	1604.8	950.4	404.2	224.9	-108.6
tHCTHhyb	aug-cc-pVTZ	3433.2	2184.5	1599.7	942.1	424.5	241.2	184.4
VSXC	aug-cc-pVTZ	3467.5	2177.8	1613.4	937.9	1092.2	212.0	-42.1
BMK	aug-cc-pVTZ	3426.4	2201.3	1600.7	947.0	435.6	244.5	198.3
B1B95	aug-cc-pVTZ	3467.2	2229.0	1622.5	960.9	442.7	262.3	230.5
B3P86	aug-cc-pVTZ	3459.4	2218.8	1622.2	957.8	439.1	257.0	225.2
B98	aug-cc-pVTZ	3441.5	2194.1	1597.1	948.5	431.4	253.7	207.9
B971	aug-cc-pVTZ	3440.3	2191.5	1597.4	947.0	428.5	255.2	204.9
B972	aug-cc-pVTZ	3476.5	2216.4	1618.0	956.6	438.9	254.1	221.6
wB97X	aug-cc-pVTZ	3465.9	2225.4	1605.5	959.3	459.4	320.1	240.2
wB97XD	aug-cc-pVTZ	3458.2	2226.3	1608.4	959.8	451.1	276.7	237.7
CAM-B3LYP	aug-cc-pVTZ	3460.1	2225.3	1611.5	963.6	458.7	306.0	236.1
M062X	aug-cc-pVTZ	3462.5	2235.3	1612.0	964.2	445.3	351.3	234.4
SVWN	aug-cc-pVTZ	3367.4	2197.5	1649.0	943.2	705.6	392.6	105.5
B3PW91	aug-cc-pVTZ	3453.9	2212.6	1618.5	955.1	437.5	255.5	227.5
B3LYP	aug-cc-pVTZ	3450.8	2196.3	1603.6	952.0	440.9	259.0	226.5
tHCTHhyb	def2SVPD	3431.0	2208.5	1625.9	945.3	477.2	247.9	220.4
VSXC	def2SVPD	3458.7	2199.3	1634.3	944.0	484.2	342.5	219.4
BMK	def2SVPD	3444.1	2230.6	1632.7	948.9	513.2	268.8	235.6
B1B95	def2SVPD	3465.8	2256.7	1652.3	965.9	490.2	274.1	247.6
B3P86	def2SVPD	3456.2	2242.3	1648.2	960.8	494.2	269.5	248.0
B98	def2SVPD	3442.1	2218.0	1623.8	951.3	485.6	252.6	239.2
B971	def2SVPD	3441.7	2216.4	1624.7	950.4	478.7	249.2	235.5
B972	def2SVPD	3473.2	2241.0	1645.3	961.3	477.9	258.3	244.7
wB97X	def2SVPD	3460.2	2249.8	1636.2	961.0	496.9	315.3	247.6
wB97XD	def2SVPD	3451.3	2250.3	1636.2	961.5	497.7	292.3	249.1
CAM-B3LYP	def2SVPD	3457.8	2249.7	1639.4	965.0	517.3	315.4	255.1
M062X	def2SVPD	3470.3	2262.7	1641.6	969.3	491.4	381.0	252.3
SVWN	def2SVPD	3374.4	2227.0	1676.2	947.2	525.0	364.6	258.3
B3PW91	def2SVPD	3452.1	2237.7	1645.0	958.8	490.5	263.7	246.6
B3LYP	def2SVPD	3447.1	2220.6	1629.5	954.5	496.2	266.1	249.8
tHCTHhyb	def2TZVPD	3438.6	2186.6	1601.2	942.5	422.7	233.8	180.6
VSXC	def2TZVPD	3464.8	2177.9	1614.9	941.1	770.4	222.6	-764.3
BMK	def2TZVPD	3457.4	2207.3	1604.0	946.9	440.1	241.8	200.0
B1B95	def2TZVPD	3472.5	2231.2	1624.5	961.9	442.2	247.4	231.7
B3P86	def2TZVPD	3463.4	2221.2	1624.3	958.3	440.5	245.9	230.0
B98	def2TZVPD	3448.9	2196.2	1598.9	948.7	432.9	238.4	199.3
B971	def2TZVPD	3448.2	2193.6	1599.3	947.3	429.7	237.7	196.1
B972	def2TZVPD	3479.0	2217.4	1619.8	957.4	438.2	243.7	222.2
wB97X	def2TZVPD	3468.9	2227.1	1608.9	959.7	461.7	300.4	235.7
wB97XD	def2TZVPD	3459.8	2228.3	1611.6	960.0	450.0	273.4	235.5
CAM-B3LYP	def2TZVPD	3464.5	2228.3	1613.8	963.9	459.9	298.1	238.3
M062X	def2TZVPD	3469.7	2238.9	1611.0	965.3	444.0	352.0	235.5
SVWN	def2TZVPD	3370.4	2199.9	1649.4	943.7	392.5	241.7	-205.8
B3PW91	def2TZVPD	3457.7	2215.2	1620.5	955.8	439.0	245.0	227.9
B3LYP	def2TZVPD	3455.1	2199.3	1605.6	952.6	442.2	245.3	227.7
tHCTHhyb	def2QZVPD	3434.5	2183.0	1599.0	941.9	420.7	232.3	177.8
VSXC	def2QZVPD	3426.5	2158.0	1663.8	935.8	882.5	-249.6	-380.0
BMK	def2QZVPD	3445.1	2202.4	1599.3	947.4	431.8	243.0	228.4
B1B95	def2QZVPD	3468.7	2226.9	1621.1	960.8	436.6	234.6	192.8
B3P86	def2QZVPD	3459.2	2216.9	1621.3	957.6	437.1	243.9	218.3
B98	def2QZVPD	3444.3	2192.9	1596.8	948.4	426.2	229.7	147.9
B971	def2QZVPD	3443.2	2190.3	1596.9	946.9	424.7	227.9	115.9
B972	def2QZVPD	3476.1	2214.2	1617.2	956.5	433.6	247.4	213.5
wB97X	def2QZVPD	3464.4	2223.0	1604.7	958.1	455.7	270.8	219.1
wB97XD	def2QZVPD	3457.0	2224.3	1607.4	958.8	444.1	262.2	232.7
CAM-B3LYP	def2QZVPD	3461.2	2223.1	1610.3	962.7	458.5	295.5	235.7
M062X	def2QZVPD	3469.4	2233.2	1609.3	964.3	438.7	362.9	232.9
B3PW91	def2QZVPD	3454.3	2211.1	1617.8	954.8	436.1	238.1	209.9
B3LYP	def2QZVPD	3450.8	2195.1	1603.1	951.7	436.3	226.2	173.6
gas[4]		n/a	2120	n/a	953	414	290	226

Table S91: Harmonic unscaled vibrational frequencies (in cm^{-1}) for S_0 of HC_3N

method	basis	1	2	3	4	5	6	7
tHCTHhyb	aug-cc-pVDZ	3460.5	2343.0	2139.5	900.2	625.7	502.6	219.5
VSXC	aug-cc-pVDZ	3486.4	2318.4	2131.4	906.4	612.3	503.9	227.3
BMK	aug-cc-pVDZ	3482.5	2420.5	2171.6	899.1	655.9	513.1	220.4
B1B95	aug-cc-pVDZ	3494.6	2409.7	2193.5	913.3	662.1	525.4	226.6
B3P86	aug-cc-pVDZ	3482.9	2385.3	2176.4	913.0	652.5	520.7	224.9
B98	aug-cc-pVDZ	3471.9	2366.7	2159.9	902.4	639.5	508.7	221.8
B971	aug-cc-pVDZ	3470.7	2361.1	2155.6	901.9	637.6	507.4	221.6
B972	aug-cc-pVDZ	3499.3	2388.6	2180.3	911.5	655.7	521.6	226.4
wB97X	aug-cc-pVDZ	3488.3	2455.1	2224.6	899.9	699.2	531.7	226.9
wB97XD	aug-cc-pVDZ	3481.7	2426.8	2203.9	902.4	678.4	524.0	223.8
CAM-B3LYP	aug-cc-pVDZ	3485.5	2439.7	2216.7	906.6	685.5	532.7	227.5
M062X	aug-cc-pVDZ	3487.2	2452.3	2222.8	904.4	686.6	529.2	222.9
SVWN	aug-cc-pVDZ	3402.1	2331.4	2121.9	918.1	613.3	517.0	220.4
B3PW91	aug-cc-pVDZ	3479.1	2380.4	2172.3	910.6	651.9	520.7	225.4
B3LYP	aug-cc-pVDZ	3473.4	2367.8	2166.6	905.2	647.8	518.0	224.8
tHCTHhyb	aug-cc-pVTZ	3444.5	2343.9	2150.3	897.3	684.4	534.6	237.1
VSXC	aug-cc-pVTZ	3471.1	2313.4	2133.8	901.8	666.9	538.8	241.6
BMK	aug-cc-pVTZ	3446.0	2430.9	2202.7	891.4	717.8	541.9	239.7
B1B95	aug-cc-pVTZ	3478.7	2405.5	2196.5	908.2	718.3	551.3	241.7
B3P86	aug-cc-pVTZ	3469.0	2383.0	2181.8	909.6	706.8	548.4	241.0
B98	aug-cc-pVTZ	3454.4	2367.2	2169.4	899.1	697.5	538.6	238.9
B971	aug-cc-pVTZ	3452.5	2360.5	2163.8	898.3	695.1	537.6	238.3
B972	aug-cc-pVTZ	3484.0	2384.1	2182.2	907.1	710.5	549.3	241.5
wB97X	aug-cc-pVTZ	3477.3	2452.3	2227.0	895.9	755.7	556.7	242.2
wB97XD	aug-cc-pVTZ	3472.5	2426.2	2209.2	899.5	735.1	550.8	240.7
CAM-B3LYP	aug-cc-pVTZ	3470.4	2438.5	2222.4	903.2	743.1	556.1	243.3
M062X	aug-cc-pVTZ	3475.0	2448.9	2226.0	900.2	748.0	554.6	239.7
SVWN	aug-cc-pVTZ	3378.2	2325.6	2128.7	913.9	661.4	538.2	234.9
B3PW91	aug-cc-pVTZ	3462.9	2377.1	2177.0	906.6	706.1	547.7	240.7
B3LYP	aug-cc-pVTZ	3460.1	2367.1	2173.3	902.7	705.6	545.8	241.4
tHCTHhyb	def2SVPD	3437.8	2367.2	2153.4	904.1	689.3	562.0	259.6
VSXC	def2SVPD	3462.1	2338.0	2141.2	908.5	668.3	551.3	252.7
BMK	def2SVPD	3455.9	2450.1	2188.1	904.6	723.4	578.4	275.5
B1B95	def2SVPD	3472.9	2433.4	2205.4	915.9	724.5	571.8	259.3
B3P86	def2SVPD	3461.3	2407.0	2188.1	915.7	711.7	573.9	261.6
B98	def2SVPD	3449.9	2390.5	2172.9	906.1	702.4	564.9	262.0
B971	def2SVPD	3448.7	2384.6	2168.4	905.5	699.1	561.4	259.6
B972	def2SVPD	3476.7	2411.2	2192.4	913.7	714.8	566.2	256.4
wB97X	def2SVPD	3467.1	2476.2	2234.7	901.4	756.3	569.5	257.1
wB97XD	def2SVPD	3460.6	2447.8	2214.6	904.5	736.8	570.3	259.1
CAM-B3LYP	def2SVPD	3463.9	2462.7	2228.1	909.1	747.5	578.5	266.7
M062X	def2SVPD	3467.9	2476.5	2234.8	905.6	753.9	573.8	261.1
SVWN	def2SVPD	3380.4	2355.2	2137.1	921.7	673.7	587.9	267.5
B3PW91	def2SVPD	3456.7	2402.3	2184.0	913.1	710.7	572.4	260.3
B3LYP	def2SVPD	3452.4	2391.2	2179.3	908.5	710.6	571.1	264.5
tHCTHhyb	def2TZVPD	3447.8	2345.5	2151.0	898.5	681.9	537.3	238.1
VSXC	def2TZVPD	3469.8	2313.9	2134.1	903.2	667.4	539.5	242.0
BMK	def2TZVPD	3473.7	2432.1	2197.6	895.1	712.8	549.7	242.4
B1B95	def2TZVPD	3481.9	2407.5	2198.8	909.5	717.0	552.6	242.2
B3P86	def2TZVPD	3471.0	2385.0	2183.8	910.7	704.5	550.4	242.2
B98	def2TZVPD	3459.5	2368.5	2170.1	900.3	694.3	541.3	240.0
B971	def2TZVPD	3458.0	2361.7	2164.4	899.5	691.9	539.6	239.2
B972	def2TZVPD	3484.6	2385.8	2184.7	908.1	708.7	549.6	241.5
wB97X	def2TZVPD	3477.5	2453.5	2229.2	896.7	754.0	557.5	243.3
wB97XD	def2TZVPD	3470.8	2427.2	2211.2	900.0	732.5	552.4	242.2
CAM-B3LYP	def2TZVPD	3472.7	2440.4	2224.3	904.4	741.4	559.2	245.0
M062X	def2TZVPD	3480.4	2450.6	2228.0	901.0	748.6	556.7	240.6
SVWN	def2TZVPD	3378.9	2330.1	2132.4	915.2	660.7	546.1	238.6
B3PW91	def2TZVPD	3464.7	2379.2	2179.0	907.7	703.6	549.8	241.8
B3LYP	def2TZVPD	3462.3	2369.2	2175.2	903.8	703.5	548.9	242.9
tHCTHhyb	def2QZVPD	3445.1	2342.6	2148.4	897.4	685.8	532.9	235.7
VSXC	def2QZVPD	3468.6	2310.0	2130.4	901.1	663.6	535.0	238.8
BMK	def2QZVPD	3459.2	2430.0	2197.1	893.3	726.9	541.9	238.8
B1B95	def2QZVPD	3478.8	2404.0	2195.2	908.2	717.9	548.6	239.6
B3P86	def2QZVPD	3468.3	2381.6	2180.3	909.4	706.8	546.1	239.4
B98	def2QZVPD	3455.9	2366.5	2168.0	899.3	698.2	537.0	237.7
B971	def2QZVPD	3453.9	2359.6	2162.3	898.4	695.3	535.5	236.8
B972	def2QZVPD	3482.7	2382.8	2181.2	906.9	710.3	546.7	239.4
wB97X	def2QZVPD	3474.2	2450.0	2225.2	895.3	753.5	552.5	239.7
wB97XD	def2QZVPD	3469.3	2424.5	2207.9	898.9	737.5	548.0	239.1
CAM-B3LYP	def2QZVPD	3469.7	2436.9	2220.4	903.1	742.4	553.4	241.6
M062X	def2QZVPD	3479.7	2447.4	2224.7	900.1	750.7	552.1	238.8
SVWN	def2QZVPD	3377.1	2324.7	2127.9	913.7	661.8	536.8	233.4
B3PW91	def2QZVPD	3462.5	2375.9	2175.5	906.5	707.0	545.8	239.2
B3LYP	def2QZVPD	3459.5	2365.8	2171.6	902.6	705.1	543.5	239.8
gas[4]		3327	2271	2077	877	663	500	223

Table S92: Harmonic unscaled vibrational frequencies (in cm^{-1}) for S_1 of HC_5N

method	basis	1	2	3	4	5	6	7	8	9	10	11
tHCTHhyb	aug-cc-pVDZ	3449.1	2219.3	1938.5	1785.8	1275.6	639.2	411.1	319.5	174.8	89.5	-591.5
VSXC	aug-cc-pVDZ	3485.8	2225.2	1968.5	1791.7	1272.6	646.1	399.3	281.6	99.2	82.5	-457.7
BMK	aug-cc-pVDZ	3463.4	2173.3	1907.5	1720.4	1280.8	632.9	424.4	362.8	201.3	90.8	-756.6
B1B95	aug-cc-pVDZ	3481.3	2245.6	1952.0	1798.6	1302.8	650.0	432.4	363.6	230.5	92.5	-519.9
B3P86	aug-cc-pVDZ	3471.1	2248.7	1959.0	1808.2	1296.9	649.2	425.9	350.7	219.2	93.1	-555.4
B98	aug-cc-pVDZ	3459.2	2221.6	1932.4	1785.2	1284.0	641.9	417.4	331.5	191.6	90.6	-604.3
B971	aug-cc-pVDZ	3458.5	2221.6	1931.6	1785.7	1282.6	641.5	416.6	331.1	194.8	90.8	-580.4
B972	aug-cc-pVDZ	3488.4	2246.8	1956.2	1807.4	1297.6	649.4	427.9	348.4	218.6	92.6	-479.0
wB97X	aug-cc-pVDZ	3471.9	2202.5	1963.5	1742.7	1302.9	644.6	438.2	415.5	239.0	88.5	-494.6
wB97XD	aug-cc-pVDZ	3465.0	2226.0	1952.5	1773.5	1301.7	645.7	430.2	387.9	224.6	90.9	-553.7
CAM-B3LYP	aug-cc-pVDZ	3471.6	2223.1	1959.9	1769.6	1304.8	647.6	439.0	399.9	229.8	90.1	-601.5
M062X	aug-cc-pVDZ	3468.0	2065.2	1870.1	1677.3	1301.8	640.4	435.6	395.3	230.8	90.6	-549.4
SVWN	aug-cc-pVDZ	3395.4	2254.2	2000.4	1799.9	1277.3	647.8	410.7	323.3	218.0	92.0	-630.7
B3PW91	aug-cc-pVDZ	3467.5	2243.5	1953.6	1804.0	1294.1	647.7	425.6	348.4	218.8	93.0	-538.0
B3LYP	aug-cc-pVDZ	3462.8	2224.9	1936.6	1793.2	1288.0	644.6	423.6	343.2	209.6	90.6	-597.1
tHCTHhyb	aug-cc-pVTZ	3433.0	2204.2	1934.5	1784.9	1276.5	640.0	447.5	434.7	367.9	259.5	103.0
VSXC	aug-cc-pVTZ	3462.0	2217.7	1984.4	1793.2	1272.1	640.1	825.2	421.2	158.1	-170.2	-408.9
BMK	aug-cc-pVTZ	3422.3	2156.2	1908.5	1717.1	1282.9	634.1	459.4	444.7	414.0	264.2	100.4
B1B95	aug-cc-pVTZ	3465.3	2226.6	1945.7	1790.9	1299.7	648.6	467.3	447.6	423.5	266.0	104.8
B3P86	aug-cc-pVTZ	3457.4	2231.6	1953.9	1803.9	1295.9	649.0	464.4	449.1	405.6	267.6	102.6
B98	aug-cc-pVTZ	3441.6	2206.9	1929.6	1783.8	1284.5	642.5	449.6	439.0	381.0	260.6	100.5
B971	aug-cc-pVTZ	3440.1	2205.4	1927.6	1783.3	1282.4	641.9	447.6	440.3	377.5	259.9	100.2
B972	aug-cc-pVTZ	3473.9	2229.6	1950.6	1801.0	1294.8	648.0	460.4	448.8	403.4	265.2	103.7
wB97X	aug-cc-pVTZ	3461.8	2188.3	1959.5	1735.1	1300.0	643.6	484.9	468.2	448.8	261.4	104.8
wB97XD	aug-cc-pVTZ	3457.1	2210.9	1949.7	1768.2	1300.7	645.6	462.8	452.1	439.4	262.6	104.7
CAM-B3LYP	aug-cc-pVTZ	3457.0	2207.5	1957.5	1763.2	1303.6	647.7	476.6	458.1	455.8	265.4	99.6
M062X	aug-cc-pVTZ	3453.1	2065.1	1871.0	1670.6	1299.1	639.6	474.6	463.2	449.9	264.4	92.3
SVWN	aug-cc-pVTZ	3369.4	2235.8	2011.4	1838.7	1278.4	643.6	455.6	431.2	373.5	263.3	99.0
B3PW91	aug-cc-pVTZ	3451.6	2225.1	1947.8	1798.8	1292.5	647.2	464.7	448.1	403.4	266.8	103.9
B3LYP	aug-cc-pVTZ	3449.6	2208.3	1933.0	1790.0	1288.0	645.1	457.9	446.2	396.4	263.6	102.0
tHCTHhyb	def2SVPD	3428.7	2224.6	1967.7	1792.1	1282.5	642.1	664.5	484.2	380.1	311.9	109.6
VSXC	def2SVPD	3456.9	2226.5	2006.7	1807.8	1276.8	643.5	586.4	465.7	385.0	213.6	142.8
BMK	def2SVPD	3437.0	2180.4	1942.7	1722.6	1289.0	637.6	726.7	515.9	423.5	331.6	116.4
B1B95	def2SVPD	3462.2	2249.6	1983.9	1799.8	1307.8	651.8	650.7	490.6	435.1	309.9	108.1
B3P86	def2SVPD	3451.9	2252.1	1987.3	1811.6	1302.3	651.3	678.4	496.5	416.5	315.4	110.3
B98	def2SVPD	3439.4	2226.6	1963.5	1788.9	1290.4	644.6	660.3	489.8	395.9	314.7	109.9
B971	def2SVPD	3438.7	2225.9	1962.0	1789.5	1288.8	644.1	643.9	483.9	394.4	311.2	108.9
B972	def2SVPD	3468.4	2249.0	1985.1	1809.2	1302.4	651.0	633.3	479.3	414.7	304.4	107.0
wB97X	def2SVPD	3453.2	2209.0	1994.1	1740.9	1305.3	645.5	624.5	497.3	475.7	305.4	105.6
wB97XD	def2SVPD	3446.8	2230.7	1984.3	1772.8	1305.6	647.2	657.1	492.4	454.8	310.5	107.3
CAM-B3LYP	def2SVPD	3452.4	2229.3	1993.0	1769.5	1309.1	649.5	669.7	511.6	463.1	321.8	110.9
M062X	def2SVPD	3450.6	2069.7	1905.2	1672.5	1305.5	641.7	658.3	499.3	465.1	310.7	108.2
SVWN	def2SVPD	3375.9	2259.5	2033.9	1832.6	1282.8	647.8	779.3	533.1	386.6	325.4	117.6
B3PW91	def2SVPD	3447.5	2246.7	1982.1	1807.1	1299.4	649.7	672.4	492.8	413.7	312.9	109.4
B3LYP	def2SVPD	3443.8	2230.0	1967.6	1797.3	1294.2	647.0	663.4	499.9	409.1	319.1	111.3
tHCTHhyb	def2TZVPD	3438.6	2207.2	1937.8	1786.0	1277.3	640.3	446.3	430.6	367.9	258.9	98.6
VSXC	def2TZVPD	3467.6	2207.0	1981.5	1818.0	1285.0	637.8	430.9	389.6	329.9	257.2	78.3
BMK	def2TZVPD	3455.9	2162.9	1910.6	1719.9	1284.0	634.7	455.6	444.2	417.8	258.2	103.5
B1B95	def2TZVPD	3471.1	2229.3	1949.1	1792.0	1301.2	649.3	467.7	447.6	426.1	265.7	103.5
B3P86	def2TZVPD	3461.6	2234.4	1957.5	1804.9	1297.1	649.6	465.5	444.8	408.8	266.0	100.7
B98	def2TZVPD	3449.0	2209.6	1932.6	1784.0	1285.3	642.9	451.5	434.5	384.5	259.9	101.2
B971	def2TZVPD	3447.8	2207.9	1930.5	1783.4	1283.1	642.2	449.1	431.4	381.7	259.7	98.1
B972	def2TZVPD	3476.3	2230.4	1953.4	1801.5	1296.0	648.8	459.9	437.4	403.8	262.9	101.5
wB97X	def2TZVPD	3464.6	2189.4	1962.7	1735.7	1300.7	644.1	484.9	469.5	451.1	259.3	97.4
wB97XD	def2TZVPD	3458.1	2213.0	1953.9	1768.9	1301.5	646.0	464.4	457.2	445.0	258.8	98.3
CAM-B3LYP	def2TZVPD	3461.5	2211.5	1961.5	1765.0	1304.7	648.2	477.2	466.0	457.8	264.4	98.8
M062X	def2TZVPD	3461.7	2068.5	1875.3	1672.0	1300.4	639.8	473.2	471.9	447.7	263.6	106.4
SVWN	def2TZVPD	3368.9	2231.8	1995.6	1809.6	1280.6	645.8	483.2	438.7	373.8	266.9	97.4
B3PW91	def2TZVPD	3455.6	2228.2	1951.5	1799.9	1293.8	647.8	464.2	442.9	406.6	265.1	101.7
B3LYP	def2TZVPD	3453.9	2211.8	1936.9	1791.0	1289.3	645.6	464.5	444.6	400.7	263.8	102.1
tHCTHhyb	def2QZVPD	3435.3	2203.2	1933.6	1783.2	1276.2	640.1	441.8	426.2	351.0	255.6	101.2
VSXC	def2QZVPD	3424.4	2217.0	1998.6	1860.5	1303.2	629.4	1147.8	378.7	299.8	-164.5	-939.1
BMK	def2QZVPD	3441.4	2156.2	1904.9	1716.1	1283.2	634.8	464.4	458.9	445.5	267.6	112.7
B1B95	def2QZVPD	3467.6	2224.8	1944.7	1788.8	1299.7	649.0	460.2	441.1	390.2	259.7	100.9
B3P86	def2QZVPD	3458.6	2230.6	1952.9	1801.4	1295.7	648.8	456.6	436.2	366.5	262.2	97.9
B98	def2QZVPD	3445.1	2205.9	1929.2	1781.0	1284.4	642.7	445.2	428.2	340.0	254.7	93.0
B971	def2QZVPD	3443.5	2204.7	1927.0	1781.3	1282.4	642.0	443.7	425.3	341.3	254.5	88.5
B972	def2QZVPD	3474.1	2227.8	1949.5	1798.7	1294.6	648.1	455.4	438.0	355.2	258.9	97.8
wB97X	def2QZVPD	3460.5	2184.7	1958.7	1733.0	1298.8	642.9	490.7	461.2	441.0	259.5	96.6
wB97XD	def2QZVPD	3456.3	2208.6	1949.8	1767.1	1300.1	644.8	466.8	452.9	430.1	258.1	101.3
CAM-B3LYP	def2QZVPD	3458.2	2207.0	1957.4	1760.5	1302.6	647.6	485.4	467.2	446.2	264.4	88.6
M062X	def2QZVPD	3460.1	2064.8	1873.0	1668.9	1298.8	639.5	468.6	462.0	443.4	262.2	106.3
SVWN	def2QZVPD	3359.6	2212.2	2036.9	1837.1	1289.7	654.1	456.9	432.8	366.6	259.0	101.1
B3PW91	def2QZVPD	3453.3	2224.5	1946.8	1796.5	1292.4	647.1	457.5	436.7	360.1	259.6	95.2
B3LYP	def2QZVPD	3451.1	2208.0	1932.2	1787.9	1287.7	645.1	454.4	435.4	352.0	256.3	94.4
Kr[5]		n/a	2136	n/a	n/a	n/a	n/a	493	n/a	n/a	n/a	n/a

Table S93: Harmonic unscaled vibrational frequencies (in cm^{-1}) for S_2 of HC_5N

method	basis	1	2	3	4	5	6	7	8	9	10	11
tHCTHhyb	aug-cc-pVDZ	3449.3	2231.8	1973.2	1800.8	1272.4	638.9	423.8	375.8	223.4	94.1	-564.1
VSXC	aug-cc-pVDZ	3477.5	2226.7	1985.2	1800.7	1272.3	644.0	462.0	323.5	233.7	-154.9	-324.2
BMK	aug-cc-pVDZ	3467.5	2252.8	1993.4	1794.7	1278.9	636.8	439.5	424.7	227.2	96.9	-731.6
B1B95	aug-cc-pVDZ	3482.1	2278.1	2006.9	1836.8	1300.0	651.1	446.6	427.9	249.4	96.2	-497.1
B3P86	aug-cc-pVDZ	3471.9	2266.5	2001.3	1828.8	1293.7	649.3	440.2	413.3	242.3	97.1	-529.3
B98	aug-cc-pVDZ	3459.9	2240.9	1973.5	1805.5	1280.8	641.9	431.5	394.6	230.4	95.9	-574.1
B971	aug-cc-pVDZ	3459.2	2240.0	1973.1	1806.2	1279.5	641.6	430.0	393.3	231.8	96.2	-550.9
B972	aug-cc-pVDZ	3489.1	2264.6	1998.3	1827.1	1294.4	649.5	442.1	408.4	246.6	96.7	-450.3
wB97X	aug-cc-pVDZ	3473.0	2264.4	2012.7	1793.6	1299.6	646.5	483.0	454.6	255.3	93.5	-452.1
wB97XD	aug-cc-pVDZ	3466.2	2270.4	2001.1	1810.5	1297.9	646.6	454.0	444.1	247.0	93.6	-515.5
CAM-B3LYP	aug-cc-pVDZ	3472.3	2267.6	2005.5	1811.0	1301.9	648.9	464.4	453.4	247.6	97.9	-570.1
M062X	aug-cc-pVDZ	3477.4	2286.3	2003.1	1844.9	1301.0	650.3	484.4	446.7	244.8	98.3	-531.5
SVWN	aug-cc-pVDZ	3393.4	2250.8	2028.7	1821.2	1274.9	644.4	438.2	312.6	259.2	-104.8	-557.8
B3PW91	aug-cc-pVDZ	3468.4	2261.3	1996.7	1825.1	1291.0	647.9	439.6	410.2	243.5	96.4	-511.6
B3LYP	aug-cc-pVDZ	3463.0	2242.4	1976.9	1812.4	1284.9	644.6	437.6	407.2	237.7	95.3	-571.7
tHCTHhyb	aug-cc-pVTZ	3433.0	2216.1	1968.2	1799.0	1272.9	639.6	472.1	447.4	438.8	268.9	106.4
VSXC	aug-cc-pVTZ	3461.5	2215.1	1985.2	1796.9	1269.5	645.8	732.0	422.1	161.2	-191.9	-450.2
BMK	aug-cc-pVTZ	3426.3	2230.5	1987.8	1792.5	1278.9	637.5	490.0	485.3	457.6	274.6	100.2
B3P86	aug-cc-pVTZ	3458.1	2248.7	1994.6	1823.7	1292.7	649.1	493.8	471.1	457.2	276.3	108.1
B98	aug-cc-pVTZ	3441.7	2225.1	1968.8	1803.4	1281.0	642.4	480.4	463.4	449.2	269.5	104.1
B971	aug-cc-pVTZ	3440.3	2223.0	1967.3	1803.0	1279.0	641.9	476.1	462.8	447.2	269.9	108.6
B972	aug-cc-pVTZ	3474.6	2246.7	1991.1	1820.3	1291.6	648.1	489.7	475.9	453.4	273.8	104.7
wB97X	aug-cc-pVTZ	3462.2	2246.1	2005.8	1783.7	1296.5	645.3	542.6	501.9	468.5	273.4	108.5
wB97XD	aug-cc-pVTZ	3457.2	2253.4	1995.7	1804.8	1296.9	646.5	514.0	496.3	463.8	272.4	105.4
CAM-B3LYP	aug-cc-pVTZ	3457.4	2249.3	2000.7	1803.4	1300.7	649.0	523.7	509.0	468.0	276.0	105.3
M062X	aug-cc-pVTZ	3459.7	2264.9	1996.2	1829.8	1296.6	648.5	553.0	497.9	455.8	273.5	101.3
SVWN	aug-cc-pVTZ	3368.7	2228.1	2014.2	1809.8	1273.1	642.9	798.9	567.7	459.5	264.6	81.5
B3PW91	aug-cc-pVTZ	3452.4	2242.4	1989.4	1819.4	1289.3	647.4	491.5	475.3	455.5	273.9	105.1
tHCTHhyb	def2SVPD	3429.0	2237.5	2002.2	1807.7	1279.2	641.9	682.3	497.2	449.6	312.3	110.2
VSXC	def2SVPD	3456.8	2226.8	2006.6	1807.7	1276.9	643.6	586.6	463.0	384.7	213.7	145.3
BMK	def2SVPD	3441.4	2259.9	2028.3	1799.1	1286.9	641.5	748.4	532.8	478.8	333.0	115.1
B3P86	def2SVPD	3452.9	2270.3	2028.9	1833.6	1299.2	651.5	698.1	513.4	474.5	316.2	111.4
B98	def2SVPD	3440.2	2246.3	2004.0	1810.9	1287.2	644.7	679.5	504.1	463.3	315.3	111.2
B971	def2SVPD	3439.6	2245.0	2002.9	1811.7	1285.6	644.3	663.4	498.0	461.6	311.8	110.5
B972	def2SVPD	3469.3	2267.3	2026.1	1830.6	1299.3	651.2	654.9	498.9	472.5	306.2	108.6
wB97X	def2SVPD	3449.8	2269.1	2043.4	1791.9	1302.2	647.5	657.4	549.9	494.8	309.5	108.8
wB97XD	def2SVPD	3448.1	2275.1	2032.8	1811.7	1302.1	648.5	684.8	531.2	493.0	312.8	110.0
CAM-B3LYP	def2SVPD	3453.2	2273.5	2037.5	1812.4	1306.6	651.0	696.3	547.3	505.5	324.0	114.1
M062X	def2SVPD	3461.0	2289.3	2035.7	1845.6	1305.5	651.9	684.1	568.8	496.7	314.0	113.3
SVWN	def2SVPD	3372.6	2261.1	2055.8	1832.9	1282.9	647.5	771.2	528.4	486.0	314.0	129.4
B3PW91	def2SVPD	3448.6	2265.1	2024.3	1829.8	1296.3	650.0	692.0	509.2	473.0	314.3	111.0
B3LYP	def2SVPD	3444.2	2247.8	2007.0	1817.6	1291.1	647.1	682.1	514.4	475.5	320.6	112.2
tHCTHhyb	def2TZVPD	3438.8	2219.4	1971.6	1800.0	1273.9	640.0	470.8	444.0	441.3	267.3	98.9
VSXC	def2TZVPD	3464.6	2210.3	1980.2	1800.8	1272.4	642.0	599.2	387.9	328.5	-86.7	-506.8
BMK	def2TZVPD	3458.2	2238.9	1991.7	1795.3	1280.9	638.4	486.2	480.2	456.5	269.2	103.7
B1B95	def2TZVPD	3471.8	2260.8	2001.5	1829.8	1298.3	650.6	491.7	486.5	453.9	272.8	102.1
B3P86	def2TZVPD	3462.3	2251.9	1998.4	1825.2	1293.9	649.7	491.2	475.4	454.3	272.9	107.8
B98	def2TZVPD	3449.2	2228.2	1972.4	1804.1	1281.9	642.9	477.2	458.3	447.7	269.1	100.1
B971	def2TZVPD	3448.3	2225.9	1970.8	1803.8	1279.9	642.2	474.4	452.8	442.5	267.8	102.5
B972	def2TZVPD	3477.0	2248.1	1994.2	1821.1	1292.9	648.9	483.2	472.7	450.6	272.6	98.4
wB97X	def2TZVPD	3465.3	2247.8	2010.1	1784.7	1297.5	645.9	550.6	503.2	468.1	274.2	108.7
wB97XD	def2TZVPD	3458.5	2255.9	2000.8	1805.8	1297.8	647.0	520.9	494.1	461.3	272.5	106.9
CAM-B3LYP	def2TZVPD	3462.1	2253.6	2004.9	1805.2	1301.9	649.4	534.3	508.6	467.8	275.8	102.6
M062X	def2TZVPD	3467.5	2269.7	1996.7	1834.7	1298.6	649.2	555.3	497.3	461.3	273.5	103.2
SVWN	def2TZVPD	3370.6	2235.6	2023.9	1821.8	1276.2	645.5	503.8	327.4	280.6	24.0	-350.0
B3PW91	def2TZVPD	3456.4	2245.9	1993.2	1820.8	1290.6	648.0	489.1	473.7	454.0	274.2	102.1
B3LYP	def2TZVPD	3454.2	2228.9	1976.0	1809.8	1286.1	645.6	490.5	473.7	454.2	271.8	106.2
tHCTHhyb	def2QZVPD	3435.5	2215.2	1967.3	1797.3	1272.5	639.8	468.0	440.9	434.2	265.0	102.3
VSXC	def2QZVPD	3417.9	2199.3	1981.0	1722.6	1268.7	650.9	1112.7	377.3	299.9	-169.2	-943.7
BMK	def2QZVPD	3444.7	2232.3	1985.2	1792.7	1280.5	638.6	486.8	471.9	451.6	276.0	93.6
B1B95	def2QZVPD	3468.5	2256.5	1996.6	1826.5	1296.8	649.9	481.9	461.4	441.5	269.8	91.1
B3P86	def2QZVPD	3459.3	2247.4	1993.5	1821.9	1292.3	649.2	482.1	456.0	444.1	268.6	102.1
B98	def2QZVPD	3444.9	2224.2	1968.7	1801.8	1280.9	642.7	469.0	444.1	429.1	263.8	87.1
B971	def2QZVPD	3443.6	2222.0	1967.0	1801.3	1278.9	641.9	466.8	439.1	431.2	264.3	96.1
B972	def2QZVPD	3474.8	2244.7	1990.1	1818.2	1291.3	648.4	480.0	466.9	442.0	271.2	99.3
wB97X	def2QZVPD	3461.7	2244.3	2005.4	1780.9	1295.5	645.0	551.4	496.5	462.8	275.5	103.2
wB97XD	def2QZVPD	3456.2	2251.9	1996.2	1802.3	1295.9	646.3	539.8	484.6	456.9	274.5	105.7
CAM-B3LYP	def2QZVPD	3459.6	2248.5	1999.8	1800.7	1300.0	648.7	525.9	501.3	462.1	275.9	99.9
M062X	def2QZVPD	3467.0	2263.1	1995.1	1828.2	1297.7	649.0	550.3	491.2	456.1	268.9	107.3
SVWN	def2QZVPD	3358.3	2202.4	1997.2	1819.5	1282.9	627.1	789.7	566.0	276.5	122.1	-569.3
B3PW91	def2QZVPD	3453.8	2241.6	1988.3	1817.4	1289.0	647.4	481.1	461.5	443.6	269.1	110.2
B3LYP	def2QZVPD	3451.3	2224.3	1971.1	1806.3	1284.2	645.1	481.0	459.9	448.3	269.2	105.5
gas[5]		n/a	n/a	n/a	n/a	n/a	n/a	462	n/a	n/a	n/a	n/a
Kr[5]		n/a	n/a	n/a	n/a	n/a	n/a	462	n/a	n/a	278	121

Table S94: Harmonic unscaled vibrational frequencies (in cm^{-1}) for S_0 of HC_5N

method	basis	1	2	3	4	5	6	7	8	9	10	11
tHCTHhyb	aug-cc-pVDZ	3460.1	2327.7	2256.7	2112.0	1202.4	625.1	597.8	476.0	222.8	102.8	-540.8
VSXC	aug-cc-pVDZ	3486.2	2297.9	2240.2	2101.3	1213.9	630.9	579.0	485.6	249.9	107.0	-354.4
BMK	aug-cc-pVDZ	3481.5	2405.6	2308.2	2140.7	1196.0	621.5	634.6	482.5	204.9	102.4	-736.5
B1B95	aug-cc-pVDZ	3494.3	2399.6	2317.7	2168.6	1215.5	633.4	638.4	495.6	240.8	105.5	-452.3
B3P86	aug-cc-pVDZ	3482.4	2372.7	2296.8	2150.4	1217.4	633.6	627.4	490.8	235.1	104.8	-496.6
B98	aug-cc-pVDZ	3471.5	2353.7	2278.5	2134.1	1202.7	626.2	613.9	480.3	224.8	103.6	-551.4
B971	aug-cc-pVDZ	3470.4	2348.6	2273.9	2129.9	1202.6	626.1	612.0	480.0	227.1	103.7	-525.9
B972	aug-cc-pVDZ	3499.0	2376.0	2300.2	2154.8	1215.0	632.9	629.2	494.1	243.0	105.9	-400.8
wB97X	aug-cc-pVDZ	3488.1	2471.3	2361.2	2210.5	1188.5	622.6	681.0	500.9	240.2	105.4	-387.4
wB97XD	aug-cc-pVDZ	3481.3	2438.3	2336.0	2188.2	1195.0	624.9	658.3	492.8	230.3	104.0	-473.0
CAM-B3LYP	aug-cc-pVDZ	3485.5	2442.9	2346.5	2197.7	1200.4	627.5	666.9	498.6	233.1	104.6	-527.7
M062X	aug-cc-pVDZ	3487.3	2451.0	2354.0	2201.0	1197.9	626.4	665.6	498.2	230.1	103.1	-479.1
SVWN	aug-cc-pVDZ	3401.8	2315.9	2246.0	2090.2	1230.9	636.8	588.5	481.6	229.3	101.4	-591.6
B3PW91	aug-cc-pVDZ	3478.6	2367.6	2292.2	2146.5	1214.0	632.0	626.3	490.9	237.0	105.0	-475.3
B3LYP	aug-cc-pVDZ	3473.2	2354.0	2281.7	2140.7	1206.2	628.3	624.4	487.5	231.4	104.2	-542.3
tHCTHhyb	aug-cc-pVTZ	3443.4	2323.9	2260.0	2122.2	1196.1	624.1	656.9	541.5	492.4	272.5	110.6
VSXC	aug-cc-pVTZ	3470.7	2289.2	2238.0	2103.6	1206.3	628.4	635.4	534.2	498.7	277.7	113.5
BMK	aug-cc-pVTZ	3443.7	2415.8	2324.7	2173.7	1180.7	618.0	695.4	575.2	498.9	277.8	111.4
B1B95	aug-cc-pVTZ	3477.6	2391.6	2315.3	2171.3	1206.9	630.7	695.4	565.9	505.9	278.0	112.5
B3P86	aug-cc-pVTZ	3467.9	2365.9	2296.5	2155.3	1210.5	632.1	681.2	563.4	504.2	277.9	112.2
B98	aug-cc-pVTZ	3453.4	2350.2	2281.5	2143.1	1196.2	625.1	672.1	548.3	496.0	274.6	111.5
B971	aug-cc-pVTZ	3451.5	2343.9	2275.9	2137.7	1195.5	624.6	669.8	545.6	495.3	273.8	111.4
B972	aug-cc-pVTZ	3483.2	2367.4	2297.3	2156.2	1207.2	630.5	685.4	557.8	504.4	277.5	112.5
wB97X	aug-cc-pVTZ	3476.5	2463.5	2361.1	2212.3	1181.5	620.7	740.3	586.0	509.2	278.1	113.2
wB97XD	aug-cc-pVTZ	3471.6	2431.8	2338.0	2192.3	1189.4	623.8	716.6	573.4	504.8	275.5	112.3
CAM-B3LYP	aug-cc-pVTZ	3469.9	2437.3	2347.8	2203.2	1193.6	626.3	724.6	584.6	509.1	280.4	112.9
M062X	aug-cc-pVTZ	3474.0	2445.1	2352.8	2204.4	1191.2	624.8	729.7	579.4	507.8	275.9	111.0
SVWN	aug-cc-pVTZ	3377.0	2302.5	2243.4	2095.9	1222.7	635.0	632.0	553.8	492.6	273.0	108.6
B3PW91	aug-cc-pVTZ	3461.8	2359.8	2290.9	2150.5	1206.6	630.2	680.6	561.6	503.5	277.3	112.0
B3LYP	aug-cc-pVTZ	3459.2	2348.8	2282.7	2147.0	1200.3	627.5	680.1	559.5	502.0	278.1	112.3
tHCTHhyb	def2SVPD	3439.4	2342.5	2269.9	2120.4	1206.8	627.7	696.4	659.3	526.5	309.4	119.6
VSXC	def2SVPD	3464.3	2306.8	2249.0	2105.7	1215.4	631.7	646.4	626.9	515.2	298.8	117.2
BMK	def2SVPD	3441.6	2430.5	2320.5	2153.0	1202.7	625.9	743.4	695.1	542.5	326.8	125.3
B1B95	def2SVPD	3474.7	2413.9	2329.1	2174.9	1218.1	634.8	699.3	690.5	532.2	307.5	119.3
B3P86	def2SVPD	3463.0	2385.1	2308.2	2156.7	1220.4	635.3	711.8	683.2	536.2	312.8	120.6
B98	def2SVPD	3451.5	2368.4	2291.1	2141.5	1207.1	628.7	690.9	673.8	528.8	311.6	120.6
B971	def2SVPD	3450.4	2362.5	2286.5	2137.2	1206.6	628.4	678.4	670.6	525.0	308.2	119.6
B972	def2SVPD	3478.6	2388.8	2311.1	2161.2	1216.7	634.0	687.9	680.6	526.7	302.6	118.4
wB97X	def2SVPD	3469.0	2480.0	2374.5	2214.9	1189.7	623.2	739.2	680.4	527.7	303.1	119.0
wB97XD	def2SVPD	3462.5	2446.9	2349.6	2193.0	1197.4	626.1	717.7	697.9	530.4	305.4	119.9
CAM-B3LYP	def2SVPD	3465.7	2455.8	2360.0	2203.7	1203.7	629.2	724.8	695.6	539.3	318.2	122.5
M062X	def2SVPD	3470.4	2465.9	2365.4	2207.5	1199.5	627.2	735.6	700.2	534.0	308.2	120.3
SVWN	def2SVPD	3381.2	2329.8	2262.3	2100.2	1236.0	639.3	797.1	638.4	556.5	328.7	123.0
B3PW91	def2SVPD	3458.4	2380.1	2303.5	2152.7	1216.7	633.5	709.2	682.2	534.3	310.4	120.0
B3LYP	def2SVPD	3454.0	2368.5	2294.4	2148.1	1210.3	630.4	688.3	678.8	534.6	317.3	121.7
tHCTHhyb	def2TZVPD	3447.7	2325.1	2260.8	2121.7	1198.2	624.9	656.7	542.5	492.4	272.7	110.9
VSXC	def2TZVPD	3470.1	2289.0	2237.7	2102.8	1208.2	629.3	635.1	528.2	492.7	278.0	112.9
BMK	def2TZVPD	3473.3	2415.9	2322.1	2167.2	1187.6	620.6	695.4	569.8	499.0	276.4	111.8
B1B95	def2TZVPD	3481.9	2392.8	2316.4	2172.2	1208.9	631.6	695.0	567.0	505.1	278.2	112.3
B3P86	def2TZVPD	3471.0	2367.3	2297.8	2156.1	1212.4	632.8	681.0	562.5	503.7	277.6	112.2
B98	def2TZVPD	3459.5	2351.0	2281.9	2142.6	1198.3	625.8	670.7	550.0	495.9	274.8	111.6
B971	def2TZVPD	3458.0	2344.4	2276.1	2137.1	1197.6	625.4	668.1	547.0	494.7	273.8	111.4
B972	def2TZVPD	3484.7	2368.4	2298.4	2157.5	1208.7	631.2	684.4	555.6	502.9	277.1	112.4
wB97X	def2TZVPD	3477.9	2463.4	2361.6	2213.2	1182.6	621.2	739.0	585.2	507.8	278.0	113.0
wB97XD	def2TZVPD	3471.2	2432.1	2338.7	2193.4	1190.3	624.2	715.4	573.0	504.0	275.5	112.6
CAM-B3LYP	def2TZVPD	3473.2	2438.4	2349.0	2203.8	1195.5	626.9	724.9	588.4	509.3	280.8	113.0
M062X	def2TZVPD	3480.9	2446.1	2353.9	2205.0	1192.7	625.2	732.6	578.8	506.4	275.4	110.8
SVWN	def2TZVPD	3378.6	2307.3	2247.1	2098.5	1224.8	635.9	636.0	566.9	497.2	275.1	109.7
B3PW91	def2TZVPD	3464.7	2361.4	2292.3	2151.4	1208.4	630.9	680.1	561.6	503.1	277.4	112.1
B3LYP	def2TZVPD	3462.5	2350.3	2284.0	2147.6	1202.2	628.1	680.3	563.1	502.2	278.4	112.5
tHCTHhyb	def2QZVPD	3444.8	2321.8	2257.8	2119.5	1196.6	624.3	658.5	536.0	489.2	270.7	109.9
VSXC	def2QZVPD	3468.9	2284.9	2234.0	2099.4	1206.0	628.1	631.2	529.1	493.5	275.1	111.7
BMK	def2QZVPD	3459.0	2413.7	2319.4	2167.4	1184.4	619.6	707.7	571.6	498.4	278.6	110.7
B1B95	def2QZVPD	3478.8	2389.1	2312.9	2169.0	1207.3	630.9	694.6	560.2	501.9	275.8	111.4
B3P86	def2QZVPD	3468.3	2363.6	2294.2	2152.9	1210.8	632.2	681.6	554.8	499.7	275.6	111.3
B98	def2QZVPD	3455.9	2348.5	2279.7	2140.8	1196.8	625.3	672.9	543.2	492.7	273.0	110.8
B971	def2QZVPD	3453.9	2341.8	2273.8	2135.3	1196.1	624.8	669.8	540.4	491.5	272.0	110.5
B972	def2QZVPD	3482.7	2365.2	2295.3	2154.3	1207.3	630.6	685.2	553.2	501.0	275.4	111.5
wB97X	def2QZVPD	3474.7	2459.6	2358.1	2209.3	1181.1	620.5	737.6	577.6	503.6	275.2	111.7
wB97XD	def2QZVPD	3469.6	2428.8	2335.7	2190.1	1189.0	623.6	718.8	565.0	500.2	273.3	111.6
CAM-B3LYP	def2QZVPD	3470.3	2434.5	2345.2	2200.1	1193.9	626.2	723.5	577.8	504.8	278.5	111.9
M062X	def2QZVPD	3479.9	2442.5	2350.6	2202.0	1191.5	624.8	733.9	573.3	504.0	274.6	110.4
SVWN	def2QZVPD	3376.9	2301.0	2241.8	2094.4	1222.5	635.1	632.8	546.3	489.6	270.7	107.9
B3PW91	def2QZVPD	3462.4	2357.8	2288.8	2148.2	1206.9	630.2	681.7	554.2	499.6	275.3	111.2
B3LYP	def2QZVPD	3459.7	2346.5	2280.4	2144.3	1200.5	627.5	679.4	553.4	498.0	276.2	111.4
Kr[5]		3298.3	2247.2	2184.8	2054.2	n/a	n/a	642.2	500.1	n/a	n/a	n/a

Table S95: Harmonic unscaled vibrational frequencies (in cm^{-1}) for S_1 of HC_7N

method	basis	1	2	3	4	5	6	7	8	9	10	11	12	13	14	15
tHCTHhyb	aug-cc-pVDZ	3451.6	2186.4	2072.6	1960.4	1862.5	1439.7	953.8	490.0	427.6	417.0	203.2	127.8	55.7	-509.1	-1627.4
VSXC	aug-cc-pVDZ	3480.8	2152.9	2086.7	1979.5	1796.8	1397.6	949.2	441.2	423.9	377.4	218.3	133.4	58.5	-384.8	-1258.6
BMK	aug-cc-pVDZ	3467.7	2124.4	2088.7	1872.0	1808.8	1444.5	946.7	486.2	477.3	432.6	194.0	136.9	59.1	-639.9	-2041.1
B1B95	aug-cc-pVDZ	3483.3	2202.3	2119.3	1946.1	1886.0	1467.8	969.1	498.3	473.8	443.9	240.6	136.0	58.3	-436.1	-1509.5
B3P86	aug-cc-pVDZ	3473.1	2212.4	2106.5	1970.7	1889.6	1462.4	967.9	497.5	460.0	437.6	228.2	135.6	57.5	-475.5	-1583.5
B98	aug-cc-pVDZ	3461.7	2184.2	2085.5	1939.9	1868.7	1447.9	957.3	492.0	443.4	427.2	208.3	130.6	56.5	-519.7	-1666.1
B971	aug-cc-pVDZ	3460.9	2184.7	2081.7	1943.0	1868.3	1446.6	956.8	491.8	441.8	426.3	212.3	131.4	57.2	-496.2	-1610.4
B972	aug-cc-pVDZ	3490.1	2208.9	2107.9	1964.0	1889.8	1462.8	967.9	497.8	458.5	442.0	240.5	138.0	57.6	-396.4	-1397.5
wB97X	aug-cc-pVDZ	3474.5	2179.3	2081.1	1929.1	1764.2	1463.8	957.5	495.1	533.3	450.1	244.1	132.5	58.4	-413.9	-1448.1
wB97XD	aug-cc-pVDZ	3468.5	2166.8	2124.3	1933.2	1823.8	1463.9	960.3	495.5	502.9	442.3	228.9	129.0	56.3	-469.7	-1572.2
CAM-B3LYP	aug-cc-pVDZ	3473.7	2171.6	2129.7	1929.7	1828.7	1467.1	963.3	497.0	511.8	447.2	232.7	130.1	56.7	-509.3	-1686.5
M062X	aug-cc-pVDZ	3470.8	2102.5	1865.2	1854.3	1702.8	1469.2	953.8	491.9	506.3	444.0	235.8	131.0	57.6	-470.6	-1565.2
SVWN	aug-cc-pVDZ	3393.9	2221.2	2099.1	2041.1	1859.3	1427.6	967.2	493.8	428.1	420.3	221.9	136.8	54.9	-541.1	-1752.0
B3PW91	aug-cc-pVDZ	3469.6	2206.7	2101.6	1965.0	1885.6	1459.2	965.9	496.4	458.7	437.1	231.6	135.0	56.8	-459.0	-1545.6
B3LYP	aug-cc-pVDZ	3464.6	2187.3	2087.1	1947.4	1875.1	1452.0	960.9	494.0	453.3	433.5	219.5	130.2	56.6	-507.8	-1657.0
tHCTHhyb	aug-cc-pVTZ	3434.4	2168.2	2073.6	1946.8	1862.1	1438.1	953.8	490.9	523.8	489.0	470.2	432.2	291.7	153.7	64.2
VSXC	aug-cc-pVTZ	3464.1	2180.4	2071.9	2009.0	1861.8	1426.6	957.5	493.1	710.9	568.8	340.3	217.4	167.4	-252.4	-490.3
BMK	aug-cc-pVTZ	3426.5	2128.5	2077.7	1881.3	1782.6	1442.1	945.1	487.8	615.8	542.1	491.7	450.4	302.4	151.9	59.3
B1B95	aug-cc-pVTZ	3467.5	2181.7	2116.0	1934.1	1873.7	1462.0	965.7	497.5	537.0	522.6	486.5	445.3	297.4	153.4	62.8
B3P86	aug-cc-pVTZ	3458.9	2192.0	2105.4	1956.6	1884.7	1458.9	966.7	497.7	564.6	519.9	490.2	445.7	300.5	157.6	66.2
B98	aug-cc-pVTZ	3443.5	2167.0	2086.7	1929.6	1865.4	1445.9	957.0	492.8	513.7	503.6	473.2	434.3	292.5	155.8	62.9
B971	aug-cc-pVTZ	3442.0	2166.3	2082.0	1930.9	1864.4	1443.8	956.0	492.3	508.4	497.1	470.5	431.7	290.7	155.7	65.2
B972	aug-cc-pVTZ	3475.0	2189.1	2105.5	1950.0	1882.4	1457.6	965.0	496.9	527.7	514.4	483.4	441.3	296.4	157.6	66.2
wB97X	aug-cc-pVTZ	3463.9	2178.3	2067.7	1925.6	1747.0	1458.0	954.4	494.7	598.1	536.2	493.2	448.7	294.6	152.6	55.8
wB97XD	aug-cc-pVTZ	3459.3	2160.7	2114.8	1930.7	1808.3	1460.2	959.3	495.8	566.7	553.0	491.5	450.4	295.2	150.7	57.8
CAM-B3LYP	aug-cc-pVTZ	3458.8	2167.3	2117.2	1928.8	1810.3	1462.9	962.0	497.3	570.9	549.0	496.0	453.4	295.7	149.8	56.8
M062X	aug-cc-pVTZ	3456.6	2106.9	1883.1	1865.3	1689.8	1462.5	951.1	491.8	584.9	570.8	493.8	453.4	296.4	148.6	58.7
SVWN	aug-cc-pVTZ	3369.3	2197.1	2097.9	2022.2	1868.1	1442.3	963.1	492.9	570.1	484.0	471.2	436.5	294.6	151.7	60.5
B3PW91	aug-cc-pVTZ	3453.0	2185.4	2099.7	1950.1	1879.2	1455.0	963.9	496.4	557.2	519.0	488.0	444.3	299.5	157.3	64.0
B3LYP	aug-cc-pVTZ	3450.8	2167.3	2087.3	1935.0	1870.4	1449.5	960.4	494.7	527.0	512.8	482.0	441.1	295.6	155.5	65.0
tHCTHhyb	def2SVPD	3431.2	2188.3	2101.6	1963.2	1867.5	1443.9	957.8	492.7	957.0	598.5	497.8	479.7	338.8	173.4	65.8
VSXC	def2SVPD	3458.7	2192.3	2102.6	2015.0	1872.0	1440.7	960.3	494.0	873.0	632.2	535.9	463.3	272.0	188.6	73.9
BMK	def2SVPD	3442.0	2154.0	2093.7	1877.6	1810.0	1450.2	952.3	490.6	1061.1	660.7	545.1	506.1	364.1	186.1	69.2
B1B95	def2SVPD	3464.7	2205.1	2147.3	1950.0	1885.5	1470.2	971.2	500.1	920.3	592.0	545.7	485.2	334.8	170.5	65.1
B3P86	def2SVPD	3454.2	2212.7	2133.5	1973.0	1892.0	1465.2	970.9	499.6	979.4	614.7	528.2	490.8	343.7	175.8	66.1
B98	def2SVPD	3442.1	2186.3	2114.2	1943.8	1871.4	1451.5	960.9	494.6	937.5	598.0	512.8	485.1	340.9	174.4	66.1
B971	def2SVPD	3441.3	2186.5	2110.2	1946.5	1870.7	1450.0	960.2	494.2	910.9	586.5	509.9	480.7	336.8	172.7	65.7
B972	def2SVPD	3470.4	2208.2	2135.4	1965.6	1890.4	1464.8	970.0	499.3	902.1	570.4	527.8	475.9	326.9	167.5	64.9
wB97X	def2SVPD	3456.6	2211.2	2080.9	1931.4	1761.6	1464.3	958.4	496.5	897.7	608.0	577.1	484.1	333.3	170.2	65.1
wB97XD	def2SVPD	3450.6	2189.4	2131.2	1936.8	1821.8	1465.5	962.3	497.2	955.0	599.8	568.4	484.9	336.4	172.4	66.0
CAM-B3LYP	def2SVPD	3454.7	2198.2	2135.4	1934.3	1827.0	1469.3	965.9	499.1	936.4	623.8	579.6	502.1	351.6	180.1	66.6
M062X	def2SVPD	3454.8	2138.0	1866.6	1847.7	1697.0	1470.1	955.4	493.6	923.7	604.2	574.4	486.1	336.9	169.1	63.8
SVWN	def2SVPD	3380.6	2229.3	2120.3	2048.1	1874.8	1452.6	970.9	497.8	1202.5	720.2	538.9	478.5	365.5	192.9	68.9
B3PW91	def2SVPD	3449.8	2207.1	2128.8	1967.2	1887.4	1461.8	968.5	498.4	973.6	606.6	526.1	487.3	340.0	173.7	65.9
B3LYP	def2SVPD	3445.7	2189.4	2116.0	1951.7	1878.4	1455.6	964.3	496.5	927.1	614.9	523.3	494.6	349.2	178.7	66.8
tHCTHhyb	def2TZVPD	3440.0	2172.0	2076.2	1950.1	1862.9	1439.2	954.4	490.7	504.7	485.6	466.1	424.5	285.9	147.8	58.9
VSXC	def2TZVPD	3465.8	2178.2	2076.5	2005.9	1866.3	1435.6	956.3	492.4	600.6	516.6	443.7	388.4	290.1	138.1	-207.2
BMK	def2TZVPD	3459.9	2130.0	2083.5	1880.2	1791.6	1444.3	947.3	488.0	534.7	526.6	472.9	429.1	284.8	148.6	56.3
B1B95	def2TZVPD	3472.6	2184.8	2119.1	1936.8	1875.4	1463.6	966.8	497.9	534.8	518.4	484.7	438.0	293.5	151.3	62.8
B3P86	def2TZVPD	3462.9	2195.8	2108.3	1959.6	1885.8	1460.1	967.3	497.7	520.2	515.1	480.9	435.0	291.8	152.3	59.0
B98	def2TZVPD	3450.7	2170.5	2089.0	1932.4	1866.1	1446.8	957.6	492.8	508.6	501.8	470.6	429.6	287.8	149.5	60.5
B971	def2TZVPD	3449.5	2169.6	2084.1	1933.6	1864.7	1444.7	956.6	492.2	504.7	498.4	469.0	426.8	287.1	149.6	59.7
B972	def2TZVPD	3477.2	2190.6	2107.9	1951.9	1882.9	1458.7	965.8	497.3	520.1	487.5	477.1	433.9	291.4	152.0	61.2
wB97X	def2TZVPD	3466.4	2181.2	2068.2	1926.7	1747.9	1458.6	955.4	494.8	600.1	520.0	489.5	441.2	290.0	149.3	60.3
wB97XD	def2TZVPD	3460.2	2163.7	2116.8	1932.3	1810.0	1460.9	959.9	495.8	567.0	522.3	482.5	436.2	287.6	148.0	67.2
CAM-B3LYP	def2TZVPD	3463.2	2170.9	2121.2	1930.7	1814.0	1464.2	962.9	497.4	576.9	554.1	492.4	444.6	292.4	154.8	68.2
M062X	def2TZVPD	3465.0	2110.9	1886.6	1867.2	1692.8	1464.1	952.5	492.1	579.7	551.1	487.6	439.3	291.5	153.5	64.4
SVWN	def2TZVPD	3372.5	2209.3	2097.0	2029.0	1878.5	1443.8	965.6	493.4	572.2	480.6	473.2	426.2	289.8	150.1	60.2
B3PW91	def2TZVPD	3456.7	2189.4	2102.8	1953.3	1880.7	1456.3	964.6	496.3	521.7	515.5	480.0	434.7	291.5	152.3	57.9
B3LYP	def2TZVPD	3455.2	2171.9	2090.6	1938.6	1871.9	1450.7	961.1	494.7	533.9	511.7	478.8	435.0	290.5	150.9	58.5
tHCTHhyb	def2QZVPD	3436.8	2167.2	2072.9	1944.5	1860.4	1438.0	953.6	491.0	484.0	467.9	455.0	418.5	284.9	150.4	59.5
VSXC	def2QZVPD	3464.2	2155.1	2084.1	2000.7	1851.1	1462.2	942.8	467.8	997.4	607.1	330.1	186.0	97.6	-605.6	-751.1
B1B95	def2QZVPD	3469.4	2180.6	2115.4	1931.6	1871.1	1461.5	965.9	497.6	511.0	488.3	473.5	431.5	291.6	149.1	46.0
B3P86	def2QZVPD	3459.9	2191.4	2104.6	1953.9	1882.1	1458.2	966.5	497.6	513.8	484.5	471.6	432.3	291.5	156.6	61.1
B971	def2QZVPD	3445.2	2165.2	2081.3	1928.3	1861.7	1443.3	956.1	492.4	485.0	468.4	454.9	420.8	285.4	151.2	53.9
B972	def2QZVPD	3474.9	2187.4	2104.7	1947.1	1879.1	1456.6	964.9	497.2	509.8	483.3	468.7	430.2	290.8	155.5	60.0
wB97X	def2QZVPD	3462.8	2177.6	2065.5	1922.4	1744.5	1456.8	953.9	494.2	600.5	491.1	475.3	437.4	286.8	143.6	57.4
wB97XD	def2QZVPD	3457.7	2160.1	2112.9	1928.8	1805.0	1459.4	958.6	495.2	569.4	484.0	468.6	436.2	284.2	147.4	46.2
CAM-B3LYP	def2QZVPD	3459.7	2167.0	2116.8	1926.3	1808.7	1462.1	961.3	497.1	581.5	498.8	484.4	439.0	290.7	160.7	56.1
M062X	def2QZVPD	3463.0	2108.3	1883.3	1864.6	1690.6	1463.1	951.8	491.9	585.8	492.2	477.9	430.9	289.3	151.1	61.6
SVWN	def2QZVPD	3362.9	2188.7	2086.7	2010.5	1883.1	1452.7	961.7	491.0	483.8	470.7	467.7	425.0	286.0	149.3	60.3
B3PW91	def2QZVPD	3454.2	2185.4	2099.0	1947.4	1877.0	1454.3	963.8	496.3	514.8	483.5	472.4	431.6	291.0	152.8	62.6
B3LYP	def2QZVPD	3452.0	2167.3	2086.7	1933.0	1868.3	1448.9	960.4	494.6	505.4	482.2	470.7	431.5	289.4	152.2	63.8

Table S96: Harmonic unscaled vibrational frequencies (in cm^{-1}) for S_2 of HC_7N

method	basis	1	2	3	4	5	6	7	8	9	10	11	12	13	14	15
tHCTHhyb	aug-cc-pVDZ	3451.9	2200.4	2088.4	1989.9	1872.9	1436.1	953.8	489.8	455.1	431.4	217.2	134.8	56.7	-494.7	-1619.2
VSXC	aug-cc-pVDZ	3479.1	2192.6	2076.3	2021.2	1870.1	1440.2	959.9	493.5	536.7	439.6	190.7	123.2	50.2	-564.4	-1141.8
BMK	aug-cc-pVDZ	3470.4	2218.2	2143.2	1951.0	1882.8	1439.3	951.4	488.2	506.3	442.0	206.5	141.4	58.6	-628.0	-2038.2
B1B95	aug-cc-pVDZ	3484.2	2242.0	2142.5	2002.9	1914.4	1463.6	970.8	498.9	501.2	453.4	247.0	142.7	57.4	-423.3	-1510.0
B3P86	aug-cc-pVDZ	3473.8	2232.8	2125.0	2009.2	1903.8	1458.8	968.3	497.6	489.8	447.3	237.7	140.7	56.6	-460.6	-1578.1
B98	aug-cc-pVDZ	3462.4	2206.5	2103.3	1977.3	1883.7	1444.1	957.4	492.1	473.8	437.8	222.4	138.5	56.4	-502.1	-1654.7
B971	aug-cc-pVDZ	3461.5	2206.2	2099.7	1981.0	1882.5	1442.8	957.0	491.8	471.7	436.8	226.1	138.0	57.1	-479.9	-1600.5
B972	aug-cc-pVDZ	3490.7	2229.3	2126.3	2000.6	1904.6	1458.9	968.2	497.9	488.6	452.3	250.9	143.4	57.3	-379.4	-1395.1
wB97X	aug-cc-pVDZ	3475.6	2221.5	2163.8	1961.0	1842.6	1458.0	959.7	495.9	562.3	461.5	250.1	139.9	57.4	-388.9	-1442.3
wB97XD	aug-cc-pVDZ	3469.4	2224.0	2157.2	1963.6	1880.5	1458.6	961.5	495.7	532.8	453.2	237.7	142.4	57.0	-449.8	-1558.7
CAM-B3LYP	aug-cc-pVDZ	3474.5	2221.7	2162.3	1961.8	1885.2	1462.7	964.9	497.5	543.6	458.7	240.7	141.6	57.5	-488.5	-1679.0
M062X	aug-cc-pVDZ	3477.4	2250.2	2172.1	1986.0	1912.0	1459.6	965.9	497.6	554.6	456.6	238.4	142.1	58.6	-456.8	-1581.8
SVWN	aug-cc-pVDZ	3395.6	2233.3	2098.3	2059.4	1863.7	1445.8	965.8	493.9	516.5	415.7	288.7	58.9	-81.5	-497.9	-1721.7
B3PW91	aug-cc-pVDZ	3470.3	2227.5	2120.4	2004.1	1900.0	1455.4	966.2	496.6	487.5	447.7	239.9	142.0	56.6	-444.1	-1540.9
B3LYP	aug-cc-pVDZ	3464.9	2207.4	2104.5	1983.1	1890.1	1448.4	961.1	494.1	484.8	443.1	231.2	139.8	57.1	-493.3	-1650.4
tHCTHhyb	aug-cc-pVTZ	3434.6	2181.6	2088.5	1974.3	1872.3	1434.3	953.4	490.7	542.9	520.3	483.7	441.9	296.7	152.7	60.3
VSXC	aug-cc-pVTZ	3465.9	2174.5	2073.9	2007.1	1868.7	1431.5	950.7	485.2	714.0	543.5	339.9	221.0	160.7	-303.8	-503.7
BMK	aug-cc-pVTZ	3429.2	2197.6	2144.7	1938.6	1873.3	1435.5	949.2	489.3	642.9	570.9	509.1	459.4	307.6	155.5	63.7
B1B95	aug-cc-pVTZ	3468.1	2219.4	2138.3	1984.3	1905.9	1458.0	967.4	498.1	561.9	543.6	500.7	454.5	302.0	158.8	63.0
B3P86	aug-cc-pVTZ	3459.4	2211.7	2122.9	1991.9	1899.5	1455.1	967.0	497.7	582.8	546.4	505.0	455.2	302.7	159.0	62.1
B98	aug-cc-pVTZ	3443.8	2188.2	2103.5	1963.4	1881.6	1441.9	957.0	492.8	541.8	530.1	488.3	447.0	297.5	157.4	62.3
B971	aug-cc-pVTZ	3442.4	2186.7	2098.9	1965.7	1879.9	1439.9	956.1	492.4	536.2	520.7	485.7	444.6	295.7	156.7	62.0
B972	aug-cc-pVTZ	3475.5	2209.0	2123.2	1983.6	1897.7	1453.9	965.3	497.0	550.4	534.8	496.3	449.6	299.7	157.7	61.4
wB97X	aug-cc-pVTZ	3464.2	2211.9	2149.2	1954.7	1821.3	1452.7	956.9	495.4	626.2	577.3	513.1	461.2	302.7	154.3	60.3
wB97XD	aug-cc-pVTZ	3459.7	2207.6	2152.8	1957.0	1865.5	1455.1	960.5	496.2	595.3	584.0	507.0	456.2	300.6	156.1	59.5
CAM-B3LYP	aug-cc-pVTZ	3459.4	2205.7	2155.9	1956.9	1866.3	1458.9	963.6	497.9	600.5	578.2	514.1	460.7	303.1	154.8	59.3
M062X	aug-cc-pVTZ	3461.4	2227.3	2166.9	1972.8	1890.2	1452.8	961.8	496.7	618.6	593.7	513.9	459.4	301.3	156.8	61.7
SVWN	aug-cc-pVTZ	3368.8	2198.0	2099.8	2040.8	1875.7	1439.8	959.5	491.2	514.2	416.4	345.2	273.1	246.4	-119.3	-324.3
B3PW91	aug-cc-pVTZ	3453.5	2205.3	2117.7	1986.1	1895.2	1451.2	964.4	496.4	574.5	545.8	502.8	452.5	302.1	158.3	62.0
B3LYP	aug-cc-pVTZ	3451.1	2186.6	2104.0	1967.7	1885.7	1445.9	960.6	494.7	550.4	541.6	497.8	450.0	299.6	159.1	62.0
tHCTHhyb	def2SVPD	3431.3	2202.8	2117.8	1991.7	1878.6	1440.3	957.6	492.6	963.4	611.0	530.3	485.0	339.6	175.7	65.8
VSXC	def2SVPD	3458.8	2192.4	2102.5	2014.9	1871.8	1440.6	960.4	494.0	873.7	633.0	537.4	457.4	275.6	189.9	72.2
BMK	def2SVPD	3444.8	2227.5	2170.0	1953.6	1886.7	1444.9	957.2	492.6	1069.7	676.8	574.1	511.0	365.3	188.8	69.4
B1B95	def2SVPD	3465.5	2244.3	2171.0	2005.2	1916.6	1466.3	973.2	500.8	931.4	608.2	572.5	490.0	336.8	172.9	65.4
B3P86	def2SVPD	3455.0	2233.6	2152.3	2010.1	1907.6	1461.6	971.3	499.8	987.4	628.3	557.9	496.4	344.9	177.7	66.5
B98	def2SVPD	3442.8	2209.0	2132.6	1980.1	1887.9	1447.8	961.2	494.7	945.4	611.7	545.9	489.1	342.1	177.5	65.5
B971	def2SVPD	3442.0	2208.3	2128.7	1983.4	1886.7	1446.3	960.6	494.4	918.9	600.1	542.9	485.3	338.2	175.0	64.6
B972	def2SVPD	3471.1	2229.2	2154.1	2000.6	1906.7	1461.2	970.3	499.5	912.1	586.1	558.1	480.9	329.2	170.6	64.8
wB97X	def2SVPD	3457.5	2244.0	2170.0	1963.2	1839.0	1459.1	960.9	497.3	915.9	638.9	597.3	491.0	336.0	173.4	67.6
wB97XD	def2SVPD	3451.3	2232.7	2178.0	1967.4	1879.1	1460.7	963.9	497.8	968.1	622.2	595.4	494.4	339.3	175.5	65.2
CAM-B3LYP	def2SVPD	3455.5	2234.6	2181.6	1966.6	1883.6	1465.5	967.8	499.8	949.7	643.6	608.9	508.9	354.1	182.4	68.3
M062X	def2SVPD	3461.9	2253.7	2198.1	1990.0	1907.9	1461.4	967.8	499.3	937.4	641.1	605.6	492.2	339.3	174.9	66.6
SVWN	def2SVPD	3374.5	2232.9	2137.2	2067.9	1886.5	1448.9	968.3	496.9	1171.6	712.7	535.3	518.4	306.3	187.0	-62.4
B3PW91	def2SVPD	3450.4	2228.2	2147.8	2005.0	1903.5	1458.2	969.0	498.6	981.6	620.4	556.1	492.7	341.4	176.5	64.8
B3LYP	def2SVPD	3446.1	2209.8	2133.8	1986.2	1893.5	1452.0	964.6	496.6	934.8	628.1	555.6	500.0	350.2	181.0	65.8
tHCTHhyb	def2TZVPD	3440.2	2185.7	2091.3	1978.1	1873.4	1435.5	954.1	490.5	522.8	519.0	479.9	432.4	291.1	155.1	57.6
VSXC	def2TZVPD	3465.0	2177.2	2076.7	2007.1	1865.1	1435.5	956.8	491.9	609.0	513.7	449.0	394.3	273.7	125.1	-190.6
BMK	def2TZVPD	3461.6	2205.2	2148.2	1943.0	1878.4	1438.5	951.8	489.8	562.3	549.2	488.5	437.2	290.5	149.9	61.4
B1B95	def2TZVPD	3473.4	2222.7	2141.5	1987.5	1907.3	1459.6	968.5	498.5	563.2	537.1	499.1	445.6	298.4	156.7	61.2
B3P86	def2TZVPD	3463.6	2215.7	2126.1	1995.5	1901.1	1456.4	967.6	497.8	548.4	538.7	495.0	441.0	296.8	155.4	61.4
B98	def2TZVPD	3451.1	2191.9	2106.2	1967.1	1882.5	1443.0	957.7	492.8	534.8	528.3	485.5	437.7	293.5	154.4	61.8
B971	def2TZVPD	3449.9	2190.4	2101.4	1969.2	1880.5	1440.8	956.7	492.3	532.3	522.2	483.3	436.1	292.6	154.9	61.3
B972	def2TZVPD	3477.8	2210.6	2125.8	1985.8	1898.6	1455.1	966.1	497.4	550.3	508.4	492.4	441.8	297.0	157.2	61.9
wB97X	def2TZVPD	3467.1	2214.7	2150.5	1956.3	1823.1	1453.4	957.7	495.5	628.4	553.7	509.0	452.3	298.9	151.9	62.6
wB97XD	def2TZVPD	3460.6	2210.5	2155.9	1959.8	1867.2	1455.9	961.2	496.2	598.8	552.2	501.8	447.4	296.4	155.3	64.3
CAM-B3LYP	def2TZVPD	3463.9	2210.3	2159.9	1959.5	1869.8	1460.2	964.6	497.9	607.8	581.5	510.6	455.4	300.4	155.3	63.4
M062X	def2TZVPD	3469.2	2233.1	2169.2	1978.1	1893.6	1454.9	963.3	497.0	623.2	572.0	504.5	450.7	296.8	155.6	67.9
SVWN	def2TZVPD	3372.4	2205.8	2105.2	2051.0	1879.4	1441.0	964.0	494.1	653.7	491.2	459.5	347.7	241.8	59.6	-91.2
B3PW91	def2TZVPD	3457.4	2209.5	2120.9	1989.9	1896.5	1452.5	965.0	496.4	547.7	539.9	494.6	442.9	297.2	157.0	63.1
B3LYP	def2TZVPD	3455.6	2191.3	2107.5	1971.8	1887.0	1447.1	961.2	494.8	554.1	544.3	493.8	443.2	296.5	156.7	62.4
tHCTHhyb	def2QZVPD	3436.9	2181.1	2088.1	1972.8	1871.4	1434.0	953.4	490.5	537.8	484.1	471.7	426.9	291.5	155.6	61.2
B1B95	def2QZVPD	3469.9	2218.6	2137.8	1982.0	1904.1	1457.9	967.5	498.2	543.0	503.1	488.3	440.5	295.9	151.8	61.0
B3P86	def2QZVPD	3460.6	2211.2	2122.4	1989.8	1897.7	1454.5	966.7	497.7	541.0	501.5	486.5	435.6	295.7	156.3	65.5
B98	def2QZVPD	3447.0	2187.8	2103.6	1961.5	1879.7	1441.9	957.0	492.9	536.4	489.9	478.2	433.3	291.5	149.8	65.2
B971	def2QZVPD	3445.5	2186.4	2098.9	1964.2	1877.8	1439.6	956.1	492.3	532.1	487.7	475.0	430.1	290.6	153.2	62.8
B972	def2QZVPD	3475.3	2207.0	2122.5	1981.6	1895.5	1453.4	965.2	497.2	533.3	499.4	485.7	437.0	294.0	152.5	63.3
wB97X	def2QZVPD	3463.2	2210.7	2146.9	1952.0	1818.5	1451.7	956.5	495.2	651.2	525.3	502.6	452.3	296.8	153.2	60.6
wB97XD	def2QZVPD	3458.3	2206.4	2152.7	1955.0	1863.1	1454.3	960.2	496.0	610.8	510.8	490.3	443.7	294.7	157.7	58.2
CAM-B3LYP	def2QZVPD	3460.5	2205.3	2155.4	1953.8	1864.0	1458.4	963.3	497.6	599.5	527.1	503.0	449.8	297.7	156.9	63.2
M062X	def2QZVPD	3467.8	2226.9	2166.6	1971.4	1888.1	1453.8	962.2	496.8	611.3	514.8	499.4	435.5	292.6	144.1	63.7
SVWN	def2QZVPD	3423.5	2210.4	2085.8	1982.2	1871.7	1433.0	949.6	494.6	870.1	601.9	393.6	286.9	100.5	-111.6	-1075.3
B3LYP	def2QZVPD	3452.2	2186.7	2103.5	1965.7	1883.7	1445.2	960.2	494.7	561.1	501.2	486.4	437.8	296.9	158.9	67.0

Table S97: Harmonic unscaled vibrational frequencies (in cm^{-1}) for S_0 of HC_7N

method	basis	1	2	3	4	5	6	7	8	9	10	11	12	13	14	15
tHCTHhyb	aug-cc-pVDZ	3460.2	2311.3	2261.5	2207.5	2095.3	1346.6	933.0	479.4	591.7	465.0	195.9	151.8	55.8	-468.2	-1655.8
VSXC	aug-cc-pVDZ	3486.1	2284.9	2233.3	2188.3	2081.9	1362.6	941.9	484.4	569.8	478.0	250.0	158.4	60.3	-268.4	-1275.9
BMK	aug-cc-pVDZ	3481.6	2392.4	2321.3	2254.9	2125.4	1336.0	927.3	475.6	633.3	471.0	164.4	140.0	50.2	-619.4	-2093.7
B1B95	aug-cc-pVDZ	3494.3	2382.3	2330.6	2268.4	2153.8	1357.1	944.3	485.1	633.8	485.0	228.0	157.3	58.5	-380.2	-1533.4
B3P86	aug-cc-pVDZ	3482.5	2355.5	2305.2	2247.6	2134.5	1361.3	945.1	485.6	622.4	479.5	216.7	155.6	57.6	-424.7	-1606.7
B98	aug-cc-pVDZ	3471.6	2336.9	2286.4	2230.1	2118.6	1344.6	934.0	479.9	609.1	469.3	198.2	153.6	56.3	-473.4	-1689.7
B971	aug-cc-pVDZ	3470.4	2331.6	2282.5	2225.5	2114.5	1344.9	933.8	479.9	607.2	469.3	204.7	153.9	56.8	-447.6	-1634.8
B972	aug-cc-pVDZ	3499.0	2359.2	2307.9	2251.3	2139.2	1358.2	943.9	485.2	622.9	484.4	233.3	157.3	59.2	-340.5	-1420.9
wB97X	aug-cc-pVDZ	3488.6	2456.8	2414.1	2313.6	2203.0	1317.6	925.2	475.7	679.4	491.7	229.9	157.7	59.6	-323.2	-1444.0
wB97XD	aug-cc-pVDZ	3481.7	2421.2	2383.2	2289.1	2179.7	1327.6	929.6	477.8	655.3	482.5	210.1	155.0	57.9	-410.5	-1575.7
CAM-B3LYP	aug-cc-pVDZ	3486.0	2425.5	2382.0	2298.9	2187.4	1334.2	933.7	479.7	666.8	487.9	214.2	156.7	57.0	-436.9	-1685.7
M062X	aug-cc-pVDZ	3487.8	2435.4	2379.4	2304.7	2189.3	1330.4	932.3	478.8	662.5	487.4	208.8	154.8	56.6	-416.5	-1591.7
SVWN	aug-cc-pVDZ	3402.0	2298.9	2254.0	2192.1	2070.0	1382.6	952.0	487.9	585.3	469.2	212.6	151.4	54.9	-489.3	-1784.6
B3PW91	aug-cc-pVDZ	3478.7	2350.6	2300.2	2243.2	2130.7	1357.5	942.7	484.3	620.6	479.8	219.9	155.8	57.9	-409.9	-1568.4
B3LYP	aug-cc-pVDZ	3473.4	2337.5	2287.0	2233.8	2125.1	1348.3	936.9	481.5	621.6	476.4	212.2	155.2	56.9	-449.1	-1675.4
tHCTHhyb	aug-cc-pVTZ	3443.5	2311.8	2251.5	2212.3	2104.3	1337.6	930.3	479.0	645.6	595.2	527.0	470.6	294.6	166.6	63.4
VSXC	aug-cc-pVTZ	3471.0	2280.2	2221.3	2186.8	2083.7	1353.2	937.5	482.8	620.7	542.6	517.5	473.3	298.2	170.0	65.0
BMK	aug-cc-pVTZ	3443.7	2404.7	2326.8	2275.2	2158.0	1313.3	919.5	473.3	702.1	686.4	557.8	481.0	304.0	168.7	63.8
B1B95	aug-cc-pVTZ	3477.9	2378.0	2317.6	2267.4	2155.8	1346.1	939.2	483.5	685.2	603.2	543.4	480.5	297.7	168.8	63.9
B3P86	aug-cc-pVTZ	3468.1	2352.8	2292.9	2248.6	2138.5	1352.1	941.7	484.8	670.9	634.9	546.4	481.7	299.7	168.8	63.9
B98	aug-cc-pVTZ	3453.7	2337.5	2277.8	2234.6	2126.7	1335.6	931.1	479.5	661.4	590.0	530.6	472.8	295.8	167.7	63.8
B971	aug-cc-pVTZ	3451.8	2330.9	2272.8	2229.0	2121.4	1335.4	930.5	479.2	658.9	579.9	527.5	471.7	294.6	167.4	63.7
B972	aug-cc-pVTZ	3483.5	2354.5	2294.1	2249.7	2139.7	1348.2	939.3	483.5	674.9	592.1	538.4	479.1	297.1	168.8	64.0
wB97X	aug-cc-pVTZ	3477.1	2450.6	2405.9	2314.1	2204.2	1308.5	921.6	474.7	733.1	642.6	559.8	486.9	300.1	170.5	64.5
wB97XD	aug-cc-pVTZ	3472.2	2417.1	2375.1	2291.6	2182.9	1320.2	927.0	477.4	709.1	644.9	553.3	483.5	297.8	169.2	64.1
CAM-B3LYP	aug-cc-pVTZ	3470.6	2423.0	2373.0	2301.0	2192.1	1324.7	930.6	479.2	716.5	635.0	557.6	483.9	300.4	169.7	64.0
M062X	aug-cc-pVTZ	3474.5	2431.9	2371.5	2305.2	2192.2	1323.0	928.7	478.3	722.2	652.3	558.1	486.5	299.5	168.6	62.5
SVWN	aug-cc-pVTZ	3377.0	2291.0	2233.6	2190.8	2074.2	1371.9	948.1	487.2	633.2	619.0	536.7	470.4	295.6	163.9	61.9
B3PW91	aug-cc-pVTZ	3462.1	2346.9	2286.8	2243.2	2133.8	1347.7	938.8	483.3	670.3	627.2	544.5	480.6	298.9	168.5	63.8
B3LYP	aug-cc-pVTZ	3459.6	2336.5	2275.7	2236.2	2130.4	1339.8	934.4	481.2	669.0	602.8	539.0	477.2	298.1	168.4	63.9
tHCTHhyb	def2SVPD	3439.7	2331.5	2256.9	2219.6	2100.2	1348.9	937.1	481.8	947.7	650.2	624.9	505.2	331.4	185.9	67.6
VSXC	def2SVPD	3464.6	2298.0	2225.2	2197.5	2082.6	1362.5	943.4	485.2	841.4	623.4	574.7	492.1	314.7	179.6	66.6
BMK	def2SVPD	3457.6	2421.0	2319.3	2266.7	2134.9	1338.2	933.9	479.7	1030.0	696.0	671.6	524.1	354.4	197.3	69.6
B1B95	def2SVPD	3475.3	2402.2	2324.9	2278.4	2157.3	1358.1	946.8	486.7	916.2	691.9	622.8	509.3	327.9	184.7	67.5
B3P86	def2SVPD	3463.4	2373.5	2299.4	2257.7	2137.7	1362.6	948.1	487.4	970.2	675.9	641.1	515.1	336.7	187.8	68.3
B98	def2SVPD	3452.0	2357.2	2281.5	2241.3	2122.8	1347.1	938.0	482.3	926.0	665.3	625.2	507.6	333.2	187.3	68.2
B971	def2SVPD	3450.9	2351.1	2277.4	2236.7	2118.4	1347.1	937.5	482.1	902.7	662.1	615.6	503.9	329.2	185.5	67.6
B972	def2SVPD	3479.1	2377.4	2301.0	2261.2	2142.6	1358.1	945.7	486.3	904.0	679.1	605.4	502.6	320.2	182.0	67.0
wB97X	def2SVPD	3470.1	2468.9	2411.4	2323.2	2204.9	1317.2	926.8	476.6	890.8	734.3	623.7	507.9	325.9	183.8	67.8
wB97XD	def2SVPD	3463.4	2434.5	2379.2	2299.3	2181.8	1328.6	931.9	479.3	939.9	710.9	628.8	509.1	326.6	184.9	68.4
CAM-B3LYP	def2SVPD	3466.6	2444.2	2377.8	2309.3	2190.8	1335.6	937.0	481.7	907.6	718.4	648.8	520.7	344.0	191.9	69.3
M062X	def2SVPD	3471.5	2455.6	2374.2	2314.2	2193.0	1331.7	933.9	480.2	912.0	729.8	627.3	509.8	327.7	185.8	67.6
SVWN	def2SVPD	3380.8	2318.3	2253.1	2207.1	2076.6	1386.5	956.7	490.9	1189.7	736.6	627.3	542.1	364.5	198.7	70.3
B3PW91	def2SVPD	3458.8	2368.7	2294.2	2253.3	2133.9	1358.5	945.3	486.1	966.5	674.8	634.8	512.5	333.0	186.4	68.0
B3LYP	def2SVPD	3454.3	2357.7	2282.4	2245.3	2129.3	1350.5	940.8	483.8	907.7	672.2	639.2	516.3	342.9	190.7	69.0
tHCTHhyb	def2TZVPD	3447.9	2312.9	2252.4	2212.8	2103.4	1340.1	931.7	479.5	646.3	595.1	522.1	464.2	290.4	166.0	63.6
VSXC	def2TZVPD	3470.4	2280.3	2220.5	2186.7	2082.3	1355.4	939.0	483.3	622.1	527.7	516.3	468.1	297.3	169.1	64.8
BMK	def2TZVPD	3473.7	2404.6	2324.2	2270.8	2150.4	1321.3	923.8	475.1	687.6	630.3	533.6	466.5	292.1	167.2	64.0
B1B95	def2TZVPD	3482.3	2379.5	2317.7	2268.5	2156.2	1348.3	940.8	484.1	686.4	614.2	542.5	476.3	296.5	168.8	64.1
B3P86	def2TZVPD	3471.3	2354.3	2293.6	2249.7	2138.8	1354.0	943.0	485.2	671.4	613.9	537.5	473.6	295.5	168.5	64.2
B98	def2TZVPD	3459.8	2338.2	2278.0	2234.7	2125.7	1337.9	932.4	479.9	661.0	601.6	528.1	468.0	292.8	167.3	64.0
B971	def2TZVPD	3458.3	2331.5	2272.7	2228.9	2120.2	1337.6	931.8	479.6	658.3	595.9	526.1	466.9	291.9	166.9	63.8
B972	def2TZVPD	3485.0	2355.7	2294.1	2250.9	2140.6	1349.8	940.5	484.0	674.9	578.5	536.0	473.6	295.3	168.5	64.2
wB97X	def2TZVPD	3478.5	2450.6	2404.6	2314.4	2204.6	1309.4	922.3	474.8	733.6	625.0	553.8	477.4	295.0	169.3	64.5
wB97XD	def2TZVPD	3471.8	2417.6	2374.6	2292.2	2183.6	1320.9	927.7	477.6	709.4	621.7	544.5	473.6	291.6	168.3	64.4
CAM-B3LYP	def2TZVPD	3473.9	2424.2	2373.2	2301.9	2192.3	1326.6	931.9	479.6	718.9	656.4	554.8	479.5	298.6	170.0	64.4
M062X	def2TZVPD	3481.5	2433.2	2371.7	2305.9	2192.4	1324.7	929.6	478.6	726.6	638.0	549.9	477.1	293.2	167.3	62.3
SVWN	def2TZVPD	3378.8	2295.3	2238.7	2194.2	2076.3	1373.7	949.6	487.7	697.3	623.9	533.3	468.6	294.3	165.3	63.1
B3PW91	def2TZVPD	3465.0	2348.6	2287.7	2244.5	2134.2	1349.6	940.1	483.7	670.7	616.3	537.4	473.6	295.3	168.4	64.2
B3LYP	def2TZVPD	3462.9	2338.1	2276.4	2237.3	2130.5	1341.8	935.7	481.6	670.8	631.4	536.6	473.0	296.2	168.7	64.4
tHCTHhyb	def2QZVPD	3445.0	2310.2	2248.0	2210.2	2101.4	1338.3	930.5	479.0	647.5	533.5	515.8	459.1	288.5	164.4	62.9
B1B95	def2QZVPD	3479.1	2376.2	2313.4	2265.3	2153.2	1346.7	939.6	483.6	685.3	561.7	535.9	471.4	293.9	167.2	63.5
B3P86	def2QZVPD	3468.6	2351.1	2289.1	2246.5	2135.8	1352.4	941.8	484.8	671.4	556.2	531.8	469.3	293.8	167.0	63.6
B98	def2QZVPD	3456.2	2336.3	2274.4	2232.8	2124.2	1336.4	931.4	479.6	662.8	544.9	522.3	463.1	290.8	165.9	63.4
B971	def2QZVPD	3454.2	2329.5	2269.0	2227.0	2118.6	1336.1	930.8	479.3	659.6	541.6	520.1	462.0	289.7	165.5	63.2
B972	def2QZVPD	3483.0	2352.9	2290.5	2247.9	2137.7	1348.4	939.4	483.6	675.2	553.0	531.5	470.4	293.5	167.3	63.7
wB97X	def2QZVPD	3475.2	2447.0	2400.7	2310.8	2200.8	1308.0	921.1	474.4	731.5	585.5	547.7	473.2	292.5	167.6	63.7
wB97XD	def2QZVPD	3470.1	2414.6	2370.8	2289.2	2180.3	1319.7	926.6	477.2	711.8	567.7	537.9	469.1	289.6	166.7	63.8
CAM-B3LYP	def2QZVPD	3471.0	2420.8	2368.6	2298.3	2188.7	1325.1	930.6	479.1	716.2	586.7	548.2	474.7	296.5	168.3	63.7
M062X	def2QZVPD	3480.6	2430.1	2367.3	2302.8	2189.5	1323.5	928.8	478.2	726.8	582.7	545.6	473.8	292.6	166.4	62.0
SVWN	def2QZVPD	3377.0	2290.0	2231.1	2189.4	2072.5	1371.6	948.0	487.3	620.3	548.9	521.8	459.1	289.7	162.1	61.7
B3PW91	def2QZVPD	3462.7	2345.5	2283.4	2241.3	2131.2	1348.1	938.9	483.3	671.7	554.9	531.4	469.0	293.5	166.8	63.5
B3LYP	def2QZVPD	3460.1	2334.9	2271.9	2234.0	2127.4	1340.2	934.5	481.2	669.0	556.1	530.3	468.1	294.3	167.0	63.6
Kr[6]		n/a	n/a	2200	n/a	n/a	n/a	n/a	n/a	n/a	n/a	n/a	n/a	n/a	n/a	

Table S98: Harmonic unscaled vibrational frequencies (in cm^{-1}) for S_1 of HC_9N

method	basis	1	2	3	4	5	6	7	8	9	10	11	12	13	14	15	16	17	18	19
tHCTHhyb	aug-cc-pVDZ	3451.8	2142.4	2133.8	2057.7	1964.4	1893.3	1530.9	1141.9	776.6	397.2	478.2	434.4	195.6	124.5	93.9	35.2	-441.5	-1190.7	-2809.6
VSXC	aug-cc-pVDZ	3479.8	2142.0	2128.8	2066.9	2022.9	1891.7	1535.4	1150.8	782.1	400.2	442.0	435.8	234.5	163.0	101.7	37.7	-278.3	-909.3	-2148.5
BMK	aug-cc-pVDZ	3469.8	2198.8	2036.3	2010.4	1883.7	1802.3	1534.6	1132.9	772.1	394.3	535.2	445.2	181.7	109.6	82.2	36.9	-546.6	-1462.6	-3549.9
B1B95	aug-cc-pVDZ	3484.7	2197.8	2136.8	2088.8	1929.2	1920.5	1559.2	1158.4	789.0	404.0	528.0	454.9	229.7	150.2	99.4	36.1	-366.2	-1092.2	-2609.5
B3P86	aug-cc-pVDZ	3474.0	2178.8	2155.5	2086.8	1966.1	1923.0	1554.5	1158.0	788.1	403.2	514.7	449.0	216.2	139.7	96.1	35.9	-409.0	-1155.0	-2737.8
B98	aug-cc-pVDZ	3462.5	2158.7	2126.1	2064.3	1930.9	1903.8	1538.7	1145.2	779.3	398.9	497.9	439.7	198.2	124.5	94.8	35.9	-449.8	-1216.6	-2877.3
B971	aug-cc-pVDZ	3461.6	2154.2	2128.0	2063.4	1936.5	1901.8	1537.5	1144.8	779.0	398.7	496.0	438.9	203.5	131.5	95.1	35.8	-428.4	-1175.5	-2783.0
B972	aug-cc-pVDZ	3490.7	2180.9	2150.7	2086.8	1955.3	1923.6	1554.5	1157.5	788.2	403.6	512.9	454.0	236.1	154.6	100.6	37.3	-325.5	-1006.9	-2404.5
wB97X	aug-cc-pVDZ	3477.2	2259.5	2093.3	1960.6	1904.9	1694.9	1551.5	1140.2	780.4	401.0	592.9	461.0	231.2	147.9	99.8	38.3	-342.1	-1050.2	-2513.6
wB97XD	aug-cc-pVDZ	3470.8	2227.2	2092.9	2036.1	1928.0	1790.2	1552.7	1145.9	782.4	401.3	561.8	453.2	214.6	135.8	98.6	37.7	-401.9	-1142.9	-2717.8
CAM-B3LYP	aug-cc-pVDZ	3475.7	2235.8	2091.9	2050.9	1933.1	1798.4	1555.7	1149.3	784.8	402.6	571.8	458.0	216.0	136.0	94.9	36.7	-428.8	-1216.2	-2911.3
M062X	aug-cc-pVDZ	3472.9	2194.8	2020.1	1852.4	1621.7	1553.1	1177.3	1101.7	778.5	394.7	556.6	452.7	223.8	133.3	98.4	38.1	-402.5	-1140.1	-2704.2
SVWN	aug-cc-pVDZ	3395.3	2183.3	2152.3	2088.2	2056.7	1894.6	1541.4	1156.4	784.6	400.2	475.6	435.5	207.7	137.0	91.5	36.1	-448.3	-1263.0	-3030.4
B3PW91	aug-cc-pVDZ	3470.2	2173.9	2149.9	2082.1	1959.9	1918.2	1550.9	1155.2	786.2	402.4	512.3	449.5	220.9	143.3	96.5	36.4	-392.6	-1124.6	-2673.3
B3LYP	aug-cc-pVDZ	3465.2	2159.0	2129.8	2070.1	1939.2	1910.0	1543.0	1149.5	782.1	400.4	510.1	444.9	208.5	132.9	95.0	36.5	-434.4	-1204.5	-2860.0
tHCTHhyb	aug-cc-pVTZ	3436.4	2141.7	2112.0	2052.9	1942.5	1893.1	1528.1	1140.1	776.6	397.9	700.1	539.9	523.7	479.9	433.1	310.5	191.9	107.8	41.8
VSXC	aug-cc-pVTZ	3469.0	2135.1	2128.5	2057.7	2014.9	1893.8	1528.8	1145.7	784.6	397.8	625.0	544.2	469.4	368.9	288.3	200.1	75.8	-24.6	-534.8
BMK	aug-cc-pVTZ	3430.6	2212.8	2038.2	1992.4	1889.8	1765.0	1530.2	1128.3	771.1	395.9	865.6	593.4	590.5	499.2	449.9	324.0	190.5	107.2	40.8
B3P86	aug-cc-pVTZ	3460.9	2177.4	2131.5	2079.1	1942.5	1919.8	1549.4	1154.8	786.9	403.4	771.8	568.4	558.9	499.2	447.4	318.4	194.6	108.7	42.3
B98	aug-cc-pVTZ	3445.8	2160.1	2105.4	2059.9	1910.4	1903.7	1535.4	1143.2	779.2	399.6	651.9	558.1	514.3	482.4	433.6	310.7	190.7	109.5	44.6
B971	aug-cc-pVTZ	3444.0	2154.3	2105.7	2057.8	1914.5	1900.9	1533.3	1142.1	778.4	399.1	618.0	554.9	505.2	478.4	430.5	308.5	190.7	108.6	44.8
B972	aug-cc-pVTZ	3476.7	2178.4	2127.4	2077.6	1932.5	1919.1	1547.9	1153.0	785.7	402.8	651.5	573.0	522.2	490.0	440.9	314.5	193.4	110.8	41.5
wB97X	aug-cc-pVTZ	3467.2	2260.1	2087.6	1953.6	1896.9	1675.0	1544.4	1135.7	778.3	400.7	674.1	652.4	544.7	498.9	452.0	315.7	195.7	109.1	35.4
wB97XD	aug-cc-pVTZ	3462.6	2228.9	2086.6	2021.3	1924.1	1770.1	1547.8	1142.5	781.6	401.6	754.6	622.1	557.8	500.0	449.7	315.7	190.0	105.6	38.0
CAM-B3LYP	aug-cc-pVTZ	3461.5	2237.9	2085.4	2033.3	1929.2	1775.3	1550.1	1146.0	783.9	402.9	700.8	628.6	550.9	502.3	449.7	317.1	189.1	103.7	35.8
M062X	aug-cc-pVTZ	3460.6	2205.3	2024.3	1860.6	1619.7	1549.7	1475.6	1131.7	777.4	398.5	769.2	625.7	565.5	499.7	446.2	319.0	185.1	102.8	42.2
SVWN	aug-cc-pVTZ	3370.7	2156.7	2145.4	2068.9	2049.7	1892.7	1534.0	1152.9	784.4	395.3	794.6	564.6	516.5	489.9	435.2	313.8	189.7	103.9	38.8
B3PW91	aug-cc-pVTZ	3454.9	2171.8	2124.7	2073.4	1935.2	1914.8	1545.1	1151.6	784.8	402.3	760.4	569.1	554.5	497.6	445.6	317.6	192.9	108.8	42.1
B3LYP	aug-cc-pVTZ	3452.7	2159.5	2105.7	2063.2	1916.3	1907.5	1538.9	1147.1	782.0	401.0	675.9	564.8	529.9	490.9	441.1	314.3	191.5	108.6	43.8
tHCTHhyb	def2SVPD	3433.2	2171.2	2128.7	2058.3	1962.9	1896.2	1534.2	1145.2	780.1	399.5	1264.6	757.4	571.6	545.0	480.5	358.1	217.6	118.5	43.9
VSXC	def2SVPD	3460.1	2159.4	2138.0	2065.1	2031.3	1895.1	1532.8	1149.8	782.3	400.6	1090.4	743.4	565.2	504.1	380.4	317.9	252.7	-97.7	-299.3
BMK	def2SVPD	3446.4	2234.4	2036.7	1998.7	1887.3	1796.7	1540.2	1137.6	777.2	398.2	1419.6	865.0	637.7	588.5	512.7	390.2	233.5	126.8	45.6
B1B95	def2SVPD	3467.3	2229.8	2130.7	2087.8	1931.0	1917.3	1560.8	1160.0	791.1	405.5	1211.6	730.1	599.0	561.3	481.4	352.5	214.6	116.9	43.0
B3P86	def2SVPD	3456.3	2207.2	2149.0	2086.0	1963.1	1924.8	1556.2	1160.1	790.7	405.1	1297.0	781.3	591.9	570.7	491.1	364.6	221.4	119.5	44.0
B98	def2SVPD	3444.6	2189.6	2120.6	2064.1	1929.3	1906.4	1541.5	1148.1	782.6	401.1	1236.6	749.7	575.1	559.5	484.4	360.1	219.0	119.7	44.3
B971	def2SVPD	3443.6	2184.3	2122.4	2063.1	1934.5	1904.4	1540.0	1147.4	782.1	400.8	1196.7	730.8	566.6	555.6	479.4	355.5	216.6	118.7	43.9
B972	def2SVPD	3472.4	2210.6	2142.9	2085.2	1950.3	1925.6	1555.4	1158.8	789.9	404.9	1184.7	696.8	580.1	542.0	469.8	341.5	209.1	115.1	42.3
wB97X	def2SVPD	3460.4	2291.2	2092.7	1957.7	1901.6	1690.7	1551.9	1140.3	781.7	402.4	1179.9	732.8	658.7	562.7	481.7	353.7	213.2	117.1	42.3
wB97XD	def2SVPD	3454.1	2258.2	2091.8	2029.1	1927.8	1786.9	1554.1	1146.5	784.4	403.0	1266.3	752.4	628.5	565.8	482.1	354.8	215.1	117.3	42.8
M062X	def2SVPD	3458.7	2229.0	2015.7	1848.2	1616.6	1546.4	1153.8	1033.9	780.0	394.8	1234.2	735.8	633.2	561.4	481.3	356.9	208.5	116.0	42.1
SVWN	def2SVPD	3377.0	2194.8	2177.1	2086.5	2082.2	1904.9	1546.8	1160.1	786.8	397.0	1608.8	984.7	692.2	550.4	514.4	395.4	244.2	128.5	45.6
B3PW91	def2SVPD	3451.9	2202.5	2143.3	2081.2	1956.5	1920.6	1552.6	1157.2	788.7	404.1	1288.1	766.7	585.1	567.4	486.6	359.5	217.8	118.7	43.6
B3LYP	def2SVPD	3447.7	2190.0	2124.3	2070.0	1937.7	1912.6	1545.8	1152.0	785.5	402.5	1205.9	779.4	595.2	570.9	497.5	372.4	225.7	121.4	44.9
tHCTHhyb	def2TZVPD	3441.6	2144.2	2116.7	2054.3	1947.1	1893.5	1529.3	1141.1	776.9	398.1	540.5	495.1	485.5	465.5	417.0	301.9	187.4	102.4	38.7
VSXC	def2TZVPD	3467.2	2133.3	2125.5	2061.2	2021.4	1888.8	1533.9	1146.6	780.8	399.3	632.6	499.6	452.6	388.0	331.9	219.8	187.5	-136.5	-292.6
BMK	def2TZVPD	3462.5	2213.0	2038.7	1998.8	1889.7	1775.1	1533.3	1131.5	772.8	396.2	590.5	516.1	491.0	473.6	421.1	303.0	185.9	107.7	43.3
B1B95	def2TZVPD	3474.7	2198.7	2115.9	2080.9	1924.8	1898.9	1553.8	1154.6	787.3	403.8	588.5	524.7	507.4	481.7	428.6	308.7	190.1	106.4	38.7
B3P86	def2TZVPD	3464.6	2180.2	2136.1	2080.8	1946.6	1920.6	1550.8	1155.9	787.5	403.7	569.7	521.0	502.7	479.6	425.8	307.7	189.7	105.7	38.4
B98	def2TZVPD	3452.6	2162.3	2109.7	2061.0	1914.3	1903.8	1536.5	1144.0	779.7	399.8	556.9	502.2	491.4	469.8	420.7	303.6	186.7	106.1	36.4
B971	def2TZVPD	3451.2	2156.3	2110.1	2058.7	1918.2	1900.8	1534.4	1142.9	778.7	399.4	554.5	498.9	489.0	467.5	418.8	302.8	185.3	103.9	36.8
B972	def2TZVPD	3478.8	2180.9	2129.4	2078.4	1934.2	1919.8	1549.1	1153.9	786.4	403.3	572.2	507.7	496.8	473.5	424.7	307.6	189.8	106.1	36.8
wB97X	def2TZVPD	3469.5	2262.4	2088.2	1952.7	1897.1	1676.6	1545.3	1136.4	778.9	400.9	653.6	526.4	510.5	480.8	429.4	305.8	188.3	108.9	44.7
wB97XD	def2TZVPD	3463.0	2231.5	2088.0	2023.0	1924.5	1773.1	1548.8	1143.2	782.1	401.9	622.4	518.2	503.6	477.0	426.5	304.3	188.2	105.3	44.6
CAM-B3LYP	def2TZVPD	3465.8	2240.7	2087.3	2038.0	1930.0	1780.5	1551.8	1147.2	784.7	403.3	628.6	525.9	523.2	487.7	435.3	308.5	191.0	109.9	47.0
M062X	def2TZVPD	3468.6	2209.1	2026.6	1862.8	1622.2	1552.3	1482.1	1133.4	778.1	398.7	629.7	522.4	509.4	481.3	427.0	308.2	186.5	107.4	42.5
SVWN	def2TZVPD	3376.4	2158.9	2148.3	2073.9	2056.8	1896.2	1538.1	1153.3	783.5	398.4	558.6	525.5	512.4	478.2	416.8	304.6	187.5	105.0	39.8
B3PW91	def2TZVPD	3458.4	2174.7	2129.7	2075.4	1939.6	1915.8	1546.7	1152.7	785.3	402.6	569.2	519.2	502.9	479.1	425.6	307.4	189.5	105.3	37.9
B3LYP	def2TZVPD	3456.7	2162.6	2111.3	2065.1	1921.0	1908.4	1540.4	1148.0	782.4	401.2	566.9	522.9	503.4	479.6	425.4	306.7	189.6	105.7	38.3

Table S99: Harmonic unscaled vibrational frequencies (in cm^{-1}) for S_2 of HC_9N

method	basis	1	2	3	4	5	6	7	8	9	10	11	12	13	14	15	16	17	18	19
tHCTHhyb	aug-cc-pVDZ	3452.1	2156.2	2146.7	2065.2	1999.0	1901.2	1527.1	1142.0	776.4	397.0	497.1	440.1	199.7	135.4	94.7	35.8	-431.9	-1184.8	-2803.7
VSXC	aug-cc-pVDZ	3479.7	2144.9	2134.6	2068.2	2035.5	1896.3	1532.9	1150.3	782.2	399.7	557.7	453.5	243.4	142.7	92.5	-49.1	-327.4	-880.8	-2097.7
BMK	aug-cc-pVDZ	3471.5	2227.9	2154.5	2074.4	1937.7	1918.0	1526.6	1137.6	774.2	395.5	551.9	451.3	184.0	121.6	87.3	37.7	-537.6	-1460.1	-3553.1
B1B95	aug-cc-pVDZ	3485.3	2215.1	2183.7	2108.2	1995.0	1946.7	1554.4	1160.1	789.8	404.3	544.5	461.2	232.7	157.7	98.1	38.3	-357.9	-1090.7	-2615.4
B3P86	aug-cc-pVDZ	3474.5	2193.3	2177.4	2097.5	2011.3	1933.3	1550.4	1158.6	788.3	403.4	532.3	454.8	219.2	148.6	94.6	37.4	-399.4	-1150.4	-2736.1
B98	aug-cc-pVDZ	3463.1	2172.4	2150.8	2075.1	1977.1	1913.7	1534.5	1145.4	779.5	398.9	517.0	445.8	203.5	136.8	93.9	36.0	-438.8	-1208.8	-2868.0
B971	aug-cc-pVDZ	3462.1	2168.2	2151.5	2074.1	1982.2	1912.2	1533.2	1145.1	779.2	398.7	514.7	445.2	207.5	142.5	94.3	36.5	-417.5	-1168.4	-2775.8
B972	aug-cc-pVDZ	3491.2	2194.9	2173.6	2097.4	1999.8	1934.3	1550.6	1157.9	788.3	403.6	531.4	460.6	240.4	161.2	99.0	37.8	-314.7	-1004.1	-2407.0
wB97X	aug-cc-pVDZ	3477.9	2273.8	2130.2	2086.3	1959.2	1802.0	1543.6	1142.9	780.8	401.5	609.4	468.7	236.2	159.1	104.3	37.4	-322.9	-1044.5	-2516.1
wB97XD	aug-cc-pVDZ	3471.4	2242.3	2142.2	2103.7	1954.4	1873.0	1546.2	1147.0	782.7	401.7	578.1	461.1	217.9	147.9	94.9	35.0	-386.3	-1133.3	-2710.1
CAM-B3LYP	aug-cc-pVDZ	3476.1	2249.2	2141.0	2105.0	1957.5	1878.9	1550.8	1151.5	785.5	403.0	587.7	466.2	219.4	149.1	97.9	35.9	-414.2	-1210.3	-2910.6
M062X	aug-cc-pVDZ	3478.0	2260.1	2185.9	2121.3	1972.9	1924.2	1546.2	1152.2	785.3	402.8	593.3	465.0	218.9	154.2	98.9	36.4	-388.9	-1146.6	-2741.4
SVWN	aug-cc-pVDZ	3394.6	2199.7	2152.4	2130.9	2063.6	1902.2	1526.3	1148.9	792.0	398.6	514.6	453.0	240.2	129.8	64.3	-20.0	-426.1	-1233.0	-3013.5
B3PW91	aug-cc-pVDZ	3470.8	2188.5	2172.2	2093.1	2005.7	1929.3	1546.8	1155.9	786.5	402.5	529.7	455.4	223.4	151.6	95.9	37.4	-383.4	-1120.6	-2672.7
B3LYP	aug-cc-pVDZ	3465.6	2172.3	2151.4	2079.9	1982.4	1919.7	1539.2	1149.8	782.4	400.5	528.6	450.7	212.3	143.5	94.5	36.4	-424.5	-1199.1	-2856.3
tHCTHhyb	aug-cc-pVTZ	3436.6	2152.7	2126.1	2060.2	1975.8	1900.3	1524.1	1139.9	776.3	397.8	712.6	559.1	537.4	488.8	439.1	314.9	192.2	103.0	40.1
VSXC	aug-cc-pVTZ	3464.0	2135.1	2121.0	2055.4	2015.9	1873.5	1524.3	1144.0	777.0	396.9	625.4	543.7	465.9	368.4	287.7	216.5	77.9	-34.2	-534.1
B3P86	aug-cc-pVTZ	3461.2	2190.0	2153.0	2089.5	1986.3	1929.5	1545.4	1155.3	787.2	403.4	783.4	584.5	573.4	508.1	452.0	322.8	198.7	110.4	40.8
B98	aug-cc-pVTZ	3445.9	2172.1	2129.0	2070.2	1954.3	1913.7	1531.1	1143.3	779.2	399.5	665.2	576.9	532.7	493.1	442.8	315.2	197.2	106.5	42.5
B971	aug-cc-pVTZ	3444.3	2166.3	2128.4	2068.0	1958.5	1910.9	1529.2	1142.4	778.5	399.0	631.7	571.5	522.4	489.6	440.4	313.9	196.9	107.2	42.8
B972	aug-cc-pVTZ	3477.1	2190.9	2149.1	2087.6	1975.0	1929.0	1544.1	1153.3	785.8	402.8	664.3	585.7	538.1	499.2	446.2	318.6	199.0	108.4	41.4
wB97X	aug-cc-pVTZ	3467.5	2273.1	2116.5	2067.2	1950.4	1774.4	1537.1	1138.3	779.0	401.1	706.8	666.9	566.8	511.5	452.7	318.8	196.1	109.8	39.5
wB97XD	aug-cc-pVTZ	3462.8	2242.4	2122.6	2094.5	1949.3	1849.5	1541.6	1144.2	782.0	401.9	781.6	635.6	576.0	509.3	451.2	319.7	192.0	105.0	38.8
CAM-B3LYP	aug-cc-pVTZ	3462.0	2249.9	2119.0	2094.7	1951.6	1851.4	1545.2	1147.9	784.5	403.3	722.9	641.5	572.2	512.7	455.1	321.9	192.9	106.0	38.5
M062X	aug-cc-pVTZ	3464.1	2257.6	2156.5	2109.1	1963.2	1889.2	1538.2	1146.0	782.3	402.1	781.4	657.9	583.5	515.2	460.8	323.0	195.8	107.8	34.4
SVWN	aug-cc-pVTZ	3364.7	2167.2	2143.9	2084.8	2047.0	1893.5	1522.8	1142.2	787.7	400.8	803.3	610.7	548.5	504.7	379.8	279.3	112.1	-63.1	-254.2
B3PW91	aug-cc-pVTZ	3455.4	2184.7	2146.7	2084.4	1979.9	1925.4	1541.3	1152.2	784.9	402.4	771.2	583.7	569.2	505.4	449.8	321.6	198.2	110.3	42.0
B3LYP	aug-cc-pVTZ	3453.0	2171.3	2126.6	2072.8	1957.3	1917.0	1535.0	1147.3	782.0	400.9	689.8	580.1	547.6	500.2	445.0	317.5	198.9	108.1	42.2
tHCTHhyb	def2SVPD	3433.5	2183.0	2143.7	2066.6	1997.7	1904.4	1530.5	1145.2	780.0	399.4	1266.3	764.3	582.1	561.6	482.7	358.7	220.3	118.9	43.2
VSXC	def2SVPD	3460.1	2159.4	2138.0	2065.2	2031.4	1895.2	1532.8	1149.8	782.3	400.6	1090.5	743.6	564.2	503.9	379.2	317.0	252.6	-91.2	-298.7
BMK	def2SVPD	3448.3	2263.0	2145.1	2074.7	1935.3	1919.4	1532.3	1142.8	779.6	399.4	1421.6	874.2	649.4	605.5	513.3	390.7	235.9	127.8	45.7
B1B95	def2SVPD	3467.5	2245.1	2178.1	2108.4	1992.0	1948.6	1556.2	1162.1	792.1	405.9	1217.8	740.6	615.0	571.0	483.6	354.2	217.1	118.0	43.5
B3P86	def2SVPD	3456.8	2220.4	2171.6	2097.5	2008.8	1935.5	1552.4	1160.8	790.9	405.2	1300.5	789.2	605.4	583.3	493.9	365.1	223.3	120.7	44.2
B98	def2SVPD	3445.0	2202.4	2145.6	2075.6	1975.3	1917.4	1537.6	1148.6	782.7	401.1	1239.1	757.6	589.9	572.4	487.0	361.1	222.0	120.1	43.0
B971	def2SVPD	3444.1	2197.3	2146.3	2074.6	1980.4	1915.3	1536.1	1148.0	782.2	400.9	1199.6	739.2	583.3	566.3	481.8	356.5	219.0	118.9	42.3
B972	def2SVPD	3472.9	2223.5	2165.8	2096.4	1994.9	1936.3	1551.6	1159.4	790.1	404.9	1189.3	706.4	597.7	550.8	471.8	343.2	212.7	116.1	42.6
wB97X	def2SVPD	3461.0	2304.8	2127.2	2079.5	1958.0	1795.3	1544.4	1143.5	782.4	402.8	1189.2	747.1	674.0	577.4	489.7	357.5	215.3	118.1	41.7
wB97XD	def2SVPD	3454.6	2272.6	2137.2	2100.9	1954.4	1869.3	1547.9	1148.6	784.9	403.3	1272.2	764.6	644.6	578.2	485.2	358.1	217.8	119.2	41.4
CAM-B3LYP	def2SVPD	3458.3	2281.7	2136.6	2102.3	1959.1	1874.5	1552.9	1153.6	788.2	405.0	1227.1	799.0	654.9	608.0	506.1	377.0	229.6	123.1	43.9
M062X	def2SVPD	3463.9	2292.6	2176.9	2118.9	1973.6	1915.2	1547.1	1153.1	786.9	404.3	1228.8	747.5	669.5	577.4	484.0	356.2	217.7	117.6	41.8
SVWN	def2SVPD	3375.7	2196.4	2175.3	2108.8	2078.1	1906.6	1542.7	1160.3	788.6	402.9	1593.9	1120.8	703.7	626.8	557.0	415.9	252.8	68.6	-105.8
B3PW91	def2SVPD	3452.5	2215.9	2166.2	2093.0	2003.0	1931.6	1548.7	1157.9	789.0	404.2	1291.6	775.0	600.0	578.3	489.1	360.3	220.9	119.3	43.9
B3LYP	def2SVPD	3448.0	2202.5	2146.2	2080.7	1980.7	1922.8	1542.0	1152.4	785.5	402.6	1209.0	786.8	606.3	587.1	499.8	372.9	227.3	121.7	43.2
tHCTHhyb	def2TZVPD	3441.8	2155.6	2131.2	2061.8	1980.8	1901.2	1525.6	1140.9	776.7	398.0	556.8	512.0	498.1	474.1	420.6	305.6	191.0	106.3	41.7
VSXC	def2TZVPD	3466.7	2134.3	2128.1	2057.9	2022.5	1885.1	1520.3	1145.0	779.2	398.7	628.3	502.8	452.3	391.8	329.0	219.2	191.3	-125.2	-293.3
BMK	def2TZVPD	3463.6	2238.1	2132.6	2075.5	1929.2	1902.2	1525.2	1136.2	774.8	397.2	607.7	537.6	505.7	485.9	427.5	307.0	189.0	107.8	38.4
B1B95	def2TZVPD	3475.2	2213.8	2160.9	2099.9	1970.3	1941.6	1549.2	1156.4	788.1	404.1	602.9	541.5	519.8	491.1	433.2	312.5	195.0	106.7	38.9
B3P86	def2TZVPD	3465.0	2193.1	2157.9	2091.5	1990.9	1930.8	1546.9	1156.3	787.6	403.7	586.5	537.0	515.2	489.2	430.3	311.3	194.2	108.5	39.3
B98	def2TZVPD	3452.9	2174.5	2133.5	2071.6	1958.8	1914.1	1532.4	1144.3	779.7	399.6	573.8	519.6	504.6	480.1	426.4	308.2	192.9	106.2	41.4
B971	def2TZVPD	3451.6	2168.8	2133.0	2069.3	1962.8	1911.1	1530.3	1143.3	778.9	399.3	570.5	515.7	500.9	477.6	423.9	307.2	191.9	105.7	39.1
B972	def2TZVPD	3479.2	2193.6	2151.4	2088.8	1977.4	1929.8	1545.3	1154.4	786.5	403.3	588.3	524.6	509.1	484.0	429.6	311.7	193.6	107.9	39.6
wB97X	def2TZVPD	3469.9	2275.5	2117.3	2068.3	1950.5	1776.7	1538.2	1139.2	779.5	401.3	670.3	556.2	530.8	498.8	438.9	311.8	191.8	104.2	39.1
wB97XD	def2TZVPD	3463.3	2245.2	2124.7	2096.5	1949.7	1853.0	1542.7	1144.9	782.5	402.1	639.3	544.1	522.4	492.9	434.3	310.4	191.6	105.3	41.1
CAM-B3LYP	def2TZVPD	3466.2	2252.9	2123.4	2098.2	1952.9	1857.3	1547.0	1149.2	785.3	403.6	645.3	550.1	543.1	502.1	441.5	315.7	196.2	109.8	45.2
M062X	def2TZVPD	3471.7	2259.9	2164.1	2112.9	1965.7	1896.4	1540.5	1147.8	783.2	402.5	660.3	543.9	530.8	496.9	437.4	311.8	193.2	107.5	38.4
SVWN	def2TZVPD	3374.3	2167.4	2150.4	2080.6	2063.3	1894.8	1533.9	1154.0	780.9	399.5	653.0	627.4	409.4	368.2	344.3	241.7	72.5	-32.9	-256.3
B3PW91	def2TZVPD	3458.9	2187.7	2151.8	2086.3	1984.7	1926.2	1542.8	1153.2	785.4	402.6	585.3	534.5	515.3	487.9	431.1	311.3	194.3	106.4	40.7
B3LYP	def2TZVPD	3457.0	2174.7	2132.4	2075.1	1962.9	1918.2	1536.6	1148.4	782.5	401.2	584.5	537.9	516.5	488.3	430.2	310.5	194.4	106.1	40.2

Table S100: Harmonic unscaled vibrational frequencies (in cm^{-1}) for S_0 of HC_9N

method	basis	1	2	3	4	5	6	7	8	9	10	11	12	13	14	15	16	17	18	19
tHCTHhyb	aug-cc-pVDZ	3459.9	2307.9	2261.9	2206.7	2174.1	2082.1	1428.7	1114.7	761.9	388.8	584.4	460.4	179.3	173.5	93.4	37.6	-404.5	-1188.6	-2797.0
VSXC	aug-cc-pVDZ	3485.4	2278.8	2245.0	2169.3	2155.4	2065.1	1449.0	1126.3	769.7	393.2	559.7	473.4	244.7	186.1	103.6	39.5	-215.2	-902.7	-2154.0
BMK	aug-cc-pVDZ	3481.9	2390.1	2309.6	2286.4	2217.4	2115.6	1413.7	1107.2	755.9	385.4	628.0	467.3	181.5	128.6	51.5	37.4	-528.4	-1474.5	-3533.8
B1B95	aug-cc-pVDZ	3493.8	2380.2	2323.0	2283.5	2234.7	2142.9	1436.3	1125.9	771.0	393.1	627.1	480.9	219.4	184.7	100.8	38.8	-317.2	-1084.7	-2590.8
B3P86	aug-cc-pVDZ	3482.2	2352.7	2302.2	2252.5	2214.0	2122.2	1442.7	1128.0	771.7	393.6	615.0	474.7	202.8	180.6	97.9	38.4	-365.5	-1147.3	-2717.3
B98	aug-cc-pVDZ	3471.3	2334.1	2283.4	2235.2	2197.1	2106.9	1424.5	1114.6	762.6	389.0	601.8	464.5	179.3	176.2	93.9	38.0	-411.1	-1210.9	-2854.0
B971	aug-cc-pVDZ	3470.1	2328.9	2279.5	2230.8	2192.7	2102.6	1425.2	1114.6	762.6	389.0	599.7	464.4	185.8	178.7	95.8	38.1	-388.8	-1170.8	-2764.0
B972	aug-cc-pVDZ	3498.7	2356.3	2305.4	2256.0	2218.0	2127.2	1439.0	1126.2	770.9	393.3	616.7	481.1	229.9	185.2	102.1	39.0	-269.8	-1000.7	-2394.2
wB97X	aug-cc-pVDZ	3488.4	2454.8	2408.1	2377.9	2284.1	2198.4	1385.8	1098.4	754.8	384.6	674.6	487.1	221.4	186.3	102.6	39.6	-275.3	-1025.7	-2455.3
wB97XD	aug-cc-pVDZ	3481.7	2420.6	2367.0	2351.5	2259.4	2174.1	1398.6	1105.0	758.5	386.5	651.0	478.8	197.2	181.5	98.3	38.9	-349.7	-1125.1	-2669.0
CAM-B3LYP	aug-cc-pVDZ	3485.8	2425.0	2358.2	2355.5	2267.8	2180.6	1406.3	1110.3	761.8	388.1	660.3	482.6	198.3	183.8	97.4	38.5	-376.0	-1192.7	-2843.7
M062X	aug-cc-pVDZ	3487.7	2433.7	2359.2	2346.4	2271.5	2181.6	1401.5	1107.4	760.7	387.1	658.6	484.0	196.5	181.8	97.4	37.8	-351.2	-1135.6	-2690.4
SVWN	aug-cc-pVDZ	3401.8	2296.1	2254.7	2193.6	2156.9	2053.1	1470.8	1139.2	776.4	395.6	575.4	461.8	199.3	175.0	93.5	37.5	-418.2	-1260.6	-3021.1
B3PW91	aug-cc-pVDZ	3478.4	2347.7	2297.5	2247.6	2209.7	2118.4	1438.6	1125.0	769.8	392.6	613.7	475.4	208.8	181.6	98.8	38.5	-347.5	-1117.5	-2654.7
B3LYP	aug-cc-pVDZ	3473.0	2334.2	2286.1	2233.6	2201.4	2113.0	1428.3	1117.9	765.0	390.3	613.0	470.4	194.1	180.1	96.3	38.4	-392.0	-1193.8	-2827.7
tHCTHhyb	aug-cc-pVTZ	3443.8	2306.5	2259.8	2196.5	2177.9	2090.5	1417.8	1109.7	760.4	388.4	738.3	640.4	572.3	515.2	457.7	307.1	202.6	110.1	40.9
VSXC	aug-cc-pVTZ	3471.3	2272.2	2238.6	2162.6	2147.8	2066.3	1438.2	1120.1	766.5	391.9	613.9	559.6	525.3	502.7	454.2	307.8	205.7	112.3	41.9
B1B95	aug-cc-pVTZ	3478.1	2374.8	2316.8	2270.2	2233.7	2144.6	1423.7	1118.7	767.4	391.8	693.1	681.2	578.4	529.2	466.8	310.9	206.2	111.6	41.2
B3P86	aug-cc-pVTZ	3468.5	2348.5	2297.4	2240.2	2214.6	2125.8	1431.8	1122.5	769.6	393.0	807.5	666.2	606.2	533.5	470.2	314.5	206.4	111.7	41.3
B98	aug-cc-pVTZ	3454.0	2333.3	2281.9	2226.4	2201.2	2114.4	1413.9	1109.5	761.0	388.7	695.6	656.6	566.3	517.2	458.3	307.4	204.0	110.8	41.1
B971	aug-cc-pVTZ	3452.1	2326.7	2276.9	2221.0	2195.8	2109.0	1414.0	1109.1	760.5	388.5	663.2	653.8	556.5	513.8	456.2	305.6	203.4	110.6	41.1
B972	aug-cc-pVTZ	3483.9	2350.3	2298.3	2242.1	2215.9	2127.5	1427.4	1119.5	767.6	392.0	693.7	670.1	572.6	525.6	465.6	310.5	206.1	111.8	41.4
wB97X	aug-cc-pVTZ	3477.8	2449.6	2397.0	2374.0	2284.3	2199.2	1375.5	1092.7	752.4	383.8	731.1	729.4	602.6	541.0	470.6	309.9	206.9	111.9	41.5
wB97XD	aug-cc-pVTZ	3472.8	2416.8	2356.8	2349.1	2261.5	2176.8	1390.2	1100.7	757.1	386.3	801.8	706.5	612.3	538.7	470.8	309.8	205.6	111.3	41.3
CAM-B3LYP	aug-cc-pVTZ	3471.2	2422.2	2354.9	2345.7	2270.1	2185.1	1395.2	1104.9	760.1	387.8	754.2	713.6	607.2	541.8	471.1	314.1	207.6	111.9	41.3
M062X	aug-cc-pVTZ	3475.1	2430.5	2356.4	2341.0	2272.6	2184.4	1394.0	1103.0	758.6	387.1	805.7	719.5	619.8	543.1	473.8	312.5	205.6	110.3	39.5
SVWN	aug-cc-pVTZ	3377.3	2284.7	2245.6	2173.9	2154.0	2056.5	1458.3	1133.0	774.1	395.1	825.8	613.3	603.6	523.5	458.4	310.9	200.8	108.3	40.0
B3PW91	aug-cc-pVTZ	3462.4	2342.6	2291.6	2234.3	2209.4	2121.2	1427.1	1119.0	767.2	391.8	796.9	665.7	601.9	532.1	469.2	313.7	206.1	111.5	41.2
B3LYP	aug-cc-pVTZ	3460.0	2331.8	2282.4	2222.7	2203.1	2117.9	1418.1	1113.3	763.8	390.1	719.4	664.3	580.1	525.9	464.3	311.7	205.8	111.5	41.3
tHCTHhyb	def2SVPD	3440.5	2325.6	2264.9	2202.0	2181.3	2086.0	1429.5	1117.8	765.7	390.8	1231.5	757.1	644.6	589.2	494.6	349.4	229.4	121.9	44.0
VSXC	def2SVPD	3465.4	2289.5	2243.8	2169.4	2152.7	2064.8	1447.5	1126.9	771.1	393.8	1062.6	662.7	611.4	543.6	476.0	326.2	219.8	118.0	43.3
BMK	def2SVPD	3458.5	2417.5	2313.4	2279.8	2226.2	2124.3	1412.7	1111.4	762.2	388.6	1363.5	854.1	688.1	643.3	519.4	378.3	245.5	128.9	44.4
B1B95	def2SVPD	3476.2	2398.1	2325.2	2276.9	2240.9	2145.5	1436.1	1127.6	773.3	394.4	1176.9	736.4	687.3	587.2	495.8	344.0	227.5	120.9	43.9
B3P86	def2SVPD	3464.2	2368.4	2304.2	2246.4	2220.0	2124.6	1442.6	1130.1	774.6	395.2	1262.7	781.5	670.5	606.0	505.1	356.2	232.2	123.1	44.5
B98	def2SVPD	3452.9	2352.2	2286.1	2229.5	2204.0	2110.0	1425.7	1117.7	766.3	391.0	1196.9	750.1	660.1	590.6	496.6	350.9	231.1	122.8	44.4
B971	def2SVPD	3451.7	2346.1	2282.1	2225.2	2199.5	2105.5	1426.0	1117.5	766.0	390.9	1160.0	733.4	656.7	582.5	492.7	346.4	228.6	121.6	44.0
B972	def2SVPD	3479.9	2372.5	2306.4	2248.7	2223.7	2129.7	1437.4	1127.0	772.7	394.3	1157.4	706.9	673.6	568.0	486.1	332.9	223.4	119.2	43.6
wB97X	def2SVPD	3470.9	2467.6	2399.9	2381.3	2290.1	2199.5	1384.4	1099.0	756.6	385.6	1127.6	740.9	730.5	594.6	497.7	344.7	226.8	120.8	44.4
wB97XD	def2SVPD	3464.4	2433.3	2359.4	2354.7	2265.9	2175.3	1398.7	1106.7	760.9	388.0	1213.4	754.1	707.8	594.2	497.7	344.2	228.0	121.5	45.0
CAM-B3LYP	def2SVPD	3467.6	2442.3	2361.3	2349.2	2274.6	2183.4	1406.5	1112.7	765.1	389.9	1153.3	780.9	714.9	621.7	513.5	365.8	237.8	125.7	45.2
M062X	def2SVPD	3472.4	2453.3	2362.1	2341.7	2278.1	2184.6	1402.9	1109.4	762.7	388.8	1172.5	737.7	727.2	590.7	495.4	343.4	228.8	121.5	43.7
SVWN	def2SVPD	3381.5	2311.7	2261.5	2194.4	2166.7	2058.7	1473.8	1143.4	780.9	398.2	1583.4	974.2	703.3	619.7	541.4	393.3	248.8	130.7	46.1
B3PW91	def2SVPD	3459.7	2363.6	2299.2	2241.3	2215.6	2120.8	1438.3	1126.8	772.4	394.1	1255.5	769.0	669.5	598.1	501.2	351.1	230.1	122.2	44.3
B3LYP	def2SVPD	3455.1	2352.3	2289.2	2228.8	2208.4	2116.3	1429.3	1120.9	768.7	392.2	1157.2	773.7	666.7	611.9	509.1	364.5	236.2	125.0	45.0
tHCTHhyb	def2TZVPD	3448.2	2307.8	2260.5	2197.6	2178.2	2089.4	1420.7	1111.5	761.4	388.9	640.9	546.1	531.1	500.2	441.1	299.5	201.3	109.5	40.8
VSXC	def2TZVPD	3470.6	2272.4	2238.2	2162.4	2147.6	2065.0	1440.4	1121.8	767.6	392.3	754.3	615.4	521.9	503.3	453.5	311.7	206.4	112.7	42.0
BMK	def2TZVPD	3474.1	2401.7	2317.6	2286.7	2234.1	2140.1	1394.5	1099.1	754.2	384.7	913.4	683.3	562.6	522.4	453.4	307.6	203.7	110.9	41.4
B1B95	def2TZVPD	3482.6	2376.2	2317.3	2270.2	2234.4	2144.9	1426.3	1120.6	768.6	392.3	681.8	578.0	555.2	518.1	453.2	305.9	205.3	111.0	41.1
B3P86	def2TZVPD	3471.6	2350.0	2298.3	2240.9	2215.4	2125.9	1434.1	1124.1	770.5	393.4	666.4	571.7	548.8	514.8	450.3	305.0	204.5	110.9	41.2
B98	def2TZVPD	3460.1	2334.1	2282.1	2226.8	2201.0	2113.3	1416.7	1111.4	762.0	389.1	656.0	555.7	538.9	505.6	444.9	301.9	203.0	110.3	41.0
B971	def2TZVPD	3458.5	2327.3	2276.9	2221.2	2195.4	2107.7	1416.3	1110.6	761.4	388.8	777.6	653.4	545.9	513.4	451.5	305.7	203.5	110.9	41.2
B972	def2TZVPD	3485.4	2351.5	2298.8	2242.1	2216.8	2128.1	1429.2	1121.0	768.5	392.4	670.0	560.8	543.2	512.0	451.1	304.9	205.0	111.1	41.2
wB97X	def2TZVPD	3479.1	2449.6	2395.2	2373.6	2284.5	2199.6	1376.2	1093.5	752.9	383.9	799.0	730.4	579.8	536.3	461.4	308.1	206.5	111.9	41.8
wB97XD	def2TZVPD	3472.3	2417.3	2356.0	2349.2	2262.0	2177.4	1391.2	1101.5	757.5	386.5	705.9	585.5	560.9	519.3	451.1	300.1	203.8	110.5	41.3
CAM-B3LYP	def2TZVPD	3474.5	2423.4	2355.4	2345.5	2270.5	2185.0	1397.5	1106.5	760.9	388.1	714.7	608.5	576.3	528.6	456.4	307.7	206.4	111.5	41.2
M062X	def2TZVPD	3481.9	2431.7	2357.0	2341.6	2273.2	2184.7	1395.9	1104.3	759.3	387.3	786.9	723.3	578.9	532.8	460.5	306.5	204.4	110.1	39.4
SVWN	def2TZVPD	3379.0	2289.5	2249.4	2179.0	2157.5	2058.4	1460.8	1134.8	775.1	395.7	618.6	593.6	552.3	509.6	442.0	302.7	199.9	108.4	40.3
B3PW91	def2TZVPD	3465.3	2344.3	2292.7	2235.3	2210.3	2121.3	1429.4	1120.6	768.1	392.2	665.7	570.2	549.3	514.2	450.3	304.7	204.4	110.8	41.2
B3LYP	def2TZVPD	3463.3	2333.4	2283.3	2223.4	2203.8	2117.7	1420.5	1114.9	764.6	390.4	665.3	573.7	550.7	513.9	449.5	305.4	204.6	111.1	41.3
Kr[7]		n/a	n/a	n/a	2143	n/a	n/a	n/a	n/a	n/a	n/a	n/a	n/a	n/a	n/a	n/a	n/a	n/a	n/a	n/a

Table S101: Harmonic unscaled vibrational frequencies (in cm^{-1}) for S_1 of C_2N_2

method	basis	1	2	3	4	5
tHCTHhyb	aug-cc-pVDZ	2218.0	926.5	1460.2	337.9	237.0
VSXC	aug-cc-pVDZ	2211.8	922.0	1516.7	325.3	233.3
BMK	aug-cc-pVDZ	2186.6	936.2	1310.8	354.8	244.6
B1B95	aug-cc-pVDZ	2255.4	950.5	1435.1	370.4	243.0
B3P86	aug-cc-pVDZ	2252.4	944.3	1468.6	361.9	241.2
B98	aug-cc-pVDZ	2223.8	934.2	1435.1	348.6	240.4
B971	aug-cc-pVDZ	2221.5	932.6	1438.0	346.7	239.4
B972	aug-cc-pVDZ	2252.9	944.8	1464.8	361.4	242.2
wB97X	aug-cc-pVDZ	2251.8	954.6	1362.2	374.2	244.4
wB97XD	aug-cc-pVDZ	2251.9	949.9	1403.0	366.9	243.8
CAM-B3LYP	aug-cc-pVDZ	2253.2	955.2	1394.9	378.0	244.5
M062X	aug-cc-pVDZ	2110.8	948.7	1208.3	382.6	243.3
SVWN	aug-cc-pVDZ	2238.0	928.5	1544.7	339.8	228.6
B3PW91	aug-cc-pVDZ	2247.4	942.3	1464.3	361.8	241.2
B3LYP	aug-cc-pVDZ	2227.4	937.3	1445.7	358.0	239.2
tHCTHhyb	aug-cc-pVTZ	2211.4	930.0	1467.7	366.9	242.8
VSXC	aug-cc-pVTZ	2200.1	921.3	1514.8	358.1	241.9
BMK	aug-cc-pVTZ	2191.8	944.9	1339.0	382.3	251.3
B1B95	aug-cc-pVTZ	2244.0	950.6	1437.3	395.6	249.9
B3P86	aug-cc-pVTZ	2243.5	946.4	1473.6	386.0	248.1
B98	aug-cc-pVTZ	2216.6	937.5	1442.7	375.8	246.9
B971	aug-cc-pVTZ	2213.1	935.3	1444.5	376.8	245.7
B972	aug-cc-pVTZ	2241.4	945.0	1466.3	384.9	248.3
wB97X	aug-cc-pVTZ	2241.8	955.8	1369.3	398.1	251.0
wB97XD	aug-cc-pVTZ	2242.9	952.6	1410.0	394.0	249.5
CAM-B3LYP	aug-cc-pVTZ	2244.6	957.8	1404.0	397.9	249.5
M062X	aug-cc-pVTZ	2110.0	949.2	1213.8	410.6	249.4
SVWN	aug-cc-pVTZ	2224.1	930.2	1547.7	375.8	234.4
B3PW91	aug-cc-pVTZ	2236.8	943.8	1468.0	385.4	247.5
B3LYP	aug-cc-pVTZ	2219.6	940.5	1453.6	383.9	246.3
tHCTHhyb	def2SVPD	2238.1	938.0	1493.4	370.9	242.5
VSXC	def2SVPD	2227.3	931.1	1641.6	323.1	238.0
BMK	def2SVPD	2217.4	948.9	1349.1	391.3	250.5
B1B95	def2SVPD	2274.3	960.7	1464.5	395.6	247.0
B3P86	def2SVPD	2269.2	954.2	1497.8	391.3	246.3
B98	def2SVPD	2243.1	945.1	1467.2	380.1	245.6
B971	def2SVPD	2240.8	943.5	1470.1	376.8	244.4
B972	def2SVPD	2269.4	954.7	1492.9	384.5	245.5
wB97X	def2SVPD	2269.9	963.4	1386.7	396.5	248.5
wB97XD	def2SVPD	2270.0	959.4	1429.8	393.7	248.3
CAM-B3LYP	def2SVPD	2271.9	964.8	1423.6	407.6	250.9
M062X	def2SVPD	2130.6	958.3	1227.7	401.7	245.2
SVWN	def2SVPD	2261.3	939.8	1581.8	403.0	244.0
B3PW91	def2SVPD	2264.4	952.1	1493.2	390.6	246.2
B3LYP	def2SVPD	2247.0	948.2	1478.4	391.0	246.3
tHCTHhyb	def2TZVPD	2212.5	930.7	1468.8	372.0	244.0
VSXC	def2TZVPD	2192.0	923.2	1680.9	-260.1	245.3
BMK	def2TZVPD	2190.8	943.8	1332.7	396.9	252.2
B1B95	def2TZVPD	2245.5	952.0	1439.2	398.8	250.2
B3P86	def2TZVPD	2245.1	947.5	1475.3	394.7	248.9
B98	def2TZVPD	2218.0	938.2	1443.7	376.2	247.0
B971	def2TZVPD	2214.5	936.0	1445.6	377.1	246.4
B972	def2TZVPD	2242.4	946.2	1468.3	388.6	248.7
wB97X	def2TZVPD	2243.5	956.8	1370.9	406.3	252.4
wB97XD	def2TZVPD	2245.0	953.4	1412.3	406.3	251.1
CAM-B3LYP	def2TZVPD	2247.0	958.8	1405.3	411.8	252.6
M062X	def2TZVPD	2110.2	950.4	1215.7	411.1	250.1
SVWN	def2TZVPD	2236.9	946.3	1553.3	354.5	241.0
B3PW91	def2TZVPD	2238.8	945.0	1469.9	393.0	248.4
B3LYP	def2TZVPD	2221.9	941.7	1455.3	393.2	247.7
tHCTHhyb	def2QZVPD	2209.5	929.9	1467.7	365.4	240.3
VSXC	def2QZVPD	2202.4	925.3	1517.3	291.8	234.2
BMK	def2QZVPD	2185.9	943.2	1334.4	375.7	249.9
B1B95	def2QZVPD	2241.3	950.6	1436.9	389.7	245.3
B3P86	def2QZVPD	2241.1	946.2	1473.3	379.2	245.7
B98	def2QZVPD	2215.3	937.6	1443.5	373.3	244.9
B971	def2QZVPD	2211.7	935.3	1445.3	368.9	243.2
B972	def2QZVPD	2239.0	945.1	1466.2	378.3	245.1
wB97X	def2QZVPD	2239.1	955.3	1367.5	392.3	246.1
wB97XD	def2QZVPD	2241.4	952.2	1409.3	390.9	247.9
CAM-B3LYP	def2QZVPD	2242.9	957.1	1402.3	402.5	251.2
M062X	def2QZVPD	2106.8	949.6	1214.0	406.0	247.4
SVWN	def2QZVPD	2223.4	930.6	1548.2	381.5	233.4
B3PW91	def2QZVPD	2235.0	943.7	1467.9	378.4	245.1
B3LYP	def2QZVPD	2217.7	940.4	1453.7	375.9	244.4
gas[8]		n/a	n/a	n/a	275.80	n/a

Table S102: Harmonic unscaled vibrational frequencies (in cm^{-1}) for S_2 of C_2N_2

method	basis	1	2	3	4	5
tHCTHhyb	aug-cc-pVDZ	2232.6	924.8	1508.9	358.5	237.1
VSXC	aug-cc-pVDZ	2217.7	920.8	1527.7	394.4	228.7
BMK	aug-cc-pVDZ	2271.6	939.1	1486.2	363.3	244.7
B1B95	aug-cc-pVDZ	2289.6	949.7	1531.3	384.6	243.1
B3P86	aug-cc-pVDZ	2272.5	942.8	1530.8	380.0	241.5
B98	aug-cc-pVDZ	2246.3	933.0	1497.4	373.1	241.1
B971	aug-cc-pVDZ	2243.2	931.4	1500.9	369.2	239.9
B972	aug-cc-pVDZ	2273.2	943.3	1524.4	380.2	242.8
wB97X	aug-cc-pVDZ	2300.0	954.9	1459.3	401.4	246.4
wB97XD	aug-cc-pVDZ	2290.1	949.7	1486.2	394.3	245.2
CAM-B3LYP	aug-cc-pVDZ	2292.9	955.2	1482.5	405.9	246.3
M062X	aug-cc-pVDZ	2296.2	954.2	1492.7	380.0	244.9
SVWN	aug-cc-pVDZ	2238.9	924.5	1578.7	309.7	225.6
B3PW91	aug-cc-pVDZ	2267.8	940.8	1527.8	379.2	241.5
B3LYP	aug-cc-pVDZ	2247.9	936.0	1504.5	380.2	239.8
tHCTHhyb	aug-cc-pVTZ	2225.2	928.1	1513.4	387.1	241.9
VSXC	aug-cc-pVTZ	2206.6	921.3	1506.1	390.3	140.5
BMK	aug-cc-pVTZ	2267.7	944.9	1500.0	394.3	251.0
B1B95	aug-cc-pVTZ	2276.7	949.7	1528.8	407.1	248.1
B3P86	aug-cc-pVTZ	2262.9	944.8	1532.8	405.0	248.1
B98	aug-cc-pVTZ	2237.9	936.0	1502.1	398.1	245.7
B971	aug-cc-pVTZ	2233.8	933.8	1504.5	392.3	244.8
B972	aug-cc-pVTZ	2261.2	943.5	1523.2	406.5	248.6
wB97X	aug-cc-pVTZ	2288.0	955.8	1460.9	419.4	253.0
wB97XD	aug-cc-pVTZ	2279.8	952.0	1489.7	413.8	251.2
CAM-B3LYP	aug-cc-pVTZ	2282.5	957.4	1486.9	427.8	251.2
M062X	aug-cc-pVTZ	2283.5	953.9	1489.1	408.3	251.7
SVWN	aug-cc-pVTZ	2225.9	926.3	1575.4	250.3	196.7
B3PW91	aug-cc-pVTZ	2256.5	942.2	1528.7	404.7	247.2
B3LYP	aug-cc-pVTZ	2239.3	939.1	1509.1	405.9	246.2
tHCTHhyb	def2SVPD	2253.3	936.0	1542.8	389.3	242.7
VSXC	def2SVPD	2235.0	930.4	1559.7	399.8	234.2
BMK	def2SVPD	2299.3	950.7	1522.5	399.3	251.6
B1B95	def2SVPD	2308.6	959.7	1562.1	408.7	247.7
B3P86	def2SVPD	2289.8	952.5	1560.8	407.7	246.8
B98	def2SVPD	2266.3	943.8	1531.1	401.8	246.4
B971	def2SVPD	2263.0	942.1	1534.4	397.0	245.0
B972	def2SVPD	2290.2	953.0	1553.2	402.4	246.2
wB97X	def2SVPD	2317.2	963.3	1484.2	422.4	250.6
wB97XD	def2SVPD	2308.4	958.9	1514.6	417.7	249.7
CAM-B3LYP	def2SVPD	2311.5	964.5	1512.5	433.6	252.9
M062X	def2SVPD	2313.4	963.3	1516.1	403.2	249.1
SVWN	def2SVPD	2262.9	935.6	1625.9	252.1	242.9
B3PW91	def2SVPD	2285.2	950.5	1557.6	406.3	246.7
B3LYP	def2SVPD	2267.8	946.7	1537.8	410.6	247.0
tHCTHhyb	def2TZVPD	2226.6	928.7	1514.6	392.0	244.1
VSXC	def2TZVPD	2208.6	922.1	1534.6	-934.4	297.5
BMK	def2TZVPD	2268.7	944.6	1497.5	404.3	252.9
B1B95	def2TZVPD	2278.5	951.0	1531.5	416.6	250.8
B3P86	def2TZVPD	2264.7	945.8	1534.9	412.4	249.7
B98	def2TZVPD	2239.6	936.6	1503.5	408.4	247.8
B971	def2TZVPD	2235.5	934.5	1506.0	402.5	246.8
B972	def2TZVPD	2262.4	944.6	1525.6	410.7	249.6
wB97X	def2TZVPD	2290.0	956.6	1463.2	423.5	255.2
wB97XD	def2TZVPD	2282.2	952.7	1492.5	429.5	252.7
CAM-B3LYP	def2TZVPD	2285.2	958.4	1488.6	435.2	254.4
M062X	def2TZVPD	2284.0	955.0	1492.1	413.6	251.7
SVWN	def2TZVPD	2230.8	928.4	1591.0	-164.7	287.9
B3PW91	def2TZVPD	2258.7	943.3	1531.0	411.4	249.3
B3LYP	def2TZVPD	2241.8	940.1	1511.5	414.3	248.5
tHCTHhyb	def2QZVPD	2223.3	927.8	1513.0	385.0	240.8
VSXC	def2QZVPD	2211.7	920.2	1507.7	-453.7	342.8
BMK	def2QZVPD	2263.6	943.7	1498.1	393.4	249.7
B1B95	def2QZVPD	2274.2	949.6	1528.8	400.7	247.4
B3P86	def2QZVPD	2260.7	944.4	1532.4	398.3	245.5
B98	def2QZVPD	2236.8	935.9	1502.8	393.0	244.7
B971	def2QZVPD	2232.5	933.6	1505.5	392.7	243.0
B972	def2QZVPD	2259.0	943.3	1523.4	400.5	246.8
wB97X	def2QZVPD	2285.5	954.8	1460.1	432.8	254.0
wB97XD	def2QZVPD	2278.2	951.5	1490.0	418.1	250.4
CAM-B3LYP	def2QZVPD	2280.5	956.8	1486.3	427.6	250.3
M062X	def2QZVPD	2280.9	953.9	1488.6	398.6	249.0
SVWN	def2QZVPD	2215.1	929.9	1596.7	292.3	65.8
B3PW91	def2QZVPD	2254.8	941.9	1528.9	397.3	245.1
B3LYP	def2QZVPD	2237.5	938.8	1509.3	400.7	243.6

Table S103: Harmonic unscaled vibrational frequencies (in cm^{-1}) for S_0 of C_2N_2

method	basis	1	2	3	4	5
tHCTHhyb	aug-cc-pVDZ	2403.5	879.4	2227.3	528.8	244.1
VSXC	aug-cc-pVDZ	2371.6	882.7	2205.0	537.9	246.8
BMK	aug-cc-pVDZ	2496.7	883.4	2312.9	527.4	247.1
B1B95	aug-cc-pVDZ	2475.6	894.9	2289.9	544.6	249.1
B3P86	aug-cc-pVDZ	2447.6	893.3	2265.6	541.9	247.9
B98	aug-cc-pVDZ	2429.9	882.9	2251.3	531.8	246.5
B971	aug-cc-pVDZ	2423.1	882.0	2244.5	530.9	245.6
B972	aug-cc-pVDZ	2451.4	891.3	2270.3	544.1	249.0
wB97X	aug-cc-pVDZ	2520.7	884.5	2319.6	545.4	249.5
wB97XD	aug-cc-pVDZ	2490.3	885.4	2294.9	541.4	249.0
CAM-B3LYP	aug-cc-pVDZ	2506.3	890.8	2314.4	547.2	250.6
M062X	aug-cc-pVDZ	2523.5	888.1	2333.6	546.2	249.3
SVWN	aug-cc-pVDZ	2386.5	897.6	2202.5	537.4	240.3
B3PW91	aug-cc-pVDZ	2442.6	890.8	2261.5	542.0	248.0
B3LYP	aug-cc-pVDZ	2429.3	885.7	2253.3	539.0	247.7
tHCTHhyb	aug-cc-pVTZ	2404.3	876.7	2242.5	543.6	247.9
VSXC	aug-cc-pVTZ	2366.9	878.5	2212.0	551.9	250.8
BMK	aug-cc-pVTZ	2503.4	876.3	2338.8	546.6	251.4
B1B95	aug-cc-pVTZ	2471.2	889.8	2298.3	557.6	252.9
B3P86	aug-cc-pVTZ	2445.6	890.1	2276.9	555.7	252.2
B98	aug-cc-pVTZ	2430.3	879.8	2265.8	546.1	250.5
B971	aug-cc-pVTZ	2422.5	878.5	2257.9	545.3	249.7
B972	aug-cc-pVTZ	2446.7	886.9	2277.8	558.1	252.8
wB97X	aug-cc-pVTZ	2518.1	880.6	2329.9	560.3	254.1
wB97XD	aug-cc-pVTZ	2490.3	882.7	2308.7	556.7	253.0
CAM-B3LYP	aug-cc-pVTZ	2505.1	887.4	2326.4	559.4	255.0
M062X	aug-cc-pVTZ	2519.3	883.3	2340.0	559.4	252.8
SVWN	aug-cc-pVTZ	2382.0	893.3	2215.5	545.7	243.8
B3PW91	aug-cc-pVTZ	2439.3	886.9	2271.9	555.6	251.9
B3LYP	aug-cc-pVTZ	2429.3	883.5	2266.5	552.2	252.5
tHCTHhyb	def2SVPD	2431.7	886.5	2264.2	542.8	249.7
VSXC	def2SVPD	2396.4	888.9	2238.2	546.9	250.8
BMK	def2SVPD	2527.7	890.1	2351.3	546.5	252.7
B1B95	def2SVPD	2502.8	900.9	2325.8	555.8	253.3
B3P86	def2SVPD	2473.0	899.0	2299.8	555.1	253.3
B98	def2SVPD	2457.3	889.6	2287.2	544.8	251.9
B971	def2SVPD	2450.5	888.8	2280.3	543.2	250.6
B972	def2SVPD	2477.7	896.9	2305.9	554.2	252.5
wB97X	def2SVPD	2545.2	889.2	2353.2	554.8	253.7
wB97XD	def2SVPD	2515.0	890.8	2328.7	553.1	253.5
CAM-B3LYP	def2SVPD	2532.2	896.1	2348.9	556.1	257.2
M062X	def2SVPD	2549.7	891.9	2370.2	555.4	253.2
SVWN	def2SVPD	2415.7	904.8	2239.5	562.2	255.4
B3PW91	def2SVPD	2468.3	896.4	2296.0	555.1	253.3
B3LYP	def2SVPD	2456.6	892.3	2289.0	549.8	254.4
tHCTHhyb	def2TZVPD	2405.5	878.1	2243.5	547.2	250.2
VSXC	def2TZVPD	2367.5	879.9	2213.1	557.1	254.4
BMK	def2TZVPD	2504.9	879.6	2336.4	551.3	252.9
B1B95	def2TZVPD	2472.9	891.3	2300.7	561.7	255.5
B3P86	def2TZVPD	2447.3	891.5	2278.8	560.6	254.9
B98	def2TZVPD	2431.3	881.1	2266.6	550.2	252.8
B971	def2TZVPD	2423.5	879.9	2258.6	549.8	252.2
B972	def2TZVPD	2448.2	888.1	2280.3	560.8	255.0
wB97X	def2TZVPD	2519.1	881.6	2331.3	564.8	257.1
wB97XD	def2TZVPD	2491.0	883.5	2309.7	561.2	256.1
CAM-B3LYP	def2TZVPD	2506.7	889.0	2327.9	565.3	258.2
M062X	def2TZVPD	2520.1	884.5	2341.5	560.5	254.1
SVWN	def2TZVPD	2386.4	895.4	2219.0	558.2	249.0
B3PW91	def2TZVPD	2441.2	888.4	2273.9	560.5	254.7
B3LYP	def2TZVPD	2431.2	885.1	2268.3	558.1	255.5
tHCTHhyb	def2QZVPD	2403.5	876.6	2242.7	540.6	247.3
VSXC	def2QZVPD	2363.7	877.4	2210.2	548.3	249.6
BMK	def2QZVPD	2502.7	877.3	2336.5	543.6	249.9
B1B95	def2QZVPD	2469.9	889.5	2298.7	554.4	252.1
B3P86	def2QZVPD	2444.4	889.7	2277.0	552.6	251.4
B98	def2QZVPD	2429.8	879.7	2266.4	543.8	250.1
B971	def2QZVPD	2421.9	878.4	2258.4	542.8	249.1
B972	def2QZVPD	2445.7	886.6	2278.4	554.6	252.0
wB97X	def2QZVPD	2516.0	879.7	2329.1	555.8	252.8
wB97XD	def2QZVPD	2488.8	881.8	2308.7	552.8	252.2
CAM-B3LYP	def2QZVPD	2503.8	887.2	2326.1	556.5	254.4
M062X	def2QZVPD	2517.8	883.1	2340.5	555.8	252.5
SVWN	def2QZVPD	2381.8	893.2	2216.6	543.4	242.9
B3PW91	def2QZVPD	2438.5	886.7	2272.2	552.5	251.2
B3LYP	def2QZVPD	2428.4	883.3	2266.6	549.4	251.8
gas[8]		2329.92	845.4	2157.84	502.83	233.15

Table S104: Harmonic unscaled vibrational frequencies (in cm^{-1}) for S_1 of C_4N_2

method	basis	1	2	3	4	5	6	7	8	9
tHCTHhyb	aug-cc-pVDZ	2246.5	1806.4	629.7	1897.8	1274.8	368.9	-67.0	413.7	91.8
VSXC	aug-cc-pVDZ	2319.9	1844.9	639.8	1949.1	1267.3	373.8	134.8	396.7	90.0
BMK	aug-cc-pVDZ	2202.6	1799.2	622.2	1798.4	1289.4	369.5	-177.6	421.5	91.9
B1B95	aug-cc-pVDZ	2273.6	1838.1	640.0	1887.4	1304.8	393.9	126.4	432.8	94.1
B3P86	aug-cc-pVDZ	2276.3	1833.9	639.5	1910.7	1296.9	387.3	97.1	427.4	94.7
B98	aug-cc-pVDZ	2248.7	1813.5	632.2	1878.7	1284.6	373.7	-74.4	418.8	92.8
B971	aug-cc-pVDZ	2247.9	1811.3	631.9	1880.3	1282.6	373.4	-34.1	417.7	93.2
B972	aug-cc-pVDZ	2274.7	1835.0	639.5	1905.4	1297.3	391.4	135.5	429.2	96.2
wB97X	aug-cc-pVDZ	2242.6	1848.5	632.6	1863.1	1301.9	397.8	106.9	433.5	89.6
wB97XD	aug-cc-pVDZ	2258.0	1843.7	635.1	1871.8	1302.7	388.7	48.1	428.4	90.6
CAM-B3LYP	aug-cc-pVDZ	2256.5	1847.6	636.5	1869.5	1306.2	395.7	23.2	436.4	93.8
M062X	aug-cc-pVDZ	2087.4	1777.6	628.3	1706.2	1307.1	394.0	106.3	434.1	93.4
SVWN	aug-cc-pVDZ	2217.1	1802.5	630.2	2003.0	1271.8	374.5	58.7	408.2	91.8
B3PW91	aug-cc-pVDZ	2270.9	1829.8	638.1	1905.2	1294.1	387.7	107.5	427.5	94.5
B3LYP	aug-cc-pVDZ	2251.5	1817.6	635.0	1885.0	1287.5	382.0	36.6	424.4	94.0
tHCTHhyb	aug-cc-pVTZ	2234.3	1811.4	630.5	1894.9	1276.6	257.6	438.1	437.7	102.5
VSXC	aug-cc-pVTZ	2245.3	1788.9	654.2	1946.9	1265.5	427.0	255.4	432.9	96.9
B1B95	aug-cc-pVTZ	2258.1	1838.4	638.6	1881.0	1302.6	458.5	265.6	453.3	103.9
B3P86	aug-cc-pVTZ	2262.5	1837.2	639.3	1907.1	1297.3	455.9	266.8	452.7	104.4
B971	aug-cc-pVTZ	2234.7	1815.4	632.1	1877.0	1283.3	436.3	257.8	440.6	104.2
B972	aug-cc-pVTZ	2260.4	1835.9	638.2	1900.3	1295.9	453.0	265.1	452.6	103.7
wB97X	aug-cc-pVTZ	2230.3	1848.9	631.5	1858.4	1298.8	450.5	256.1	452.7	103.4
wB97XD	aug-cc-pVTZ	2245.4	1846.2	634.8	1867.4	1302.0	258.2	449.9	449.4	99.2
CAM-B3LYP	aug-cc-pVTZ	2244.7	1850.8	636.6	1868.0	1305.6	460.7	259.3	456.5	96.2
M062X	aug-cc-pVTZ	2089.7	1780.8	627.3	1709.0	1303.5	460.9	259.1	455.4	91.9
SVWN	aug-cc-pVTZ	2250.8	1849.0	633.0	1999.1	1280.2	446.7	261.1	435.8	97.3
B3PW91	aug-cc-pVTZ	2255.9	1832.1	637.5	1900.4	1293.8	453.4	265.7	449.4	103.0
B3LYP	aug-cc-pVTZ	2238.8	1822.2	635.5	1883.1	1289.2	453.1	263.4	448.4	104.0
tHCTHhyb	def2SVPD	2260.5	1834.1	632.3	1932.0	1286.0	640.4	322.6	466.5	115.6
VSXC	def2SVPD	2243.1	1825.3	633.7	1980.0	1279.3	546.4	310.5	445.5	111.6
BMK	def2SVPD	2221.9	1828.9	626.2	1840.5	1299.2	687.2	339.3	490.7	123.2
B1B95	def2SVPD	2286.4	1864.2	641.4	1921.6	1313.5	626.4	322.9	474.4	113.3
B3P86	def2SVPD	2288.2	1859.5	641.3	1943.0	1306.4	652.7	326.3	478.3	116.5
B98	def2SVPD	2262.7	1840.9	634.5	1913.7	1294.9	638.1	326.5	472.9	117.1
B971	def2SVPD	2261.2	1838.3	634.1	1914.5	1292.9	623.9	322.9	467.4	115.3
B972	def2SVPD	2285.1	1860.1	640.8	1936.9	1306.3	609.7	318.4	466.6	111.9
wB97X	def2SVPD	2257.1	1870.8	632.7	1900.1	1305.4	597.5	317.4	473.1	108.9
wB97XD	def2SVPD	2271.5	1867.8	636.0	1907.3	1308.8	627.9	323.1	475.0	112.9
CAM-B3LYP	def2SVPD	2272.2	1873.1	637.8	1907.2	1313.0	646.0	333.3	489.3	117.6
M062X	def2SVPD	2103.5	1802.8	628.8	1744.8	1312.5	627.6	324.4	474.1	111.8
SVWN	def2SVPD	2311.0	1847.9	634.0	2055.5	1288.5	757.8	330.9	510.7	123.9
B3PW91	def2SVPD	2282.7	1855.3	639.7	1937.3	1303.4	646.1	324.4	475.5	115.9
B3LYP	def2SVPD	2265.9	1845.2	637.0	1920.1	1298.2	648.1	329.5	480.8	118.0
VSXC	def2TZVPD	2239.2	1835.7	619.2	2004.9	1284.8	430.9	257.9	373.5	-50.8
BMK	def2TZVPD	2194.1	1809.1	623.5	1804.7	1295.5	443.3	256.7	447.3	100.3
B1B95	def2TZVPD	2260.3	1840.6	639.1	1887.7	1307.5	458.0	264.3	450.0	96.3
B3P86	def2TZVPD	2265.2	1838.8	639.8	1914.5	1302.8	457.1	266.8	449.2	95.7
B98	def2TZVPD	2239.4	1819.8	633.0	1882.8	1290.5	442.1	257.6	440.7	98.4
B972	def2TZVPD	2261.1	1837.4	638.7	1904.8	1299.2	452.2	263.6	447.2	98.1
wB97X	def2TZVPD	2231.7	1850.9	631.9	1865.9	1303.3	461.4	256.9	457.5	94.9
CAM-B3LYP	def2TZVPD	2248.2	1852.9	637.0	1876.9	1312.6	465.0	262.3	460.6	108.4
M062X	def2TZVPD	2092.6	1783.3	627.4	1715.6	1307.7	458.1	261.1	452.7	97.1
SVWN	def2TZVPD	2190.8	1839.0	580.2	1993.2	1281.4	456.0	265.7	422.7	94.8
B3PW91	def2TZVPD	2258.8	1833.9	638.0	1907.9	1299.3	455.3	266.1	449.5	96.5
B3LYP	def2TZVPD	2242.1	1824.1	635.9	1891.6	1295.4	455.3	264.7	447.9	96.3
tHCTHhyb	def2QZVPD	2233.4	1810.6	630.6	1893.8	1276.3	435.7	255.5	433.7	97.5
VSXC	def2QZVPD	2281.6	1850.0	642.5	1957.0	1252.0	436.8	263.1	400.7	78.9
BMK	def2QZVPD	2187.1	1807.2	623.4	1794.4	1289.3	445.0	265.6	441.7	106.8
B1B95	def2QZVPD	2256.1	1838.2	638.4	1879.6	1302.3	455.7	263.1	448.1	105.1
B3P86	def2QZVPD	2261.6	1836.6	639.3	1905.7	1296.9	452.1	262.2	447.2	94.4
B98	def2QZVPD	2236.5	1818.5	632.6	1875.9	1285.9	257.0	438.5	437.6	95.7
B971	def2QZVPD	2234.1	1815.2	632.1	1876.3	1283.3	439.2	257.3	436.4	96.1
B972	def2QZVPD	2258.4	1834.9	638.3	1898.7	1295.5	449.2	263.4	445.4	96.2
wB97X	def2QZVPD	2227.8	1848.1	630.8	1857.7	1297.3	448.5	252.4	452.5	79.4
wB97XD	def2QZVPD	2244.2	1846.8	634.2	1867.6	1301.0	446.2	253.4	447.8	82.8
CAM-B3LYP	def2QZVPD	2243.7	1849.9	635.8	1867.3	1305.0	462.8	257.5	458.6	82.0
M062X	def2QZVPD	2088.7	1781.8	627.0	1709.1	1303.4	260.4	461.3	449.3	104.4
SVWN	def2QZVPD	2262.6	1814.8	634.8	1972.7	1277.4	450.5	260.5	435.1	97.8
B3PW91	def2QZVPD	2254.9	1831.3	637.5	1899.2	1293.4	452.2	264.2	445.8	97.3
B3LYP	def2QZVPD	2238.5	1821.3	635.4	1881.9	1288.8	449.7	262.2	445.6	96.3
gas[11]		n/a	n/a	n/a	n/a	n/a	422.0	223.2	339.8	86.9
Kr[12]		n/a	n/a	n/a	n/a	n/a	466.0	234	n/a	n/a

Table S105: Harmonic unscaled vibrational frequencies (in cm^{-1}) for S_2 of C_4N_2

method	basis	1	2	3	4	5	6	7	8	9
tHCTHhyb	aug-cc-pVDZ	2262.4	1825.0	629.1	1943.7	1271.6	386.2	122.1	424.2	95.1
VSXC	aug-cc-pVDZ	2254.0	1814.9	632.6	1962.8	1266.8	409.8	189.6	399.1	-64.5
BMK	aug-cc-pVDZ	2289.4	1861.0	627.0	1934.5	1285.6	386.2	-78.7	431.0	92.9
B1B95	aug-cc-pVDZ	2311.0	1872.3	641.6	1966.8	1301.1	414.1	178.3	442.2	99.0
B3P86	aug-cc-pVDZ	2298.0	1857.2	639.5	1967.6	1293.6	406.5	162.6	438.5	96.9
B98	aug-cc-pVDZ	2272.0	1836.8	632.1	1935.3	1281.3	393.3	127.5	432.2	97.3
B971	aug-cc-pVDZ	2270.3	1834.7	631.8	1936.9	1279.3	392.7	138.3	430.1	96.8
B972	aug-cc-pVDZ	2296.6	1857.8	639.4	1961.8	1294.0	412.1	185.8	440.8	98.3
wB97X	aug-cc-pVDZ	2304.0	1885.1	634.8	1941.5	1298.5	427.8	189.8	448.7	95.7
wB97XD	aug-cc-pVDZ	2304.8	1875.4	636.2	1944.5	1298.7	414.6	165.0	443.7	95.6
CAM-B3LYP	aug-cc-pVDZ	2303.0	1879.7	638.2	1942.6	1302.9	421.6	157.9	450.9	96.8
M062X	aug-cc-pVDZ	2316.8	1895.7	641.1	1932.3	1301.8	415.6	168.5	444.6	98.4
SVWN	aug-cc-pVDZ	2282.4	1835.0	635.3	2017.1	1271.3	417.5	113.1	472.8	98.3
B3PW91	aug-cc-pVDZ	2293.0	1853.6	638.1	1963.0	1290.8	407.1	168.6	438.5	97.6
B3LYP	aug-cc-pVDZ	2273.0	1839.9	634.8	1939.7	1284.4	401.9	142.6	436.3	96.7
VSXC	aug-cc-pVTZ	2230.8	1811.7	630.4	1969.0	1259.5	516.9	271.9	394.0	139.2
B1B95	aug-cc-pVTZ	2294.0	1870.9	640.3	1957.8	1298.9	487.5	276.3	463.8	105.8
B3P86	aug-cc-pVTZ	2283.8	1858.9	639.3	1962.2	1293.8	484.2	276.0	461.5	108.4
B98	aug-cc-pVTZ	2259.4	1840.6	632.6	1931.3	1282.2	475.9	270.9	457.0	107.3
B972	aug-cc-pVTZ	2281.7	1857.6	638.1	1955.0	1292.5	482.2	275.0	461.0	109.1
wB97X	aug-cc-pVTZ	2288.0	1883.7	633.6	1933.7	1295.5	501.2	276.0	473.1	103.5
wB97XD	aug-cc-pVTZ	2290.0	1876.2	635.9	1938.0	1297.7	493.6	276.1	470.4	102.4
CAM-B3LYP	aug-cc-pVTZ	2288.6	1881.2	638.1	1938.3	1302.2	502.8	276.8	472.4	103.5
M062X	aug-cc-pVTZ	2299.3	1893.0	638.9	1923.6	1297.8	495.3	275.3	465.2	102.9
SVWN	aug-cc-pVTZ	2263.3	1830.0	636.4	2009.2	1294.1	358.9	214.1	559.3	-36.8
B3PW91	aug-cc-pVTZ	2277.3	1854.4	637.6	1956.5	1290.3	482.6	276.0	458.2	108.2
B3LYP	aug-cc-pVTZ	2259.5	1842.9	635.2	1936.2	1285.8	483.2	275.5	458.0	106.5
tHCTHhyb	def2SVPD	2276.8	1852.5	631.8	1978.8	1282.4	663.1	322.3	475.6	116.5
VSXC	def2SVPD	2262.3	1838.3	632.9	1991.3	1276.1	651.5	282.0	549.8	122.0
BMK	def2SVPD	2308.4	1890.1	631.1	1977.3	1295.6	714.6	339.9	497.2	124.8
B1B95	def2SVPD	2323.9	1898.0	643.3	2001.3	1309.9	653.3	323.6	483.8	115.8
B3P86	def2SVPD	2310.4	1882.5	641.4	2000.6	1302.8	676.2	326.4	487.3	117.5
B98	def2SVPD	2286.4	1864.0	634.5	1971.4	1291.3	662.3	326.1	481.5	117.4
B971	def2SVPD	2284.2	1861.5	634.2	1972.2	1289.3	648.3	322.8	477.0	116.6
B972	def2SVPD	2307.5	1882.7	640.8	1993.9	1302.6	635.9	319.1	475.8	113.8
wB97X	def2SVPD	2316.6	1907.3	635.1	1977.5	1302.7	636.2	320.5	487.1	112.7
wB97XD	def2SVPD	2317.9	1899.4	637.2	1980.4	1305.0	660.5	324.1	487.5	115.3
CAM-B3LYP	def2SVPD	2318.0	1905.0	639.6	1979.9	1310.2	678.5	334.6	501.0	119.9
M062X	def2SVPD	2329.1	1921.2	642.3	1968.3	1309.3	658.7	326.2	483.9	116.1
SVWN	def2SVPD	2301.3	1864.0	638.3	2054.0	1284.9	823.3	333.6	395.2	76.7
B3PW91	def2SVPD	2305.1	1878.8	639.9	1995.9	1299.8	670.0	324.6	484.7	116.8
B3LYP	def2SVPD	2287.7	1867.0	636.9	1975.5	1294.7	671.8	329.8	489.9	119.0
tHCTHhyb	def2TZVPD	2252.5	1830.4	629.9	1946.0	1277.6	464.8	269.0	447.3	94.4
VSXC	def2TZVPD	2241.3	1817.0	631.7	1973.1	1271.7	293.1	-162.4	364.1	-553.8
BMK	def2TZVPD	2278.2	1868.9	628.3	1936.8	1292.3	478.4	274.5	460.8	104.4
B1B95	def2TZVPD	2296.5	1873.4	640.8	1965.0	1304.0	486.3	277.4	465.6	106.2
B3P86	def2TZVPD	2286.6	1861.2	639.8	1969.9	1299.4	484.7	276.7	459.8	99.9
B98	def2TZVPD	2262.1	1842.0	632.8	1938.1	1286.9	471.9	272.1	452.8	89.7
B971	def2TZVPD	2259.2	1838.9	632.3	1938.5	1284.3	469.3	270.4	450.6	98.9
B972	def2TZVPD	2282.8	1859.5	638.7	1960.0	1295.8	480.7	275.0	460.7	94.9
wB97X	def2TZVPD	2290.2	1885.9	634.1	1941.8	1300.3	497.2	277.9	471.4	105.3
wB97XD	def2TZVPD	2293.0	1878.8	636.1	1946.7	1302.8	487.9	275.6	462.6	100.9
CAM-B3LYP	def2TZVPD	2292.5	1883.3	638.5	1947.4	1309.5	503.4	280.1	472.9	101.4
M062X	def2TZVPD	2302.7	1895.1	639.7	1928.1	1303.3	489.9	277.4	464.3	105.7
SVWN	def2TZVPD	2269.5	1839.0	636.2	2029.8	1287.5	402.2	267.5	318.2	-161.8
B3PW91	def2TZVPD	2280.4	1856.6	638.1	1964.3	1296.0	482.7	276.2	459.3	100.3
B3LYP	def2TZVPD	2263.2	1845.3	635.7	1945.0	1292.1	483.5	275.7	460.3	96.3
tHCTHhyb	def2QZVPD	2248.7	1828.7	629.3	1938.3	1272.8	461.4	267.2	444.7	102.4
VSXC	def2QZVPD	2259.4	1784.3	620.9	1922.6	1241.9	553.3	220.8	389.3	125.7
BMK	def2QZVPD	2271.8	1867.0	628.1	1925.8	1284.8	477.4	277.5	456.1	102.8
B1B95	def2QZVPD	2292.5	1871.1	639.9	1956.6	1298.5	478.6	276.1	452.3	100.8
B3P86	def2QZVPD	2282.4	1859.1	639.1	1961.0	1293.5	479.6	275.4	456.6	97.0
B98	def2QZVPD	2259.1	1840.3	632.6	1930.8	1282.3	466.8	267.6	451.3	100.0
B972	def2QZVPD	2280.0	1857.5	637.7	1953.6	1292.2	478.2	273.6	456.8	101.5
wB97X	def2QZVPD	2286.1	1883.1	632.9	1933.1	1294.5	491.1	274.6	467.6	105.5
wB97XD	def2QZVPD	2288.6	1875.6	635.4	1937.9	1297.0	482.8	271.8	461.7	102.1
CAM-B3LYP	def2QZVPD	2287.6	1880.8	637.8	1937.5	1301.6	496.5	277.2	468.4	99.9
M062X	def2QZVPD	2297.6	1892.3	639.1	1922.1	1298.6	492.4	277.5	465.4	111.1
B3PW91	def2QZVPD	2276.4	1854.0	637.4	1955.3	1289.9	476.8	273.6	456.6	96.1
B3LYP	def2QZVPD	2258.9	1842.5	635.0	1935.3	1285.4	478.0	274.7	453.8	109.3
gas[13]		2192	n/a	591	n/a	n/a	458	260	443	99
Kr[12]		2191	n/a	n/a	n/a	n/a	461	267	n/a	n/a

Table S106: Harmonic unscaled vibrational frequencies (in cm^{-1}) for S_0 of C_4N_2

method	basis	1	2	3	4	5	6	7	8	9
tHCTHhyb	aug-cc-pVDZ	2356.0	2179.4	615.4	2309.5	1189.6	445.6	176.1	486.8	107.7
VSXC	aug-cc-pVDZ	2323.6	2159.0	620.3	2283.9	1199.5	463.3	218.4	497.7	109.8
BMK	aug-cc-pVDZ	2435.4	2238.9	612.9	2394.7	1186.1	441.7	108.1	487.6	109.1
B1B95	aug-cc-pVDZ	2430.8	2242.6	624.1	2377.5	1204.2	480.0	212.7	501.0	109.8
B3P86	aug-cc-pVDZ	2402.4	2219.4	624.1	2351.6	1205.2	469.7	201.0	498.2	109.3
B98	aug-cc-pVDZ	2383.6	2204.4	616.7	2334.2	1190.6	450.8	178.7	489.7	108.7
B971	aug-cc-pVDZ	2378.2	2198.5	616.5	2328.0	1190.1	451.8	185.1	489.3	108.6
B972	aug-cc-pVDZ	2406.0	2224.3	623.2	2355.8	1202.5	478.2	217.0	501.5	109.9
wB97X	aug-cc-pVDZ	2503.5	2284.1	614.7	2420.1	1179.6	499.8	224.4	500.9	110.6
wB97XD	aug-cc-pVDZ	2469.2	2258.7	616.4	2391.8	1185.2	478.3	203.2	497.4	109.9
CAM-B3LYP	aug-cc-pVDZ	2475.2	2273.1	619.3	2406.9	1190.8	485.2	202.4	501.0	110.1
M062X	aug-cc-pVDZ	2484.7	2285.2	617.9	2423.3	1188.2	486.5	206.8	501.4	109.3
SVWN	aug-cc-pVDZ	2340.9	2152.0	627.2	2292.1	1218.8	453.9	173.5	488.6	105.6
B3PW91	aug-cc-pVDZ	2397.3	2215.4	622.4	2346.9	1201.8	471.0	204.9	498.5	109.4
B3LYP	aug-cc-pVDZ	2383.8	2208.3	618.9	2334.9	1193.9	462.9	190.1	494.9	109.2
tHCTHhyb	aug-cc-pVTZ	2351.7	2193.1	614.2	2318.5	1183.7	544.4	278.5	504.3	112.7
VSXC	aug-cc-pVTZ	2314.2	2165.1	617.7	2286.2	1192.2	539.3	281.8	513.6	114.2
BMK	aug-cc-pVTZ	2446.0	2272.6	609.7	2413.5	1172.1	567.3	284.0	507.0	113.5
B1B95	aug-cc-pVTZ	2421.8	2249.0	621.3	2380.8	1195.7	568.1	284.4	516.9	114.3
B3P86	aug-cc-pVTZ	2395.0	2228.4	622.4	2357.2	1198.8	563.1	283.8	515.1	114.2
B98	aug-cc-pVTZ	2379.7	2217.1	615.4	2342.8	1184.5	550.7	281.1	507.4	113.6
B971	aug-cc-pVTZ	2373.1	2210.1	614.8	2335.5	1183.6	548.5	280.0	506.9	113.4
B972	aug-cc-pVTZ	2396.4	2229.7	620.6	2358.6	1195.0	561.3	283.8	517.1	114.3
wB97X	aug-cc-pVTZ	2495.6	2290.6	612.6	2425.1	1173.0	585.4	285.2	518.9	115.0
wB97XD	aug-cc-pVTZ	2462.8	2268.2	615.2	2399.6	1179.9	574.1	283.2	515.2	114.7
CAM-B3LYP	aug-cc-pVTZ	2469.4	2282.4	617.8	2413.8	1184.6	584.3	287.6	518.3	115.0
M062X	aug-cc-pVTZ	2478.4	2290.8	615.8	2426.1	1180.9	579.5	283.3	518.1	113.9
SVWN	aug-cc-pVTZ	2327.6	2162.5	625.1	2296.4	1211.1	552.1	277.8	502.5	110.3
B3PW91	aug-cc-pVTZ	2388.7	2223.5	620.4	2351.6	1194.7	562.1	283.5	514.7	114.1
B3LYP	aug-cc-pVTZ	2377.9	2218.9	617.9	2342.7	1188.7	561.4	284.3	512.5	114.3
tHCTHhyb	def2SVPD	2368.6	2204.2	617.4	2344.6	1195.6	675.8	315.1	514.4	124.2
VSXC	def2SVPD	2329.3	2180.4	620.9	2313.9	1202.7	628.7	308.1	511.0	120.3
BMK	def2SVPD	2457.9	2264.5	616.5	2434.1	1194.2	708.5	326.2	522.5	130.5
B1B95	def2SVPD	2442.4	2264.6	625.0	2411.0	1208.2	671.1	314.3	522.7	123.7
B3P86	def2SVPD	2412.6	2241.5	625.2	2383.8	1209.6	688.1	318.1	524.9	124.9
B98	def2SVPD	2396.0	2227.9	618.6	2368.8	1196.5	675.3	317.6	516.9	125.4
B971	def2SVPD	2390.0	2222.0	618.2	2362.2	1195.8	663.8	314.5	514.5	124.2
B972	def2SVPD	2415.9	2246.7	623.8	2388.5	1205.4	658.8	310.7	519.1	122.2
wB97X	def2SVPD	2512.2	2304.1	614.7	2451.3	1182.1	670.5	309.9	521.6	123.0
wB97XD	def2SVPD	2478.0	2279.6	617.0	2423.3	1188.8	681.0	312.6	521.3	124.9
CAM-B3LYP	def2SVPD	2487.1	2294.7	620.3	2440.7	1195.9	693.6	323.7	528.7	127.3
M062X	def2SVPD	2496.4	2306.7	617.9	2457.1	1190.5	672.8	313.5	521.7	125.4
SVWN	def2SVPD	2355.1	2179.3	629.3	2328.1	1225.9	783.9	330.1	541.7	128.4
B3PW91	def2SVPD	2407.4	2237.6	623.4	2379.3	1205.9	684.1	316.4	523.6	124.3
B3LYP	def2SVPD	2395.9	2232.1	620.5	2369.6	1200.0	684.6	322.9	524.2	126.5
tHCTHhyb	def2TZVPD	2352.8	2193.2	614.9	2319.5	1185.8	543.6	280.8	504.0	113.1
VSXC	def2TZVPD	2313.9	2165.3	618.7	2287.1	1194.5	543.3	285.3	513.8	115.2
BMK	def2TZVPD	2445.6	2266.0	612.1	2413.0	1178.0	567.9	287.4	506.9	114.3
B1B95	def2TZVPD	2422.9	2250.5	622.2	2382.7	1197.9	568.9	287.6	516.4	114.9
B3P86	def2TZVPD	2396.2	2229.6	623.1	2359.0	1200.8	563.3	286.9	514.7	114.7
B98	def2TZVPD	2380.2	2217.0	616.1	2343.6	1186.5	551.0	283.5	507.1	114.1
B971	def2TZVPD	2373.4	2209.9	615.6	2336.2	1185.6	548.8	282.5	506.4	113.8
B972	def2TZVPD	2397.2	2231.4	621.3	2360.4	1196.7	560.3	286.0	516.2	114.7
wB97X	def2TZVPD	2495.5	2291.6	613.2	2426.2	1174.6	586.2	289.2	517.9	115.7
wB97XD	def2TZVPD	2462.9	2269.1	615.6	2400.3	1181.0	573.1	286.6	514.5	115.5
CAM-B3LYP	def2TZVPD	2470.4	2283.3	618.6	2415.3	1186.8	585.9	291.3	518.3	115.8
M062X	def2TZVPD	2478.9	2291.5	616.2	2427.3	1182.4	577.4	285.7	516.4	113.9
SVWN	def2TZVPD	2332.8	2165.8	626.3	2300.0	1213.3	561.6	282.8	506.6	112.0
B3PW91	def2TZVPD	2390.1	2224.9	621.1	2353.5	1196.8	562.4	286.4	514.4	114.6
B3LYP	def2TZVPD	2379.3	2220.1	618.6	2344.4	1190.8	562.6	287.5	512.6	115.0
tHCTHhyb	def2QZVPD	2349.1	2191.6	614.2	2318.1	1184.2	541.1	278.4	501.2	111.9
VSXC	def2QZVPD	2309.5	2161.9	617.3	2283.5	1191.9	536.9	281.8	509.2	113.3
BMK	def2QZVPD	2441.9	2265.6	610.7	2412.7	1175.4	567.0	286.5	504.2	113.0
B1B95	def2QZVPD	2418.7	2247.7	621.3	2380.1	1196.2	565.0	284.7	513.3	113.6
B3P86	def2QZVPD	2392.1	2227.0	622.3	2356.6	1199.1	559.7	283.9	511.4	113.3
B98	def2QZVPD	2377.3	2216.0	615.4	2342.8	1185.1	548.4	281.4	504.4	113.0
B971	def2QZVPD	2370.4	2208.8	614.8	2335.3	1184.2	546.1	280.4	503.5	112.7
B972	def2QZVPD	2393.8	2228.9	620.5	2358.2	1195.2	558.5	283.9	513.7	113.6
wB97X	def2QZVPD	2491.4	2288.3	612.2	2423.4	1172.7	580.4	285.3	513.7	114.0
wB97XD	def2QZVPD	2459.5	2266.7	614.7	2398.6	1179.5	568.4	282.7	511.0	113.8
CAM-B3LYP	def2QZVPD	2466.1	2280.3	617.6	2412.9	1185.0	580.4	288.0	514.4	114.2
M062X	def2QZVPD	2475.2	2289.4	615.7	2425.5	1181.5	574.9	284.2	514.1	113.2
SVWN	def2QZVPD	2325.7	2162.2	625.1	2296.3	1210.8	549.9	277.1	499.9	109.4
B3PW91	def2QZVPD	2386.1	2222.3	620.3	2351.2	1195.1	558.7	283.3	511.3	113.2
B3LYP	def2QZVPD	2375.1	2217.4	617.8	2342.1	1189.1	558.3	284.6	509.0	113.5
Kr[12]		n/a	n/a	617	n/a	n/a	508	247	n/a	n/a

Table S107: Harmonic unscaled vibrational frequencies (in cm^{-1}) for S_1 of C_6N_2

method	basis	1	2	3	4	5	6	7	8	9	10	11	12	13
tHCTHhyb	aug-cc-pVDZ	2216.0	2071.2	1441.5	484.4	1989.0	1870.0	943.6	424.3	133.8	-1013.9	407.8	57.6	-61.3
VSXC	aug-cc-pVDZ	2218.2	2066.5	1442.5	488.3	2037.1	1860.6	961.9	418.6	129.7	-784.4	415.2	170.5	57.3
BMK	aug-cc-pVDZ	2156.4	2077.4	1449.6	480.3	1997.5	1776.9	935.6	432.5	140.9	-1279.4	410.9	59.4	-160.4
B1B95	aug-cc-pVDZ	2234.8	2112.9	1469.8	492.4	2016.9	1867.5	957.9	442.4	141.9	-925.4	429.9	128.6	56.4
B3P86	aug-cc-pVDZ	2242.9	2103.8	1464.2	491.8	2012.7	1888.3	957.4	438.0	136.5	-983.6	423.4	87.6	58.1
B98	aug-cc-pVDZ	2214.7	2081.8	1449.6	486.3	1988.3	1862.2	946.6	429.6	133.1	-1037.4	412.6	56.0	-65.4
B971	aug-cc-pVDZ	2214.8	2078.7	1448.1	486.1	1985.7	1864.6	946.1	428.9	135.0	-999.7	412.4	55.9	29.6
B972	aug-cc-pVDZ	2239.8	2105.0	1464.2	492.0	2011.4	1884.4	957.1	441.0	137.4	-848.9	429.4	144.1	58.3
wB97X	aug-cc-pVDZ	2193.4	2099.7	1461.2	489.0	2070.7	1745.5	943.6	443.6	134.8	-890.9	433.7	134.1	56.7
wB97XD	aug-cc-pVDZ	2201.8	2118.4	1463.1	489.5	2040.7	1799.1	948.0	438.4	133.1	-974.9	425.7	73.0	56.5
CAM-B3LYP	aug-cc-pVDZ	2206.5	2122.3	1466.9	490.9	2048.8	1801.3	950.7	444.7	136.8	-1065.6	431.1	60.3	41.4
M062X	aug-cc-pVDZ	2083.7	1914.6	1469.4	485.4	1982.0	1674.2	940.3	440.6	133.7	-953.8	428.1	101.3	57.9
SVWN	aug-cc-pVDZ	2259.9	2104.4	1448.3	487.1	2095.8	1892.7	955.0	423.8	139.3	-1123.6	408.0	59.4	-50.6
B3PW91	aug-cc-pVDZ	2237.3	2099.1	1460.9	490.8	2007.5	1883.5	955.1	438.2	136.3	-955.5	424.1	102.0	58.6
B3LYP	aug-cc-pVDZ	2218.1	2084.3	1453.4	488.4	1990.1	1870.0	950.1	435.0	135.7	-1044.5	419.0	58.1	14.2
tHCTHhyb	aug-cc-pVTZ	2200.1	2073.4	1440.7	485.3	1988.5	1864.1	943.8	485.8	444.5	152.0	468.1	289.3	66.1
VSXC	aug-cc-pVTZ	2183.4	2064.9	1424.4	475.9	2080.9	1897.3	940.9	461.0	428.2	147.5	463.1	289.7	61.0
BMK	aug-cc-pVTZ	2151.0	2080.9	1446.7	482.0	2016.4	1754.1	934.1	529.6	456.1	149.2	479.9	297.2	63.9
B1B95	aug-cc-pVTZ	2216.6	2110.3	1464.8	491.7	2017.6	1852.7	955.0	508.9	458.1	150.2	485.6	296.6	65.5
B3P86	aug-cc-pVTZ	2225.1	2103.8	1461.7	492.0	2013.1	1878.0	956.4	511.3	457.5	151.8	484.9	296.9	65.8
B98	aug-cc-pVTZ	2200.1	2084.0	1448.4	487.2	1991.6	1853.9	946.5	485.7	447.8	149.4	471.0	290.1	63.5
B971	aug-cc-pVTZ	2198.7	2079.9	1446.2	486.6	1987.4	1855.1	945.6	482.9	447.6	150.9	468.7	289.1	63.7
B972	aug-cc-pVTZ	2222.4	2103.1	1460.0	491.2	2011.1	1872.8	954.5	502.0	456.9	152.8	482.2	294.4	64.2
wB97X	aug-cc-pVTZ	2189.9	2088.7	1456.8	488.7	2074.1	1730.9	941.8	518.5	463.7	148.3	490.1	292.7	53.3
wB97XD	aug-cc-pVTZ	2191.9	2114.6	1460.6	489.9	2044.9	1787.5	947.8	516.3	461.6	147.5	486.1	292.1	58.5
CAM-B3LYP	aug-cc-pVTZ	2196.6	2117.6	1463.5	491.2	2054.8	1787.4	949.9	518.0	462.4	145.8	489.5	293.0	51.7
M062X	aug-cc-pVTZ	2090.8	1929.7	1462.8	485.2	1992.4	1663.2	937.6	525.1	460.8	146.4	486.4	292.7	58.2
SVWN	aug-cc-pVTZ	2227.3	2082.4	1447.0	488.8	2045.5	1887.2	954.0	508.2	442.1	148.7	476.2	292.8	59.6
B3PW91	aug-cc-pVTZ	2218.4	2098.1	1457.6	490.7	2007.1	1872.2	953.7	507.2	455.2	151.2	483.6	296.1	65.1
B3LYP	aug-cc-pVTZ	2200.9	2085.6	1452.0	489.1	1992.8	1860.3	950.1	500.6	454.4	150.3	480.3	294.2	64.5
tHCTHhyb	def2SVPD	2224.7	2099.9	1447.7	487.1	2026.4	1874.3	948.1	995.2	478.5	182.8	585.2	348.2	66.1
VSXC	def2SVPD	2221.0	2090.8	1446.1	488.8	2043.6	1889.9	952.1	877.7	458.6	174.4	526.4	332.5	65.5
BMK	def2SVPD	2179.4	2099.3	1455.6	484.7	2041.3	1779.6	941.8	1061.8	500.9	196.4	631.5	368.9	70.7
B1B95	def2SVPD	2243.6	2139.6	1473.8	494.2	2057.9	1866.6	960.5	947.9	482.6	178.1	578.2	345.1	66.3
B3P86	def2SVPD	2249.8	2130.3	1468.8	493.8	2049.8	1889.3	960.7	1007.0	488.3	183.8	599.1	352.2	67.4
B98	def2SVPD	2224.1	2110.1	1455.1	488.9	2028.5	1864.0	950.6	975.2	482.1	184.2	585.3	350.6	67.1
B971	def2SVPD	2223.3	2106.7	1453.5	488.5	2025.2	1865.6	950.0	950.7	478.0	181.3	575.6	346.7	66.1
B972	def2SVPD	2245.2	2131.1	1468.1	493.4	2049.9	1882.7	959.3	936.6	475.0	174.1	561.0	338.9	65.5
wB97X	def2SVPD	2221.9	2106.3	1462.7	490.1	2109.7	1743.7	944.7	923.9	483.1	174.1	568.8	341.9	64.2
wB97XD	def2SVPD	2219.5	2135.5	1465.9	491.1	2079.6	1798.7	950.2	978.9	483.6	178.6	580.5	346.4	64.9
CAM-B3LYP	def2SVPD	2226.5	2140.2	1470.2	493.0	2089.6	1802.8	953.6	970.6	499.5	189.1	607.3	359.8	67.6
M062X	def2SVPD	2120.0	1917.9	1470.6	487.0	2024.6	1671.3	941.5	926.2	479.1	175.4	572.9	345.7	64.2
SVWN	def2SVPD	2260.8	2116.0	1459.1	488.8	2133.8	1919.4	955.2	1259.2	524.1	204.0	700.2	369.5	70.6
B3PW91	def2SVPD	2244.0	2125.5	1465.4	492.6	2044.9	1884.0	958.3	1003.5	485.2	181.8	591.9	349.5	67.2
B3LYP	def2SVPD	2227.3	2113.0	1459.0	490.8	2030.4	1872.6	954.3	974.4	492.9	188.7	605.8	357.4	67.8
tHCTHhyb	def2TZVPD	2203.4	2075.2	1441.6	485.2	1991.9	1865.4	943.9	469.7	439.9	144.4	463.6	286.9	58.8
VSXC	def2TZVPD	2202.8	2066.6	1439.6	487.2	2013.2	1878.7	948.2	446.3	394.1	132.9	461.4	289.1	60.3
BMK	def2TZVPD	2154.4	2082.7	1448.6	482.0	2015.3	1761.5	935.3	491.0	453.6	147.5	468.6	286.6	57.8
B1B95	def2TZVPD	2219.0	2112.7	1466.2	492.0	2021.2	1854.1	955.5	490.0	451.7	151.7	481.3	295.0	60.0
B3P86	def2TZVPD	2228.1	2106.0	1462.8	491.9	2016.7	1879.5	956.6	490.9	451.3	148.9	479.0	294.2	59.9
B98	def2TZVPD	2202.9	2085.8	1449.3	487.1	1994.6	1854.8	946.6	475.0	442.7	148.4	467.3	288.4	57.7
B971	def2TZVPD	2201.5	2081.4	1446.9	486.5	1990.3	1855.7	945.5	471.9	441.8	148.6	465.7	287.8	60.6
B972	def2TZVPD	2223.4	2105.0	1461.1	491.5	2014.2	1872.7	954.9	442.3	149.3	476.5	475.7	292.7	61.6
wB97X	def2TZVPD	2192.3	2089.3	1457.2	488.6	2077.5	1731.2	941.8	496.1	457.4	147.7	485.5	289.1	64.5
wB97XD	def2TZVPD	2194.3	2116.4	1461.1	489.9	2048.7	1787.9	947.6	487.4	454.0	146.1	478.0	288.8	66.4
CAM-B3LYP	def2TZVPD	2200.1	2120.2	1464.6	491.3	2058.0	1790.3	950.0	504.6	460.5	150.7	487.9	292.6	73.6
M062X	def2TZVPD	2094.3	1933.2	1464.3	485.4	1996.7	1665.9	938.3	502.2	455.3	147.7	482.6	289.9	62.4
SVWN	def2TZVPD	2222.7	2087.1	1446.6	485.4	2063.6	1881.8	953.2	518.2	437.6	147.7	474.7	292.8	59.9
B3PW91	def2TZVPD	2221.7	2100.5	1458.8	490.5	2010.9	1873.6	953.9	488.4	450.0	150.4	477.3	293.4	61.8
B3LYP	def2TZVPD	2204.5	2088.2	1453.1	489.0	1996.7	1862.0	950.2	492.8	451.2	151.4	476.2	292.5	61.0
tHCTHhyb	def2QZVPD	2199.2	2072.6	1440.2	485.0	1988.2	1861.7	943.1	465.2	429.6	147.6	463.3	285.3	64.3
VSXC	def2QZVPD	2202.5	2075.1	1439.1	481.5	2020.9	1877.3	953.7	459.0	414.2	138.6	457.8	289.3	60.6
BMK	def2QZVPD	2149.5	2077.6	1447.2	482.2	2013.5	1750.9	935.4	488.0	447.7	146.8	472.2	296.6	61.1
B1B95	def2QZVPD	2215.0	2109.2	1464.6	491.8	2017.5	1850.5	955.0	481.7	443.6	149.6	477.5	292.6	62.5
B3P86	def2QZVPD	2224.7	2102.5	1461.5	491.7	2012.8	1875.3	955.9	481.6	443.4	147.3	477.5	291.8	67.0
B98	def2QZVPD	2198.4	2083.6	1448.4	487.0	1991.8	1851.5	946.6	467.1	435.7	148.0	465.0	287.2	70.5
B971	def2QZVPD	2198.1	2078.8	1446.2	486.6	1987.7	1852.1	945.2	467.0	433.0	147.8	463.3	285.7	69.4
B972	def2QZVPD	2220.7	2101.5	1459.6	491.3	2010.8	1869.4	953.7	479.3	442.6	147.0	477.1	291.8	69.1
wB97X	def2QZVPD	2188.9	2086.3	1455.6	488.4	2073.8	1726.6	940.4	486.4	444.9	135.7	482.9	285.5	65.1
wB97XD	def2QZVPD	2191.3	2113.7	1459.7	489.5	2045.5	1783.4	946.8	480.0	435.1	136.3	475.8	286.6	56.1
CAM-B3LYP	def2QZVPD	2197.4	2116.7	1463.1	491.4	2054.3	1783.9	949.7	497.3	453.8	151.5	487.5	292.3	67.3
M062X	def2QZVPD	2091.8	1930.7	1463.0	485.4	1994.3	1662.8	937.8	487.5	445.3	146.4	478.6	287.9	65.9
SVWN	def2QZVPD	2228.2	2092.7	1448.9	485.0	2064.6	1880.0	959.0	483.1	433.2	147.8	470.5	286.9	58.1
B3PW91	def2QZVPD	2217.8	2097.0	1457.4	490.6	2007.1	1869.7	953.1	481.4	442.0	144.4	477.1	291.6	66.6
B3LYP	def2QZVPD	2200.6	2084.2	1451.6	488.9	1992.9	1858.5	949.6	479.1	443.2	143.2	475.0	291.1	66.5

Table S108: Harmonic unscaled vibrational frequencies (in cm^{-1}) for S_2 of C_6N_2

method	basis	1	2	3	4	5	6	7	8	9	10	11	12	13
tHCTHhyb	aug-cc-pVDZ	2232.4	2085.5	1437.5	484.3	2025.6	1885.3	943.1	433.2	139.0	-1000.9	418.2	82.7	56.4
VSXC	aug-cc-pVDZ	2224.5	2073.4	1439.1	487.1	2069.6	1869.5	953.7	398.8	-107.5	-641.9	436.7	192.8	56.5
BMK	aug-cc-pVDZ	2256.0	2130.5	1443.6	482.6	2063.0	1869.3	941.2	439.7	143.8	-1273.7	421.6	63.5	-117.2
B1B95	aug-cc-pVDZ	2275.9	2139.1	1465.6	493.3	2061.6	1913.7	960.1	451.0	145.6	-920.0	441.5	158.6	58.3
B3P86	aug-cc-pVDZ	2265.6	2122.0	1460.3	492.0	2053.4	1911.9	957.7	446.6	143.0	-973.2	434.5	131.1	57.4
B98	aug-cc-pVDZ	2239.1	2099.7	1445.6	486.4	2026.7	1888.0	946.7	438.7	139.2	-1023.3	424.0	88.6	57.0
B971	aug-cc-pVDZ	2238.2	2096.6	1444.1	486.2	2025.2	1888.5	946.3	437.7	140.8	-986.1	424.0	110.2	57.7
B972	aug-cc-pVDZ	2262.9	2122.8	1460.1	492.1	2050.7	1908.6	957.1	449.7	143.1	-840.5	441.7	172.1	59.3
wB97XD	aug-cc-pVDZ	2261.5	2147.7	1457.9	489.8	2074.1	1856.3	949.5	450.9	-954.9	440.3	140.5	139.7	56.9
CAM-B3LYP	aug-cc-pVDZ	2260.6	2151.3	1463.0	491.6	2080.4	1859.6	953.1	456.8	142.2	-1048.2	445.6	127.0	57.8
B3PW91	aug-cc-pVDZ	2260.3	2117.5	1457.0	490.9	2048.5	1908.0	955.5	446.7	143.5	-946.1	435.5	140.9	57.4
B3LYP	aug-cc-pVDZ	2240.0	2101.6	1449.6	488.4	2028.1	1894.0	950.3	443.7	141.0	-1032.5	430.4	106.6	56.6
tHCTHhyb	aug-cc-pVTZ	2215.7	2087.0	1436.5	485.0	2021.9	1880.8	943.2	507.7	447.6	152.9	482.2	295.9	61.5
VSXC	aug-cc-pVTZ	2207.2	2068.9	1437.1	485.3	2018.3	1816.7	948.0	404.9	163.5	-379.4	455.1	303.6	90.6
BMK	aug-cc-pVTZ	2238.4	2136.0	1440.1	483.9	2071.4	1852.0	939.3	555.5	466.4	151.5	498.8	306.2	63.0
B1B95	aug-cc-pVTZ	2255.5	2135.5	1460.6	492.5	2057.3	1899.7	957.2	531.8	460.3	158.8	503.8	302.9	64.7
B3P86	aug-cc-pVTZ	2247.0	2121.0	1457.7	492.1	2049.7	1902.7	956.8	534.2	458.2	156.1	499.9	303.6	64.6
B98	aug-cc-pVTZ	2223.4	2101.1	1444.1	487.2	2025.8	1880.5	946.6	508.4	452.4	154.7	488.9	297.5	60.8
B971	aug-cc-pVTZ	2221.1	2097.0	1442.0	486.7	2022.5	1880.7	945.7	503.8	450.7	154.8	486.9	297.1	62.5
B972	aug-cc-pVTZ	2244.5	2120.4	1456.0	491.3	2046.7	1897.8	954.7	524.9	458.3	158.0	497.5	302.2	63.8
wB97X	aug-cc-pVTZ	2245.2	2146.3	1451.7	489.4	2102.9	1801.8	944.3	558.0	471.3	152.9	513.8	303.9	55.4
wB97XD	aug-cc-pVTZ	2246.7	2145.0	1455.3	490.3	2075.6	1843.7	948.8	551.8	472.6	155.8	504.6	302.3	60.6
CAM-B3LYP	aug-cc-pVTZ	2244.7	2148.0	1459.5	491.9	2083.7	1843.3	952.0	549.6	466.8	152.0	511.1	305.1	61.6
M062X	aug-cc-pVTZ	2264.4	2159.0	1453.8	490.9	2079.6	1863.3	950.7	554.3	466.4	151.6	508.6	302.6	60.6
SVWN	aug-cc-pVTZ	2235.8	2093.4	1441.3	486.7	2050.8	1874.7	947.0	606.8	418.8	158.5	507.1	280.3	84.7
B3PW91	aug-cc-pVTZ	2240.5	2115.6	1453.7	490.7	2044.0	1897.6	954.1	532.6	459.8	157.3	498.5	302.6	64.2
B3LYP	aug-cc-pVTZ	2222.1	2102.2	1448.0	489.1	2027.2	1884.7	950.3	525.2	455.7	155.6	495.7	301.3	63.0
tHCTHhyb	def2SVPD	2241.2	2114.2	1443.7	486.9	2060.7	1892.2	947.7	1004.8	483.9	184.9	599.9	348.3	67.4
VSXC	def2SVPD	2227.8	2097.2	1443.2	488.0	2065.6	1893.2	949.9	947.3	550.6	91.7	543.3	338.5	94.7
BMK	def2SVPD	2272.2	2159.5	1450.3	487.0	2107.2	1873.5	947.6	1079.3	506.6	199.0	651.3	370.5	70.8
B1B95	def2SVPD	2284.0	2166.2	1469.9	495.1	2099.9	1914.7	963.2	964.4	489.4	181.3	595.2	347.0	67.3
B3P86	def2SVPD	2272.7	2148.3	1465.0	494.0	2087.9	1915.2	961.1	1018.6	494.2	186.5	614.3	353.1	68.2
B98	def2SVPD	2248.5	2128.1	1451.1	489.0	2064.9	1891.6	950.9	986.4	488.5	186.9	601.0	351.3	66.5
B971	def2SVPD	2247.0	2124.7	1449.5	488.6	2062.3	1892.4	950.3	962.3	484.2	184.3	591.6	347.5	66.4
B972	def2SVPD	2268.4	2148.9	1464.2	493.6	2086.4	1909.4	959.6	950.0	481.5	177.0	577.5	340.8	66.6
wB97X	def2SVPD	2276.0	2169.5	1458.0	491.0	2140.6	1817.0	947.5	946.1	492.5	179.1	592.9	345.9	65.0
wB97XD	def2SVPD	2273.6	2169.9	1461.1	491.7	2113.2	1857.0	951.9	996.8	492.5	182.4	601.8	348.8	65.9
CAM-B3LYP	def2SVPD	2275.2	2174.3	1466.6	493.8	2120.8	1860.3	956.0	985.9	507.5	192.4	628.1	362.0	67.8
M062X	def2SVPD	2293.8	2190.2	1463.5	493.5	2119.2	1882.9	957.2	951.9	489.8	184.1	597.1	348.0	65.6
SVWN	def2SVPD	2270.9	2126.9	1453.4	491.6	2122.3	1915.1	960.2	1232.3	645.8	218.4	715.5	356.7	68.7
B3PW91	def2SVPD	2267.2	2143.9	1461.5	492.9	2083.2	1910.6	958.8	1015.2	491.5	184.2	607.6	350.7	67.6
B3LYP	def2SVPD	2249.5	2130.2	1455.2	490.8	2066.3	1897.7	954.4	984.6	499.2	191.6	621.1	358.0	67.9
tHCTHhyb	def2TZVPD	2219.2	2089.1	1437.5	485.0	2025.6	1881.9	943.5	491.6	446.2	153.5	479.4	294.1	63.2
VSXC	def2TZVPD	2209.4	2073.9	1437.1	486.6	2051.9	1881.8	949.2	643.1	152.5	-394.3	491.8	170.6	78.5
BMK	def2TZVPD	2245.0	2137.8	1442.8	484.2	2072.9	1857.6	940.9	523.6	460.5	152.4	488.6	296.0	64.9
B1B95	def2TZVPD	2258.3	2138.2	1462.1	492.8	2061.3	1901.1	957.7	513.7	458.0	155.5	497.9	302.1	63.3
B3P86	def2TZVPD	2250.3	2123.6	1458.8	492.0	2053.7	1904.4	956.9	513.2	457.9	155.6	494.2	300.9	60.2
B98	def2TZVPD	2226.4	2103.0	1445.1	487.1	2029.4	1881.5	946.6	498.2	452.5	154.9	484.5	296.3	58.6
B971	def2TZVPD	2224.2	2098.8	1442.8	486.5	2026.2	1881.6	945.7	495.7	449.6	155.5	483.4	295.4	60.6
B972	def2TZVPD	2245.9	2122.4	1457.1	491.6	2050.1	1898.2	955.1	498.5	453.0	152.9	494.0	300.3	59.8
wB97X	def2TZVPD	2247.3	2147.5	1452.3	489.4	2106.7	1802.8	944.4	534.0	467.7	151.6	506.5	301.8	61.4
wB97XD	def2TZVPD	2249.0	2147.3	1456.0	490.3	2080.3	1845.1	949.1	523.6	463.2	153.6	499.8	299.8	55.5
CAM-B3LYP	def2TZVPD	2248.9	2150.9	1460.7	492.1	2087.3	1846.3	952.1	540.3	470.1	158.6	508.5	304.4	67.0
M062X	def2TZVPD	2269.0	2161.9	1456.0	491.1	2081.0	1867.2	952.3	531.6	465.0	157.0	504.0	299.6	62.6
B3PW91	def2TZVPD	2244.1	2118.3	1454.8	490.7	2048.2	1899.3	954.3	510.2	457.1	154.0	493.2	300.5	61.0
B3LYP	def2TZVPD	2226.1	2104.9	1449.1	489.0	2031.5	1886.5	950.2	515.6	457.8	158.6	493.9	299.3	59.4
tHCTHhyb	def2QZVPD	2214.7	2086.5	1436.1	484.7	2021.4	1879.1	942.4	486.5	439.7	153.0	476.8	292.3	65.5
VSXC	def2QZVPD	2209.5	2028.1	1432.3	468.7	2352.0	1748.8	771.6	504.7	374.7	-368.9	782.3	323.2	63.5
BMK	def2QZVPD	2238.6	2133.9	1441.0	484.3	2068.8	1849.8	941.1	514.0	454.7	149.6	487.6	303.4	58.8
B1B95	def2QZVPD	2254.3	2134.9	1460.5	492.2	2057.0	1896.9	957.0	503.3	448.2	148.9	489.5	300.5	60.1
B3P86	def2QZVPD	2246.1	2120.4	1457.0	491.9	2049.2	1900.7	956.2	503.8	448.3	150.3	490.4	297.9	63.1
B98	def2QZVPD	2222.2	2101.0	1443.8	487.0	2026.0	1878.3	946.4	489.8	446.0	153.5	482.8	294.2	64.4
B971	def2QZVPD	2219.9	2096.7	1441.7	486.4	2022.5	1879.1	945.6	487.4	443.2	154.2	481.2	293.3	63.2
B972	def2QZVPD	2242.1	2119.4	1455.5	491.2	2046.3	1895.5	954.2	499.6	446.4	149.9	489.2	296.5	64.1
wB97X	def2QZVPD	2243.8	2143.7	1450.5	489.1	2102.6	1797.6	943.2	518.7	463.0	156.2	502.2	300.1	41.4
wB97XD	def2QZVPD	2245.2	2143.9	1454.2	490.1	2076.5	1840.2	948.4	509.2	457.2	161.3	494.5	297.3	54.5
CAM-B3LYP	def2QZVPD	2244.8	2146.8	1458.6	491.6	2083.6	1841.9	951.7	526.7	461.8	158.1	507.9	301.7	57.5
M062X	def2QZVPD	2263.0	2158.0	1454.9	491.3	2079.3	1861.0	951.0	512.7	455.5	146.4	500.5	301.0	65.3
SVWN	def2QZVPD	2314.7	1900.1	1426.6	507.2	2117.4	1990.9	960.1	481.0	-306.8	766.3	345.1	323.0	-39.1
B3PW91	def2QZVPD	2239.5	2115.0	1453.7	490.8	2044.0	1895.0	953.8	501.5	447.2	149.7	491.9	297.1	66.7
B3LYP	def2QZVPD	2222.0	2101.6	1447.6	488.8	2027.2	1882.5	949.7	501.3	450.2	153.0	490.2	298.6	59.2

Table S109: Harmonic unscaled vibrational frequencies (in cm^{-1}) for S_0 of C_6N_2

method	basis	1	2	3	4	5	6	7	8	9	10	11	12	13
tHCTHhyb	aug-cc-pVDZ	2294.0	2254.7	1338.6	473.6	2331.7	2147.6	923.0	468.4	159.5	-1026.3	450.7	103.6	-13.9
VSXC	aug-cc-pVDZ	2254.3	2232.2	1353.7	478.4	2304.6	2126.9	930.8	479.6	163.1	-755.5	470.4	196.0	59.5
BMK	aug-cc-pVDZ	2388.3	2316.4	1330.0	470.3	2410.2	2198.8	919.3	471.9	164.8	-1323.0	450.0	79.0	-153.2
B1B95	aug-cc-pVDZ	2373.6	2318.0	1349.7	479.6	2402.1	2211.5	935.1	484.9	164.1	-930.3	476.4	164.4	51.3
B3P86	aug-cc-pVDZ	2341.6	2295.2	1353.6	479.9	2375.9	2188.4	935.5	480.9	162.7	-989.6	468.3	140.4	44.2
B98	aug-cc-pVDZ	2324.1	2278.1	1336.8	474.3	2357.3	2173.6	924.4	471.9	161.6	-1048.4	455.0	106.9	-19.7
B971	aug-cc-pVDZ	2318.5	2272.7	1337.0	474.2	2351.7	2168.1	924.0	471.6	161.5	-1010.4	455.8	120.2	31.9
B972	aug-cc-pVDZ	2345.4	2299.0	1350.2	479.4	2379.6	2193.3	934.0	484.9	163.7	-851.6	476.9	176.0	55.2
wB97X	aug-cc-pVDZ	2471.6	2362.5	1312.4	470.9	2460.0	2262.0	918.1	488.1	165.6	-871.8	487.4	180.5	50.9
wB97XD	aug-cc-pVDZ	2432.7	2336.2	1321.6	472.7	2429.5	2236.0	921.7	482.4	164.0	-967.0	473.3	141.2	38.4
CAM-B3LYP	aug-cc-pVDZ	2432.4	2348.8	1328.4	474.7	2438.9	2247.2	926.1	486.2	165.0	-1053.7	477.8	140.1	29.4
M062X	aug-cc-pVDZ	2438.1	2357.9	1323.7	473.7	2450.5	2255.0	924.2	486.5	163.8	-961.7	479.5	146.7	40.1
SVWN	aug-cc-pVDZ	2276.5	2237.2	1375.5	482.3	2318.3	2118.1	942.2	470.7	157.8	-1131.9	452.9	104.6	-27.2
B3PW91	aug-cc-pVDZ	2336.6	2290.7	1349.7	478.7	2370.9	2184.5	933.0	481.2	162.9	-961.0	469.4	148.3	47.9
B3LYP	aug-cc-pVDZ	2322.8	2280.4	1340.5	475.9	2357.9	2178.2	927.3	477.5	162.4	-1048.8	463.2	125.4	30.5
tHCTHhyb	aug-cc-pVTZ	2288.9	2259.9	1329.8	473.1	2333.3	2158.7	920.4	568.2	479.9	166.6	531.1	299.7	64.1
VSXC	aug-cc-pVTZ	2242.5	2235.0	1344.6	476.7	2300.8	2131.3	926.7	545.4	485.2	169.5	529.9	304.8	65.4
BMK	aug-cc-pVTZ	2399.2	2332.2	1307.8	468.1	2424.0	2231.5	912.0	630.7	487.3	169.0	553.0	307.9	64.6
B1B95	aug-cc-pVTZ	2365.0	2317.7	1339.0	477.8	2399.0	2215.6	930.2	598.9	492.5	170.0	551.5	307.2	64.9
B3P86	aug-cc-pVTZ	2334.0	2297.1	1344.6	479.0	2374.3	2194.7	932.3	594.6	490.5	169.4	548.3	306.0	64.8
B98	aug-cc-pVTZ	2320.1	2283.0	1328.2	473.7	2358.9	2183.7	921.6	571.6	482.3	168.1	535.7	301.9	64.6
B971	aug-cc-pVTZ	2313.0	2277.0	1327.8	473.4	2352.3	2177.2	920.8	567.4	481.6	167.7	534.0	301.0	64.5
B972	aug-cc-pVTZ	2336.3	2298.1	1340.5	477.7	2375.8	2196.4	929.6	585.3	491.5	169.8	546.6	306.0	64.9
wB97X	aug-cc-pVTZ	2462.4	2365.6	1303.6	469.7	2458.2	2266.0	914.5	626.9	495.2	171.0	564.8	307.8	65.3
wB97XD	aug-cc-pVTZ	2424.6	2341.7	1314.6	472.2	2429.4	2242.4	919.2	617.7	492.1	169.9	556.2	304.7	65.1
CAM-B3LYP	aug-cc-pVTZ	2425.5	2352.6	1319.2	474.1	2438.9	2253.9	923.1	617.0	493.3	170.8	561.6	309.1	65.0
M062X	aug-cc-pVTZ	2432.3	2359.5	1316.5	472.9	2448.5	2259.0	920.3	624.3	494.9	169.2	560.7	305.7	63.8
SVWN	aug-cc-pVTZ	2258.4	2240.3	1365.0	481.4	2312.3	2125.0	938.5	582.7	477.3	164.2	536.3	300.1	62.6
B3PW91	aug-cc-pVTZ	2328.0	2291.6	1340.2	477.5	2368.4	2190.0	929.3	590.8	489.7	169.2	546.8	305.2	64.7
B3LYP	aug-cc-pVTZ	2317.0	2283.5	1332.4	475.5	2358.2	2185.9	925.1	586.1	487.4	169.3	544.5	305.9	64.8
tHCTHhyb	def2SVPD	2311.0	2270.0	1341.9	475.9	2353.6	2163.1	927.5	993.0	507.0	193.8	616.5	340.0	68.6
VSXC	def2SVPD	2266.4	2243.2	1354.8	479.1	2320.2	2138.5	932.8	920.6	500.3	186.1	579.7	327.6	67.5
BMK	def2SVPD	2414.8	2328.7	1333.1	474.1	2440.0	2216.1	926.0	1029.9	518.7	205.2	656.9	356.4	69.3
B1B95	def2SVPD	2388.9	2331.9	1351.6	480.9	2423.0	2224.5	937.9	953.6	512.7	191.7	615.3	337.1	68.5
B3P86	def2SVPD	2355.4	2309.3	1355.7	481.5	2395.3	2201.6	938.7	1006.2	516.9	195.2	631.1	344.4	69.4
B98	def2SVPD	2340.6	2292.8	1340.3	476.5	2378.9	2187.8	928.7	967.6	509.2	195.2	617.2	341.8	69.3
B971	def2SVPD	2333.9	2288.0	1340.2	476.3	2372.8	2182.2	928.1	946.9	506.4	193.0	609.0	338.2	68.7
B972	def2SVPD	2360.1	2311.7	1350.8	480.4	2398.9	2206.6	936.0	950.5	508.0	188.5	601.0	331.4	68.0
wB97X	def2SVPD	2477.2	2381.9	1312.7	471.6	2478.3	2273.7	919.7	928.3	511.3	189.9	616.5	333.6	69.1
wB97XD	def2SVPD	2438.5	2356.1	1323.5	474.0	2447.6	2248.4	924.1	976.0	512.0	192.1	620.3	335.7	70.4
CAM-B3LYP	def2SVPD	2443.3	2367.1	1330.8	476.5	2460.3	2260.1	929.6	941.9	521.7	199.5	641.0	350.3	70.4
M062X	def2SVPD	2452.4	2373.6	1325.8	474.8	2472.0	2267.6	925.8	925.6	510.9	192.7	614.3	335.2	69.0
SVWN	def2SVPD	2288.8	2262.3	1380.5	485.1	2340.9	2136.2	947.7	1249.5	542.5	208.5	717.1	367.2	71.7
B3PW91	def2SVPD	2350.7	2304.5	1351.5	480.2	2390.4	2197.7	935.9	1005.9	515.1	193.6	625.4	341.7	69.0
B3LYP	def2SVPD	2339.4	2295.4	1343.8	478.0	2379.6	2192.7	931.7	954.8	517.9	198.8	634.1	350.0	70.1
tHCTHhyb	def2TZVPD	2290.3	2260.4	1332.1	473.6	2334.3	2158.0	921.8	553.1	477.6	167.0	524.7	296.5	64.1
VSXC	def2TZVPD	2242.5	2234.9	1346.6	477.3	2301.0	2130.4	928.2	526.6	477.2	169.4	525.9	301.4	65.2
BMK	def2TZVPD	2399.0	2328.4	1315.4	469.8	2423.6	2223.2	915.8	603.0	482.9	168.1	536.6	300.9	64.4
B1B95	def2TZVPD	2366.1	2318.6	1341.1	478.4	2400.3	2216.2	931.8	578.6	488.7	170.3	544.5	303.5	64.7
B3P86	def2TZVPD	2335.3	2298.1	1346.5	479.4	2375.7	2195.1	933.6	576.9	488.0	169.6	540.0	302.5	64.7
B98	def2TZVPD	2320.9	2283.1	1330.3	474.2	2359.5	2182.7	922.9	561.0	480.5	168.5	530.1	299.1	64.5
B971	def2TZVPD	2313.6	2276.9	1329.9	473.8	2352.7	2176.1	922.2	557.2	479.4	168.1	528.3	297.9	64.4
B972	def2TZVPD	2337.3	2299.0	1342.0	478.2	2376.9	2197.3	930.8	558.2	485.9	169.8	539.7	301.9	64.7
wB97X	def2TZVPD	2461.7	2366.0	1304.6	470.0	2458.6	2266.3	915.5	603.2	490.7	171.3	555.4	303.9	65.1
wB97XD	def2TZVPD	2424.5	2342.1	1315.3	472.4	2429.8	2242.8	919.9	588.2	487.8	170.4	546.5	300.5	65.1
CAM-B3LYP	def2TZVPD	2426.3	2353.4	1321.1	474.5	2440.0	2254.0	924.5	607.2	491.9	171.6	554.8	306.7	65.1
M062X	def2TZVPD	2433.2	2360.1	1318.1	473.2	2449.3	2259.0	921.2	597.7	489.9	169.3	551.0	301.1	63.4
SVWN	def2TZVPD	2263.6	2243.6	1366.5	482.0	2316.5	2127.5	940.2	598.0	481.2	165.6	532.8	299.2	63.4
B3PW91	def2TZVPD	2329.6	2292.7	1342.0	478.0	2369.9	2190.5	930.7	573.8	487.2	169.6	539.6	302.2	64.6
B3LYP	def2TZVPD	2318.5	2284.4	1334.3	475.9	2359.6	2186.1	926.5	579.4	486.5	169.9	538.3	303.0	64.9
tHCTHhyb	def2QZVPD	2287.2	2257.7	1330.5	473.2	2331.8	2156.4	920.6	537.7	471.3	165.4	522.3	294.6	63.5
VSXC	def2QZVPD	2238.4	2231.5	1344.4	476.5	2297.1	2127.2	926.2	529.5	477.6	167.7	522.1	298.6	64.3
BMK	def2QZVPD	2397.1	2325.4	1311.9	469.1	2422.2	2223.6	914.1	575.5	478.7	168.8	539.5	303.8	64.1
B1B95	def2QZVPD	2362.5	2315.3	1339.4	477.9	2397.0	2213.5	930.4	565.6	483.3	168.7	541.4	300.8	64.1
B3P86	def2QZVPD	2331.7	2294.8	1344.9	479.0	2372.6	2192.6	932.3	559.3	481.3	168.1	537.4	300.3	64.1
B98	def2QZVPD	2318.4	2281.1	1328.8	473.8	2357.7	2181.7	921.8	547.5	475.1	167.1	527.9	297.3	64.0
B971	def2QZVPD	2311.0	2274.9	1328.4	473.5	2350.8	2175.0	921.1	544.6	474.1	166.7	526.0	296.1	63.8
B972	def2QZVPD	2334.2	2296.1	1340.6	477.8	2374.2	2194.8	929.6	557.4	483.2	168.5	537.8	300.1	64.2
wB97X	def2QZVPD	2457.9	2362.8	1303.1	469.5	2455.2	2263.0	914.1	586.5	484.3	169.4	551.7	300.6	64.3
wB97XD	def2QZVPD	2421.1	2339.8	1314.0	472.0	2427.2	2240.2	918.7	568.8	480.3	168.3	543.2	297.5	64.4
CAM-B3LYP	def2QZVPD	2422.2	2350.2	1319.6	474.0	2436.8	2251.0	923.1	588.2	485.8	169.9	551.9	304.4	64.3
M062X	def2QZVPD	2429.7	2357.4	1317.1	472.9	2446.7	2256.9	920.5	582.1	485.2	168.3	548.7	300.1	63.1
SVWN	def2QZVPD	2256.7	2239.2	1364.6	481.5	2311.4	2124.0	938.4	551.3	470.1	162.6	526.6	295.0	62.1
B3PW91	def2QZVPD	2326.1	2289.5	1340.6	477.5	2366.9	2187.9	929.3	557.7	481.0	167.9	537.0	299.9	64.0
B3LYP	def2QZVPD	2315.0	2281.0	1332.8	475.5	2356.5	2183.5	925.2	559.5	480.0	168.3	535.6	301.0	64.2
gas[20, 21]		n/a	2183	1287	n/a	2266	2097	n/a	501	455	170	490.5	276	61.5

Table S110: Harmonic unscaled vibrational frequencies (in cm^{-1}) for S_1 of C_8N_2

method	basis	1	2	3	4	5	6	7	8	9	10	11	12	13	14	15	16	17
tHCTHhyb	aug-cc-pVDZ	2163.9	2140.5	1909.3	1133.6	393.6	2106.5	1969.4	1533.1	769.3	427.8	96.1	-114.7	-2137.5	432.8	150.2	36.3	-738.2
VSXC	aug-cc-pVDZ	2169.8	2133.7	1908.7	1142.4	396.6	2088.0	2032.1	1536.9	774.4	433.5	162.9	95.3	-1627.8	441.4	167.3	37.5	-519.1
BMK	aug-cc-pVDZ	2187.5	2043.0	1892.4	1123.9	390.4	2165.2	1815.5	1537.8	764.7	435.4	98.1	-182.2	-2696.5	441.3	152.2	37.1	-915.3
B1B95	aug-cc-pVDZ	2196.7	2162.5	1943.0	1149.1	400.2	2166.7	1929.0	1560.8	781.2	448.2	101.8	93.3	-1983.9	451.7	159.9	37.4	-658.8
B3P86	aug-cc-pVDZ	2188.9	2172.8	1938.4	1149.2	399.6	2145.4	1971.6	1556.3	780.6	441.8	96.2	-53.7	-2090.3	446.3	155.5	36.9	-712.6
B98	aug-cc-pVDZ	2163.6	2147.7	1918.9	1136.0	395.2	2127.0	1936.7	1540.4	771.8	432.4	95.6	-120.0	-2190.7	437.5	150.5	35.9	-755.7
B971	aug-cc-pVDZ	2161.8	2147.2	1917.1	1135.4	395.0	2121.8	1941.8	1539.0	771.5	431.9	95.5	-93.4	-2117.7	436.7	152.4	36.4	-726.5
B972	aug-cc-pVDZ	2186.8	2171.8	1940.2	1148.5	399.9	2148.8	1960.9	1556.0	780.4	447.3	129.8	96.5	-1821.7	451.3	161.4	37.4	-595.2
wB97X	aug-cc-pVDZ	2249.0	2005.6	1909.1	1128.9	397.3	2231.3	1734.4	1551.1	772.2	452.8	115.0	95.1	-1920.4	456.2	156.0	36.1	-636.7
wB97XD	aug-cc-pVDZ	2221.1	2076.3	1929.9	1134.8	397.6	2199.1	1815.6	1552.7	774.3	445.0	98.2	-45.0	-2072.3	449.8	152.1	36.0	-703.3
CAM-B3LYP	aug-cc-pVDZ	2228.1	2089.4	1935.4	1139.1	398.8	2206.9	1822.7	1556.5	777.0	449.7	97.7	-86.2	-2233.8	454.7	153.1	34.5	-760.4
M062X	aug-cc-pVDZ	2173.4	1858.7	1445.8	1123.3	393.8	2169.9	1647.6	1558.2	771.0	445.4	99.3	60.6	-2039.6	449.0	146.7	35.1	-690.4
SVWN	aug-cc-pVDZ	2209.5	2156.9	1914.0	1149.7	396.7	2101.0	2078.1	1544.8	777.8	428.4	94.6	-110.7	-2323.2	436.1	156.2	35.9	-794.1
B3LYP	aug-cc-pVDZ	2164.8	2151.1	1924.4	1140.0	396.8	2127.9	1945.0	1544.6	774.6	438.0	96.9	-101.6	-2192.3	442.4	152.7	36.5	-754.7
tHCTHhyb	aug-cc-pVTZ	2148.3	2135.2	1909.0	1131.9	394.4	2111.6	1949.0	1530.1	769.1	600.9	481.1	310.6	104.4	509.2	443.0	187.4	37.7
VSXC	aug-cc-pVTZ	2148.2	2129.1	1907.2	1136.6	395.2	2088.6	2012.3	1528.2	771.0	496.4	472.8	306.9	104.8	483.3	428.4	187.6	41.7
BMK	aug-cc-pVTZ	2202.2	2026.3	1895.9	1119.5	392.1	2185.1	1782.2	1532.6	763.7	715.1	498.5	323.8	109.6	559.2	459.4	190.1	42.0
B1B95	aug-cc-pVTZ	2193.4	2139.6	1935.9	1144.4	399.6	2167.7	1904.7	1553.6	778.7	617.4	496.9	316.6	110.6	526.2	454.7	192.9	43.7
B3P86	aug-cc-pVTZ	2180.3	2157.3	1935.2	1146.7	399.7	2148.1	1949.7	1551.4	779.5	632.2	497.0	318.1	108.4	533.5	456.0	192.7	42.2
B98	aug-cc-pVTZ	2160.9	2132.2	1918.1	1134.7	395.9	2132.4	1917.7	1537.3	771.6	580.3	483.6	311.3	107.3	506.0	445.0	191.2	41.7
B971	aug-cc-pVTZ	2155.8	2132.0	1915.5	1133.6	395.5	2126.1	1921.3	1535.2	770.9	568.1	482.0	309.6	107.4	502.1	442.4	189.9	44.2
B972	aug-cc-pVTZ	2180.0	2153.8	1934.1	1144.4	399.2	2149.5	1939.5	1549.7	778.0	605.9	494.4	315.2	108.1	519.8	453.5	193.3	43.7
wB97X	aug-cc-pVTZ	2250.3	1995.9	1901.3	1125.2	396.8	2234.1	1713.5	1544.0	770.4	640.0	504.6	315.9	104.9	543.0	463.9	187.1	39.9
wB97XD	aug-cc-pVTZ	2222.5	2061.4	1925.3	1132.7	397.9	2203.0	1795.6	1548.2	773.8	672.8	503.3	316.2	103.9	543.4	459.6	189.0	38.3
CAM-B3LYP	aug-cc-pVTZ	2230.5	2071.7	1929.6	1135.9	399.1	2211.7	1800.1	1550.8	776.0	622.7	504.3	316.9	104.8	538.0	461.6	183.3	37.0
M062X	aug-cc-pVTZ	2184.3	1861.3	1600.1	1121.2	394.6	2179.2	1640.3	1550.5	769.1	672.0	498.5	318.8	102.6	545.4	456.5	181.3	38.3
SVWN	aug-cc-pVTZ	2175.9	2162.4	1928.4	1150.0	395.9	2101.9	2061.3	1533.3	772.1	630.9	486.4	313.8	103.3	530.8	442.0	188.5	39.3
B3PW91	aug-cc-pVTZ	2174.2	2150.6	1930.0	1143.4	398.7	2142.8	1942.2	1547.2	777.2	628.0	495.3	317.3	107.9	530.0	454.3	192.7	42.2
B3LYP	aug-cc-pVTZ	2160.8	2133.0	1921.7	1138.6	397.4	2132.2	1923.5	1540.7	774.6	601.6	491.2	314.5	107.3	520.4	451.7	191.2	41.6
tHCTHhyb	def2SVPD	2179.7	2154.1	1916.5	1137.1	395.9	2139.1	1972.4	1536.8	772.6	1341.6	561.6	363.4	121.8	773.8	479.6	226.1	42.9
VSXC	def2SVPD	2178.3	2156.1	1916.9	1141.4	397.0	2119.4	2045.6	1534.9	774.7	1243.3	516.3	340.7	123.6	743.5	411.5	145.4	-146.5
BMK	def2SVPD	2224.8	2042.3	1899.5	1128.9	394.3	2203.1	1817.1	1542.6	769.9	1401.5	607.2	387.9	128.8	856.6	503.3	241.6	45.0
B1B95	def2SVPD	2228.4	2160.3	1945.8	1150.9	401.7	2199.8	1929.4	1562.7	783.2	1281.2	558.2	358.7	119.3	743.7	482.1	221.6	43.3
B3P86	def2SVPD	2211.5	2176.7	1943.4	1151.7	401.4	2176.0	1973.0	1558.5	782.9	1368.7	576.6	369.2	122.2	792.8	489.9	228.0	44.1
B98	def2SVPD	2192.0	2149.5	1924.3	1139.4	397.4	2159.6	1939.8	1543.5	774.9	1302.8	563.0	365.5	121.6	766.3	483.0	227.1	44.1
B971	def2SVPD	2187.5	2150.4	1922.4	1138.8	397.1	2153.9	1944.4	1542.1	774.3	1267.2	555.1	361.1	120.4	748.6	478.7	223.7	43.9
B972	def2SVPD	2213.0	2170.9	1943.0	1149.9	401.1	2180.2	1959.8	1557.4	781.9	1272.0	541.0	350.0	117.4	716.1	474.5	215.5	43.2
wB97X	def2SVPD	2281.7	2006.1	1907.6	1129.3	398.4	2264.0	1732.4	1551.0	773.2	1247.2	556.2	359.0	117.7	740.2	483.5	218.1	42.6
wB97XD	def2SVPD	2252.3	2075.0	1930.5	1136.2	399.1	2230.9	1814.9	1554.0	776.0	1330.8	560.5	361.2	118.7	762.3	483.7	221.0	43.6
CAM-B3LYP	def2SVPD	2262.1	2088.5	1937.3	1141.1	400.8	2241.1	1822.9	1558.4	779.3	1276.8	590.4	378.7	124.3	799.4	500.9	233.8	42.8
M062X	def2SVPD	2210.7	1857.8	1447.7	1123.3	395.2	2205.5	1645.8	1557.1	772.3	1237.6	551.5	359.4	117.1	731.9	477.3	215.9	42.0
SVWN	def2SVPD	2210.5	2188.2	1921.4	1153.8	392.8	2158.3	2085.3	1550.1	778.9	1759.2	673.7	396.2	132.6	1003.3	528.2	252.8	45.7
B3PW91	def2SVPD	2206.5	2170.9	1938.9	1148.7	400.4	2171.6	1966.2	1554.8	781.0	1367.8	569.4	365.5	121.2	780.9	486.7	225.2	43.5
B3LYP	def2SVPD	2192.7	2153.8	1930.2	1143.6	398.8	2160.4	1948.5	1547.8	777.8	1281.8	586.3	376.4	125.0	799.0	495.2	233.4	44.8
tHCTHhyb	def2TZVPD	2151.8	2138.8	1909.9	1132.8	394.4	2113.6	1953.4	1531.6	769.4	509.6	472.2	303.3	105.6	483.4	425.8	186.2	38.6
VSXC	def2TZVPD	2150.9	2130.5	1905.1	1139.5	396.0	2088.1	2016.1	1532.5	773.0	500.7	467.5	307.4	103.4	475.7	401.2	180.6	39.5
BMK	def2TZVPD	2202.4	2032.6	1897.3	1122.2	392.4	2183.9	1792.1	1535.9	765.2	526.6	479.6	304.6	107.1	493.3	432.7	185.1	40.2
B1B95	def2TZVPD	2196.4	2143.0	1937.2	1145.6	400.0	2170.5	1907.9	1555.5	779.5	539.9	488.4	310.8	107.5	503.2	437.7	188.9	37.8
B3P86	def2TZVPD	2183.2	2161.3	1935.8	1147.5	400.1	2150.6	1953.5	1552.9	779.8	539.6	486.0	309.8	107.8	499.8	438.1	188.6	36.2
B98	def2TZVPD	2163.2	2135.7	1918.0	1135.3	396.2	2134.0	1921.2	1538.3	771.8	514.8	475.4	304.6	104.4	487.5	429.8	184.9	33.0
B971	def2TZVPD	2158.0	2135.5	1915.2	1134.3	395.7	2127.6	1924.9	1536.2	771.0	512.3	474.3	303.7	105.4	485.0	428.4	184.7	36.0
B972	def2TZVPD	2182.3	2155.2	1934.3	1145.2	399.6	2151.9	1941.1	1550.8	778.6	519.3	483.5	309.3	105.2	495.4	434.0	187.7	38.1
wB97X	def2TZVPD	2252.6	1995.2	1901.4	1125.9	397.1	2236.3	1715.3	1544.9	771.0	533.6	488.4	307.1	103.1	509.3	439.6	186.2	40.7
wB97XD	def2TZVPD	2224.9	2063.1	1925.8	1133.2	398.0	2205.4	1798.5	1549.1	774.0	528.3	484.1	305.9	105.6	501.8	437.6	186.3	44.6
CAM-B3LYP	def2TZVPD	2233.4	2076.0	1931.2	1137.1	399.5	2214.2	1805.0	1552.5	776.7	552.4	492.4	310.8	109.1	513.9	443.7	189.6	48.7
M062X	def2TZVPD	2187.8	1863.3	1606.6	1122.6	395.0	2182.6	1643.0	1552.8	769.7	525.4	485.2	308.3	106.4	505.2	438.0	183.7	41.8
SVWN	def2TZVPD	2186.5	2154.2	1913.4	1147.0	397.3	2105.0	2057.1	1541.7	777.1	605.4	479.8	307.1	104.7	503.0	428.1	186.5	39.9
B3PW91	def2TZVPD	2177.3	2154.9	1930.8	1144.3	399.0	2145.4	1946.3	1548.7	777.7	536.9	485.4	309.3	107.5	498.7	435.9	188.4	36.2
B3LYP	def2TZVPD	2163.8	2138.0	1922.6	1139.5	397.6	2134.8	1928.1	1542.3	774.8	542.0	484.5	308.2	109.3	497.7	436.6	187.6	35.7
B98	def2QZVPD	2161.3	2130.7	1915.8	1134.3	396.0	2132.2	1914.7	1536.6	771.8	466.4	304.1	101.8	485.2	484.6	423.0	183.3	46.3
B972	def2QZVPD	2179.6	2151.7	1932.4	1144.0	399.4	2148.9	1937.3	1549.0	778.0	494.9	472.8	309.0	104.3	500.8	434.7	191.8	39.1

Table S111: Harmonic unscaled vibrational frequencies (in cm^{-1}) for S_2 of C_8N_2

method	basis	1	2	3	4	5	6	7	8	9	10	11	12	13	14	15	16	17
tHCTHhyb	aug-cc-pVDZ	2181.9	2153.1	1918.5	1133.7	393.4	2114.8	2008.1	1529.1	768.9	434.4	97.2	-83.5	-2131.8	439.3	155.8	36.4	-729.7
VSXC	aug-cc-pVDZ	2177.3	2143.5	1912.8	1141.9	395.9	2155.0	2090.3	1500.2	766.6	355.4	180.6	-290.6	-1582.7	474.5	189.0	-63.4	-554.3
BMK	aug-cc-pVDZ	2232.1	2174.9	1939.2	1129.3	391.7	2191.0	1946.8	1530.2	767.0	442.7	99.3	-166.5	-2703.4	448.4	158.2	39.7	-908.8
B1B95	aug-cc-pVDZ	2226.8	2201.6	1962.6	1151.6	400.6	2179.2	2003.4	1556.3	782.4	455.8	119.6	100.0	-1989.5	459.0	165.4	38.6	-653.7
B3P86	aug-cc-pVDZ	2213.3	2189.5	1950.6	1150.1	399.6	2155.3	2020.6	1552.4	780.7	449.2	97.7	52.9	-2088.3	452.9	161.2	37.7	-705.1
B98	aug-cc-pVDZ	2187.7	2166.1	1931.4	1136.8	395.2	2136.6	1985.8	1536.4	771.9	439.6	98.7	-85.7	-2182.2	444.3	156.9	36.2	-745.0
B971	aug-cc-pVDZ	2186.6	2162.7	1929.1	1136.1	395.0	2131.5	1990.2	1535.1	771.4	438.9	96.9	-45.1	-2111.0	443.6	158.5	36.5	-716.8
B972	aug-cc-pVDZ	2210.5	2189.2	1951.5	1149.0	399.9	2158.7	2009.4	1552.1	780.6	455.5	147.2	99.1	-1823.5	458.2	166.7	38.0	-588.5
wB97X	aug-cc-pVDZ	2271.6	2137.3	1954.9	1132.5	397.6	2243.6	1836.9	1542.9	772.8	142.1	100.7	-1924.6	464.7	463.5	165.1	38.2	-624.7
wB97XD	aug-cc-pVDZ	2243.5	2165.5	1957.1	1136.6	397.7	2211.1	1898.4	1546.2	774.6	454.7	99.5	78.2	-2065.1	458.0	160.4	37.5	-689.4
M062X	aug-cc-pVDZ	2262.2	2206.6	1980.9	1143.3	399.1	2226.5	1936.2	1547.6	777.8	100.9	86.3	-2079.5	462.0	459.1	164.5	37.0	-687.1
SVWN	aug-cc-pVDZ	2216.8	2169.2	1922.3	1149.3	396.8	2190.8	2107.5	1508.3	771.3	434.8	109.1	-112.2	-2305.6	460.3	165.6	32.1	-803.9
B3PW91	aug-cc-pVDZ	2208.1	2184.4	1946.6	1147.3	398.8	2150.9	2014.9	1548.8	779.0	450.0	99.6	84.2	-2034.8	453.4	162.5	37.0	-681.0
B3LYP	aug-cc-pVDZ	2187.7	2167.6	1936.4	1141.0	396.7	2137.3	1991.4	1540.7	774.7	445.1	98.0	-63.0	-2189.2	449.5	159.7	36.2	-746.1
tHCTHhyb	aug-cc-pVTZ	2165.0	2147.7	1918.1	1131.6	394.1	2119.9	1986.1	1526.3	768.8	614.0	490.3	315.7	106.8	525.5	448.5	193.0	38.0
VSXC	aug-cc-pVTZ	2158.3	2143.6	1895.7	1138.9	395.0	2100.4	1900.8	1542.8	778.8	710.1	405.5	234.0	163.9	573.8	350.1	237.8	-79.1
BMK	aug-cc-pVTZ	2237.6	2150.3	1941.1	1124.7	393.1	2207.2	1909.0	1524.4	765.6	734.9	509.2	328.2	111.0	576.9	466.9	195.0	41.6
B1B95	aug-cc-pVTZ	2216.3	2182.5	1956.4	1146.7	399.9	2179.9	1975.6	1549.2	779.7	634.7	505.1	321.3	108.7	541.0	456.3	199.6	41.9
B3P86	aug-cc-pVTZ	2198.8	2177.3	1946.4	1147.2	399.7	2157.8	1996.2	1547.5	779.6	646.9	506.2	322.6	108.5	547.5	458.8	198.5	42.9
B98	aug-cc-pVTZ	2178.5	2154.5	1929.7	1134.8	395.9	2141.6	1964.3	1533.0	771.6	597.9	494.3	316.3	107.6	522.8	450.3	195.0	43.1
B971	aug-cc-pVTZ	2173.9	2153.1	1926.9	1133.9	395.5	2135.4	1968.1	1531.1	770.9	585.8	491.6	314.9	107.9	517.8	446.5	194.3	42.1
B972	aug-cc-pVTZ	2197.8	2174.5	1945.3	1144.7	399.1	2159.4	1985.5	1545.8	778.1	619.5	503.5	320.7	110.1	533.4	455.6	198.2	42.0
wB97X	aug-cc-pVTZ	2269.8	2114.3	1945.7	1128.2	397.2	2245.5	1810.9	1536.6	771.1	669.8	518.0	323.1	105.3	563.8	461.4	193.2	39.8
wB97XD	aug-cc-pVTZ	2242.3	2145.6	1952.7	1134.3	398.0	2214.3	1875.3	1541.9	774.0	699.2	512.1	324.3	106.7	565.0	465.9	188.8	40.1
CAM-B3LYP	aug-cc-pVTZ	2248.7	2143.0	1955.4	1138.4	399.5	2222.5	1875.5	1545.9	776.7	650.4	514.8	325.0	108.1	559.4	465.2	194.4	41.3
M062X	aug-cc-pVTZ	2257.5	2180.8	1969.8	1137.0	398.5	2227.8	1903.6	1539.3	774.6	691.9	515.9	324.2	112.0	565.8	467.2	196.2	40.7
SVWN	aug-cc-pVTZ	2185.2	2148.7	1922.7	1153.5	398.5	2103.4	2011.3	1555.3	783.6	603.6	401.3	323.6	-221.0	621.2	553.5	166.1	-60.2
B3PW91	aug-cc-pVTZ	2192.9	2171.1	1941.5	1143.8	398.7	2152.6	1989.5	1543.1	777.5	643.3	505.1	321.5	108.7	545.0	456.7	197.5	43.3
tHCTHhyb	def2SVPD	2196.0	2167.8	1926.2	1137.1	395.8	2148.6	2009.7	1533.0	772.4	1347.0	571.7	363.9	121.8	781.6	482.4	227.6	44.5
VSXC	def2SVPD	2178.3	2156.1	1917.0	1141.5	397.0	2119.8	2045.8	1534.9	774.6	1243.3	515.7	343.1	124.1	741.6	411.7	148.8	-142.2
BMK	def2SVPD	2266.2	2175.6	1945.6	1134.8	395.6	2230.1	1947.6	1535.3	772.4	1414.7	620.8	389.1	130.6	870.9	506.5	245.8	46.9
B1B95	def2SVPD	2251.9	2205.2	1967.8	1153.7	402.1	2212.7	2002.8	1558.4	784.4	1292.2	569.3	360.7	120.4	756.0	486.9	224.6	43.8
B3P86	def2SVPD	2230.4	2197.9	1956.1	1152.4	401.5	2186.9	2020.6	1554.7	783.2	1375.6	586.8	370.0	122.5	801.9	493.5	230.1	43.6
B98	def2SVPD	2210.3	2173.1	1937.4	1140.0	397.4	2170.4	1987.8	1539.6	775.0	1308.8	573.8	366.1	120.8	774.3	485.6	229.5	44.2
B971	def2SVPD	2206.2	2172.8	1935.4	1139.5	397.2	2164.7	1992.2	1538.2	774.5	1274.1	565.7	362.1	120.5	758.0	482.5	226.3	42.6
B972	def2SVPD	2230.8	2193.0	1955.6	1150.4	401.2	2191.1	2006.7	1553.5	782.1	1280.1	551.8	351.7	118.7	726.7	477.9	218.6	43.5
wB97X	def2SVPD	2302.2	2133.7	1954.6	1132.9	399.0	2276.3	1834.3	1543.7	773.9	1261.1	572.3	361.9	119.7	756.8	489.9	221.0	42.3
wB97XD	def2SVPD	2273.3	2163.8	1959.4	1138.5	399.5	2243.4	1898.3	1548.0	776.6	1341.9	574.8	363.5	120.9	776.1	489.4	223.7	42.0
CAM-B3LYP	def2SVPD	2281.1	2164.8	1964.8	1143.9	401.1	2252.7	1902.1	1553.6	780.1	1286.1	604.3	380.4	125.7	811.9	505.5	236.7	43.6
M062X	def2SVPD	2293.0	2202.9	1981.8	1144.1	400.5	2261.6	1931.2	1548.2	779.0	1253.2	570.3	360.5	119.9	749.2	484.9	224.0	41.5
SVWN	def2SVPD	2225.6	2188.3	1932.4	1154.3	399.5	2147.6	2107.5	1547.1	781.8	1745.1	1006.0	714.6	687.9	404.2	186.7	94.4	-137.1
B3PW91	def2SVPD	2225.6	2192.6	1952.0	1149.5	400.5	2182.6	2014.7	1551.0	781.2	1375.1	579.6	366.4	121.5	790.1	490.2	227.6	43.5
B3LYP	def2SVPD	2210.3	2174.7	1942.2	1144.1	398.9	2170.9	1993.6	1544.0	777.9	1287.3	596.7	376.8	124.9	807.2	498.5	235.4	42.6
tHCTHhyb	def2TZVPD	2168.7	2151.2	1918.9	1132.6	394.2	2121.9	1990.4	1527.7	769.1	524.8	480.7	307.8	108.4	495.2	432.3	189.4	37.1
BMK	def2TZVPD	2239.5	2158.8	1941.9	1127.8	393.5	2207.2	1919.1	1528.1	767.3	554.5	490.5	309.8	108.8	509.2	441.2	191.0	41.4
B1B95	def2TZVPD	2219.5	2186.0	1957.7	1148.0	400.4	2182.6	1979.1	1551.2	780.6	554.3	497.6	315.6	108.2	517.4	445.2	193.1	34.6
B3P86	def2TZVPD	2202.3	2181.4	1948.0	1148.0	400.1	2160.5	2000.4	1549.0	780.0	557.0	494.7	314.1	108.9	512.7	442.5	192.5	36.6
B98	def2TZVPD	2181.3	2158.1	1930.4	1135.6	396.1	2143.5	1968.3	1534.3	771.8	531.1	486.6	310.5	107.1	502.4	438.1	191.2	40.7
B971	def2TZVPD	2176.9	2156.4	1927.6	1134.7	395.8	2137.2	1971.8	1532.2	771.1	527.7	485.6	309.8	106.9	499.3	435.0	190.3	40.0
B972	def2TZVPD	2200.3	2176.4	1946.2	1145.6	399.6	2161.9	1987.3	1547.0	778.6	535.4	493.3	314.5	108.9	509.0	440.5	194.8	39.0
wB97X	def2TZVPD	2272.2	2115.0	1946.3	1129.1	397.5	2247.9	1813.5	1537.7	771.6	563.4	505.5	316.0	107.8	529.8	449.1	192.7	38.6
wB97XD	def2TZVPD	2245.0	2148.0	1953.1	1135.1	398.3	2217.0	1879.3	1542.9	774.4	556.5	498.9	313.4	108.7	521.5	446.3	192.6	39.0
CAM-B3LYP	def2TZVPD	2251.6	2148.0	1956.8	1139.5	399.8	2225.2	1881.1	1547.6	777.4	579.7	506.9	318.3	110.1	532.8	451.0	196.0	45.8
M062X	def2TZVPD	2260.3	2187.1	1972.4	1138.8	398.7	2229.9	1908.3	1541.7	775.5	550.1	500.3	314.5	107.0	527.6	447.5	194.5	42.5
SVWN	def2TZVPD	2193.7	2162.0	1919.8	1147.1	397.8	2116.7	2077.3	1541.7	776.8	639.9	421.1	312.7	112.0	487.2	333.1	226.8	-193.3
B3PW91	def2TZVPD	2196.5	2175.4	1943.2	1144.8	399.1	2155.4	1994.0	1544.8	777.9	554.0	494.3	314.0	108.9	512.1	441.4	193.1	38.8
B3LYP	def2TZVPD	2181.2	2158.1	1934.0	1139.8	397.6	2144.4	1972.6	1538.5	774.8	557.3	493.6	313.5	106.3	512.0	441.1	193.3	36.4

Table S112: Harmonic unscaled vibrational frequencies (in cm^{-1}) for S_0 of C_8N_2

method	basis	1	2	3	4	5	6	7	8	9	10	11	12	13	14	15	16	17
tHCTHhyb	aug-cc-pVDZ	2299.3	2229.5	2123.1	1106.8	385.0	2321.9	2217.5	1423.0	755.1	456.9	105.7	-143.0	-2192.1	463.6	172.9	36.3	-735.9
VSXC	aug-cc-pVDZ	2272.4	2187.9	2100.9	1117.7	389.4	2291.6	2196.1	1443.1	762.2	471.1	154.9	102.7	-1674.0	475.0	185.4	38.3	-511.8
BMK	aug-cc-pVDZ	2386.2	2304.8	2170.8	1099.8	381.5	2402.1	2274.9	1406.3	750.3	463.0	108.0	-239.1	-2789.7	470.2	171.8	36.5	-936.9
B1B95	aug-cc-pVDZ	2370.8	2306.8	2188.2	1119.0	389.5	2394.5	2281.0	1431.5	764.6	478.6	116.6	20.8	-2032.2	482.3	182.4	37.2	-650.7
B3P86	aug-cc-pVDZ	2343.1	2276.0	2164.5	1120.6	389.9	2366.9	2258.1	1437.4	765.1	471.7	110.3	-99.1	-2136.1	476.9	178.4	36.8	-704.6
B98	aug-cc-pVDZ	2325.5	2258.9	2149.8	1107.2	385.3	2348.4	2241.5	1419.3	756.1	461.0	107.1	-149.3	-2241.0	467.6	174.8	36.3	-752.0
B971	aug-cc-pVDZ	2319.6	2254.3	2144.6	1107.0	385.3	2343.1	2236.2	1419.8	755.9	460.9	107.7	-128.1	-2169.0	467.3	175.9	36.5	-722.6
B972	aug-cc-pVDZ	2347.4	2279.4	2169.9	1118.5	389.6	2370.4	2262.3	1433.5	764.2	479.1	125.5	83.3	-1870.6	482.4	183.8	37.7	-584.9
wB97X	aug-cc-pVDZ	2449.9	2404.0	2246.9	1093.4	381.5	2466.2	2327.1	1382.4	749.6	484.4	144.9	47.3	-1940.4	485.2	193.6	39.5	-575.9
wB97XD	aug-cc-pVDZ	2411.1	2372.7	2219.8	1099.6	383.3	2434.5	2301.7	1395.7	753.2	476.3	112.1	-113.5	-2102.5	480.4	179.2	36.2	-687.8
CAM-B3LYP	aug-cc-pVDZ	2415.1	2370.3	2228.4	1104.9	384.8	2439.2	2312.9	1402.5	756.5	480.5	113.0	-125.2	-2258.1	484.4	180.2	36.4	-743.4
SVWN	aug-cc-pVDZ	2282.6	2214.7	2090.8	1132.1	392.0	2309.6	2197.7	1466.4	769.8	459.1	105.3	-135.0	-2382.8	465.0	172.5	36.1	-795.1
B3PW91	aug-cc-pVDZ	2338.4	2271.1	2160.7	1117.6	388.9	2361.9	2253.8	1433.3	763.1	472.6	111.4	-75.7	-2082.5	477.5	179.5	37.0	-680.0
B3LYP	aug-cc-pVDZ	2326.0	2257.5	2154.6	1110.6	386.6	2348.3	2244.4	1422.9	758.5	467.5	109.0	-128.2	-2234.3	473.3	177.2	36.6	-745.6
tHCTHhyb	aug-cc-pVTZ	2303.5	2216.8	2132.7	1101.9	384.7	2317.6	2224.7	1412.3	753.2	654.4	519.0	311.5	110.1	561.3	466.9	202.9	41.0
VSXC	aug-cc-pVTZ	2270.6	2173.0	2103.8	1111.5	388.0	2282.4	2198.1	1432.4	758.9	558.0	514.6	312.8	112.0	534.0	465.5	205.3	41.8
BMK	aug-cc-pVTZ	2401.0	2310.1	2203.9	1087.9	379.8	2412.1	2296.7	1381.8	745.3	771.2	539.8	323.4	111.4	620.8	478.6	206.7	41.5
B1B95	aug-cc-pVTZ	2369.8	2292.1	2191.3	1111.6	388.1	2386.6	2282.2	1418.6	760.8	674.4	535.9	317.5	111.8	581.8	477.9	206.8	41.4
B3P86	aug-cc-pVTZ	2344.2	2261.7	2169.6	1115.1	389.2	2360.0	2261.5	1426.5	762.7	687.8	534.7	318.6	111.7	586.4	478.1	206.7	41.4
B98	aug-cc-pVTZ	2329.5	2248.3	2158.9	1102.1	385.0	2344.8	2248.3	1408.6	754.1	638.3	522.1	312.8	111.0	561.7	468.2	204.5	41.3
B971	aug-cc-pVTZ	2322.5	2242.7	2152.7	1101.5	384.8	2338.3	2242.0	1408.6	753.6	624.4	520.0	311.3	110.8	556.4	466.8	203.9	41.2
B972	aug-cc-pVTZ	2346.0	2263.7	2171.6	1111.9	388.2	2361.7	2262.8	1421.9	760.6	660.3	532.6	316.5	111.9	573.1	476.8	206.6	41.4
wB97X	aug-cc-pVTZ	2444.2	2398.6	2249.6	1087.8	380.6	2460.8	2330.0	1372.4	747.2	702.0	545.9	317.4	112.2	603.6	480.9	207.9	41.7
wB97XD	aug-cc-pVTZ	2408.0	2366.1	2224.8	1095.1	383.0	2428.8	2306.5	1386.6	751.3	734.0	542.0	316.4	111.7	603.5	480.3	206.7	41.6
CAM-B3LYP	aug-cc-pVTZ	2414.8	2361.4	2234.2	1099.4	384.4	2434.4	2317.1	1391.5	754.5	690.7	543.6	320.0	112.2	599.0	479.1	208.2	41.4
M062X	aug-cc-pVTZ	2424.8	2359.6	2237.1	1096.7	383.6	2442.3	2323.3	1389.5	752.5	733.4	545.5	317.9	110.8	607.5	482.2	206.5	40.0
SVWN	aug-cc-pVTZ	2280.1	2190.2	2096.1	1125.6	391.4	2295.6	2200.4	1453.6	767.1	676.3	521.9	313.5	108.0	576.1	465.0	200.7	40.0
B3PW91	aug-cc-pVTZ	2338.4	2255.7	2164.9	1111.5	388.1	2354.0	2256.2	1421.7	760.3	685.1	533.7	317.9	111.5	583.6	477.2	206.4	41.3
tHCTHhyb	def2SVPD	2324.6	2224.7	2134.1	1110.0	386.9	2336.2	2235.6	1424.3	758.3	1297.7	583.4	354.3	123.0	773.4	495.6	235.5	43.7
VSXC	def2SVPD	2291.0	2180.3	2107.6	1118.4	389.9	2298.4	2211.9	1441.9	763.2	1186.8	548.7	335.5	119.1	694.7	485.3	226.0	43.3
BMK	def2SVPD	2417.7	2303.5	2185.5	1105.5	384.9	2427.2	2291.2	1408.7	755.7	1332.5	627.4	375.7	128.8	846.0	511.5	249.8	42.3
B1B95	def2SVPD	2395.8	2300.1	2197.0	1120.6	390.6	2408.8	2296.1	1431.2	766.4	1238.8	583.7	349.9	121.9	750.7	499.5	233.2	43.8
B3P86	def2SVPD	2366.3	2269.7	2173.6	1122.6	391.4	2379.3	2273.8	1437.5	767.3	1323.7	599.1	360.8	124.2	792.5	506.0	237.9	44.4
B98	def2SVPD	2350.8	2253.4	2159.8	1110.4	387.3	2363.0	2258.3	1420.7	759.2	1254.1	585.7	355.9	123.9	766.6	497.9	237.1	44.3
B971	def2SVPD	2344.4	2248.7	2154.4	1109.9	387.1	2356.9	2253.1	1420.9	758.8	1221.3	578.5	351.7	122.7	750.9	494.7	234.4	43.9
B972	def2SVPD	2371.0	2271.6	2178.6	1119.2	390.4	2382.7	2277.5	1432.0	765.3	1236.7	567.9	340.6	120.1	726.2	493.1	229.2	43.5
wB97X	def2SVPD	2463.7	2405.4	2254.5	1094.0	382.3	2478.1	2342.3	1381.4	750.9	1198.3	589.7	349.5	122.0	751.9	500.6	231.6	44.8
wB97XD	def2SVPD	2427.3	2370.6	2228.3	1100.9	384.5	2444.6	2317.5	1395.3	754.8	1278.8	589.5	349.7	123.0	767.8	500.7	233.7	45.9
CAM-B3LYP	def2SVPD	2437.8	2366.5	2237.7	1107.3	386.5	2453.6	2328.6	1403.1	759.1	1201.7	616.3	369.2	127.1	795.0	513.2	243.5	45.1
M062X	def2SVPD	2450.6	2362.0	2242.1	1103.1	385.2	2463.3	2335.8	1398.5	756.3	1184.1	582.5	346.5	122.3	739.6	496.8	233.6	44.0
SVWN	def2SVPD	2307.1	2215.3	2104.7	1136.5	394.4	2323.8	2220.4	1469.7	773.8	1723.5	685.8	394.4	133.7	989.5	538.0	255.9	46.1
B3PW91	def2SVPD	2361.7	2264.5	2169.7	1119.3	390.3	2374.3	2269.4	1433.1	765.1	1325.5	592.7	356.7	123.3	782.5	503.5	235.9	44.2
B3LYP	def2SVPD	2351.3	2252.7	2164.9	1113.7	388.5	2363.0	2261.8	1424.4	761.6	1222.7	607.7	368.6	126.6	793.3	509.1	242.3	44.9
tHCTHhyb	def2TZVPD	2304.6	2218.0	2131.7	1103.6	385.1	2318.9	2225.0	1415.2	754.2	555.6	506.8	304.7	110.2	529.8	452.7	202.2	40.9
VSXC	def2TZVPD	2271.1	2172.8	2102.7	1113.2	388.5	2282.5	2198.0	1434.7	760.0	550.0	510.5	310.3	112.1	521.1	453.7	205.5	41.6
BMK	def2TZVPD	2400.8	2307.3	2194.7	1093.2	381.3	2412.0	2291.8	1390.9	748.1	590.1	516.3	308.6	110.8	549.6	457.3	203.9	41.0
B1B95	def2TZVPD	2371.5	2292.1	2191.4	1113.4	388.6	2387.9	2283.1	1421.1	761.9	588.5	524.5	311.9	111.8	553.7	464.8	206.6	41.3
B3P86	def2TZVPD	2345.7	2262.4	2169.6	1116.7	389.6	2361.5	2262.4	1428.8	763.6	583.5	520.8	310.9	111.6	547.4	462.7	205.8	41.3
B98	def2TZVPD	2330.3	2248.6	2157.5	1103.8	385.4	2345.6	2248.2	1411.2	755.0	564.0	511.6	307.3	111.1	537.0	456.4	204.0	41.2
B971	def2TZVPD	2323.1	2242.6	2151.1	1103.1	385.2	2338.9	2241.7	1411.2	754.5	560.9	510.2	306.2	110.8	534.3	455.2	203.5	41.1
B972	def2TZVPD	2347.5	2263.7	2172.1	1113.3	388.6	2362.8	2263.9	1423.7	761.5	570.5	520.4	310.5	111.7	544.6	462.5	206.1	41.3
wB97X	def2TZVPD	2444.3	2397.5	2249.6	1088.8	380.8	2460.8	2330.2	1373.6	747.8	609.2	532.1	311.2	112.0	570.2	467.1	207.5	41.5
wB97XD	def2TZVPD	2408.6	2365.4	2225.0	1095.9	383.1	2429.2	2307.0	1387.5	751.8	594.4	525.1	307.4	111.6	559.0	463.7	205.9	41.6
CAM-B3LYP	def2TZVPD	2416.2	2361.3	2233.8	1101.1	384.8	2435.5	2317.7	1393.8	755.3	615.3	532.4	314.6	112.4	570.9	468.3	208.1	41.3
M062X	def2TZVPD	2426.2	2359.7	2236.7	1098.0	383.9	2443.3	2323.8	1391.5	753.1	594.5	528.4	309.0	110.5	566.0	466.0	205.4	39.6
SVWN	def2TZVPD	2283.9	2195.9	2098.1	1127.3	391.9	2300.6	2203.7	1456.0	768.1	622.2	513.3	307.6	109.0	542.7	454.4	200.8	40.3
B3PW91	def2TZVPD	2340.2	2256.7	2165.0	1113.1	388.4	2355.6	2257.3	1424.0	761.2	581.9	520.4	310.5	111.6	547.2	462.4	205.6	41.2
B3LYP	def2TZVPD	2330.5	2245.2	2160.9	1107.6	386.7	2344.6	2250.3	1415.3	757.8	584.9	519.4	311.3	111.8	547.2	461.7	205.9	41.3
tHCTHhyb	def2QZVPD	2302.5	2213.5	2130.2	1102.2	384.7	2315.7	2223.0	1413.0	753.4	534.4	502.6	303.9	109.1	526.7	450.0	200.8	40.7
Kr[17]		n/a	2175	n/a	n/a	365	n/a	n/a	n/a	n/a	640	501	n/a	n/a	n/a	n/a	n/a	n/a

Table S113: Harmonic unscaled vibrational frequencies (in cm^{-1}) for S_1 of $C_{10}N_2$

method	basis	1	2	3	4	5	6	7	8	9	10	11	12	13	14	15	16	17	18	19	20	21
tHCTHhyb	aug-cc-pVDZ	2172.1	2104.0	2086.1	1589.5	958.5	331.3	2171.4	1944.4	1917.9	1258.3	649.8	437.2	157.3	69.7	-622.8	-3234.3	434.3	125.2	25.2	-137.2	-1473.8
VSXC	aug-cc-pVDZ	2160.5	2116.0	2083.1	1595.6	965.4	333.9	2151.8	2015.7	1917.6	1269.0	654.5	446.9	182.3	72.5	-406.2	-2431.5	443.2	165.4	126.3	26.5	-1114.0
BMK	aug-cc-pVDZ	2237.0	2062.0	1933.6	1592.5	953.2	328.7	2238.1	1920.6	1740.6	1246.3	645.8	445.9	158.3	71.2	-768.2	-4110.2	442.0	131.3	26.6	-200.6	-1812.7
B1B95	aug-cc-pVDZ	2230.8	2116.4	2095.1	1616.9	972.8	336.9	2233.3	1962.4	1877.5	1274.7	659.9	457.1	173.3	70.3	-544.4	-2994.7	455.1	129.0	79.8	24.7	-1352.8
B3P86	aug-cc-pVDZ	2209.8	2121.5	2115.7	1613.1	972.2	336.3	2210.5	1957.2	1930.3	1274.9	659.3	450.7	165.0	70.2	-600.4	-3159.0	447.9	128.7	25.3	-96.3	-1440.8
B98	aug-cc-pVDZ	2189.9	2093.3	2091.4	1596.4	961.3	332.6	2191.8	1936.7	1895.9	1260.5	651.9	441.7	158.8	69.3	-637.1	-3313.4	439.0	126.2	24.3	-143.0	-1506.9
B971	aug-cc-pVDZ	2184.9	2093.7	2092.0	1595.1	960.9	332.5	2186.4	1935.8	1901.2	1260.4	651.6	441.0	161.5	69.5	-611.3	-3205.2	438.3	126.8	25.1	-120.1	-1457.3
B972	aug-cc-pVDZ	2212.3	2115.9	2115.2	1612.7	971.9	336.6	2214.0	1957.5	1919.1	1274.3	659.3	456.9	176.8	71.2	-479.7	-2737.9	455.1	132.2	124.6	25.2	-1233.0
wB97X	aug-cc-pVDZ	2299.7	2094.0	1818.3	1601.4	960.5	334.1	2299.3	1972.5	1586.4	1249.8	651.2	462.3	169.1	70.9	-529.5	-2910.0	461.8	132.1	85.2	24.7	-1316.8
wB97XD	aug-cc-pVDZ	2265.0	2104.2	1946.9	1606.6	963.2	334.4	2266.1	1967.8	1705.5	1256.9	653.4	455.1	162.9	70.9	-589.2	-3130.6	453.0	130.9	28.2	-91.8	-1419.4
CAM-B3LYP	aug-cc-pVDZ	2273.2	2105.5	1979.0	1609.8	967.0	335.4	2273.9	1971.1	1721.9	1261.6	655.7	458.4	163.5	68.7	-636.2	-3375.8	456.6	129.8	29.5	-117.0	-1524.2
SVWN	aug-cc-pVDZ	2188.0	2160.6	2092.4	1603.7	970.7	333.8	2168.5	2067.2	1922.1	1277.0	656.7	436.3	161.2	67.6	-664.9	-3534.7	432.4	124.5	25.6	-137.7	-1599.2
B3PW91	aug-cc-pVDZ	2205.0	2116.1	2111.0	1609.4	970.0	335.6	2206.1	1953.1	1925.2	1272.2	657.8	451.3	167.8	70.2	-576.2	-3079.4	448.9	130.2	26.1	-62.7	-1398.9
B3LYP	aug-cc-pVDZ	2189.7	2098.3	2095.5	1600.7	964.8	333.9	2192.3	1942.6	1904.0	1265.0	654.4	446.1	161.2	69.9	-636.8	-3319.8	443.5	127.6	26.5	-130.9	-1511.2
tHCTHhyb	aug-cc-pVTZ	2172.6	2081.6	2080.0	1585.4	957.7	332.0	2174.0	1928.0	1909.6	1255.5	649.8	507.8	476.0	423.4	216.6	76.8	515.7	473.2	317.9	142.0	26.2
VSXC	aug-cc-pVTZ	2155.0	2097.1	2072.5	1586.9	963.7	335.6	2152.6	2071.4	1867.5	1236.9	653.6	558.6	485.1	378.5	94.7	-87.7	482.1	443.2	371.0	180.0	-102.1
B1B95	aug-cc-pVTZ	2230.2	2104.3	2070.1	1608.7	969.1	336.4	2233.2	1955.8	1850.2	1268.0	658.0	527.5	495.4	434.4	222.5	76.7	532.3	485.2	323.6	143.9	29.3
B3P86	aug-cc-pVTZ	2209.5	2106.4	2096.7	1606.9	970.4	336.4	2211.8	1951.3	1906.9	1270.9	658.5	535.9	496.0	437.0	221.2	79.1	547.8	488.9	326.4	144.3	26.2
B98	aug-cc-pVTZ	2192.7	2086.7	2069.7	1591.9	960.5	333.3	2195.7	1935.2	1872.9	1257.5	651.9	510.2	481.7	426.1	217.7	77.2	516.6	474.5	319.9	143.5	27.3
B971	aug-cc-pVTZ	2186.5	2084.6	2070.8	1590.0	959.6	332.8	2189.4	1932.5	1878.2	1256.6	651.3	506.3	479.4	423.3	215.5	76.9	510.6	470.7	318.7	141.7	28.6
B972	aug-cc-pVTZ	2210.7	2104.6	2091.6	1605.1	968.4	335.9	2213.4	1950.5	1895.1	1268.5	657.3	520.0	490.3	433.5	220.8	78.2	525.3	481.0	323.9	143.5	29.6
wB97X	aug-cc-pVTZ	2302.6	2083.7	1818.2	1592.2	957.5	333.6	2302.3	1969.2	1563.7	1243.4	649.5	549.8	501.0	446.7	217.4	73.2	554.7	489.7	323.7	141.9	24.3
wB97XD	aug-cc-pVTZ	2268.8	2095.5	1935.2	1599.8	961.9	334.7	2269.7	1965.1	1680.7	1252.7	652.8	543.0	494.1	440.8	214.4	77.1	550.4	488.6	323.1	142.6	28.1
CAM-B3LYP	aug-cc-pVTZ	2277.5	2094.3	1964.4	1602.1	965.1	335.9	2278.3	1968.0	1696.1	1256.7	655.0	548.1	507.1	439.6	214.0	75.9	553.3	492.5	325.9	143.9	25.0
SVWN	aug-cc-pVTZ	2175.5	2126.0	2086.7	1597.0	968.5	334.1	2163.7	2035.7	1917.6	1272.0	656.1	533.5	488.9	424.5	215.8	74.2	548.6	479.9	321.1	138.8	27.6
B3PW91	aug-cc-pVTZ	2203.8	2100.4	2089.9	1602.4	967.6	335.5	2206.2	1946.2	1899.4	1267.2	656.7	532.3	493.9	434.6	222.5	77.7	542.7	487.0	325.6	144.7	25.4
B3LYP	aug-cc-pVTZ	2191.6	2089.3	2070.7	1595.5	964.0	334.4	2195.0	1938.1	1879.5	1261.7	654.3	523.4	492.1	431.4	219.5	77.6	529.9	481.8	323.1	142.8	25.7
tHCTHhyb	def2SVPD	2201.9	2099.5	2088.4	1592.4	962.3	333.2	2200.0	1944.7	1922.0	1261.2	652.8	1624.4	692.5	477.4	255.3	86.1	963.4	548.3	374.0	164.5	32.1
VSXC	def2SVPD	2186.1	2114.8	2089.6	1592.6	965.4	334.4	2177.9	2027.6	1922.9	1267.2	654.6	1442.2	589.9	390.6	249.7	-276.8	965.6	530.1	372.5	220.2	-52.8
BMK	def2SVPD	2274.8	2061.9	1923.4	1596.9	958.7	332.1	2274.2	1925.3	1736.0	1250.6	650.4	1705.9	784.9	504.8	273.4	91.4	1074.9	595.5	403.8	174.3	31.1
B1B95	def2SVPD	2264.0	2115.1	2088.5	1618.1	974.9	338.1	2264.4	1964.1	1875.2	1275.7	661.7	1552.4	668.4	479.0	249.3	84.4	916.8	545.5	367.9	161.5	31.0
B3P86	def2SVPD	2239.0	2117.2	2113.9	1614.5	974.8	337.9	2238.5	1959.3	1929.5	1277.0	661.5	1662.3	714.1	489.2	257.9	86.0	990.8	563.8	381.4	165.2	31.3
B98	def2SVPD	2222.0	2094.2	2084.9	1598.8	964.8	334.6	2222.0	1940.1	1894.7	1263.3	654.8	1578.3	689.6	480.6	256.4	85.6	948.1	549.9	375.6	165.3	31.4
B971	def2SVPD	2216.3	2093.3	2087.3	1597.4	964.1	334.3	2216.3	1938.1	1900.8	1262.7	654.3	1528.7	674.1	476.2	253.2	85.1	922.7	542.7	370.9	163.2	31.8
B972	def2SVPD	2242.8	2113.6	2107.7	1613.1	973.4	337.6	2242.9	1958.3	1914.9	1274.9	660.7	1535.3	634.4	468.0	242.2	83.2	880.2	528.4	355.5	158.3	30.0
wB97X	def2SVPD	2332.2	2091.0	1814.7	1600.7	961.4	335.1	2330.8	1973.8	1581.6	1249.0	652.3	1500.9	678.4	481.4	245.1	83.6	912.7	548.9	370.5	160.8	27.6
wB97XD	def2SVPD	2296.8	2102.1	1940.4	1606.9	964.9	335.8	2296.3	1968.7	1700.3	1257.5	655.2	1613.1	684.3	479.5	248.2	85.0	946.0	549.6	371.1	162.0	28.2
CAM-B3LYP	def2SVPD	2307.5	2103.9	1973.2	1610.9	969.2	337.3	2306.9	1974.4	1717.8	1263.0	658.0	1532.8	738.9	500.7	264.4	88.3	983.4	580.5	393.0	168.3	29.4
SVWN	def2SVPD	2210.7	2160.2	2106.2	1608.9	975.7	336.1	2194.9	2074.6	1930.7	1281.9	660.7	2137.7	915.4	532.9	288.9	93.3	1301.4	660.4	417.1	179.8	32.3
B3PW91	def2SVPD	2234.4	2111.7	2108.4	1610.6	972.4	337.1	2234.1	1954.5	1922.7	1273.7	659.9	1659.0	699.0	484.7	254.7	85.7	974.5	556.1	376.4	163.7	31.3
B3LYP	def2SVPD	2221.9	2099.6	2089.6	1603.2	968.3	335.8	2222.4	1945.9	1903.7	1267.8	657.2	1535.8	734.6	496.0	265.0	87.9	987.2	575.4	390.3	168.6	31.9
tHCTHhyb	def2TZVPD	2175.0	2086.2	2082.0	1586.8	958.4	332.3	2175.9	1930.2	1912.9	1256.5	650.0	499.6	484.3	417.3	214.9	74.8	512.7	472.1	314.9	140.8	26.5
BMK	def2TZVPD	2255.0	2057.7	1927.5	1588.3	952.8	330.4	2256.0	1925.7	1709.6	1243.2	646.1	509.6	494.6	422.1	214.6	80.6	538.7	479.0	315.2	138.7	27.6
B1B95	def2TZVPD	2233.0	2105.5	2073.9	1610.2	970.2	336.7	2235.8	1956.8	1854.1	1269.6	658.5	526.7	503.9	429.3	216.7	71.2	531.1	482.7	321.4	140.1	28.9
B3P86	def2TZVPD	2212.1	2108.2	2101.4	1608.4	971.2	336.7	2214.1	1952.1	1911.6	1272.2	658.9	521.5	500.4	426.5	215.9	74.5	528.5	482.5	320.3	141.4	25.6
B98	def2TZVPD	2194.6	2087.7	2074.3	1593.1	961.0	333.7	2197.3	1935.2	1877.5	1258.6	652.0	505.9	488.9	422.7	212.6	72.4	518.5	474.9	316.7	140.4	22.9
B971	def2TZVPD	2188.2	2085.6	2075.3	1591.0	960.0	333.3	2190.9	1932.1	1882.5	1257.5	651.4	502.6	487.2	420.2	212.6	73.3	512.8	472.7	316.2	139.4	24.4
B972	def2TZVPD	2212.8	2105.1	2093.6	1606.2	969.3	336.3	2215.4	1950.8	1897.1	1269.6	657.8	513.6	497.2	425.1	216.7	74.4	512.9	474.8	320.5	142.0	27.2
wB97X	def2TZVPD	2304.4	2083.2	1816.2	1593.3	958.2	333.8	2304.0	1968.5	1566.2	1244.3	650.0	532.3	505.9	430.8	209.9	81.1	537.0	482.0	316.9	144.0	27.9
wB97XD	def2TZVPD	2270.6	2096.0	1936.8	1600.7	962.0	334.8	2271.5	1964.7	1684.7	1253.5	653.3	521.7	499.4	426.2	212.4	78.2	531.3	480.1	316.4	145.2	34.9
CAM-B3LYP	def2TZVPD	2279.8	2096.3	1969.5	1603.9	965.5	336.0	2280.6	1968.4	1702.2	1258.4	655.8	540.0	512.5	434.4	218.4	78.2	554.7	489.9	322.4	144.5	31.6
SVWN	def2TZVPD	2181.2	2135.3	2090.5	1598.7	969.1	334.3	2168.3	2044.1	1920.0	1273.7	656.3	545.5	499.0	416.1	214.4	74.8	560.9	478.8	316.4	139.7	28.0
B3PW91	def2TZVPD	2206.5	2102.5	2094.9	1604.0	968.4	335.8	2208.8	1947.1	1904.3	1268.4	657.1	520.1	499.7	425.3	215.9	75.4	528.0	482.0	320.5	141.9	24.1
B3LYP	def2TZVPD	2194.4	2091.3	2076.4	1597.0	964.5	334.5	2197.6	1939.1	1884.9	1263.1	654.6	520.9	499.1	426.1	215.9	74.3	534.9	482.8	319.6	141.6	24.9

Table S114: Harmonic unscaled vibrational frequencies (in cm^{-1}) for S_2 of $C_{10}N_2$

method	basis	1	2	3	4	5	6	7	8	9	10	11	12	13	14	15	16	17	18	19	20	21
tHCTHhyb	aug-cc-pVDZ	2183.7	2122.5	2092.3	1585.7	958.5	331.2	2177.0	1983.3	1927.3	1258.2	649.6	441.7	162.9	68.8	-616.6	-3230.9	439.2	126.9	27.8	-123.4	-1470.0
VSXC	aug-cc-pVDZ	2165.0	2125.1	2092.2	1592.7	963.6	333.9	2280.8	2102.9	1860.1	1188.7	646.2	432.7	145.3	82.4	-465.8	-2405.4	445.2	210.3	66.9	-20.6	-1096.3
BMK	aug-cc-pVDZ	2262.1	2124.0	2103.9	1583.0	955.5	329.6	2257.1	1961.3	1890.3	1251.4	646.9	450.6	165.0	69.9	-761.9	-4125.2	447.7	135.4	28.0	-196.0	-1819.2
B1B95	aug-cc-pVDZ	2246.2	2151.9	2136.6	1612.0	974.0	337.1	2242.9	1985.9	1953.0	1276.9	660.4	462.0	179.0	72.1	-539.6	-3004.3	460.7	132.0	91.7	26.9	-1354.7
B3P86	aug-cc-pVDZ	2223.1	2148.8	2125.9	1608.9	972.7	336.3	2218.0	1992.1	1957.0	1276.1	659.3	455.4	170.9	69.8	-594.4	-3159.3	453.9	130.2	27.4	-77.2	-1438.2
wB97XD	aug-cc-pVDZ	2277.2	2136.7	2076.1	1598.8	963.7	334.5	2274.2	1990.7	1804.0	1259.0	653.7	461.0	171.3	70.8	-577.7	-3126.0	459.4	133.1	30.8	-46.7	-1414.2
CAM-B3LYP	aug-cc-pVDZ	2283.9	2135.4	2082.3	1603.4	967.9	335.7	2282.1	1993.9	1814.2	1264.7	656.1	171.7	70.1	-626.9	-3379.1	464.6	462.7	134.0	28.2	-92.8	-1522.9
M062X	aug-cc-pVDZ	2296.9	2161.2	2133.6	1598.3	967.7	335.4	2293.4	2007.5	1863.7	1265.0	655.5	465.6	177.1	69.6	-569.4	-3149.2	464.5	134.1	34.9	19.2	-1419.3
SVWN	aug-cc-pVDZ	1599.8	971.6	336.0	2235.4	2195.7	2167.4	2095.5	2066.1	1977.7	1138.8	533.9	164.5	64.4	-674.0	-3528.2	445.4	443.4	121.7	36.5	-129.5	-1600.3
B3PW91	aug-cc-pVDZ	2218.2	2143.4	2121.5	1605.4	970.5	335.6	2213.6	1986.6	1953.2	1273.2	657.9	456.2	173.0	69.5	-570.4	-3081.0	454.6	130.7	30.9	-28.0	-1397.9
VSXC	aug-cc-pVTZ	2156.5	2096.7	2076.0	1585.8	964.4	334.3	2152.7	2070.1	1867.6	1238.2	654.0	557.7	485.2	378.8	96.5	-88.5	481.9	443.6	370.8	179.5	-101.8
BMK	aug-cc-pVTZ	2274.6	2106.6	2082.7	1574.6	952.8	330.7	2271.6	1964.8	1842.8	1244.9	645.9	569.7	507.6	445.3	225.2	78.4	593.8	500.5	333.2	148.0	31.9
B1B95	aug-cc-pVTZ	2243.1	2127.5	2121.7	1603.4	970.2	336.6	2242.1	1974.8	1925.9	1270.2	658.4	541.2	509.1	441.5	225.7	78.1	543.9	490.5	327.8	145.5	28.5
B3P86	aug-cc-pVTZ	2220.6	2122.9	2117.1	1602.6	970.5	336.4	2218.8	1970.1	1947.8	1271.5	658.5	549.4	506.4	441.5	224.7	75.4	560.9	494.8	330.2	145.0	32.1
B98	aug-cc-pVTZ	2203.1	2099.4	2096.7	1587.5	960.4	333.2	2202.6	1948.8	1919.3	1258.0	651.9	524.1	492.6	434.2	222.4	78.0	530.3	482.4	323.3	144.6	29.0
B971	aug-cc-pVTZ	2197.1	2098.4	2095.2	1585.5	959.5	333.0	2196.2	1947.6	1922.5	1257.0	651.3	519.2	491.7	430.0	220.8	78.3	524.3	479.4	322.0	144.1	27.0
B972	aug-cc-pVTZ	2221.4	2117.9	2115.3	1600.9	968.5	336.0	2220.5	1964.7	1939.4	1269.0	657.3	532.6	502.1	435.7	226.1	77.7	536.7	488.4	327.6	147.1	32.8
wB97X	aug-cc-pVTZ	2312.3	2124.6	2003.8	1584.4	958.4	333.9	2309.6	1996.8	1682.8	1246.5	649.6	564.8	522.3	448.3	222.1	76.4	572.0	500.2	329.8	144.8	25.5
wB97XD	aug-cc-pVTZ	2279.3	2126.0	2054.4	1592.7	962.2	334.8	2277.6	1987.3	1776.0	1254.7	653.0	554.9	511.5	444.5	221.6	76.2	564.1	497.6	328.2	142.2	26.7
CAM-B3LYP	aug-cc-pVTZ	2286.8	2123.1	2057.7	1596.6	965.8	336.0	2285.5	1988.2	1783.8	1259.2	655.4	565.7	524.2	444.5	223.5	72.9	571.4	501.7	331.5	145.4	31.1
M062X	aug-cc-pVTZ	2296.4	2140.8	2108.2	1588.9	963.5	335.2	2293.9	1998.5	1825.5	1257.2	653.3	563.5	517.5	446.5	222.3	77.2	572.8	503.9	330.4	144.6	21.4
B3PW91	aug-cc-pVTZ	2214.9	2116.4	2111.6	1598.1	967.7	335.6	2213.3	1964.3	1941.9	1267.9	656.7	546.6	505.2	439.0	224.4	78.9	556.1	493.5	329.3	144.9	30.9
B3LYP	aug-cc-pVTZ	2201.9	2100.1	2095.1	1591.3	963.9	334.5	2201.7	1951.2	1922.8	1262.1	654.3	504.0	436.3	224.1	75.9	543.9	537.3	488.9	327.2	145.0	29.5
tHCTHhyb	def2SVPD	2212.2	2118.0	2096.4	1588.5	962.1	333.3	2206.5	1983.0	1932.1	1261.3	652.7	1624.5	698.0	479.1	256.8	85.7	968.0	554.9	374.7	164.4	30.8
VSXC	def2SVPD	2186.2	2114.8	2089.5	1592.7	965.5	334.5	2177.8	2027.7	1922.9	1267.3	654.6	1442.2	589.6	391.3	255.4	-274.5	965.3	530.0	372.3	220.0	-54.3
BMK	def2SVPD	2298.7	2123.0	2095.6	1587.2	961.3	333.0	2292.8	1967.1	1884.5	1256.6	651.7	1708.3	792.1	505.1	277.7	93.3	1085.7	604.8	404.2	175.6	33.4
B1B95	def2SVPD	2277.2	2147.6	2135.7	1613.1	976.3	338.4	2273.4	1986.3	1952.2	1278.4	662.3	1556.7	676.3	481.6	252.7	85.7	925.5	552.7	369.4	162.4	31.0
B3P86	def2SVPD	2250.5	2143.3	2127.2	1610.3	975.1	338.0	2246.0	1990.5	1960.6	1277.8	661.6	1664.1	720.2	490.7	260.5	86.0	996.5	570.5	382.1	165.7	31.0
B98	def2SVPD	2233.0	2116.8	2104.6	1594.6	965.0	334.6	2229.4	1960.8	1937.0	1264.0	654.8	1578.0	696.1	482.3	257.2	86.1	952.9	556.7	376.4	165.2	30.1
B971	def2SVPD	2227.5	2117.8	2103.6	1593.2	964.4	334.4	2223.7	1963.5	1938.0	1263.4	654.4	1529.5	680.7	478.1	253.9	85.4	928.4	549.4	371.8	163.3	29.2
B972	def2SVPD	2253.7	2135.8	2125.2	1609.0	973.7	337.7	2250.3	1978.4	1956.3	1275.6	660.8	1537.9	642.0	470.1	246.4	83.3	887.2	535.2	357.2	159.3	30.0
wB97X	def2SVPD	2342.5	2135.4	2018.9	1592.4	962.4	335.3	2338.5	2003.9	1707.5	1252.4	652.6	1506.2	688.7	485.6	247.7	83.9	925.4	558.8	373.7	162.5	30.5
wB97XD	def2SVPD	2308.1	2135.9	2067.8	1599.6	965.6	336.0	2304.6	1992.6	1799.9	1259.9	655.4	1616.4	692.8	478.7	251.0	86.2	955.6	558.7	372.8	161.3	30.0
CAM-B3LYP	def2SVPD	2317.4	2135.6	2074.3	1605.0	970.4	337.6	2314.4	1996.1	1810.3	1265.9	658.4	1533.8	747.0	503.1	266.8	87.5	992.1	589.3	394.5	170.8	30.5
M062X	def2SVPD	2330.5	2157.1	2123.7	1598.0	969.0	336.7	2326.5	2008.7	1853.1	1264.9	656.7	1522.0	674.2	479.2	252.1	84.8	909.7	553.2	369.1	162.3	29.3
SVWN	def2SVPD	2221.1	2164.7	2109.1	1605.4	975.6	336.0	2199.3	2104.5	1935.7	1281.8	660.5	2117.4	1003.7	330.4	1311.2	657.0	655.6	456.1	93.2	54.9	-103.8
B3PW91	def2SVPD	2246.0	2137.7	2122.4	1606.5	972.8	337.2	2241.6	1984.6	1956.1	1274.5	660.0	1660.7	705.1	486.8	256.8	85.8	980.4	562.8	377.1	164.3	30.9
B3LYP	def2SVPD	2232.6	2118.2	2108.5	1599.2	968.5	335.8	2229.6	1966.1	1942.2	1268.4	657.3	1536.5	740.9	497.9	266.5	88.3	992.5	582.1	390.8	169.0	30.7
tHCTHhyb	def2TZVPD	2185.3	2103.6	2089.6	1582.9	958.2	332.2	2182.0	1963.4	1926.4	1256.4	649.9	511.2	492.5	420.4	217.0	76.9	524.6	477.2	317.1	142.6	26.2
BMK	def2TZVPD	2275.5	2109.3	2091.5	1578.6	955.4	331.0	2272.1	1964.0	1855.2	1248.5	647.1	530.7	506.0	429.0	216.7	79.8	552.9	487.8	318.9	142.2	26.3
B1B95	def2TZVPD	2246.1	2129.7	2125.2	1605.3	971.4	337.0	2244.7	1975.8	1930.0	1271.9	659.0	539.7	513.3	433.5	221.3	73.2	543.5	490.1	324.4	144.5	21.6
B3P86	def2TZVPD	2223.5	2127.9	2119.3	1604.3	971.4	336.7	2221.3	1973.0	1950.8	1272.7	658.9	535.7	509.4	429.5	221.0	75.7	540.9	489.4	322.7	144.3	23.6
B98	def2TZVPD	2205.4	2102.8	2099.2	1588.8	961.1	333.6	2204.4	1949.8	1923.5	1258.9	652.1	519.1	498.7	424.8	219.3	79.5	532.4	481.7	320.1	144.5	24.5
B971	def2TZVPD	2199.3	2102.7	2096.8	1586.8	960.2	333.3	2197.9	1949.0	1926.0	1258.0	651.4	517.0	497.1	423.6	219.0	78.6	525.7	479.7	318.6	144.6	25.7
B972	def2TZVPD	2223.9	2120.1	2116.3	1602.2	969.5	336.4	2222.6	1965.5	1941.6	1270.1	657.9	528.5	506.8	430.9	221.5	75.4	524.7	483.4	323.0	143.4	23.2
wB97X	def2TZVPD	2314.1	2124.3	2004.7	1585.5	959.1	334.0	2311.4	1996.5	1686.2	1247.5	650.2	553.9	524.3	437.7	218.7	77.0	553.8	495.8	323.9	144.6	31.3
wB97XD	def2TZVPD	2281.4	2126.9	2057.1	1593.5	962.6	334.9	2279.5	1987.4	1780.7	1255.5	653.4	543.0	516.5	433.8	218.6	78.0	548.9	492.3	321.8	145.5	31.2
CAM-B3LYP	def2TZVPD	2289.3	2125.2	2063.8	1598.3	966.4	336.2	2287.9	1988.8	1790.5	1260.8	656.1	561.8	527.0	441.6	221.4	81.0	572.6	501.3	328.0	149.3	32.8
M062X	def2TZVPD	2298.6	2144.9	2115.2	1591.2	964.7	335.3	2296.1	2000.7	1831.9	1259.2	654.0	547.8	519.2	439.1	221.7	75.6	568.9	498.3	324.5	146.9	22.0
B3PW91	def2TZVPD	2218.1	2121.7	2113.9	1599.9	968.6	335.8	2216.0	1967.2	1945.3	1269.1	657.1	535.1	509.0	429.9	221.2	74.4	540.6	488.8	322.6	143.8	25.1
B3LYP	def2TZVPD	2205.1	2102.5	2101.0	1593.0	964.7	334.6	2204.6	1953.0	1927.9	1263.5	654.5	534.1	508.3	428.9	221.4	78.7	547.2	489.4	322.5	144.6	26.4

Table S115: Harmonic unscaled vibrational frequencies (in cm^{-1}) for S_0 of $C_{10}N_2$

method	basis	1	2	3	4	5	6	7	8	9	10	11	12	13	14	15	16	17	18	19	20	21
tHCTHhyb	aug-cc-pVDZ	2311.7	2189.7	2173.3	1476.3	941.1	324.1	2298.8	2259.3	2104.4	1226.3	638.6	460.5	180.0	69.1	-609.1	-3288.6	456.6	135.6	25.9	-197.6	-1489.2
VSXC	aug-cc-pVDZ	2283.9	2168.6	2124.8	1500.4	950.5	328.1	2262.4	2239.8	2080.3	1240.2	645.2	473.9	201.9	73.8	-387.6	-2490.4	472.3	149.7	125.8	26.8	-1110.0
BMK	aug-cc-pVDZ	2392.9	2267.2	2242.4	1454.7	934.5	320.8	2389.2	2312.4	2152.4	1216.3	633.5	466.5	176.1	67.5	-769.0	-4192.7	461.3	138.8	26.9	-296.9	-1868.3
B1B95	aug-cc-pVDZ	2383.1	2258.8	2251.7	1482.0	951.9	327.7	2375.5	2321.8	2170.8	1237.4	646.3	480.1	194.6	71.7	-525.0	-3039.4	477.7	143.0	26.8	-116.7	-1369.1
B3P86	aug-cc-pVDZ	2356.0	2230.7	2221.1	1489.8	953.1	328.1	2345.2	2299.7	2146.4	1240.4	646.8	474.1	187.3	70.4	-581.3	-3199.8	470.8	139.7	26.3	-168.7	-1452.6
B98	aug-cc-pVDZ	2337.7	2214.6	2205.5	1470.7	941.8	324.3	2327.4	2281.7	2132.1	1225.4	639.3	464.7	182.1	69.4	-621.1	-3359.7	460.6	137.5	26.0	-205.5	-1522.0
B971	aug-cc-pVDZ	2332.3	2209.6	2200.4	1471.5	941.6	324.3	2321.8	2277.3	2127.0	1225.5	639.2	464.5	184.5	69.9	-594.6	-3253.2	460.7	137.9	26.1	-185.9	-1472.0
B972	aug-cc-pVDZ	2359.9	2235.0	2225.3	1485.5	951.7	327.9	2349.1	2303.1	2152.0	1237.9	646.2	480.7	197.9	72.6	-461.3	-2786.5	478.9	143.9	29.9	-26.2	-1248.0
wB97X	aug-cc-pVDZ	2453.0	2401.3	2302.8	1424.2	931.0	320.4	2461.4	2375.7	2236.0	1203.8	633.0	486.3	197.5	71.3	-491.0	-2916.5	484.6	146.0	27.0	-117.4	-1321.4
wB97XD	aug-cc-pVDZ	2418.9	2357.7	2277.0	1439.3	935.8	322.0	2425.4	2349.1	2208.3	1212.1	636.0	478.2	189.4	69.6	-562.5	-3149.1	475.3	141.3	26.1	-180.0	-1430.8
CAM-B3LYP	aug-cc-pVDZ	2425.3	2343.9	2286.9	1446.9	940.2	323.3	2427.3	2356.5	2215.1	1218.1	638.7	482.1	190.3	69.8	-603.6	-3378.5	479.0	142.9	26.2	-191.2	-1527.2
M062X	aug-cc-pVDZ	2435.8	2336.5	2294.3	1445.2	938.8	322.9	2435.7	2361.1	2218.4	1215.7	637.9	483.3	190.1	69.3	-556.0	-3160.7	480.8	141.5	25.3	-168.6	-1430.7
SVWN	aug-cc-pVDZ	2298.2	2169.1	2154.6	1524.7	960.6	330.0	2283.7	2247.9	2069.7	1256.2	650.5	461.7	179.6	68.1	-652.2	-3590.3	457.2	135.2	25.9	-192.3	-1601.3
B3PW91	aug-cc-pVDZ	2351.2	2226.5	2216.4	1485.5	950.6	327.3	2340.3	2295.1	2142.6	1237.0	645.2	474.8	189.5	70.8	-557.9	-3120.5	471.8	140.3	26.4	-149.8	-1410.9
B3LYP	aug-cc-pVDZ	2338.1	2217.7	2202.7	1474.4	944.7	325.3	2326.4	2283.9	2136.8	1229.0	641.3	470.3	185.4	69.9	-613.1	-3350.0	466.4	139.1	26.2	-190.4	-1516.5
tHCTHhyb	aug-cc-pVTZ	2311.9	2195.9	2160.1	1464.2	938.1	324.0	2296.4	2257.7	2112.8	1219.6	637.4	551.5	515.8	444.2	227.3	77.4	556.8	497.9	315.0	146.2	28.5
VSXC	aug-cc-pVTZ	2278.6	2169.6	2109.7	1488.7	946.0	327.1	2254.3	2235.4	2082.3	1232.8	642.6	528.3	509.7	445.1	230.2	79.0	527.8	490.9	317.1	148.6	29.2
BMK	aug-cc-pVTZ	2406.0	2270.5	2265.7	1426.7	926.5	319.4	2400.8	2323.4	2184.5	1199.9	629.8	610.2	539.8	456.1	232.2	78.1	624.9	516.0	326.5	148.3	29.0
B1B95	aug-cc-pVTZ	2379.1	2253.7	2242.8	1467.8	946.6	326.6	2369.2	2316.1	2173.1	1228.3	643.4	576.7	538.9	455.5	231.9	78.4	575.6	511.1	320.4	148.7	28.8
B3P86	aug-cc-pVTZ	2353.4	2232.8	2207.4	1477.6	949.4	327.7	2340.1	2295.6	2150.5	1233.3	645.2	581.0	534.6	455.9	232.0	78.4	588.5	513.6	322.7	148.6	28.8
B98	aug-cc-pVTZ	2338.3	2220.1	2195.4	1458.8	938.6	324.1	2325.7	2280.7	2140.1	1218.7	638.0	557.0	522.5	446.9	229.1	78.0	559.4	500.7	316.6	147.4	28.7
B971	aug-cc-pVTZ	2331.7	2214.1	2189.3	1459.1	938.0	324.0	2318.6	2275.5	2134.0	1218.3	637.6	551.9	520.4	445.5	228.4	77.9	552.9	498.2	315.0	147.1	28.7
B972	aug-cc-pVTZ	2355.2	2234.3	2210.1	1472.7	946.8	326.9	2342.4	2296.6	2152.8	1229.7	643.4	565.2	532.1	453.9	231.5	78.5	566.2	508.5	319.5	148.7	28.8
wB97X	aug-cc-pVTZ	2448.9	2390.0	2304.9	1412.8	927.0	319.8	2454.0	2374.3	2238.2	1196.5	631.0	609.2	556.1	460.3	233.0	78.6	604.9	520.5	320.3	149.2	29.1
wB97XD	aug-cc-pVTZ	2417.0	2346.2	2281.2	1429.3	932.9	321.9	2419.3	2349.2	2212.4	1206.2	634.8	597.5	545.2	457.6	231.3	78.2	601.0	517.0	318.4	148.3	29.0
CAM-B3LYP	aug-cc-pVTZ	2424.1	2332.7	2290.9	1434.6	936.8	323.2	2422.7	2355.3	2220.2	1210.9	637.4	606.3	554.0	459.3	233.7	78.6	603.6	519.8	324.1	149.5	28.8
M062X	aug-cc-pVTZ	2433.0	2327.5	2295.6	1433.2	934.5	322.6	2430.7	2358.4	2221.4	1208.5	635.9	601.9	550.6	459.8	231.7	77.2	603.3	519.6	320.6	147.8	27.2
SVWN	aug-cc-pVTZ	2289.7	2169.3	2129.8	1510.8	956.6	329.7	2269.0	2242.3	2073.3	1248.2	648.9	571.3	522.6	443.5	225.8	75.9	581.1	502.3	319.0	144.0	27.8
B3PW91	aug-cc-pVTZ	2347.5	2227.7	2201.6	1472.7	946.4	326.7	2334.2	2289.9	2145.9	1229.3	643.2	577.6	533.4	454.8	231.6	78.3	584.3	512.3	321.8	148.4	28.7
B3LYP	aug-cc-pVTZ	2337.2	2221.5	2190.1	1463.0	942.0	325.3	2323.4	2280.7	2142.3	1222.7	640.4	570.8	532.0	452.0	231.2	78.4	572.4	507.5	320.8	148.4	28.8
tHCTHhyb	def2SVPD	2329.7	2202.7	2167.1	1476.6	944.7	325.9	2318.7	2263.4	2111.9	1229.0	641.7	1594.7	695.5	489.8	264.6	86.6	958.5	564.4	365.6	166.6	30.7
VSXC	def2SVPD	2295.0	2178.7	2116.8	1498.2	951.6	328.7	2276.7	2240.3	2083.1	1240.4	646.2	1445.5	617.4	473.3	252.1	84.2	845.1	531.0	340.9	160.5	30.4
BMK	def2SVPD	2420.4	2262.6	2254.2	1455.3	940.5	323.8	2417.4	2316.9	2164.3	1220.6	638.6	1655.7	779.9	511.0	282.2	89.5	1062.6	610.8	392.3	176.1	28.4
B1B95	def2SVPD	2401.6	2263.9	2249.5	1480.7	953.9	328.8	2394.2	2324.9	2176.6	1238.4	648.1	1517.2	677.3	491.1	261.8	85.8	916.5	564.1	359.4	164.9	30.8
B3P86	def2SVPD	2372.3	2241.4	2214.2	1489.0	955.5	329.5	2362.4	2302.9	2152.3	1242.0	649.2	1630.1	717.5	501.0	268.0	87.4	985.8	580.6	373.7	168.4	31.3
B98	def2SVPD	2356.1	2226.1	2199.0	1471.1	945.2	326.0	2347.1	2285.3	2138.7	1228.1	642.3	1538.3	693.3	491.8	266.4	87.2	941.7	566.6	366.8	167.7	31.2
B971	def2SVPD	2350.0	2221.0	2194.0	1471.6	944.8	326.0	2340.5	2281.1	2133.4	1227.9	642.0	1492.6	679.5	488.1	263.3	86.4	918.4	559.9	362.1	166.0	30.9
B972	def2SVPD	2376.5	2245.3	2216.9	1483.0	952.9	328.8	2366.8	2304.9	2157.5	1238.0	647.5	1509.3	646.1	481.5	256.4	84.8	883.3	547.8	346.8	162.2	30.6
wB97X	def2SVPD	2467.8	2392.5	2313.7	1421.8	932.0	321.2	2471.1	2383.7	2241.4	1203.6	634.2	1438.3	686.9	494.8	260.7	86.1	911.2	572.2	362.0	164.5	31.8
wB97XD	def2SVPD	2433.9	2348.2	2288.7	1438.1	937.6	323.3	2435.3	2357.0	2214.1	1213.0	637.8	1555.1	691.6	494.1	262.6	87.1	942.4	570.4	360.8	165.7	32.9
CAM-B3LYP	def2SVPD	2444.1	2336.0	2298.5	1446.6	943.1	324.9	2443.0	2363.2	2221.9	1220.0	641.4	1451.9	737.9	510.3	275.1	89.2	967.9	599.4	384.4	172.2	31.8
M062X	def2SVPD	2455.5	2327.7	2304.0	1442.3	939.7	323.9	2453.5	2365.2	2224.2	1215.8	639.1	1463.7	670.1	488.0	262.3	86.1	891.7	562.6	356.2	165.3	30.8
SVWN	def2SVPD	2314.6	2185.2	2154.7	1527.6	965.3	331.9	2300.2	2260.0	2080.1	1260.8	654.5	2121.6	906.2	541.0	291.7	93.9	1292.6	669.8	415.8	181.6	32.6
B3PW91	def2SVPD	2367.5	2237.1	2209.2	1484.4	952.8	328.6	2357.6	2298.0	2148.5	1238.3	647.3	1628.7	703.8	497.3	265.4	86.8	971.4	573.5	368.3	167.0	31.1
B3LYP	def2SVPD	2356.4	2230.0	2196.9	1474.9	948.2	327.0	2346.6	2287.9	2143.9	1231.7	644.4	1484.2	733.8	506.2	273.6	88.9	973.8	591.2	383.3	171.4	31.6
tHCTHhyb	def2TZVPD	2313.0	2195.9	2161.5	1467.3	939.4	324.3	2297.9	2258.2	2111.4	1221.7	638.1	539.2	515.9	437.1	226.7	77.6	550.3	495.1	311.2	146.2	28.5
VSXC	def2TZVPD	2278.9	2169.0	2110.0	1491.1	947.4	327.4	2254.9	2234.9	2080.8	1234.5	643.5	527.5	512.7	439.3	230.8	79.0	520.3	482.5	317.1	148.6	29.0
BMK	def2TZVPD	2405.7	2267.7	2259.8	1436.5	930.5	320.7	2400.9	2319.9	2174.6	1206.3	632.1	564.8	530.6	439.3	228.2	77.8	585.7	504.0	313.1	146.9	28.7
B1B95	def2TZVPD	2380.5	2254.3	2242.6	1470.4	948.1	327.0	2370.7	2316.4	2172.7	1230.3	644.2	570.4	537.6	449.1	231.8	78.5	570.9	509.2	318.1	148.8	28.8
B3P86	def2TZVPD	2354.8	2233.4	2207.9	1480.0	950.7	328.0	2341.7	2296.2	2150.0	1235.0	645.8	562.1	532.3	446.2	230.7	78.4	567.3	507.1	316.9	148.3	28.8
B98	def2TZVPD	2339.0	2219.7	2195.7	1461.5	939.9	324.5	2326.6	2280.7	2138.3	1220.6	638.7	548.5	522.4	441.1	228.7	78.2	557.9	499.4	313.8	147.5	28.7
B971	def2TZVPD	2332.2	2213.4	2189.3	1461.8	939.3	324.3	2319.4	2275.1	2131.9	1220.1	638.3	544.9	520.1	439.9	228.1	78.0	552.3	497.4	312.6	147.1	28.6
B972	def2TZVPD	2356.3	2235.1	2209.9	1474.5	947.9	327.2	2343.6	2296.8	2152.8	1231.1	644.2	556.5	530.8	446.9	231.1	78.5	551.9	502.9	316.7	148.5	28.8
wB97X	def2TZVPD	2449.2	2387.9	2305.0	1413.9	927.8	319.9	2453.9	2373.8	2237.7	1197.5	631.4	595.0	551.0	451.5	232.4	78.5	588.0	514.8	316.3	148.9	29.0
wB97XD	def2TZVPD	2417.5	2345.0	2281.5	1430.2	933.6	322.0	2419.6	2349.1	2212.2	1207.0	635.1	578.0	540.8	447.3	230.1	78.2	578.8	509.7	312.2	148.0	29.0
CAM-B3LYP	def2TZVPD	2425.3	2332.1	2291.1	1437.0	938.0	323.4	2423.9	2355.5	2219.2	1212.6	638.0	595.9	551.1	452.4	233.3	78.8	600.4	517.8	320.3	149.6	28.8
M062X	def2TZVPD	2434.1	2327.6	2295.8	1435.3	935.5	322.8	2432.0	2358.5	2220.7	1210.0	636.4	585.8	547.0	450.9	230.5	77.1	596.9	515.8	315.4	147.6	26.9
SVWN	def2TZVPD	2294.1	2172.3	2135.8	1513.1	958.0	330.1	2274.2	2246.0	2074.9	1250.0	649.6	570.9	526.0	436.7	225.7	76.7	591.4	501.6	314.2	144.7	28.2
B3PW91	def2TZVPD	2349.1	2228.4	2202.4	1475.1	947.7	327.0	2336.0	2290.7	2145.5	1231.1	643.8	561.3	531.9	446.1	230.6	78.4	566.8	506.6	316.6	148.3	28.7
B3LYP	def2TZVPD	2338.7	2222.0	2190.6	1465.5	943.3	325.6	2325.2	2281.3	2141.5	1224.5	641.0	562.2	531.6	445.2	230.8	78.6	573.2	506.5	317.3	148.5	28.8
Kr[18]		2242	n/a	2137	n/a	n/a	322	n/a	n/a	n/a	n/a	n/a	n/a	n/a	n/a	n/a	n/a	n/a	n/a	n/a	n/a	n/a

Table S116: Scaling factors for fundamental vibrational frequencies in excited electronic states. Experimental values measured in gas phase

method	aug-cc-pVDZ						aug-cc-pVTZ						def2SVPD						def2TZVPD						def2QZVPD					
	f	s	RMS	f_b	s_b	RMS_b	f	s	RMS	f_b	s_b	RMS_b	f	s	RMS	f_b	s_b	RMS_b	f	s	RMS	f_b	s_b	RMS_b	f	s	RMS	f_b	s_b	RMS_b
tHCTHhyb	0.9765	0.0431	92	1.0327	0.2772	121	0.9703	0.0392	78	0.9100	0.3105	100	0.9366	0.0307	80	0.7526	0.0861	53	0.9756	0.0095	24	1.0066	0.0779	26	0.9745	0.0111	29	0.9690	0.0805	35
VSXC	0.9867	0.0363	42	n/a	n/a	n/a	0.9688	0.0515	108	0.9254	0.1133	39	0.9461	0.0238	61	0.7870	0.0509	28	n/a	n/a	n/a	n/a	n/a	n/a	0.9695	0.0199	39	0.9291	0.1157	43
BMK	0.9640	0.0645	144	0.8866	0.3386	187	0.9743	0.0150	26	n/a	n/a	n/a	0.9202	0.0376	98	0.7009	0.0824	55	0.9652	0.0111	29	0.9369	0.0722	32	0.9669	0.0106	28	0.9361	0.0695	30
B1B95	0.9611	0.0226	48	1.0200	0.1579	58	0.9554	0.0107	28	0.9137	0.0708	29	0.9289	0.0247	65	0.7697	0.0824	44	0.9545	0.0106	28	0.9208	0.0660	30	0.9574	0.0115	31	0.9376	0.0784	35
B3P86	0.9646	0.0256	55	1.0356	0.1791	67	0.9577	0.0105	28	0.9212	0.0667	29	0.9227	0.0311	82	0.7348	0.0786	49	0.9570	0.0106	28	0.9243	0.0674	30	0.9598	0.0106	28	0.9349	0.0694	31
B98	0.9719	0.0433	93	1.0136	0.2743	122	0.9711	0.0070	17	0.9788	0.0540	18	0.9325	0.0302	79	0.7492	0.0817	50	0.9678	0.0105	28	0.9498	0.0730	32	0.9681	0.0255	68	0.9383	0.1901	86
B971	0.9743	0.0389	83	1.0354	0.2561	108	0.9706	0.0153	30	0.9376	0.1130	36	0.9360	0.0284	74	0.7631	0.0817	48	0.9724	0.0085	21	0.9942	0.0695	23	0.9729	0.0219	44	0.9575	0.1608	56
B972	0.9633	0.0212	45	1.0237	0.1512	55	0.9583	0.0103	27	0.9251	0.0683	30	0.9292	0.0255	67	0.7719	0.0772	45	0.9584	0.0103	27	0.9321	0.0689	31	0.9602	0.0102	27	0.9380	0.0692	31
wB97X	0.9591	0.0180	49	1.0231	0.1329	58	0.9516	0.0109	29	0.8875	0.0483	22	0.9248	0.0250	66	0.7647	0.0613	35	0.9504	0.0114	30	0.8857	0.0520	25	0.9532	0.0114	30	0.9011	0.0609	29
wB97XD	0.9609	0.0235	65	1.0421	0.1710	80	0.9517	0.0246	66	0.8849	0.1748	79	0.9220	0.0282	75	0.7462	0.0708	43	0.9559	0.0071	18	0.9363	0.0463	16	0.9540	0.0104	28	0.9130	0.0615	29
CAM-B3LYP	0.9588	0.0234	65	1.0190	0.1705	81	0.9512	0.0103	27	0.8920	0.0486	23	0.9170	0.0309	81	0.7255	0.0658	40	0.9492	0.0110	29	0.8858	0.0518	24	0.9515	0.0104	28	0.8955	0.0521	25
M062X	0.9736	0.0224	61	1.0325	0.1347	57	0.9662	0.0192	50	0.8832	0.0548	26	0.9343	0.0344	90	0.7399	0.0638	38	0.9655	0.0194	51	0.8845	0.0559	26	0.9640	0.0305	80	0.8608	0.1768	80
SVWN	0.9752	0.0240	65	1.0460	0.1701	78	0.9337	0.0603	119	0.6798	0.1404	82	0.9133	0.0479	125	0.6582	0.0972	72	0.9761	0.0383	43	n/a	n/a	n/a	0.9798	0.0475	52	n/a	n/a	n/a
B3PW91	0.9664	0.0244	52	1.0343	0.1718	64	0.9604	0.0103	27	0.9255	0.0663	29	0.9255	0.0305	80	0.7409	0.0795	49	0.9595	0.0106	28	0.9268	0.0685	31	0.9623	0.0109	29	0.9380	0.0727	32
B3LYP	0.9727	0.0319	68	1.0390	0.2171	86	0.9659	0.0106	28	0.9224	0.0719	29	0.9297	0.0313	82	0.7388	0.0774	47	0.9644	0.0106	28	0.9261	0.0670	30	0.9677	0.0122	32	0.9430	0.0838	38

Table S117: Scaling factors for fundamental vibrational frequencies in the ground electronic state. Experimental values measured in gas phase

method	aug-cc-pVDZ						aug-cc-pVTZ						def2SVPD						def2TZVPD						def2QZVPD					
	f	s	RMS	f_b	s_b	RMS_b	f	s	RMS	f_b	s_b	RMS_b	f	s	RMS	f_b	s_b	RMS_b	f	s	RMS	f_b	s_b	RMS_b	f	s	RMS	f_b	s_b	RMS_b
tHCTHhyb	0.9674	0.0053	19	n/a	n/a	n/a	0.9660	0.0028	13	0.9394	0.0225	14	0.9544	0.0210	98	0.7810	0.1448	109	0.9657	0.0023	11	0.9450	0.0174	11	0.9668	0.0019	9	0.9512	0.0122	7
VSXC	0.9631	0.0390	200	0.7415	0.2523	283	0.9709	0.0044	21	0.9472	0.0226	13	0.9610	0.0180	84	0.8199	0.1394	99	0.9712	0.0041	19	0.9529	0.0191	11	0.9725	0.0042	20	0.9576	0.0189	11
BMK	0.9481	0.0071	26	n/a	n/a	n/a	0.9444	0.0076	37	0.9016	0.0387	25	0.9332	0.0224	106	0.7505	0.1397	109	0.9429	0.0060	29	0.9118	0.0282	18	0.9447	0.0060	29	0.9172	0.0187	12
B1B95	0.9359	0.0430	229	0.6378	0.2419	309	0.9469	0.0044	21	0.9055	0.0251	16	0.9360	0.0184	88	0.7843	0.1275	96	0.9465	0.0038	18	0.9122	0.0177	11	0.9478	0.0034	16	0.9186	0.0144	9
B3P86	0.9411	0.0455	242	0.6089	0.2432	321	0.9532	0.0039	19	0.9128	0.0262	17	0.9417	0.0210	99	0.7665	0.1389	107	0.9529	0.0033	16	0.9191	0.0196	12	0.9541	0.0027	13	0.9266	0.0142	9
B98	0.9606	0.0043	15	n/a	n/a	n/a	0.9590	0.0030	14	0.9301	0.0203	13	0.9479	0.0196	92	0.7855	0.1371	102	0.9585	0.0026	12	0.9343	0.0167	10	0.9594	0.0022	11	0.9397	0.0121	7
B971	0.9479	0.0468	248	0.6001	0.2472	327	0.9610	0.0028	13	0.9331	0.0193	12	0.9501	0.0187	88	0.7956	0.1346	99	0.9605	0.0024	11	0.9374	0.0159	10	0.9615	0.0020	10	0.9428	0.0117	7
B972	0.9423	0.0408	215	0.6788	0.2434	296	0.9517	0.0035	17	0.9121	0.0210	13	0.9410	0.0181	86	0.7910	0.1293	97	0.9516	0.0027	13	0.9212	0.0125	8	0.9526	0.0026	12	0.9234	0.0123	8
wB97X	0.9284	0.0407	218	0.6618	0.2365	296	0.9366	0.0077	37	0.8813	0.0306	20	0.9279	0.0180	87	0.7854	0.1154	87	0.9367	0.0073	35	0.8890	0.0228	15	0.9381	0.0069	34	0.8973	0.0205	13
wB97XD	0.9330	0.0442	237	0.6177	0.2399	315	0.9431	0.0062	30	0.8949	0.0300	20	0.9338	0.0196	94	0.7743	0.1289	99	0.9434	0.0056	27	0.9047	0.0195	13	0.9444	0.0053	25	0.9115	0.0162	10
CAM-B3LYP	0.9267	0.0466	252	0.5753	0.2360	326	0.9396	0.0068	33	0.8895	0.0287	19	0.9298	0.0187	90	0.7756	0.1197	90	0.9392	0.0065	32	0.8928	0.0247	16	0.9406	0.0061	30	0.9009	0.0204	13
M062X	0.9261	0.0436	235	0.6183	0.2372	312	0.9369	0.0073	36	0.8851	0.0299	20	0.9271	0.0178	86	0.7857	0.1148	86	0.9365	0.0067	33	0.8932	0.0215	14	0.9374	0.0064	31	0.8986	0.0188	12
SVWN	0.9783	0.0064	22	n/a	n/a	n/a	0.9774	0.0051	24	0.9470	0.0382	23	0.9548	0.0343	156	0.6796	0.1696	153	0.9761	0.0057	26	0.9407	0.0436	26	0.9781	0.0042	20	0.9608	0.0274	16
B3PW91	0.9434	0.0447	237	0.6235	0.2438	316	0.9553	0.0038	18	0.9146	0.0249	16	0.9434	0.0210	99	0.7683	0.1393	107	0.9549	0.0032	15	0.9207	0.0187	12	0.9561	0.0027	13	0.9272	0.0138	9
B3LYP	0.9442	0.0478	254	0.5800	0.2431	331	0.9576	0.0037	18	0.9180	0.0238	15	0.9469	0.0193	91	0.7823	0.1316	98	0.9571	0.0034	16	0.9206	0.0210	13	0.9585	0.0027	13	0.9291	0.0147	9

Table S118: Scaling factors for fundamental vibrational frequencies in excited electronic states. Experimental values measured in solid Kr

method	aug-cc-pVDZ						aug-cc-pVTZ						def2SVPD						def2TZVPD						def2QZVPD					
	f	s	RMS	f_b	s_b	RMS_b	f	s	RMS	f_b	s_b	RMS_b	f	s	RMS	f_b	s_b	RMS_b	f	s	RMS	f_b	s_b	RMS_b	f	s	RMS	f_b	s_b	RMS_b
tHCTHhyb	n/a	n/a	n/a	n/a	n/a	n/a	0.9704	0.0749	112	n/a	n/a	n/a	0.9215	0.0482	101	0.7272	0.0582	30	0.9743	0.0118	24	n/a	n/a	n/a	0.9776	0.0122	26	1.0315	0.0685	21
VSXC	n/a	n/a	n/a	n/a	n/a	n/a	n/a	n/a	n/a	n/a	n/a	n/a	0.9423	0.0340	72	0.8103	0.1055	48	n/a	n/a	n/a	n/a	n/a	n/a	n/a	n/a	n/a	n/a	n/a	n/a
BMK	n/a	n/a	n/a	n/a	n/a	n/a	n/a	n/a	n/a	n/a	n/a	n/a	0.9070	0.0646	133	0.6720	0.0578	33	0.9769	0.0116	25	1.0030	0.0676	22	0.9787	0.0114	24	0.9951	0.0687	22
B1B95	n/a	n/a	n/a	n/a	n/a	n/a	0.9595	0.0114	22	n/a	n/a	n/a	0.9211	0.0489	89	n/a	n/a	n/a	0.9588	0.0093	20	0.9833	0.0620	20	0.9617	0.0105	23	0.9969	0.0661	22
B3P86	n/a	n/a	n/a	n/a	n/a	n/a	0.9609	0.0094	20	0.9837	0.0647	21	0.9080	0.0487	103	0.7132	0.0584	31	0.9598	0.0093	20	0.9845	0.0632	20	0.9628	0.0105	23	0.9991	0.0660	21
B98	n/a	n/a	n/a	n/a	n/a	n/a	0.9718	0.0109	22	n/a	n/a	n/a	0.9199	0.0472	99	0.7285	0.0565	29	0.9721	0.0109	24	1.0130	0.0668	21	0.9707	0.0436	95	0.9864	0.3312	105
B971	n/a	n/a	n/a	n/a	n/a	n/a	0.9771	0.0193	29	n/a	n/a	n/a	0.9243	0.0434	91	0.7443	0.0561	27	0.9723	0.0112	23	n/a	n/a	n/a	0.9786	0.0207	31	n/a	n/a	n/a
B972	n/a	n/a	n/a	n/a	n/a	n/a	0.9623	0.0099	22	0.9907	0.0664	21	0.9197	0.0381	81	0.7573	0.0575	28	0.9624	0.0102	22	0.9959	0.0654	21	0.9640	0.0107	23	1.0020	0.0676	22
wB97X	n/a	n/a	n/a	n/a	n/a	n/a	0.9648	0.0126	27	0.9517	0.0798	28	0.9255	0.0390	83	0.7611	0.0591	28	0.9635	0.0129	28	0.9452	0.0793	28	0.9658	0.0133	29	0.9511	0.0828	30
wB97XD	n/a	n/a	n/a	n/a	n/a	n/a	0.9589	0.0439	95	0.9379	0.3171	106	0.9148	0.0449	95	0.7314	0.0593	30	0.9607	0.0117	24	n/a	n/a	n/a	0.9629	0.0126	27	0.9699	0.0873	30
CAM-B3LYP	n/a	n/a	n/a	n/a	n/a	n/a	0.9619	0.0103	22	0.9581	0.0703	24	0.9106	0.0492	104	0.7142	0.0559	29	0.9595	0.0112	24	0.9502	0.0751	26	0.9620	0.0098	21	0.9565	0.0656	23
M062X	n/a	n/a	n/a	n/a	n/a	n/a	0.9847	0.0239	50	0.9492	0.0873	31	0.9374	0.0564	115	0.7313	0.0577	29	0.9835	0.0239	50	0.9506	0.0904	32	0.9813	0.0515	108	0.9147	0.3100	109
SVWN	n/a	n/a	n/a	n/a	n/a	n/a	0.9246	0.0756	115	n/a	n/a	n/a	0.8802	0.0753	157	0.6245	0.0696	44	n/a	n/a	n/a	n/a	n/a	n/a	n/a	n/a	n/a	n/a	n/a	n/a
B3PW91	n/a	n/a	n/a	n/a	n/a	n/a	0.9637	0.0094	20	0.9874	0.0643	21	0.9114	0.0476	101	0.7194	0.0585	30	0.9625	0.0095	21	0.9880	0.0641	21	0.9651	0.0099	22	0.9996	0.0637	20
B3LYP	n/a	n/a	n/a	n/a	n/a	n/a	0.9709	0.0122	23	n/a	n/a	n/a	0.9173	0.0488	102	0.7214	0.0553	28	0.9690	0.0090	19	0.9876	0.0630	20	0.9720	0.0100	22	1.0019	0.0667	21

Table S119: Scaling factors for fundamental vibrational frequencies in the ground electronic state. Experimental values measured in solid Kr

method	aug-cc-pVDZ						aug-cc-pVTZ						def2SVPD						def2TZVPD						def2QZVPD					
	f	s	RMS	f_b	s_b	RMS_b	f	s	RMS	f_b	s_b	RMS_b	f	s	RMS	f_b	s_b	RMS_b	f	s	RMS	f_b	s_b	RMS_b	f	s	RMS	f_b	s_b	RMS_b
tHCTHhyb	n/a	n/a	n/a	n/a	n/a	n/a	0.9694	0.0052	20	n/a	n/a	n/a	0.9463	0.0422	155	n/a	n/a	n/a	0.9700	0.0083	32	n/a	n/a	n/a	0.9683	0.0115	39	n/a	n/a	n/a
VSXC	n/a	n/a	n/a	n/a	n/a	n/a	0.9793	0.0110	42	n/a	n/a	n/a	0.9602	0.0361	133	n/a	n/a	n/a	0.9795	0.0113	43	n/a	n/a	n/a	0.9674	0.0106	30	n/a	n/a	n/a
BMK	n/a	n/a	n/a	n/a	n/a	n/a	0.9415	0.0087	33	n/a	n/a	n/a	0.9225	0.0426	159	n/a	n/a	n/a	0.9424	0.0075	29	n/a	n/a	n/a	0.9442	0.0100	29	n/a	n/a	n/a
B1B95	n/a	n/a	n/a	n/a	n/a	n/a	0.9453	0.0042	16	n/a	n/a	n/a	0.9260	0.0358	135	n/a	n/a	n/a	0.9458	0.0067	26	n/a	n/a	n/a	0.9447	0.0068	19	n/a	n/a	n/a
B3P86	n/a	n/a	n/a	n/a	n/a	n/a	0.9537	0.0046	18	n/a	n/a	n/a	0.9310	0.0421	157	n/a	n/a	n/a	0.9545	0.0069	27	n/a	n/a	n/a	0.9509	0.0060	17	n/a	n/a	n/a
B98	n/a	n/a	n/a	n/a	n/a	n/a	0.9602	0.0044	17	n/a	n/a	n/a	0.9395	0.0386	143	n/a	n/a	n/a	0.9605	0.0075	29	n/a	n/a	n/a	0.9562	0.0053	15	n/a	n/a	n/a
B971	n/a	n/a	n/a	n/a	n/a	n/a	0.9624	0.0048	18	n/a	n/a	n/a	0.9425	0.0366	136	n/a	n/a	n/a	0.9626	0.0077	30	n/a	n/a	n/a	0.9580	0.0053	15	n/a	n/a	n/a
B972	n/a	n/a	n/a	n/a	n/a	n/a	0.9527	0.0045	17	n/a	n/a	n/a	0.9333	0.0362	136	n/a	n/a	n/a	0.9535	0.0075	29	n/a	n/a	n/a	0.9489	0.0060	17	n/a	n/a	n/a
wB97X	n/a	n/a	n/a	n/a	n/a	n/a	0.9233	0.0089	36	n/a	n/a	n/a	0.9080	0.0319	123	n/a	n/a	n/a	0.9243	0.0100	40	n/a	n/a	n/a	0.9321	0.0132	38	n/a	n/a	n/a
wB97XD	n/a	n/a	n/a	n/a	n/a	n/a	0.9315	0.0076	30	n/a	n/a	n/a	0.9137	0.0372	142	n/a	n/a	n/a	0.9331	0.0088	35	n/a	n/a	n/a	0.9389	0.0100	29	n/a	n/a	n/a
CAM-B3LYP	n/a	n/a	n/a	n/a	n/a	n/a	0.9303	0.0074	29	n/a	n/a	n/a	0.9136	0.0333	127	n/a	n/a	n/a	0.9308	0.0085	34	n/a	n/a	n/a	0.9363	0.0115	33	n/a	n/a	n/a
M062X	n/a	n/a	n/a	n/a	n/a	n/a	0.9287	0.0077	30	n/a	n/a	n/a	0.9134	0.0317	122	n/a	n/a	n/a	0.9298	0.0088	35	n/a	n/a	n/a	0.9342	0.0114	33	n/a	n/a	n/a
SVWN	n/a	n/a	n/a	n/a	n/a	n/a	0.9809	0.0061	23	n/a	n/a	n/a	0.9303	0.0718	256	n/a	n/a	n/a	0.9797	0.0067	25	n/a	n/a	n/a	0.9762	0.0069	19	n/a	n/a	n/a
B3PW91	n/a	n/a	n/a	n/a	n/a	n/a	0.9560	0.0047	18	n/a	n/a	n/a	0.9328	0.0424	157	n/a	n/a	n/a	0.9567	0.0070	27	n/a	n/a	n/a	0.9528	0.0061	17	n/a	n/a	n/a
B3LYP	n/a	n/a	n/a	n/a	n/a	n/a	0.9584	0.0062	21	n/a	n/a	n/a	0.9396	0.0372	138	n/a	n/a	n/a	0.9598	0.0072	28	n/a	n/a	n/a	0.9550	0.0065	18	n/a	n/a	n/a

Table S120: Harmonic unscaled vibrational frequencies (in cm^{-1}) for S_1 of $HC_{11}N$

method	basis	1	2	3	4	5	6	7	8	9	10	11	12	13	14	15	16	17	18	19	20	21	22	23
B972	aug-cc-pVTZ	3478.4	2218.9	2124.8	2089.7	2062.9	1937.7	1884.3	1603.2	1275.0	975.8	662.9	338.5	600.6	527.5	507.3	492.9	469.5	422.6	320.6	219.3	143.3	76.6	28.5
wB97X	aug-cc-pVTZ	3470.6	2311.6	2143.7	2085.5	1945.2	1765.1	1596.5	1527.1	1251.0	964.2	654.7	336.2	683.3	557.0	530.6	504.1	477.1	428.6	319.8	215.6	143.1	76.2	28.3

Table S121: Harmonic unscaled vibrational frequencies (in cm^{-1}) for S_2 of $HC_{11}N$

method	basis	1	2	3	4	5	6	7	8	9	10	11	12	13	14	15	16	17	18	19	20	21	22	23
B972	aug-cc-pVTZ	3478.6	2227.1	2138.4	2097.6	2086.0	1952.7	1924.7	1599.4	1275.5	976.0	662.9	338.5	610.8	538.5	520.2	502.6	477.2	426.1	323.2	222.4	144.5	77.0	28.5
wB97X	aug-cc-pVTZ	3470.9	2319.0	2157.3	2121.0	1984.8	1962.3	1654.2	1583.6	1254.0	965.1	654.8	336.3	691.4	575.0	553.8	522.9	491.0	435.5	324.8	220.9	144.7	76.6	28.0

Table S122: Harmonic unscaled vibrational frequencies (in cm^{-1}) for S_0 of $HC_{11}N$

method	basis	1	2	3	4	5	6	7	8	9	10	11	12	13	14	15	16	17	18	19	20	21	22	23
B972	aug-cc-pVTZ	3484.0	2346.1	2298.7	2270.8	2198.4	2184.1	2118.4	1476.7	1235.4	953.2	648.5	329.5	667.3	568.7	551.2	527.6	497.9	443.2	314.1	231.1	148.7	78.4	28.9
wB97X	aug-cc-pVTZ	3478.0	2448.7	2399.6	2378.9	2343.1	2264.3	2196.3	1414.7	1199.7	931.3	634.9	322.0	729.0	610.6	591.5	552.1	511.4	449.4	313.4	231.8	148.8	78.4	29.1

Table S123: Harmonic unscaled vibrational frequencies (in cm^{-1}) for S_1 of $HC_{13}N$

method	basis	1	2	3	4	5	6	7	8	9	10	11	12	13	14	15	16	17	18	19	20	21	22	23	24	25	26	27
B972	aug-cc-pVTZ	3479.6	2246.9	2149.2	2132.5	2087.9	2001.6	1939.6	1830.4	1639.0	1359.2	1109.3	846.3	573.0	291.8	618.4	568.5	541.4	514.4	495.0	467.9	420.5	329.6	240.5	172.5	109.3	57.3	21.0
wB97X	aug-cc-pVTZ	3473.1	2346.8	2191.2	2149.1	2077.2	1932.5	1633.7	1596.5	1370.7	1323.2	1093.7	835.7	563.0	289.2	700.8	599.3	562.1	527.8	508.7	471.6	424.9	327.9	236.3	172.6	109.1	56.9	20.8

Table S124: Harmonic unscaled vibrational frequencies (in cm^{-1}) for S_2 of $HC_{13}N$

method	basis	1	2	3	4	5	6	7	8	9	10	11	12	13	14	15	16	17	18	19	20	21	22	23	24	25	26	27
B972	aug-cc-pVTZ	3479.8	2252.3	2155.9	2149.1	2092.2	2029.0	1947.8	1880.0	1635.4	1359.7	1109.5	846.5	573.1	291.8	624.7	577.6	551.1	521.4	503.2	473.4	423.1	331.6	243.2	173.7	109.8	57.5	21.1
wB97X	aug-cc-pVTZ	3473.2	2351.4	2200.6	2169.0	2111.9	1972.6	1887.5	1620.5	1511.5	1330.0	1094.1	836.2	563.1	289.3	705.1	614.9	580.9	544.2	524.6	483.3	430.7	331.9	241.4	174.1	109.6	57.0	20.6

Table S125: Harmonic unscaled vibrational frequencies (in cm^{-1}) for S_0 of $HC_{13}N$

method	basis	1	2	3	4	5	6	7	8	9	10	11	12	13	14	15	16	17	18	19	20	21	22	23	24	25	26	27
B972	aug-cc-pVTZ	3484.3	2344.5	2295.5	2275.0	2245.0	2173.7	2145.4	2110.7	1509.9	1314.9	1082.6	828.8	561.1	284.0	665.6	600.7	575.2	542.2	523.0	488.4	436.4	321.1	249.8	176.8	111.2	58.0	21.2
wB97X	aug-cc-pVTZ	3478.5	2448.2	2402.5	2377.8	2364.4	2318.3	2250.0	2194.5	1439.4	1271.0	1053.8	809.5	548.6	277.2	727.8	638.8	611.2	572.2	543.3	498.7	441.4	319.2	250.1	176.6	110.8	57.8	21.3

Table S126: Harmonic unscaled vibrational frequencies (in cm^{-1}) for S_1 of $HC_{15}N$

method	basis	1	2	3	4	5	6	7	8	9	10	11	12	13	14	15	16	17	18	19	20	21	22	23	24	25	26	27	28	29	30	31
B972	aug-cc-pVTZ	3480.5	2266.5	2180.5	2147.8	2120.7	2079.0	1944.5	1940.8	1766.3	1665.1	1419.8	1206.9	982.1	747.1	504.4	256.4	629.5	549.6	524.2	508.1	503.2	483.8	453.0	410.7	332.1	255.4	195.4	136.9	85.4	44.3	16.2
wB97X	aug-cc-pVTZ	3474.7	2373.0	2231.3	2206.7	2139.8	2023.0	1944.6	1648.2	1441.5	1385.3	1203.1	1180.4	968.2	736.6	491.6	253.4	710.8	587.6	557.0	524.4	516.2	495.6	456.7	415.4	330.0	250.9	195.9	136.5	85.0	44.0	16.1

Table S127: Harmonic unscaled vibrational frequencies (in cm^{-1}) for S_2 of $HC_{15}N$

method	basis	1	2	3	4	5	6	7	8	9	10	11	12	13	14	15	16	17	18	19	20	21	22	23	24	25	26	27	28	29	30	31
B972	aug-cc-pVTZ	3480.6	2270.3	2188.2	2152.3	2138.0	2083.3	1971.9	1950.8	1819.2	1661.2	1420.4	1207.2	982.3	747.2	504.4	256.4	633.5	555.8	531.6	517.7	509.0	490.0	457.7	413.0	333.8	257.8	196.5	137.4	85.6	44.4	16.2
wB97X	aug-cc-pVTZ	3474.7	2375.6	2239.3	2217.9	2153.6	2076.6	1982.6	1819.6	1631.5	1402.7	1352.0	1185.6	968.1	736.8	491.9	253.4	712.9	597.3	569.5	549.5	529.4	508.3	467.7	420.9	333.6	255.9	197.3	137.4	85.2	44.0	15.9

Table S128: Harmonic unscaled vibrational frequencies (in cm^{-1}) for S_0 of $HC_{15}N$

method	basis	1	2	3	4	5	6	7	8	9	10	11	12	13	14	15	16	17	18	19	20	21	22	23	24	25	26	27	28	29	30	31
B972	aug-cc-pVTZ	3484.4	2343.3	2296.9	2277.8	2246.1	2222.3	2158.8	2109.1	2102.3	1533.4	1372.1	1176.8	961.3	732.5	494.3	249.6	664.0	575.2	551.6	541.0	524.9	505.4	470.5	424.9	322.5	263.2	198.9	138.7	86.1	44.6	16.3
wB97X	aug-cc-pVTZ	3478.5	2448.2	2398.8	2392.2	2356.1	2353.3	2298.8	2239.6	2193.4	1455.9	1320.7	1141.5	936.4	715.0	482.8	243.4	726.6	618.2	595.6	582.0	549.9	523.8	481.5	431.2	320.9	263.5	198.7	138.2	85.7	44.5	16.5

Table S129: Harmonic unscaled vibrational frequencies (in cm^{-1}) for S_1 of $HC_{17}N$

method	basis	1	2	3	4	5	6	7	8	9	10	11	12	13	14	15	16	17	18	19	20	21	22	23	24	25	26	27	28	29	30	31	32	33	34	35
B972	aug-cc-pVTZ	3481.2	2281.0	2193.5	2183.4	2140.6	2092.5	2073.9	1943.9	1886.4	1701.3	1682.9	1465.8	1280.6	1085.7	880.9	668.4	450.2	228.6	637.5	581.1	539.7	519.1	517.6	498.8	478.0	449.4	408.5	336.4	267.9	214.0	159.9	110.5	68.3	35.2	12.8
wB97X	aug-cc-pVTZ	3476.0	2393.2	2271.0	2246.4	2180.4	2106.0	2012.9	1938.9	1660.4	1418.9	1310.3	1257.2	1066.6	1039.3	866.3	657.9	433.5	225.3	717.4	619.6	574.0	537.2	530.3	515.3	485.9	452.3	412.7	333.2	262.8	214.8	159.4	109.9	67.9	35.0	12.7

Table S130: Harmonic unscaled vibrational frequencies (in cm^{-1}) for S_2 of $HC_{17}N$

method	basis	1	2	3	4	5	6	7	8	9	10	11	12	13	14	15	16	17	18	19	20	21	22	23	24	25	26	27	28	29	30	31	32	33	34	35
B972	aug-cc-pVTZ	3481.4	2283.6	2200.8	2189.5	2145.0	2110.8	2077.4	1950.2	1918.5	1756.8	1679.5	1466.1	1280.9	1085.6	881.0	668.5	450.3	228.5	640.4	586.7	545.9	526.4	523.1	504.8	483.2	453.2	410.5	337.8	270.1	215.1	160.6	110.9	68.5	35.5	13.1
wB97X	aug-cc-pVTZ	3475.9	2394.3	2275.2	2253.0	2188.4	2129.8	2065.6	1986.1	1766.3	1641.9	1420.4	1266.7	1212.4	1066.9	866.3	657.8	434.5	225.3	718.3	627.6	583.6	557.9	544.4	528.5	498.0	461.5	417.7	336.2	267.6	216.0	160.4	110.4	68.0	34.9	12.5

Table S131: Harmonic unscaled vibrational frequencies (in cm^{-1}) for S_0 of $HC_{17}N$

method	basis	1	2	3	4	5	6	7	8	9	10	11	12	13	14	15	16	17	18	19	20	21	22	23	24	25	26	27	28	29	30	31	32	33	34	35
B972	aug-cc-pVTZ	3484.5	2342.7	2293.3	2283.9	2259.1	2216.2	2204.0	2146.6	2100.4	2072.6	1550.7	1414.8	1248.0	1062.2	863.6	656.0	441.6	222.5	663.4	601.0	561.8	547.5	535.8	520.1	495.6	463.2	421.0	326.0	274.4	216.8	161.5	111.3	68.6	35.4	12.9
wB97X	aug-cc-pVTZ	3478.7	2448.2	2401.4	2386.4	2379.4	2344.9	2336.5	2283.4	2231.7	2192.6	1467.4	1356.7	1206.4	1031.8	841.3	639.9	430.9	216.8	726.4	641.6	602.9	590.8	562.7	544.5	511.5	472.5	426.9	323.5	274.3	216.4	160.9	110.7	68.3	35.3	13.1

Table S132: Harmonic unscaled vibrational frequencies (in cm^{-1}) for S_1 of $HC_{19}N$

method	basis	1	2	3	4	5	6	7	8	9	10	11	12	13	14	15	16	17	18	19	20	21	22	23	24	25	26	27	28	29	30	31	32	33	34	35	36	37	38	39
B972	aug-cc-pVTZ	3481.9	2292.1	2210.2	2191.9	2172.3	2128.8	2073.5	2058.4	1943.8	1835.0	1697.2	1634.7	1501.1	1338.5	1166.5	986.2	798.3	604.6	406.4	206.2	642.8	551.8	525.8	512.6	509.3	502.6	489.4	466.8	438.3	401.0	336.9	276.4	228.6	179.0	132.4	90.7	55.7	28.7	10.4
wB97X	aug-cc-pVTZ	3476.9	2408.8	2306.3	2275.6	2206.6	2158.5	2117.1	1972.5	1949.1	1666.4	1444.3	1306.8	1221.6	1145.9	967.4	899.6	778.8	593.9	384.4	202.6	720.8	596.2	567.8	535.9	529.1	518.3	502.4	475.2	441.7	405.4	333.2	271.1	229.6	178.2	131.7	90.1	55.3	28.5	10.4

Table S133: Harmonic unscaled vibrational frequencies (in cm^{-1}) for S_2 of $HC_{19}N$

method	basis	1	2	3	4	5	6	7	8	9	10	11	12	13	14	15	16	17	18	19	20	21	22	23	24	25	26	27	28	29	30	31	32	33	34	35	36	37	38	39
B972	aug-cc-pVTZ	3481.8	2293.8	2215.0	2198.4	2179.1	2132.0	2086.7	2068.3	1950.5	1869.4	1694.0	1692.5	1501.5	1338.7	1166.7	986.2	798.4	604.6	406.5	206.1	644.6	555.1	530.2	519.8	515.9	507.6	494.1	470.9	441.5	402.8	338.0	278.3	229.4	179.5	132.7	90.9	55.8	28.7	10.4
wB97X	aug-cc-pVTZ	3476.7	2408.7	2306.7	2278.9	2212.1	2176.7	2133.1	2038.7	1993.4	1726.7	1646.6	1447.1	1303.8	1149.9	1079.9	968.5	781.4	593.7	386.7	202.7	721.0	599.9	573.2	554.7	546.7	530.9	513.5	485.0	449.9	410.1	335.9	275.7	230.6	179.4	132.2	90.3	55.3	28.4	10.2

Table S134: Harmonic unscaled vibrational frequencies (in cm^{-1}) for S_0 of $HC_{19}N$

method	basis	1	2	3	4	5	6	7	8	9	10	11	12	13	14	15	16	17	18	19	20	21	22	23	24	25	26	27	28	29	30	31	32	33	34	35	36	37	38	39
B972	aug-cc-pVTZ	3484.6	2342.3	2294.9	2280.4	2266.3	2241.0	2193.1	2183.4	2137.2	2095.6	2044.1	1563.7	1447.6	1303.2	1141.0	966.6	783.4	593.7	399.0	200.8	662.5	568.4	544.9	539.9	532.8	520.6	506.0	481.3	450.4	412.7	326.2	281.9	230.7	180.2	133.1	91.0	55.9	28.8	10.5
wB97X	aug-cc-pVTZ	3478.7	2448.3	2400.7	2391.8	2378.0	2363.1	2338.6	2319.8	2271.0	2225.7	2192.1	1475.6	1383.6	1255.9	1105.3	939.5	763.0	578.8	389.0	195.5	725.6	610.6	591.6	585.4	570.0	547.4	526.2	496.2	459.8	419.2	323.8	281.6	230.2	179.5	132.4	90.5	55.6	28.7	10.7

Table S135: Harmonic unscaled vibrational frequencies (in cm^{-1}) for S_1 of $HC_{21}N$

method	basis	1	2	3	4	5	6	7	8	9	10	11	12	13	14	15	16	17	18	19	20	21	22	23	24	25	26	27	28	29	30	31	32	33	34	35	36	37	38	39	40	41	42	43
B972	aug-cc-pVTZ	3482.3	2300.7	2221.6	2208.4	2176.3	2159.4	2120.8	2063.5	2030.9	1943.2	1787.6	1707.5	1569.1	1528.9	1384.3	1231.7	1070.5	902.9	729.6	551.7	370.2	187.7	647.0	570.8	536.5	519.9	515.4	512.2	500.3	484.1	463.9	435.6	399.7	339.8	284.7	241.2	195.3	151.4	111.0	75.6	46.3	23.8	8.7
wB97X	aug-cc-pVTZ	3477.5	2420.4	2333.7	2298.6	2233.9	2202.7	2162.5	2092.9	1980.2	1930.4	1670.0	1462.5	1343.1	1211.2	1169.9	1050.7	884.8	787.5	694.5	540.9	341.0	183.9	722.9	616.3	579.5	545.7	537.7	528.8	515.7	493.9	471.7	438.7	404.0	335.2	279.1	242.5	194.2	150.5	110.3	75.0	45.9	23.7	8.6

Table S136: Harmonic unscaled vibrational frequencies (in cm^{-1}) for S_2 of $HC_{21}N$

method	basis	1	2	3	4	5	6	7	8	9	10	11	12	13	14	15	16	17	18	19	20	21	22	23	24	25	26	27	28	29	30	31	32	33	34	35	36	37	38	39	40	41	42	43
B972	aug-cc-pVTZ	3482.2	2301.9	2225.7	2212.3	2183.4	2166.8	2123.4	2066.3	2051.6	1949.6	1823.7	1703.4	1630.3	1529.3	1384.5	1231.8	1070.6	903.0	729.7	551.8	370.3	187.7	648.1	573.6	539.8	524.6	521.8	517.4	505.0	488.1	467.1	438.4	401.2	340.8	286.4	242.0	195.8	151.7	111.2	75.7	46.3	23.8	8.7
wB97X	aug-cc-pVTZ	3477.4	2419.6	2331.5	2300.0	2237.5	2211.2	2169.5	2121.5	2030.6	1992.2	1695.4	1649.8	1465.6	1342.7	1202.1	1051.7	956.2	885.6	708.0	540.6	345.4	184.1	722.9	618.9	583.0	559.7	554.4	542.0	527.9	504.8	479.6	446.0	408.3	337.7	283.4	243.3	195.5	151.1	110.5	75.1	45.9	23.5	8.4

Table S137: Harmonic unscaled vibrational frequencies (in cm^{-1}) for S_0 of $HC_{21}N$

method	basis	1	2	3	4	5	6	7	8	9	10	11	12	13	14	15	16	17	18	19	20	21	22	23	24	25	26	27	28	29	30	31	32	33	34	35	36	37	38	39	40	41	42	43
B972	aug-cc-pVTZ	3484.7	2342.1	2292.1	2286.5	2266.5	2245.7	2225.2	2177.1	2159.8	2129.0	2091.5	2019.2	1573.9	1473.4	1346.9	1203.8	1049.2	886.1	716.6	542.1	363.8	182.8	662.1	584.4	551.7	542.8	538.0	529.3	517.1	498.7	475.7	445.9	410.5	328.9	289.3	242.8	196.2	151.9	111.3	75.8	46.4	23.8	8.7
wB97X	aug-cc-pVTZ	3478.8	2448.3	2400.4	2393.6	2380.3	2370.1	2347.5	2333.8	2305.5	2260.9	2220.9	2191.8	1481.8	1404.2	1294.4	1163.3	1017.6	861.5	697.7	528.2	354.5	178.0	725.7	625.4	595.0	590.7	577.9	557.9	540.9	517.4	488.6	454.6	417.1	326.0	289.0	242.3	195.5	151.2	110.7	75.3	46.2	23.8	8.9

Table S138: Harmonic unscaled vibrational frequencies (in cm^{-1}) for S_0 of $HC_{23}N$

method	basis	1	2	3	4	5	6	7	8	9	10	11	12	13	14	15	16	17	18	19	20	21	22	23	24	25	26	27	28	29	30	31	32	33	34	35	36	37	38	39	40	41	42	43	44	45	46	47
B972	aug-cc-pVTZ	3484.8	2342.0	2293.4	2281.8	2275.3	2252.1	2225.6	2210.5	2166.0	2135.6	2122.3	2088.0	1997.7	1581.8	1494.0	1382.3	1255.0	1116.7	970.2	817.6	660.0	498.6	334.3	167.8	661.7	573.3	546.4	540.1	536.7	529.8	520.2	507.6	488.4	465.9	437.9	405.8	329.1	294.3	252.3	209.5	168.0	129.3	94.3	64.0	39.1	20.0	7.3
wB97X	aug-cc-pVTZ	3478.9	2448.4	2400.9	2390.9	2388.5	2367.4	2363.1	2333.3	2330.2	2293.3	2252.7	2217.2	2191.5	1486.5	1420.3	1325.0	1209.8	1080.8	941.6	794.9	642.4	485.6	325.6	163.4	725.4	616.2	592.8	589.2	581.0	566.4	546.8	530.3	506.5	478.8	446.9	413.0	326.4	293.9	251.8	208.7	167.3	128.6	93.7	63.6	38.9	20.1	7.5

Table S139: Harmonic unscaled vibrational frequencies (in cm^{-1}) for S_0 of $HC_{25}N$

method	basis	1	2	3	4	5	6	7	8	9	10	11	12	13	14	15	16	17	18	19	20	21	22	23	24	25	26	27	28	29	30	31	32	33	34	35	36	37	38	39	40	41	42	43	44	45	46	47	48	49	50	51
B972	aug-cc-pVTZ	3484.8	2341.9	2291.7	2286.9	2270.9	2261.0	2238.8	2205.3	2198.0	2156.1	2119.2	2110.8	2085.0	1979.0	1588.3	1510.9	1411.4	1297.2	1172.6	1040.2	901.8	758.6	611.6	461.5	309.1	155.1	661.4	587.7	550.0	542.0	538.8	533.8	527.1	515.4	499.5	482.9	460.5	433.5	403.6	330.6	299.3	260.7	221.1	182.2	145.4	111.3	80.9	54.7	33.4	17.1	6.2
wB97X	aug-cc-pVTZ	3478.9	2448.4	2400.4	2395.0	2383.3	2380.8	2357.1	2354.6	2327.4	2320.6	2282.9	2245.8	2214.2	2191.4	1490.1	1433.0	1349.6	1247.7	1132.6	1007.7	875.5	737.5	595.1	449.3	301.0	150.9	725.1	629.2	594.6	591.5	584.9	572.1	554.4	541.1	520.8	498.8	471.9	441.6	410.4	327.3	298.3	259.8	220.0	181.1	144.3	110.4	80.2	54.3	33.2	17.1	6.4

Table S140: Harmonic unscaled vibrational frequencies (in cm^{-1}) for S_0 of $HC_{27}N$

method	basis	1	2	3	4	5	6	7	8	9	10	11	12	13	14	15	16	17	18	19	20	21	22	23	24	25	26	27	28	29	30	31	32	33	34	35	36	37	38	39	40	41	42	43	44	45	46	47	48	49	50	51	52	53	54	55
B972	aug-cc-pVTZ	3484.9	2341.9	2292.4	2283.1	2279.2	2259.2	2246.0	2226.1	2190.0	2182.6	2148.0	2113.0	2091.7	2082.2	1962.9	1593.4	1524.8	1435.6	1332.5	1219.5	1099.1	972.9	842.1	707.4	569.7	429.6	287.5	144.2	661.1	569.4	543.1	539.3	536.8	532.7	526.4	517.4	507.3	491.3	474.3	452.8	427.5	400.3	330.7	302.6	267.4	230.8	194.5	159.5	126.7	96.7	70.1	47.3	28.8	14.8	5.4
wB97X	aug-cc-pVTZ	3478.9	2448.4	2400.7	2393.9	2389.5	2377.5	2370.2	2351.9	2342.4	2325.2	2309.3	2274.0	2240.0	2211.8	2191.2	1493.0	1443.3	1369.8	1279.0	1175.7	1063.0	943.1	817.6	687.7	554.2	418.0	279.8	140.3	724.9	611.7	591.6	589.2	583.4	574.8	561.8	545.5	530.6	511.6	489.8	464.2	436.5	407.7	327.5	301.6	266.6	229.8	193.4	158.4	125.7	95.9	69.5	47.0	28.7	14.8	5.6

Table S141: Harmonic unscaled vibrational frequencies (in cm^{-1}) for S_0 of $HC_{29}N$

method	basis	1	2	3	4	5	6	7	8	9	10	11	12	13	14	15	16	17	18	19	20	21	22	23	24	25	26	27	28	29	30	31	32	33	34	35	36	37	38	39	40	41	42	43	44	45	46	47	48	49	50	51	52	53	54	55	56	57	58	59
B972	aug-cc-pVTZ	3484.9	2341.9	2291.5	2286.8	2273.6	2269.3	2248.1	2230.1	2214.9	2178.4	2165.6	2140.6	2108.7	2080.3	2072.5	1948.9	1597.8	1536.4	1455.9	1362.4	1259.4	1149.3	1033.6	913.4	789.5	662.6	533.1	401.7	268.7	134.7	660.9	580.6	546.2	540.9	538.1	535.2	530.4	523.1	514.1	499.9	487.2	469.8	448.7	424.6	398.8	331.9	306.4	273.7	239.5	205.4	172.2	140.7	111.4	84.8	61.3	41.4	25.2	12.9	4.7
wB97X	aug-cc-pVTZ	3479.0	2448.4	2400.6	2394.6	2390.0	2381.3	2372.1	2359.5	2347.5	2331.3	2323.4	2299.4	2266.3	2235.2	2209.8	2191.2	1495.3	1451.7	1386.4	1305.1	1211.9	1109.7	1000.5	885.8	766.6	644.0	518.5	390.8	261.4	131.0	724.9	621.8	592.5	591.1	586.1	578.9	567.3	552.1	539.0	522.8	505.2	483.7	459.1	432.4	405.9	328.3	304.9	272.6	238.3	204.1	170.9	139.5	110.4	84.0	60.8	41.0	25.0	12.9	4.9

Table S142: Harmonic unscaled vibrational frequencies (in cm^{-1}) for S_1 of $C_{12}N_2$

method	basis	1	2	3	4	5	6	7	8	9	10	11	12	13	14	15	16	17	18	19	20	21	22	23	24	25
B972	aug-cc-pVTZ	2238.6	2135.3	2027.6	1950.2	1354.2	840.5	290.0	2246.4	2110.7	1841.6	1640.1	1103.2	568.9	552.8	509.9	473.8	331.3	171.9	57.3	556.2	507.1	428.7	240.1	109.3	21.0
wB97X	aug-cc-pVTZ	2341.3	2122.7	1932.1	1651.9	1320.6	831.0	287.4	2342.0	2141.0	1632.6	1407.5	1088.1	559.7	585.8	524.9	479.7	331.0	172.4	57.1	587.5	519.3	435.7	236.6	109.4	20.9

Table S143: Harmonic unscaled vibrational frequencies (in cm^{-1}) for S_2 of $C_{12}N_2$

method	basis	1	2	3	4	5	6	7	8	9	10	11	12	13	14	15	16	17	18	19	20	21	22	23	24	25
B972	aug-cc-pVTZ	2244.5	2151.2	2056.1	1958.8	1354.7	840.6	290.0	2252.4	2116.7	1893.3	1636.5	1103.4	568.9	562.8	518.9	480.0	333.8	173.2	57.5	566.0	513.4	431.6	243.1	109.9	21.0
wB97X	aug-cc-pVTZ	2346.3	2156.3	1976.3	1915.4	1324.8	831.4	287.4	2346.9	2159.0	1625.9	1542.6	1088.4	559.7	603.7	544.9	492.2	336.0	174.1	57.2	605.0	530.9	441.6	242.1	110.0	20.6

Table S144: Harmonic unscaled vibrational frequencies (in cm^{-1}) for S_0 of $C_{12}N_2$

method	basis	1	2	3	4	5	6	7	8	9	10	11	12	13	14	15	16	17	18	19	20	21	22	23	24	25
B972	aug-cc-pVTZ	2351.1	2275.1	2163.3	2137.7	1310.5	823.5	282.1	2342.8	2285.3	2211.3	1506.8	1077.2	557.4	586.9	541.7	495.7	325.2	176.9	58.1	589.2	530.5	445.2	250.5	111.5	21.1
wB97X	aug-cc-pVTZ	2452.2	2374.6	2348.7	2229.9	1268.9	805.9	275.6	2448.4	2394.9	2286.4	1438.3	1050.6	545.7	631.5	573.8	506.9	324.9	177.2	58.1	629.3	547.4	451.2	251.7	111.5	21.4

Table S145: Harmonic unscaled vibrational frequencies (in cm^{-1}) for S_1 of $C_{14}N_2$

method	basis	1	2	3	4	5	6	7	8	9	10	11	12	13	14	15	16	17	18	19	20	21	22	23	24	25	26	27	28	29
B972	aug-cc-pVTZ	2263.7	2131.9	2098.6	1966.2	1666.0	1201.9	742.4	254.9	2265.0	2164.5	1951.4	1777.3	1415.5	976.8	501.2	538.8	508.7	491.9	418.3	255.8	136.9	44.3	540.0	502.7	461.8	334.8	195.2	85.4	16.2
wB97X	aug-cc-pVTZ	2370.5	2189.2	2025.9	1647.3	1490.2	1179.9	733.3	252.0	2369.5	2201.4	1952.5	1384.9	1237.7	963.3	489.7	574.9	525.0	502.7	425.2	252.0	137.0	44.2	576.8	517.2	466.3	334.1	196.3	85.3	16.2

Table S146: Harmonic unscaled vibrational frequencies (in cm^{-1}) for S_2 of $C_{14}N_2$

method	basis	1	2	3	4	5	6	7	8	9	10	11	12	13	14	15	16	17	18	19	20	21	22	23	24	25	26	27	28	29
B972	aug-cc-pVTZ	2267.4	2148.2	2105.2	1997.1	1662.1	1202.2	742.5	255.0	2269.5	2171.5	1959.1	1831.9	1416.2	976.9	501.2	545.2	519.1	497.4	420.8	258.4	137.5	44.4	546.6	509.9	466.8	336.8	196.4	85.7	16.2
wB97X	aug-cc-pVTZ	2373.3	2201.6	2080.2	1844.0	1630.6	1181.4	733.4	252.1	2372.4	2215.5	1987.9	1425.4	1362.3	963.2	489.9	584.3	550.8	513.2	430.6	257.3	137.9	44.2	586.4	531.9	477.6	338.2	197.8	85.6	15.9

Table S147: Harmonic unscaled vibrational frequencies (in cm^{-1}) for S_0 of $C_{14}N_2$

method	basis	1	2	3	4	5	6	7	8	9	10	11	12	13	14	15	16	17	18	19	20	21	22	23	24	25	26	27	28	29
B972	aug-cc-pVTZ	2344.3	2293.8	2192.6	2122.4	1530.9	1172.4	728.3	248.1	2346.7	2256.4	2252.5	2125.7	1368.6	956.5	491.4	564.7	542.8	511.6	433.0	264.3	139.1	44.7	566.3	527.8	479.4	327.0	199.3	86.4	16.3
wB97X	aug-cc-pVTZ	2451.5	2377.4	2361.7	2272.2	1455.2	1139.1	712.1	242.1	2447.9	2396.9	2327.6	2223.5	1319.3	933.4	480.5	606.3	583.8	527.3	440.2	265.4	139.1	44.8	607.7	553.9	490.6	326.4	199.7	86.3	16.6

Table S148: Harmonic unscaled vibrational frequencies (in cm^{-1}) for S_1 of $C_{16}N_2$

method	basis	1	2	3	4	5	6	7	8	9	10	11	12	13	14	15	16	17	18	19	20	21	22	23	24	25	26	27	28	29	30	31	32	33
B972	aug-cc-pVTZ	2281.2	2164.8	2101.0	1950.1	1909.3	1462.3	1081.0	664.6	227.4	2278.8	2186.6	2092.8	1711.7	1683.8	1276.3	876.4	447.7	561.1	517.2	510.3	456.2	339.2	213.5	110.3	35.1	564.6	515.5	485.9	415.3	268.3	159.4	68.2	12.8
wB97X	aug-cc-pVTZ	2392.4	2240.1	2033.7	1938.5	1417.0	1349.2	1061.8	655.5	224.2	2390.9	2240.4	2105.6	1659.6	1256.1	1077.6	861.9	432.9	600.0	534.6	521.4	458.5	337.3	214.8	110.0	35.0	605.0	533.3	494.6	421.3	263.9	159.4	67.9	12.7

Table S149: Harmonic unscaled vibrational frequencies (in cm^{-1}) for S_2 of $C_{16}N_2$

method	basis	1	2	3	4	5	6	7	8	9	10	11	12	13	14	15	16	17	18	19	20	21	22	23	24	25	26	27	28	29	30	31	32	33
B972	aug-cc-pVTZ	2283.9	2169.1	2121.0	1957.3	1942.2	1462.9	1081.1	664.7	227.4	2281.7	2195.0	2096.6	1768.7	1680.0	1276.6	876.4	447.8	566.5	526.2	514.8	460.3	340.8	214.6	110.6	35.2	570.1	520.8	491.4	417.3	270.7	160.0	68.3	12.8
wB97X	aug-cc-pVTZ	2393.6	2246.1	2083.9	1986.5	1785.2	1416.8	1063.4	655.4	224.2	2391.9	2248.4	2130.7	1641.7	1281.8	1229.9	862.0	433.6	607.1	558.7	531.6	468.6	340.9	216.1	110.4	34.9	612.6	545.4	507.2	425.9	268.9	160.4	67.9	12.4

Table S150: Harmonic unscaled vibrational frequencies (in cm^{-1}) for S_0 of $C_{16}N_2$

method	basis	1	2	3	4	5	6	7	8	9	10	11	12	13	14	15	16	17	18	19	20	21	22	23	24	25	26	27	28	29	30	31	32	33
B972	aug-cc-pVTZ	2343.3	2290.0	2233.7	2116.0	2087.2	1411.9	1058.0	652.5	221.4	2345.7	2279.6	2225.2	2176.7	1548.4	1244.3	859.5	439.3	581.9	547.7	527.0	470.6	330.5	216.8	111.3	35.3	586.0	536.1	504.1	427.8	275.4	161.4	68.6	12.8
wB97X	aug-cc-pVTZ	2449.8	2392.6	2351.8	2310.5	2218.9	1355.9	1029.5	637.5	215.9	2449.0	2390.5	2358.5	2261.1	1466.9	1204.8	838.6	429.1	622.1	592.1	545.0	480.1	329.3	217.3	111.3	35.4	627.5	567.0	520.0	435.2	276.5	161.6	68.6	13.1

Table S151: Harmonic unscaled vibrational frequencies (in cm^{-1}) for S_1 of $C_{18}N_2$

method	basis	1	2	3	4	5	6	7	8	9	10	11	12	13	14	15	16	17	18	19	20	21	22	23	24	25	26	27	28	29	30	31	32	33	34	35	36	37
B972	aug-cc-pVTZ	2292.6	2196.2	2084.7	2072.5	1856.4	1697.6	1334.9	982.0	601.5	205.2	2290.9	2190.8	2148.2	1948.6	1645.0	1498.1	1162.3	794.5	404.4	549.8	513.9	506.9	474.7	407.3	277.3	178.6	90.6	28.6	548.5	512.7	497.0	445.5	339.6	228.3	132.1	55.7	10.4
wB97X	aug-cc-pVTZ	2409.0	2273.4	2140.5	1975.8	1665.8	1306.2	1246.5	964.2	592.2	201.7	2407.7	2270.8	2154.1	1955.3	1442.0	1144.2	931.4	775.2	384.9	593.0	535.7	522.0	483.1	413.5	272.7	178.3	90.2	28.5	590.9	535.1	508.1	448.4	337.1	230.0	131.8	55.4	10.3

Table S152: Harmonic unscaled vibrational frequencies (in cm^{-1}) for S_2 of $C_{18}N_2$

method	basis	1	2	3	4	5	6	7	8	9	10	11	12	13	14	15	16	17	18	19	20	21	22	23	24	25	26	27	28	29	30	31	32	33	34	35	36	37
B972	aug-cc-pVTZ	2294.7	2200.7	2095.6	2086.0	1891.2	1693.6	1335.1	982.0	601.5	205.2	2292.7	2199.2	2153.2	1955.4	1704.7	1498.7	1162.5	794.5	404.4	553.4	522.0	511.8	479.1	409.2	279.4	179.2	90.8	28.6	552.1	519.2	501.4	449.0	341.0	229.3	132.4	55.7	10.4
wB97X	aug-cc-pVTZ	2408.7	2276.7	2154.4	2045.2	1741.1	1646.4	1301.2	965.4	591.9	201.8	2407.3	2273.8	2176.0	1995.5	1444.9	1154.2	1105.7	777.6	386.7	597.3	556.7	535.0	493.3	417.9	277.5	179.5	90.4	28.4	595.3	550.0	518.5	457.6	340.3	231.1	132.3	55.4	10.2

Table S153: Harmonic unscaled vibrational frequencies (in cm^{-1}) for S_0 of $C_{18}N_2$

method	basis	1	2	3	4	5	6	7	8	9	10	11	12	13	14	15	16	17	18	19	20	21	22	23	24	25	26	27	28	29	30	31	32	33	34	35	36	37
B972	aug-cc-pVTZ	2344.4	2273.9	2262.9	2163.6	2056.8	1561.9	1300.2	962.8	590.9	199.8	2343.4	2291.4	2217.0	2195.6	2108.0	1445.3	1137.4	779.9	397.1	566.5	542.2	524.8	489.5	419.1	283.1	180.1	91.0	28.7	565.2	536.5	512.2	458.0	330.2	230.9	133.0	55.9	10.4
wB97X	aug-cc-pVTZ	2449.0	2397.4	2344.2	2341.3	2252.0	1475.3	1254.7	937.3	576.7	194.7	2449.3	2382.0	2378.9	2296.3	2215.1	1383.0	1103.5	760.7	387.5	609.2	589.8	551.4	504.3	426.3	283.9	179.9	90.6	28.7	607.3	574.7	530.3	468.0	328.6	231.0	132.6	55.7	10.7

Table S154: Harmonic unscaled vibrational frequencies (in cm^{-1}) for S_1 of $C_{20}N_2$

method	basis	1	2	3	4	5	6	7	8	9	10	11	12	13	14	15	16	17	18	19	20	21	22	23	24	25	26	27	28	29	30	31	32	33	34	35	36	37	38	39	40	41
B972	aug-cc-pVTZ	2301.2	2212.2	2141.0	2042.1	1946.9	1807.5	1526.4	1228.2	899.3	549.2	187.0	2300.6	2199.2	2165.7	2075.4	1707.8	1578.8	1381.3	1066.6	726.4	368.6	566.2	518.9	515.7	490.4	441.9	342.1	241.0	151.1	75.6	23.7	563.8	519.0	508.0	468.1	405.2	285.6	194.9	111.0	46.2	8.6
wB97X	aug-cc-pVTZ	2421.2	2297.8	2200.4	1988.1	1932.9	1460.8	1215.2	1179.1	882.2	539.7	183.2	2420.3	2295.8	2194.8	2093.2	1669.9	1341.5	1048.6	809.7	693.9	342.7	611.7	544.8	530.6	501.1	444.6	338.5	242.9	150.6	75.2	23.7	608.1	544.7	519.7	474.4	411.0	280.6	194.3	110.5	46.0	8.6

Table S155: Harmonic unscaled vibrational frequencies (in cm^{-1}) for S_2 of $C_{20}N_2$

method	basis	1	2	3	4	5	6	7	8	9	10	11	12	13	14	15	16	17	18	19	20	21	22	23	24	25	26	27	28	29	30	31	32	33	34	35	36	37	38	39	40	41
B972	aug-cc-pVTZ	2302.5	2217.2	2144.3	2064.1	1953.4	1844.0	1527.0	1228.3	899.4	549.2	186.9	2301.8	2203.0	2176.3	2078.0	1703.9	1640.9	1381.5	1066.8	726.5	368.6	569.2	525.3	520.3	495.0	444.8	343.3	241.9	151.5	75.7	23.7	566.7	524.5	512.1	471.8	406.8	287.5	195.5	111.2	46.3	8.6
wB97X	aug-cc-pVTZ	2420.1	2299.1	2208.2	2040.0	1992.8	1706.6	1463.9	1199.9	882.9	539.3	183.4	2419.1	2296.2	2204.3	2122.5	1649.9	1341.0	1050.8	983.2	704.8	346.3	614.8	560.9	545.5	512.5	452.5	341.4	243.7	151.2	75.2	23.5	611.0	557.5	529.8	483.8	415.1	285.1	195.6	110.8	45.9	8.4

Table S156: Harmonic unscaled vibrational frequencies (in cm^{-1}) for S_0 of $C_{20}N_2$

method	basis	1	2	3	4	5	6	7	8	9	10	11	12	13	14	15	16	17	18	19	20	21	22	23	24	25	26	27	28	29	30	31	32	33	34	35	36	37	38	39	40	41
B972	aug-cc-pVTZ	2344.1	2281.5	2253.6	2202.4	2101.5	2030.7	1471.5	1200.8	882.7	539.8	182.1	2342.7	2290.1	2247.3	2168.2	2152.2	1572.4	1344.5	1045.8	713.6	362.3	580.0	544.1	531.7	505.8	452.2	332.3	242.9	151.9	75.8	23.8	577.4	540.5	522.0	480.9	415.9	290.6	196.1	111.4	46.3	8.6
wB97X	aug-cc-pVTZ	2449.0	2396.0	2364.2	2338.4	2284.6	2212.2	1403.9	1161.9	859.4	526.5	177.3	2449.0	2390.8	2373.7	2326.4	2244.8	1481.6	1293.6	1015.8	695.7	353.3	622.3	592.9	561.3	524.4	461.1	330.0	242.9	151.3	75.4	23.8	618.8	581.5	541.1	494.2	422.8	290.9	195.7	110.9	46.2	8.9

Table S157: Harmonic unscaled vibrational frequencies (in cm^{-1}) for S_1 of $C_{22}N_2$

method	basis	1	2	3	4	5	6	7	8	9	10	11	12	13	14	15	16	17	18	19	20	21	22	23	24	25	26	27	28	29	30	31	32	33	34	35	36	37	38	39	40	41	42	43	44	45
B972	aug-cc-pVTZ	2308.3	2218.4	2175.5	2067.5	2009.8	1762.5	1715.7	1418.8	1136.6	829.2	505.2	171.6	2307.9	2216.8	2154.4	2131.9	1945.2	1549.2	1513.4	1281.5	985.0	669.0	338.5	554.1	518.1	512.9	497.8	460.4	400.3	291.4	208.7	129.1	64.0	20.0	555.6	518.1	508.7	482.6	434.5	342.4	251.2	167.5	94.3	39.0	7.3

Table S158: Harmonic unscaled vibrational frequencies (in cm^{-1}) for S_2 of $C_{22}N_2$

method	basis	1	2	3	4	5	6	7	8	9	10	11	12	13	14	15	16	17	18	19	20	21	22	23	24	25	26	27	28	29	30	31	32	33	34	35	36	37	38	39	40	41	42	43	44	45
B972	aug-cc-pVTZ	2309.0	2223.2	2179.7	2069.9	2032.6	1800.6	1711.7	1419.0	1136.7	829.3	505.2	171.6	2308.7	2219.8	2166.6	2134.4	1951.5	1577.9	1549.5	1281.7	985.2	669.0	338.6	556.1	523.8	518.4	501.4	463.6	401.8	293.2	209.2	129.3	64.0	20.0	557.6	523.3	512.6	486.3	437.1	343.4	252.0	167.9	94.4	39.1	7.3
wB97X	aug-cc-pVTZ	2428.5	2317.3	2240.2	2140.1	2014.9	1680.1	1649.9	1370.6	1111.0	812.8	495.1	168.0	2427.8	2315.9	2233.1	2156.5	2001.6	1478.4	1248.1	966.5	869.4	638.8	310.3	601.1	561.9	548.8	519.7	475.5	410.2	290.4	209.1	128.8	63.5	19.8	603.6	560.8	536.8	502.2	444.8	340.9	253.9	167.5	93.8	38.7	7.1

Table S159: Harmonic unscaled vibrational frequencies (in cm^{-1}) for S_0 of $C_{22}N_2$

method	basis	1	2	3	4	5	6	7	8	9	10	11	12	13	14	15	16	17	18	19	20	21	22	23	24	25	26	27	28	29	30	31	32	33	34	35	36	37	38	39	40	41	42	43	44	45
B972	aug-cc-pVTZ	2343.3	2289.8	2233.7	2231.7	2142.7	2008.0	1580.6	1380.3	1113.7	814.6	496.7	167.2	2343.0	2280.6	2268.6	2189.7	2142.7	2096.1	1492.5	1252.4	967.2	657.5	333.0	565.2	541.2	532.3	510.6	471.3	410.6	295.6	209.5	129.4	64.0	20.0	566.8	539.1	523.1	495.3	443.8	332.4	252.6	168.0	94.4	39.1	7.3
wB97X	aug-cc-pVTZ	2449.0	2391.5	2379.9	2334.0	2313.4	2238.8	1486.4	1324.4	1079.3	793.0	484.2	162.8	2448.7	2396.2	2366.3	2350.1	2274.8	2210.0	1420.1	1208.8	939.8	640.7	324.5	606.5	591.0	569.0	529.8	484.2	417.9	295.8	209.0	128.8	63.7	20.1	608.6	583.7	550.4	512.5	452.9	330.0	252.4	167.4	93.8	38.9	7.5

Table S160: Harmonic unscaled vibrational frequencies (in cm^{-1}) for S_1 of $C_{24}N_2$

method	basis	1	2	3	4	5	6	7	8	9	10	11	12	13	14	15	16	17	18	19	20	21	22	23	24	25	26	27	28	29	30	31	32	33	34	35	36	37	38	39	40	41	42	43	44	45	46	47	48	49
wB97X	aug-cc-pVTZ	2436.3	2335.3	2261.5	2184.5	1975.8	1919.9	1484.7	1286.8	1168.1	1030.9	752.6	457.9	154.7	2435.7	2334.9	2259.2	2173.3	2086.4	1671.2	1394.4	1164.9	895.8	660.3	525.5	266.0	613.4	558.4	534.5	519.7	482.1	434.3	338.8	262.5	181.0	110.6	54.3	17.1	617.3	558.4	536.5	505.2	464.5	404.2	291.9	219.0	144.4	80.4	33.1	6.3

Table S161: Harmonic unscaled vibrational frequencies (in cm^{-1}) for S_2 of $C_{24}N_2$

method	basis	1	2	3	4	5	6	7	8	9	10	11	12	13	14	15	16	17	18	19	20	21	22	23	24	25	26	27	28	29	30	31	32	33	34	35	36	37	38	39	40	41	42	43	44	45	46	47	48	49
wB97X	aug-cc-pVTZ	2434.7	2333.0	2262.5	2193.2	2018.6	1993.8	1656.6	1488.4	1285.7	1033.0	752.6	457.5	155.0	2434.1	2332.6	2258.5	2180.8	2117.3	1651.1	1394.4	1164.5	895.6	772.3	575.7	276.9	614.2	566.7	553.8	528.4	491.7	440.9	341.5	262.9	181.7	110.8	54.3	16.9	618.2	566.4	545.8	515.9	470.9	407.9	295.7	220.7	144.8	80.4	33.0	6.1

Table S162: Harmonic unscaled vibrational frequencies (in cm^{-1}) for S_0 of $C_{24}N_2$

method	basis	1	2	3	4	5	6	7	8	9	10	11	12	13	14	15	16	17	18	19	20	21	22	23	24	25	26	27	28	29	30	31	32	33	34	35	36	37	38	39	40	41	42	43	44	45	46	47	48	49
B972	aug-cc-pVTZ	2342.9	2289.7	2255.9	2211.7	2178.4	2091.6	1988.4	1509.6	1295.1	1037.4	756.1	459.9	154.6	2342.9	2282.3	2266.7	2219.9	2134.3	2119.3	1587.2	1409.7	1170.1	899.0	609.5	308.1	576.6	542.6	536.2	518.4	486.7	439.7	333.9	261.3	182.2	111.4	54.8	17.1	579.3	541.7	529.6	506.9	466.4	408.4	300.9	221.2	145.4	81.0	33.4	6.2
wB97X	aug-cc-pVTZ	2449.0	2389.8	2386.0	2337.3	2330.5	2266.5	2208.1	1433.0	1247.0	1006.2	735.8	448.1	150.5	2448.7	2397.1	2367.9	2359.9	2302.2	2233.8	1490.1	1349.2	1131.4	873.8	593.6	300.1	617.0	592.9	575.1	537.4	502.9	448.2	331.0	260.9	181.4	110.7	54.4	17.1	620.6	587.7	558.7	527.1	477.8	415.4	300.8	220.5	144.7	80.4	33.2	6.4

Table S163: Harmonic unscaled vibrational frequencies (in cm^{-1}) for S_1 of $C_{26}N_2$

method	basis	1	2	3	4	5	6	7	8	9	10	11	12	13	14	15	16	17	18	19	20	21	22	23	24	25	26	27	28	29	30	31	32	33	34	35	36	37	38	39	40	41	42	43	44	45	46	47	48	49	50	51	52	53
B972	aug-cc-pVTZ	2318.7	2232.9	2189.2	2160.2	2054.2	1945.5	1726.6	1683.6	1475.1	1243.1	987.8	717.0	435.1	147.4	2318.1	2234.7	2192.7	2116.1	2114.6	1942.4	1582.6	1386.5	1362.4	1117.8	853.6	577.3	290.6	560.0	522.1	515.6	509.7	486.6	450.2	395.2	301.2	230.7	159.6	96.9	47.4	14.7	558.9	522.2	513.5	499.5	469.0	425.7	344.0	267.6	194.4	126.9	70.2	28.8	5.4

Table S164: Harmonic unscaled vibrational frequencies (in cm^{-1}) for S_2 of $C_{26}N_2$

method	basis	1	2	3	4	5	6	7	8	9	10	11	12	13	14	15	16	17	18	19	20	21	22	23	24	25	26	27	28	29	30	31	32	33	34	35	36	37	38	39	40	41	42	43	44	45	46	47	48	49	50	51	52	53
wB97X	aug-cc-pVTZ	2439.1	2346.2	2281.3	2223.4	2134.7	1999.3	1652.3	1636.0	1412.6	1207.5	964.6	700.4	425.2	143.8	2438.6	2346.1	2280.1	2214.0	2142.0	2005.9	1496.2	1317.2	1089.7	835.0	695.8	513.6	244.6	607.7	569.2	554.7	538.0	507.3	463.5	404.6	299.3	230.4	159.0	96.2	47.0	14.6	605.9	569.1	547.9	521.7	485.4	435.0	340.7	270.1	194.1	126.2	69.7	28.5	5.3

Table S165: Harmonic unscaled vibrational frequencies (in cm^{-1}) for S_0 of $C_{26}N_2$

method	basis	1	2	3	4	5	6	7	8	9	10	11	12	13	14	15	16	17	18	19	20	21	22	23	24	25	26	27	28	29	30	31	32	33	34	35	36	37	38	39	40	41	42	43	44	45	46	47	48	49	50	51	52	53
B972	aug-cc-pVTZ	2342.8	2283.9	2272.0	2208.2	2191.4	2127.0	1971.4	1592.6	1434.1	1217.3	970.4	705.2	428.2	143.7	2342.6	2288.5	2251.5	2243.6	2168.5	2098.0	2087.8	1523.7	1330.7	1096.7	839.6	567.9	286.6	567.5	540.4	535.5	521.9	498.0	458.3	404.8	304.2	230.9	159.6	96.8	47.4	14.8	566.4	539.5	529.3	510.4	479.1	433.1	333.6	268.0	194.5	126.8	70.2	28.8	5.4
wB97X	aug-cc-pVTZ	2448.8	2395.0	2380.2	2356.1	2327.8	2292.4	2229.5	1493.1	1369.6	1174.8	941.7	686.2	416.9	139.9	2448.7	2395.2	2380.5	2354.3	2325.7	2259.4	2206.6	1443.3	1278.4	1061.8	816.1	552.9	279.1	609.7	591.6	578.4	549.4	517.1	469.7	412.3	303.9	230.3	158.7	96.2	47.1	14.8	608.2	586.9	565.1	530.7	494.6	441.9	330.7	267.7	193.7	126.0	69.7	28.8	5.6

Table S166: Harmonic unscaled vibrational frequencies (in cm^{-1}) for S_1 of $C_{28}N_2$

method	basis	1	2	3	4	5	6	7	8	9	10	11	12	13	14	15	16	17	18	19	20	21	22	23	24	25	26	27	28	29	30	31	32	33	34	35	36	37	38	39	40	41	42	43	44	45	46	47	48	49	50	51	52	53	54	55	56	57
B972	aug-cc-pVTZ	2322.5	2241.1	2187.8	2177.9	2106.9	1941.0	1914.4	1649.6	1595.1	1393.7	1169.4	926.6	671.3	406.8	137.7	2321.9	2239.2	2205.4	2151.8	2094.8	2048.5	1730.2	1496.5	1324.7	1284.3	1049.9	799.9	540.2	271.3	570.8	525.5	518.5	514.8	494.7	466.1	422.5	344.8	274.1	205.3	140.7	84.8	41.3	12.8	569.1	525.4	517.4	505.9	478.3	446.7	393.5	305.1	239.5	172.2	111.4	61.4	25.1	4.7

Table S167: Harmonic unscaled vibrational frequencies (in cm^{-1}) for S_2 of $C_{28}N_2$

method	basis	1	2	3	4	5	6	7	8	9	10	11	12	13	14	15	16	17	18	19	20	21	22	23	24	25	26	27	28	29	30	31	32	33	34	35	36	37	38	39	40	41	42	43	44	45	46	47	48	49	50	51	52	53	54	55	56	57
B972	aug-cc-pVTZ	2322.5	2242.8	2190.6	2186.1	2108.5	1947.0	1938.7	1691.2	1595.5	1393.9	1169.5	926.7	671.4	406.9	137.7	2321.9	2241.0	2210.4	2153.7	2107.7	2050.3	1726.3	1496.8	1395.0	1284.4	1049.9	800.0	540.2	271.4	571.7	522.9	518.7	498.0	468.5	424.3	345.5	274.7	205.6	140.9	84.9	41.4	12.8	570.0	528.5	528.3	521.1	508.9	481.3	448.7	394.6	306.5	240.0	172.4	111.6	61.3	25.2	4.5
wB97X	aug-cc-pVTZ	2442.2	2357.0	2299.0	2243.8	2185.3	2014.4	1990.6	1621.5	1501.5	1342.3	1136.5	904.1	654.8	397.1	134.1	2441.8	2356.9	2299.1	2239.7	2166.2	2113.6	1650.6	1427.5	1244.2	1023.2	782.9	638.2	454.2	212.3	618.4	573.8	557.2	545.1	518.1	481.1	431.6	340.8	276.6	205.0	140.1	84.2	41.0	12.8	615.8	573.6	552.9	529.1	496.8	459.1	402.5	303.0	238.9	171.6	110.8	60.8	24.8	4.6

Table S168: Harmonic unscaled vibrational frequencies (in cm^{-1}) for S_0 of $C_{28}N_2$

method	basis	1	2	3	4	5	6	7	8	9	10	11	12	13	14	15	16	17	18	19	20	21	22	23	24	25	26	27	28	29	30	31	32	33	34	35	36	37	38	39	40	41	42	43	44	45	46	47	48	49	50	51	52	53	54	55	56	57
B972	aug-cc-pVTZ	2342.7	2282.9	2273.8	2232.2	2172.2	2159.5	2084.6	1956.7	1535.3	1360.8	1147.1	911.0	660.6	400.5	134.3	2342.4	2289.0	2261.6	2235.6	2197.5	2120.7	2078.4	1596.9	1454.6	1257.5	1031.2	787.3	531.5	267.9	576.9	541.2	537.3	527.0	506.7	474.5	429.3	334.5	274.2	205.2	140.6	84.7	41.3	12.8	575.2	540.8	532.4	516.5	489.5	453.7	402.7	307.8	239.5	172.0	111.3	61.3	25.1	4.7
wB97X	aug-cc-pVTZ	2448.7	2397.0	2374.7	2370.5	2325.6	2315.3	2253.3	2205.4	1451.8	1304.7	1108.6	884.3	642.6	389.8	130.7	2448.6	2391.9	2388.9	2349.5	2345.0	2283.9	2226.0	1495.4	1386.3	1211.2	999.2	765.2	517.3	260.8	619.0	592.6	582.1	556.3	528.3	488.6	438.0	331.3	273.8	204.4	139.8	84.2	41.1	12.9	616.7	589.1	570.4	536.7	508.3	464.3	410.0	307.4	238.9	171.2	110.7	60.9	25.1	4.9

Table S169: Harmonic unscaled vibrational frequencies (in cm^{-1}) for S_1 of $C_{30}N_2$

method	basis	1	2	3	4	5	6	7	8	9	10	11	12	13	14	15	16	17	18	19	20	21	22	23	24	25	26	27	28	29	30	31	32	33	34	35	36	37	38	39	40	41	42	43	44	45	46	47	48	49	50	51	52	53	54	55	56	57	58	59	60	61
B972	aug-cc-pVTZ	2325.6	2246.5	2205.4	2165.2	2143.7	2043.6	1884.5	1733.2	1618.8	1514.6	1319.8	1103.5	872.4	631.1	381.9	129.1	2325.1	2245.1	2207.3	2178.8	2100.0	2073.3	1940.3	1605.6	1420.4	1264.3	1213.7	989.5	752.4	507.5	254.2	561.3	517.2	500.2	474.6	441.4	391.0	308.1	247.2	183.6	125.0	75.0	36.4	11.3	562.4	525.9	525.6	517.0	513.3	509.7	489.9	460.5	418.5	344.8	279.6	215.0	153.4	98.7	54.1	22.1	4.1

Table S170: Harmonic unscaled vibrational frequencies (in cm^{-1}) for S_2 of $C_{30}N_2$

method	basis	1	2	3	4	5	6	7	8	9	10	11	12	13	14	15	16	17	18	19	20	21	22	23	24	25	26	27	28	29	30	31	32	33	34	35	36	37	38	39	40	41	42	43	44	45	46	47	48	49	50	51	52	53	54	55	56	57	58	59	60	61
B972	aug-cc-pVTZ	2325.4	2248.0	2208.4	2173.8	2145.4	2045.2	1909.1	1729.2	1661.2	1514.9	1319.9	1103.5	872.5	631.1	382.0	129.1	2325.0	2246.1	2212.9	2181.2	2101.5	2086.4	1946.2	1606.0	1420.5	1335.9	1213.9	989.5	752.6	507.5	254.4	561.9	522.0	516.8	502.8	477.2	443.2	392.1	309.4	247.6	183.8	125.1	75.0	36.4	11.3	563.0	528.0	527.8	520.2	512.6	492.7	462.6	420.0	345.4	280.0	215.4	153.7	98.7	54.2	22.2	4.3
wB97X	aug-cc-pVTZ	2444.3	2365.5	2315.0	2261.3	2213.5	2132.6	1993.3	1650.5	1608.4	1439.1	1274.2	1072.5	850.4	614.6	372.4	125.6	2444.0	2365.2	2315.3	2260.1	2203.4	2132.7	2008.2	1505.7	1363.7	1176.9	963.6	737.9	594.9	402.0	179.8	607.3	575.9	558.5	547.0	523.0	492.4	453.5	400.1	305.5	246.0	182.6	124.0	74.2	36.0	11.2	609.0	576.1	556.1	538.3	511.6	474.8	427.1	340.3	281.8	214.4	152.4	97.8	53.5	21.9	4.1

Table S171: Harmonic unscaled vibrational frequencies (in cm^{-1}) for S_0 of $C_{30}N_2$

method	basis	1	2	3	4	5	6	7	8	9	10	11	12	13	14	15	16	17	18	19	20	21	22	23	24	25	26	27	28	29	30	31	32	33	34	35	36	37	38	39	40	41	42	43	44	45	46	47	48	49	50	51	52	53	54	55	56	57	58	59	60	61
B972	aug-cc-pVTZ	2342.6	2287.7	2262.8	2251.2	2187.9	2153.7	2115.1	1943.9	1600.6	1472.1	1291.9	1083.7	858.3	621.2	376.1	126.0	2342.4	2285.3	2274.2	2221.9	2219.3	2151.7	2081.9	2060.5	1545.3	1386.5	1190.5	972.7	741.0	499.5	251.5	566.3	539.8	536.7	527.0	509.8	484.1	447.8	400.1	310.3	247.0	183.3	124.7	74.8	36.4	11.3	567.4	539.5	532.6	520.7	500.2	468.0	424.8	334.5	279.3	214.8	153.1	98.5	54.1	22.1	4.1
wB97X	aug-cc-pVTZ	2448.7	2396.6	2381.0	2369.4	2334.7	2323.9	2276.4	2222.9	1497.2	1400.3	1242.0	1048.4	833.3	604.2	366.0	122.6	2448.6	2393.9	2387.8	2360.5	2345.5	2306.0	2247.9	2204.3	1458.7	1326.8	1148.6	943.0	720.1	486.0	244.7	607.6	591.6	582.6	562.1	530.4	501.6	458.2	407.6	309.5	246.1	182.2	123.8	74.2	36.2	11.4	609.1	588.6	574.0	548.5	520.4	481.6	433.1	331.1	278.7	213.7	152.1	97.7	53.6	22.1	4.3
